# An improved manta ray foraging optimization algorithm

**DOI:** 10.1038/s41598-024-59960-1

**Published:** 2024-05-05

**Authors:** Pengju Qu, Qingni Yuan, Feilong Du, Qingyang Gao

**Affiliations:** 1 Key Laboratory of Advanced Manufacturing Technology of the Ministry of Education, Guizhou University, Guiyang, China; 2 Engineering Training Center, Guizhou Institute of Technology, Guiyang, China

**Keywords:** Metaheuristic, Improved manta ray foraging optimization, Bidirectional search strategy, Levy flight, Mechanical engineering, Electrical and electronic engineering

## Abstract

The Manta Ray Foraging Optimization Algorithm (MRFO) is a metaheuristic algorithm for solving real-world problems. However, MRFO suffers from slow convergence precision and is easily trapped in a local optimal. Hence, to overcome these deficiencies, this paper proposes an Improved MRFO algorithm (IMRFO) that employs Tent chaotic mapping, the bidirectional search strategy, and the Levy flight strategy. Among these strategies, Tent chaotic mapping distributes the manta ray more uniformly and improves the quality of the initial solution, while the bidirectional search strategy expands the search area. The Levy flight strategy strengthens the algorithm’s ability to escape from local optimal. To verify IMRFO’s performance, the algorithm is compared with 10 other algorithms on 23 benchmark functions, the CEC2017 and CEC2022 benchmark suites, and five engineering problems, with statistical analysis illustrating the superiority and significance of the difference between IMRFO and other algorithms. The results indicate that the IMRFO outperforms the competitor optimization algorithms.

## Introduction

Under certain constraints, the optimization problem involves finding the objective function's best fitness. As real-world optimization problems have become more complex in recent years, deterministic algorithms have encountered significant performance limitations^1^. On the contrary, stochastic algorithms perform much better at solving these problems. Besides, the Metaheuristic (MH) algorithms have proven highly effective in tackling complex problems^2^. This is because MH algorithms generate a feasible solution space with a stochastic algorithm, search the space for solutions in each iteration, evaluate individual fitness through the fitness function, and perform updates to produce the optimal solution^3^. The MH algorithms have shown advantages in many fields, including global optimization^4,5^, feature selection^6,7^, sentiment classification^8–10^, and case forecasting^11^. Considering the no free lunch (NFL) theorem, no algorithm can perform well on every optimization problem^12^, it is significant to study the MH algorithms.

Several MH algorithms were inspired by the various behaviors or patterns formed by the natural evolution of organisms. For instance, the Genetic Algorithm (GA)^13^ adopts the Darwinian biological evolution, natural selection, and the genetic mechanism of the biological evolution process. The particle swarm optimization algorithm (PSO)^14^ originated from biologists' observations of and studies of birds' foraging behavior. Bacterial Foraging Optimization (BFO)^15^ Mimics the eating habits of E. coli in the human gut. Glowworm Swarm Optimization (GSO)^16^ is inspired by the behavior of fireflies that are attracted and moved by light during their life habits of foraging, courtship, and vigilance. The Cuckoo Search Algorithm (CS)^17^ is inspired by a parasitic habit, i.e., some cuckoos lay eggs in other birds' nests until the young hatch. Gray wolf prey hunting activities inspired the Grey Wolf Optimizer (GWO)^18^. The Whale Optimization Algorithm (WOA)^19^ is inspired by humpback whale hunting behavior, while Pigeon-inspired Optimization (PIO)^20^ is inspired by pigeons' intelligence and collaborative abilities in spatial navigation and social behavior. The Slime Mould algorithm (SMA) Group^21^ is inspired by the behavior capable of efficiently finding food and establishing communication networks in a space environment. Furthermore, the Marine Predators Algorithm (MPA)^22^ is inspired by predator behavior in Marine life. The Bald Eagle Search (BES) algorithm^23^ is inspired by changes in the predatory behavior of bald eagles, and the Grasshopper Optimization Algorithm (GOA)^24^ is inspired by the mobile foraging behavior of grasshoppers. The Mayfly Algorithm (MA)^25^ is inspired by the mayfly's short life span and genetic behavior. The multiple population hybrid equalization optimizer (MHEO)^26^ is inspired by the population distributed mechanism.

**Figure 1:**
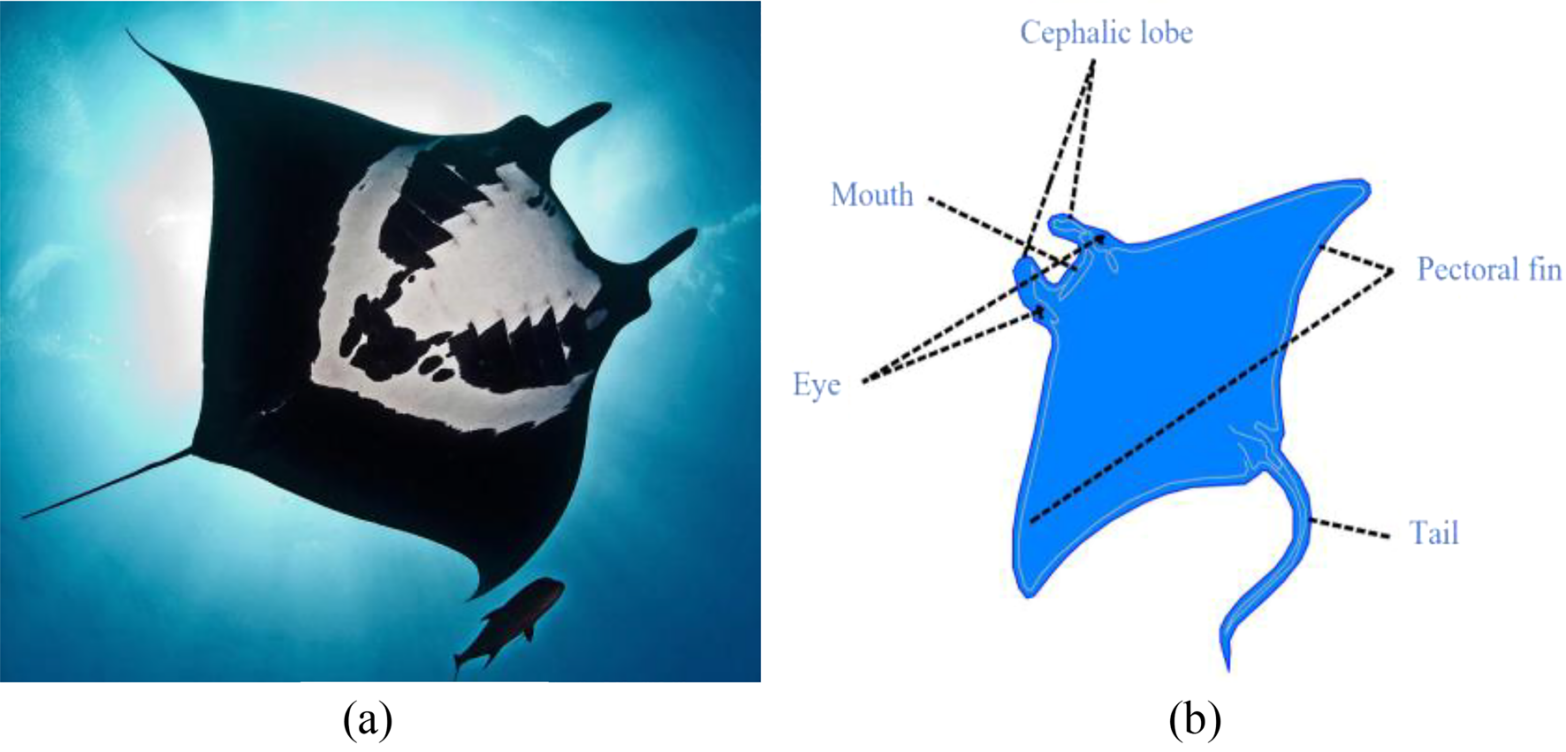
(**a**) Picture a manta ray, and (**b**) itsbody structure.

The Manta Ray Foraging Optimization algorithm (MRFO)^27^ is a meta-heuristic algorithm that imitates the chain, cyclone, and somersault foraging modes of manta rays in the group foraging process. Figure 1a depicts a manta ray, and Fig. 1b illustrates its body structure. In nature, manta rays have three main parts during their foraging process. Firstly, the mantas line up, forming an orderly chain. The smaller male rests on the back of the female and moves in tandem with the beat of her pectoral fins. Thus, this mechanism allows them to maximize their foraging efficiency. Secondly, manta rays cluster as cyclones to filter the prey layer when plankton concentrations are high. Finally, somersault foraging is conducted if the densest food spot is found. Because the somersault phase coexists with randomness and periodicity, it helps the mantas control their food intake. MRFO has certain advantages, such as fast convergence speed and a strong ability to search for global optimal, which is widely used in various fields, such as economic load dispatching problems^28^, image segmentation problems^29^, minimization of energy consumption^30^, and radial distribution networks^31^. Although compared to other algorithms, MRFO shows good performance, defects emerge due to the lack of disturbance in the exploration and exploitation phase, such as low solving precision and easily trapped into local optimal.

Spurred by the above deficiencies, this paper extends the MRFO by incorporating Tent chaotic mapping, bidirectional search, and the Levy flight strategy. The bidirectional search strategy aims to start the search from the starting point and select one node from both directions for expansion at a time. This strategy reduces the search space searching from both directions simultaneously and finds a solution faster. It has been employed in algorithm improvement^32^, tourism demand forecasting^33^, and Model for Web Crawling^34^. However, it has never been used in the MRFO algorithm. In the IMRFO algorithm, the bidirectional search strategy not only searches along the direction of the fitness value decrease but also in the opposite direction. In the algorithm-solving process, especially in some multimodal and composite functions, the improving strategy can prevent the algorithm from being trapped into the local optimal and enhance global search ability. Moreover, we introduce the Levy flight strategy into MRFO to help the algorithm jump out from local optimal during exploitation.

The contributions of this paper are as follows. (1) During the algorithm’s initialization phase, Tent chaos mapping provides the initial solution with better ergodicity, uniformity, and randomness in the search space. (2) After the cyclone foraging phase, the bidirectional search strategy helps the algorithm search bidirectionally, which can enlarge the search scope and help the manta ray jump out of local optimal. (3) During the somersault foraging stage, the Levy flight strategy strengthens the algorithm’s ability to escape from local optimal. (4) The proposed algorithm is evaluated on 23 benchmark functions, the CEC2017 and CEC2022 benchmark suites, and five engineering problems. (5) Various evaluation measurements illustrate the superiority of our proposed IMRFO.

The remainder of this paper is organized as follows. "MRFO algorithm" section briefly presents MRFO. "Improved strategy for MRFO" section introduces three improvement strategies and the proposed IMRFO. "Experimental results and discussion" section presents the experimental results on 23 benchmark functions and the CEC2017 and CEC2022 benchmark suites. "IMRFO for engineering problems" section solves the engineering problems, and "Conclusion" section concludes this work.

## MRFO algorithm

The manta ray’s chain, cyclone, and somersault foraging process are related to the three stages of the manta ray foraging algorithm.

### Chain foraging

**Figure 2:**
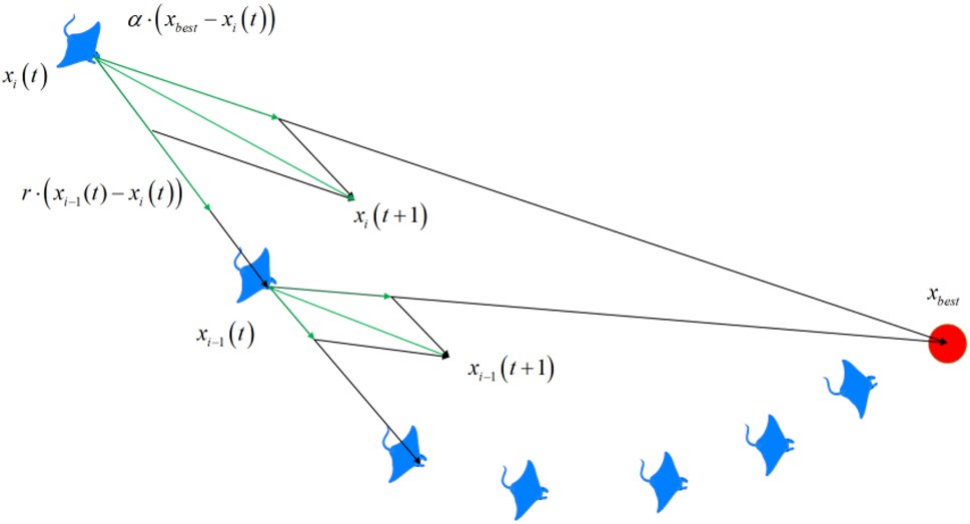
Chain foraging behavior sectional drawing.

During the manta rays’ chain foraging, they line up to form an orderly chain, and the mathematical model is:

1 $${\varvec{x}}_{i}^{d} \left( {t + 1} \right) = \left\{ \begin{gathered} {\varvec{x}}_{i}^{d} \left( t \right) + r \cdot \left( {{\varvec{x}}_{best}^{d} \left( t \right) - {\varvec{x}}_{i}^{d} \left( t \right)} \right) + \alpha \cdot \left( {{\varvec{x}}_{best}^{d} \left( t \right) - {\varvec{x}}_{i}^{d} \left( t \right)} \right),i = 1 \hfill \\ {\varvec{x}}_{i}^{d} \left( t \right) + r \cdot \left( {{\varvec{x}}_{i - 1}^{d} \left( t \right) - {\varvec{x}}_{i}^{d} \left( t \right)} \right) + \alpha \cdot \left( {{\varvec{x}}_{best}^{d} \left( t \right) - {\varvec{x}}_{i}^{d} \left( t \right)} \right),i = 2,3, \ldots ,N \hfill \\ \end{gathered} \right.$$

2 $$\alpha = 2 \cdot r \cdot \sqrt {\left| {\log \left( r \right)} \right|}$$

where $r \in \left( {0,1} \right)$ is a random number, ${\varvec{x}}_{i}^{d} \left( t \right)$ is the current position of the $d - {\text{th}}$ dimension of the $i - {\text{th}}$ individual, and ${\varvec{x}}_{best}^{d} \left( t \right)$ is the best position at *t*th iteration of the current *d*th dimension, i.e., the position with the highest concentration of plankton. The update of the current manta ray individual position ${\varvec{x}}_{i}^{d} \left( t \right)$ is determined by the current optimal individual position ${\varvec{x}}_{best}^{d} \left( t \right)$ and the previous individual position ${\varvec{x}}_{i - 1}^{d} \left( t \right)$, $\alpha$ is the weight coefficient, and $N$ is the population size. Figure 2 illustrates the chain foraging behavior sectional drawing.

### Cyclone foraging

**Figure 3:**
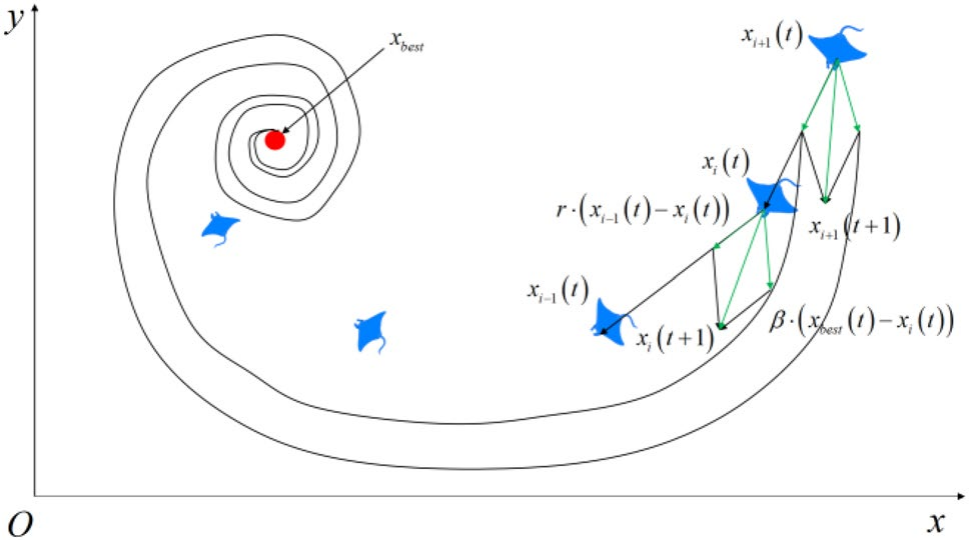
Cyclone foraging behavior sectional drawing.

In the cyclone foraging phase, the plankton concentration is high, and individual manta rays follow the previous individual and move along the cyclone path toward food. This is mathematically modeled as follows:

3 $${\varvec{x}}_{i}^{d} \left( {t + 1} \right) = \left\{ \begin{gathered} {\varvec{x}}_{best}^{d} \left( t \right) + r \cdot \left( {{\varvec{x}}_{best}^{d} \left( t \right) - {\varvec{x}}_{i}^{d} \left( t \right)} \right) + \beta \cdot \left( {{\varvec{x}}_{best}^{d} \left( t \right) - {\varvec{x}}_{i}^{d} \left( t \right)} \right),i = 1 \hfill \\ {\varvec{x}}_{best}^{d} \left( t \right) + r \cdot \left( {{\varvec{x}}_{i - 1}^{d} \left( t \right) - {\varvec{x}}_{i}^{d} \left( t \right)} \right) + \beta \cdot \left( {{\varvec{x}}_{best}^{d} \left( t \right) - {\varvec{x}}_{i}^{d} \left( t \right)} \right),i = 2,3, \ldots ,N \hfill \\ \end{gathered} \right.$$

4 $$\beta = 2 \cdot e^{{\frac{{r_{1} \left( {T - t + 1} \right)}}{T}}} \cdot \sin \left( {2\pi r_{1} } \right)$$

where $r \in rand\left( {0,1} \right)$, $\beta$ is the inertia weight, $r_{1} \in \left[ {0,1} \right]$ is the uniformly distributed random number, $t$ and $T$ are the current and maximum number of iterations, respectively. When $\frac{t}{T} \prec r$, in order to ensure the diversity of individuals, all individuals randomly assign a new position as their reference position in the whole search space, formulated as follows:

5 $${\varvec{x}}_{rand}^{d} = {\varvec{L}}_{b}^{d} + r \cdot \left( {{\varvec{U}}_{b}^{d} - {\varvec{L}}_{b}^{d} } \right)$$

6 $${\varvec{x}}_{i}^{d} \left( {t + 1} \right) = \left\{ \begin{gathered} {\varvec{x}}_{rand}^{d} \left( t \right) + r \cdot \left( {{\varvec{x}}_{rand}^{d} \left( t \right) - {\varvec{x}}_{i}^{d} \left( t \right)} \right) + \beta \cdot \left( {{\varvec{x}}_{rand}^{d} \left( t \right) - {\varvec{x}}_{i}^{d} \left( t \right)} \right),i = 1 \hfill \\ {\varvec{x}}_{rand}^{d} \left( t \right) + r \cdot \left( {{\varvec{x}}_{i - 1}^{d} \left( t \right) - {\varvec{x}}_{i}^{d} \left( t \right)} \right) + \beta \cdot \left( {{\varvec{x}}_{rand}^{d} \left( t \right) - {\varvec{x}}_{i}^{d} \left( t \right)} \right),i = 2,3, \cdots ,N \hfill \\ \end{gathered} \right.$$

where ${\varvec{x}}_{rand}^{d} \left( t \right)$ is the random location of random production, ${\varvec{L}}_{b}^{d}$ and ${\varvec{U}}_{b}^{d}$ are the lower and upper bounds of the search space, respectively. A sectional drawing of the cyclone foraging behavior is presented in Fig. 3.

### Somersault foraging

**Figure 4:**
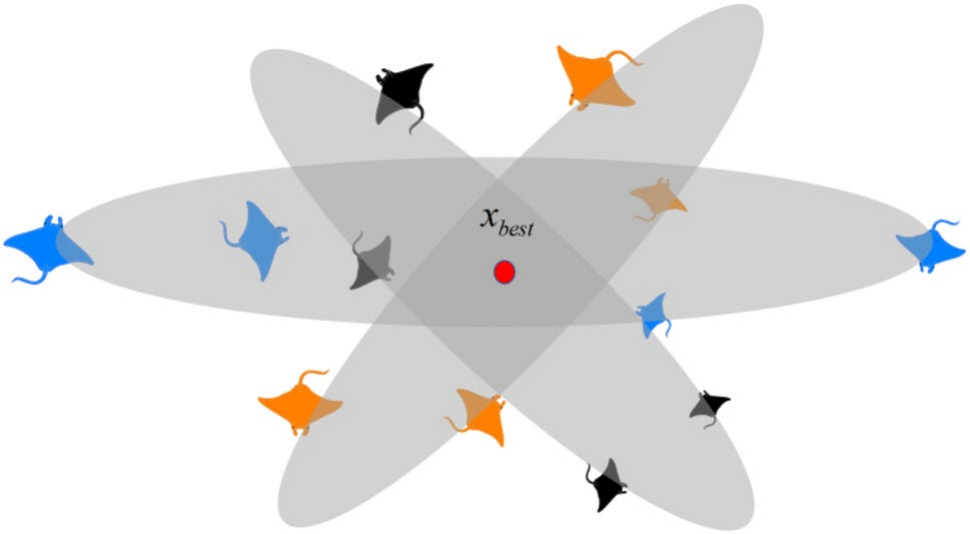
Somersault foraging behavior sectional drawing.


When the manta rays find the densest spot of food, they start to forage, forming a somersault, and the mathematical model is as follows:

7 $${\varvec{x}}_{i}^{d} \left( {t + 1} \right) = {\varvec{x}}_{i}^{d} \left( t \right) + s \cdot \left( {r_{2} \cdot {\varvec{x}}_{best}^{d} - r_{3} \cdot {\varvec{x}}_{i}^{d} \left( t \right)} \right),i = 1,2, \ldots ,N$$

where $s$ is the somersault factor, representing the manta ray somersault intensity. Generally, $s = 2$^35^, and $r_{2} ,r_{3} \in \left( {0,1} \right)$ is a random number. Figure 4 depicts the somersault foraging behavior sectional drawing. The MRFO pseudo code of the model above is presented in MRFO pseudo code.

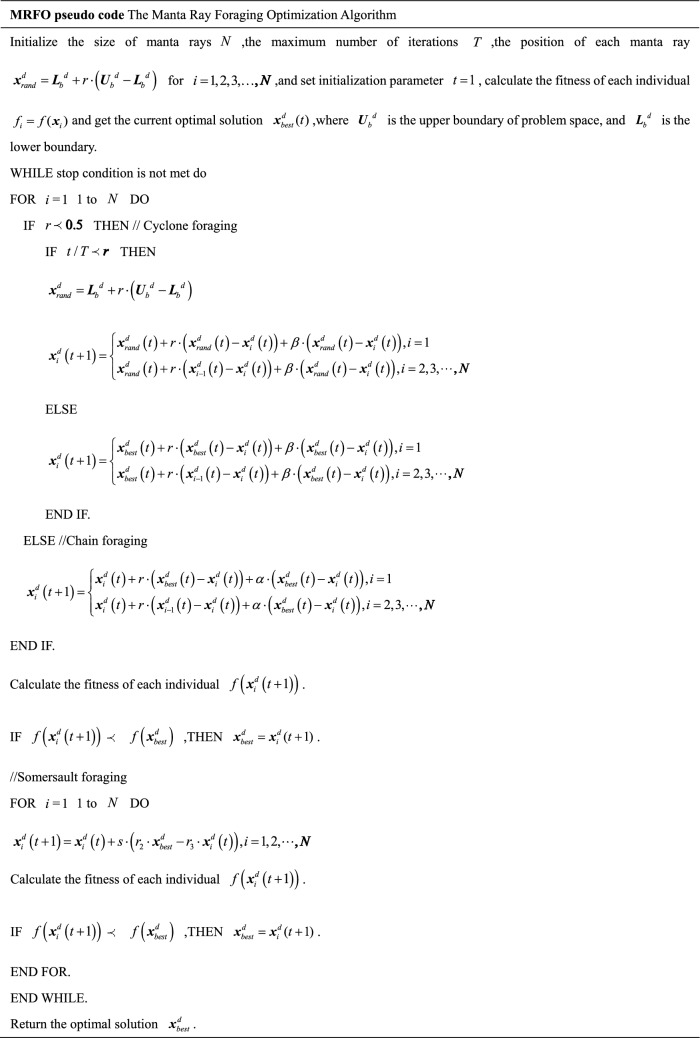


## Improved strategy for MRFO

### Tent mapping

For strong randomness and ergodicity, chaos theory has been widely applied in the optimization process of various algorithms^36^, as it can increase the search space compared with random theory. Tent Chaos mapping^37^ is a chaotic mathematical model with uniform ergodicity, making the population more uniform and improving the initial solution's quality. It is mathematically expressed as follows:

8 $$\left\{ \begin{gathered} x_{k + 1} = x_{k} /\mu ,0 < x_{k} < \mu \hfill \\ x_{k + 1} = (1 - x_{k} )/(1 - \mu ),\mu \le x_{k} \le 1 \hfill \\ \end{gathered} \right.$$

9 $$x_{i,j} = x_{\min ,j} + x_{k,j} \times (x_{\max ,j} - x_{\min ,j} )$$

**Figure 5:**
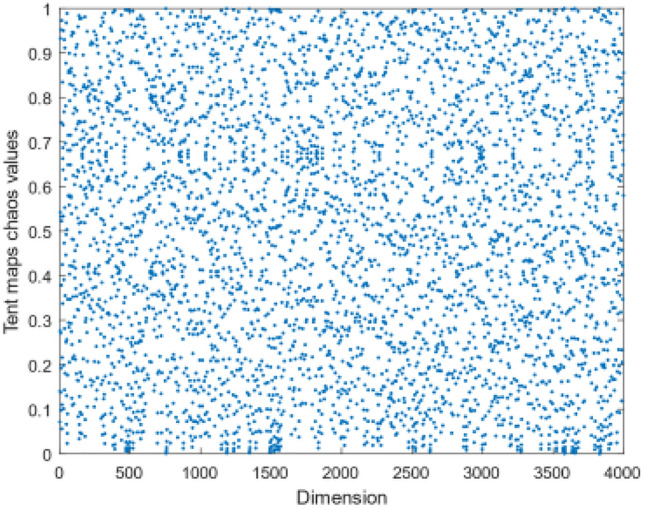
Distribution of Tent Mapping when $\mu = 0.509$.

The Tent map is in a chaotic state in the range of $(0,1)$, but for $\mu = 0.5$ it is a periodic distribution. To ensure the randomness and ergodicity of the Tent map, $\mu \ne 0.5$ is taken. Figure 5 presents the distribution of Tent Mapping for $\mu = 0.509$. In this paper, the steps of initialization of manta rays by Tent chaotic mapping are as follows:

- *Step 1* Set the manta ray population of $N$, dimension $D$, and maximum iterations of $k$, randomly generate the initial population value $x(i,j)$, generate $\mu (j) \in rand\left( {0,1} \right)$, and $\mu \ne 0.5$. The initial value of $i,j,k$ is 1.
- *Step 2* Iterate according to formula (9), $j \to j + 1$, $k \to k + 1$, and generate the $x_{k,j}$ sequence. The initial population $x_{i,j}$ sequence is generated by iterating $i \to i + 1$ according to Eq. (8).
- *Step 3* Determine the maximum number of iterations. If $k$ is reached, output the $x$ sequence. Otherwise, return to Step 2 and continue the iteration.

### The bidirectional search strategy

**Figure 6:**
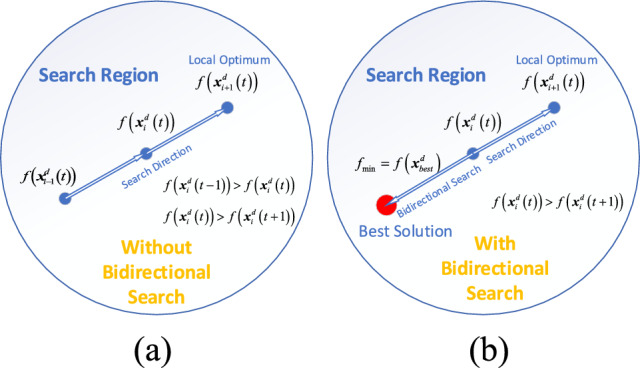
The bidirectional search strategy.

We employ the bidirectional search strategy^38^ to enlarge the search scope and prevent the algorithm from searching along a fixed direction. This strategy is presented in Fig. 6 and is formulated as follows:

10 $${\varvec{x}}_{i + 1}^{d} = {\varvec{x}}_{i}^{d} + rand*\left( {{\varvec{x}}_{best}^{d} - {\varvec{x}}_{i}^{d} } \right) - rand*\left( {{\varvec{x}}_{worst}^{d} - {\varvec{x}}_{i}^{d} } \right)$$

11 $$\left\{ \begin{gathered} f\left( {{\varvec{x}}_{i}^{d} \left( {t + 1} \right)} \right) < f\left( {{\varvec{x}}_{i}^{d} \left( t \right)} \right),{\varvec{x}}_{i}^{d} \left( {t + 1} \right)\user2{ = x}_{i}^{d} \left( {t + 1} \right) \hfill \\ f\left( {{\varvec{x}}_{i}^{d} \left( {t + 1} \right)} \right) \ge f\left( {{\varvec{x}}_{i}^{d} \left( t \right)} \right),{\varvec{x}}_{i}^{d} \left( {t + 1} \right)\user2{ = x}_{i}^{d} \left( t \right) \hfill \\ \end{gathered} \right.$$

where ${\varvec{x}}_{best}^{d}$ and ${\varvec{x}}_{worst}^{d}$ are the current optimal solution and the worst solution respectively. In the IMRFO algorithm, after the end of the chain foraging stage, the fitness value of the $t - {\text{th}}$ iteration is $f\left( {{\varvec{x}}_{i}^{d} \left( t \right)} \right)$, the fitness value of the $\left( {t + 1} \right)$th iteration is $f\left( {{\varvec{x}}_{i}^{d} \left( {t + 1} \right)} \right)$, and the optimal solution is $f_{\min } = f({\varvec{x}}_{best}^{d} )$. If a bidirectional search strategy is not employed when $f\left( {{\varvec{x}}_{i}^{d} \left( t \right)} \right) > f\left( {{\varvec{x}}_{i}^{d} \left( {t + 1} \right)} \right)$, the search will be conducted along the Search Direction (the direction of the arrows in Fig. 6a). In this case, the algorithm will find the local optimal solution in the $\left( {t + 1} \right)$th iteration while adding the bidirectional search strategy. Additionally, the algorithm will search along the bidirectional directions, jump out the local optimal solution, and find the optimal solution $f({\varvec{x}}_{best}^{d} )$ in Fig. 6b. Thus, the bidirectional search strategy could expand the search scope effectively.

### Levy flight strategy

The Levy Flight is related to chaos theory^39^ and has a wide range of applications in the measurement and simulation of random and pseudo-random natural phenomena^40^. The Levy flight is a random walking process whose action trajectory is a combination of size and size steps, which is essentially a non-Gaussian random process. In the somersault foraging stage of the IMRFO algorithm, the Levy flight is added to renew the population, thereby improving the solution's richness, increasing the search scope, and enhancing the optimization ability.

The Levy flight is formulated as follows:

12 $${\varvec{x}}_{i}^{t + 1} = {\varvec{x}}_{i}^{t} + \alpha \oplus Levy(\lambda )$$

where ${\varvec{x}}_{i}^{t}$ is the position of the $t$th iteration, $\oplus$ indicates the point-to-point multiplication, $\alpha$ is the step control parameter, and $Levy\left( \lambda \right)$ is the random search path. The following conditions should be met:

13 $$Levy \sim u = t^{ - \lambda } ,1 < \lambda \le 3$$

**Figure 7:**
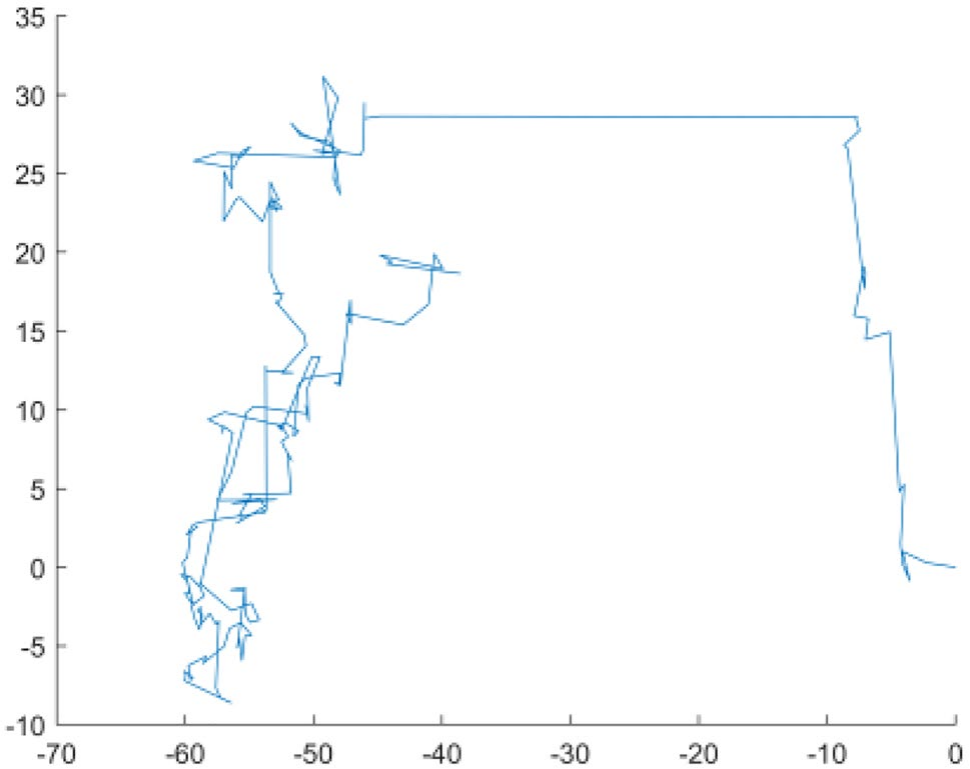
The path of Levy flight when $\beta = 1.40$.

**Figure 8:**
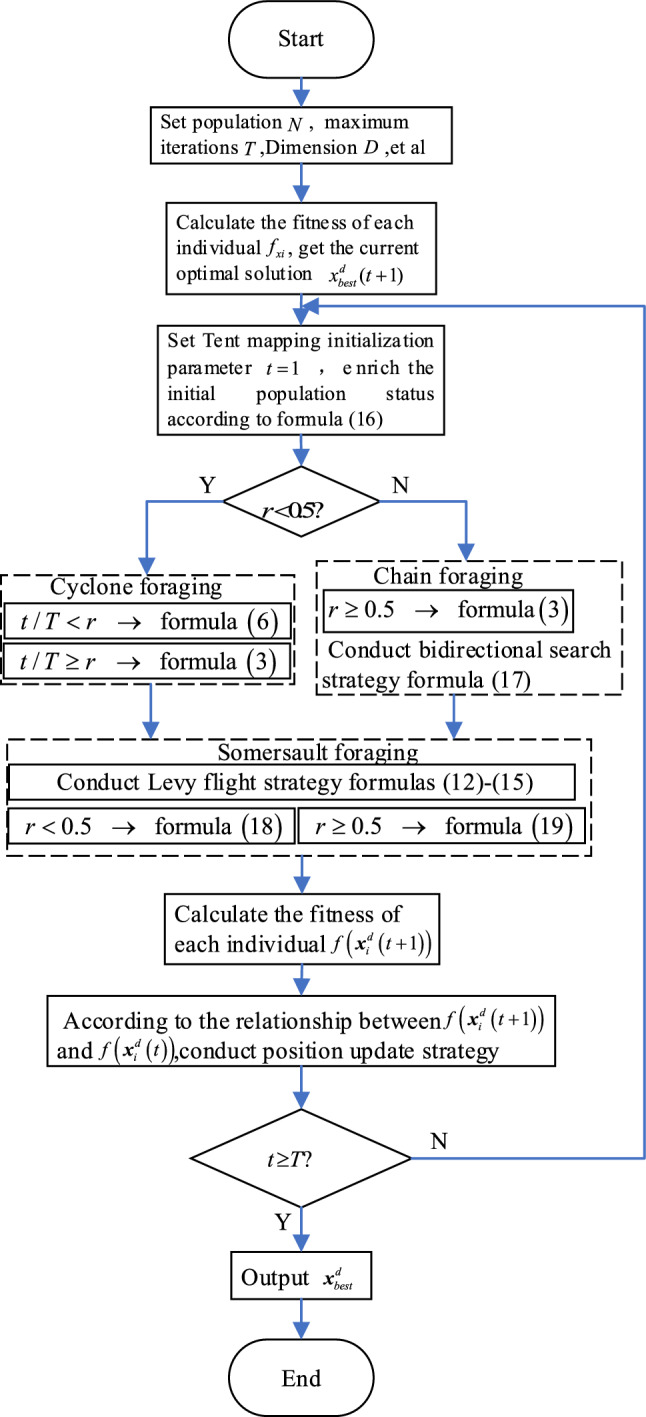
The IMRFO algorithm’s flowchart.


The random search step size of the Levy Flight is:

14 $$s = \frac{\mu }{{\left| \upsilon \right|^{{\frac{1}{\beta }}} }}$$

15 $$\left\{ \begin{gathered} \sigma_{\mu } = \left\{ {\frac{\Gamma (1 + \beta )\sin (\pi \beta /2)}{{\Gamma \left[ {(1 + \beta )/2} \right]\beta 2^{{\frac{(\beta - 1)}{2}}} }}} \right\} \hfill \\ \sigma_{\upsilon } = 1 \hfill \\ \end{gathered} \right.^{{\frac{1}{\beta }}}$$

where $$s$$ is the flight search step size, $$\beta$$$$\in$$ $$\left( {1,2} \right]$$, usually $$\beta = 1.5$$, and $$\mu ,\upsilon$$ follows the normal distribution, with $$\mu \sim N(0,\sigma_{\mu }^{2} )$$ and $$\upsilon \sim N(0,\sigma_{\upsilon }^{2} )$$. The IMRFO pseudo code of the model above is presented in IMRFO pseudo code. Figure 7 depicts the path of the Levy flight, and Fig. 8 shows the IMRFO algorithm flowchart.

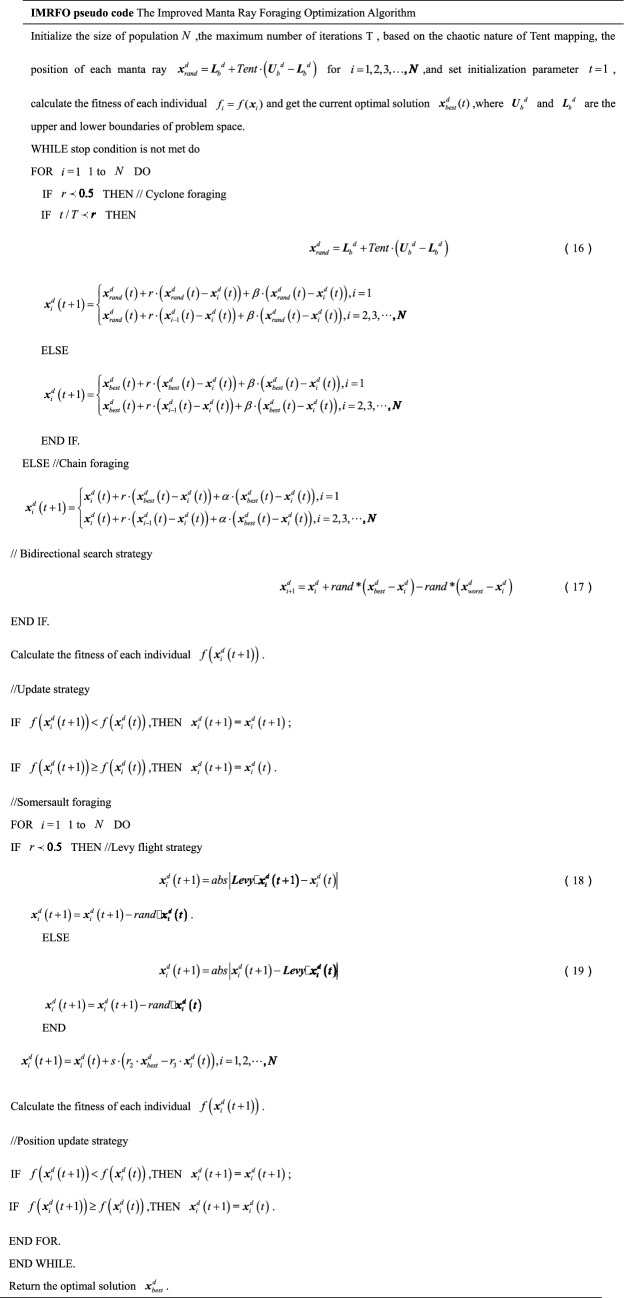


### Exploitation and exploration analysis

The operators $\alpha ,\beta ,r,s$ in the original MRFO allow the search agents to update their position based on the location of ${\varvec{x}}_{i}^{d} \left( t \right)$ and ${\varvec{x}}_{i - 1}^{d} \left( t \right)$. However, the MRFO easily falls into local solutions. Therefore, we introduced Tent chaos mapping into the algorithm’s initialization phase to make the initial solution have better ergodicity, uniformity, and randomness in the search space. Furthermore, the bidirectional search strategy helps the algorithm search bidirectionally and thus enlarges the search scope. The two strategies strengthen IMRFO’s exploitation ability when $t/T < 0.5$. In IMRFO’s somersault foraging stage, the search agents have reached the highest concentration of food, and the Levy flight strategy takes advantage of the randomness of the search step size to escape from the local optimal. Moreover, the location update strategy $abs\left| {{\varvec{Levy}} \cdot {\varvec{x}}_{i}^{d} \left( {t + 1} \right) - {\varvec{x}}_{i}^{d} \left( t \right)} \right|$ enhances IMRFO’s exploration ability.

## Experimental results and discussion

This section challenges the performance of IMRFO on three classes of widely recognized algorithms. (1) Classical algorithm, namely, PSO^14^, GWO^18^, GA^41^. (2) Advanced algorithms, such as SO^42^, SMA^21^, BWO^43^, SFO^44^, Chimp^45^. (3) New meta-heuristic algorithms, namely, AOA^46^.

To increase the experiment's credibility, we tested IMRFO and 10 other state-of-the-art algorithms under the same test environment on 23 benchmark functions, the CEC 2017 and CEC 2022 benchmark suites. Additionally, we tested three variants of IMRFO and MRFO on 23 benchmark functions simultaneously to analyze the performance of the IMRFO modifications. During the test process on the 23 benchmark functions, F1-F13 refer to Dim = 30, and F14-F23 are fixed dimensions. For the CEC 2017 and CEC2022 benchmark suites, all functions are Dim = 30, all algorithms run 30 times, and the best (Best), average (Mean), and standard deviation (Std) were recorded, respectively. The best results and standard deviation values are highlighted in bold.

The last three lines in Tables 1, 2, 3, 4 and 5 involve symbol analysis and Friedman mean analysis to obtain meaningful statistical results. When solving the tested function, the analysis of the symbols (W|T|L) represents the algorithms’ statistical number with win, tie, and loss. The Friedman mean analysis includes Friedman's mean value and ranking, which indicate the comprehensive ability of all algorithms to solve the test function.

**Table 1:** Experimental results of 11 algorithms on the benchmark functions.

ID	PSO	GA	GWO	AOA	SO	SMA	BWO	SFO	Chimp	MRFO	IMRFO
F1											
Best	8.7049E+03	1.3765E+06	8.1363E−05	5.3715E−01	2.4972E−77	0.0000E+00	1.9176E−263	3.2600E−20	1.2949E+02	0.0000E+00	0.0000E+00
Avg	1.0227E+04	1.4795E+06	1.6380E−04	6.0554E−01	3.4937E−72	3.5314E−140	7.0797E−252	1.7809E−17	2.8805E+02	**0.0000E**+**00**	**0.0000E**+**00**
Std	6.1498E+02	3.9015E+04	4.1954E−05	4.3056E−02	8.2280E−72	1.8350E−139	**0.0000E**+**00**	3.2364E−17	1.1834E+02	**0.0000E**+**00**	**0.0000E**+**00**
F2											
Best	1.5344E+00	3.2126E+01	1.1870E−20	0.0000E+00	7.7197E−48	1.5048E−267	3.3663E−137	3.8005E−11	6.4301E−09	2.3815E−225	5.9595E−238
Avg	3.3777E+00	5.3279E+01	5.0615E−20	**0.0000E**+**00**	1.1184E−45	8.6105E−169	1.2694E−132	2.7681E−09	2.1010E−06	8.0064E−217	1.7975E−224
Std	9.6997E−01	9.0760E+00	3.3814E−20	**0.0000E**+**00**	1.8432E−45	**0.0000E**+**00**	5.4010E−132	2.4056E−09	5.9867E−06	**0.0000E**+**00**	**0.0000E**+**00**
F3											
Best	5.7162E+01	2.2475E+04	9.0331E−12	6.3570E−201	1.8391E−67	0.0000E+00	8.6086E−259	3.7101E−20	2.1770E−02	0.0000E+00	0.0000E+00
Avg	1.2932E+02	3.9107E+04	1.8563E−08	5.4302E−03	6.4054E−60	8.1642E−261	1.8031E−247	7.8365E−16	1.8238E+01	**0.0000E**+**00**	**0.0000E**+**00**
Std	3.7138E+01	9.1814E+03	4.8603E−08	1.1053E−02	2.3860E−59	**0.0000E**+**00**	**0.0000E**+**00**	2.3612E−15	2.9587E+01	**0.0000E**+**00**	**0.0000E**+**00**
F4											
Best	1.2048E+00	4.8787E+01	2.4659E−09	4.9633E−97	1.5175E−44	1.0201E−220	5.0348E−132	4.9297E−13	4.3830E−04	9.9074E−222	9.0621E−230
Avg	1.7480E+00	6.1008E+01	2.1747E−08	2.8559E−02	2.9573E−42	4.8851E−162	2.1702E−127	4.2199E−10	6.5825E−02	1.4187E−211	**2.0578E−217**
Std	2.5021E−01	6.7361E+00	1.7235E−08	2.1431E−02	4.8047E−42	**0.0000E+00**	6.9950E−127	5.1911E−10	6.6314E−02	**0.0000E+00**	**0.0000E+00**
F5											
Best	2.5554E+02	7.3735E+05	2.6105E+01	2.7044E+01	9.3200E−02	2.0361E−01	7.7013E−10	2.8704E+01	2.8706E+01	2.0542E+01	5.1502E−08
Avg	6.5663E+02	7.4070E+06	2.6807E+01	2.8267E+01	2.2798E+01	3.6552E+00	**2.0256E−07**	2.8707E+01	2.8905E+01	2.1569E+01	4.1318E−01
Std	3.0337E+02	5.7561E+06	6.4795E−01	3.5859E−01	2.2491E+01	8.0838E+00	**4.6419E−07**	7.6413E−04	9.1571E−02	4.3284E−01	7.0716E−01
F6											
Best	4.5775E−01	4.9685E+03	3.6347E−05	2.5842E+00	8.4783E−03	4.6723E−07	2.9517E−16	1.3352E−02	2.3105E+00	8.4731E−15	5.2916E−18
Avg	1.9738E+00	1.2324E+04	5.1018E−01	2.9171E+00	1.7027E−01	3.1918E−03	8.5895E−15	2.0111E+00	2.9593E+00	1.0177E−13	**3.0960E−15**
Std	1.0502E+00	5.4620E+03	3.2540E−01	1.9502E−01	1.4965E−01	1.6681E−03	9.2603E−15	2.2178E+00	3.0583E−01	1.2682E−13	**5.6606E−15**
F7											
Best	1.2322E+00	5.6363E−01	5.1170E−04	5.0882E−06	2.0579E−05	1.9641E−05	1.8009E−05	1.4892E−05	3.6291E−05	2.4558E−05	6.1779E−07
Avg	1.2981E+01	4.5192E+00	1.2054E−03	5.3454E−05	1.5064E−04	1.0001E−04	8.0140E−05	1.1364E−04	1.1091E−03	1.1982E−04	**2.1519E−05**
Std	9.0999E+00	3.3524E+00	5.3692E−04	3.8327E−05	8.6658E−05	7.9440E−05	4.0619E−05	1.0835E−04	1.1634E−03	9.2437E−05	**1.5926E−05**
F8											
Best	− 8.7970E+03	− 3.2575E+03	− 7.4132E+03	− 6.2767E+03	− 1.2569E+04	− 1.2569E+04	− 1.2569E+04	− 5.8023E+03	− 5.9397E+03	− 9.9045E+03	− 1.2569E+04
Avg	− 6.7124E+03	− 2.4136E+03	− 5.8984E+03	− 5.5026E+03	− 1.2490E+04	− **1.2569E+04**	− **1.2569E+04**	− 4.1693E+03	− 5.7440E+03	− 8.4923E+03	− 1.2295E+04
Std	1.1718E+03	3.5971E+02	8.5023E+02	3.8813E+02	1.6373E+02	1.6865E−01	**5.4570E−12**	6.8259E+02	7.3622E+01	7.3566E+02	3.5825E+02
F9											
Best	6.7929E+01	1.1926E+02	0.0000E+00	0.0000E+00	0.0000E+00	0.0000E+00	0.0000E+00	0.0000E+00	5.8648E−04	0.0000E+00	0.0000E+00
Avg	1.6000E+02	1.8169E+02	1.2742E+00	**0.0000E+00**	6.2336E+00	**0.0000E+00**	**0.0000E+00**	**0.0000E+00**	1.3297E+01	**0.0000E+00**	**0.0000E+00**
Std	3.7884E+01	3.1549E+01	2.1370E+00	**0.0000E+00**	1.0900E+01	**0.0000E+00**	**0.0000E+00**	**0.0000E+00**	1.0257E+01	**0.0000E+00**	**0.0000E+00**
F10											
Best	1.6377E+00	1.5689E+01	3.6415E−14	8.8818E−16	8.8818E−16	8.8818E−16	8.8818E−16	5.1760E−11	1.9958E+01	8.8818E−16	8.8818E−16
Avg	2.4227E+00	1.9149E+01	4.2810E−14	**8.8818E−16**	4.3225E−15	**8.8818E−16**	**8.8818E−16**	4.5661E−10	1.9962E+01	**8.8818E−16**	**8.8818E−16**
Std	5.0978E−01	9.1921E−01	3.7148E−15	**1.9722E−31**	6.3773E−16	**1.9722E−31**	**1.9722E−31**	4.0286E−10	1.5059E−03	**1.9722E−31**	**1.9722E−31**
F11											
Best	5.9557E−02	3.7455E+01	0.0000E+00	8.4917E−04	0.0000E+00	0.0000E+00	0.0000E+00	0.0000E+00	7.3386E−14	0.0000E+00	0.0000E+00
Avg	9.7051E−02	1.1138E+02	5.0375E−03	1.4481E−01	2.4715E−02	**0.0000E+00**	**0.0000E+00**	**0.0000E+00**	9.7930E−03	**0.0000E+00**	**0.0000E+00**
Std	3.1246E−02	4.2659E+01	8.4367E−03	1.0107E−01	3.7288E−02	**0.0000E+00**	**0.0000E+00**	**0.0000E+00**	1.7140E−02	**0.0000E+00**	**0.0000E+00**
F12											
Best	5.2268E−03	3.4144E+01	6.7630E−03	3.4457E−01	6.2991E−04	8.5299E−05	1.5847E−16	2.3110E−03	1.5213E−01	2.3740E−16	3.6995E−20
Avg	2.2500E−02	2.0389E+06	3.0791E−02	4.4691E−01	5.8968E−02	1.3803E−03	4.0371E−14	2.6909E−01	4.0034E−01	3.4556E−03	**8.5711E−17**
Std	2.3562E−02	3.7442E+06	1.7539E−02	4.6133E−02	1.4352E−01	1.3471E−03	7.7256E−14	3.6314E−01	2.2000E−01	1.8609E−02	**1.6720E−16**
F13											
Best	1.3528E−01	1.3696E+06	7.9362E−05	2.6089E+00	1.4842E−03	3.9296E−05	1.7555E−15	9.6099E−06	2.5483E+00	5.1012E−14	2.2688E−20
Avg	4.2095E−01	1.8757E+07	3.9915E−01	2.8222E+00	3.6937E−01	2.5975E−03	9.3380E−14	3.2929E−03	2.7844E+00	1.9774E+00	**2.3768E−15**
Std	2.0031E−01	1.7891E+07	1.6763E−01	9.5403E−02	5.3018E−01	5.3698E−03	1.8256E−13	1.7613E−02	1.1510E−01	1.3199E+00	**5.1209E−15**
F14											
Best	9.9800E−01	9.9800E−01	9.9800E−01	9.9800E−01	9.9800E−01	9.9800E−01	9.9800E−01	9.9800E−01	9.9800E−01	9.9800E−01	9.9800E−01
Avg	1.7890E+00	1.1351E+00	3.8084E+00	8.7754E+00	1.0348E+00	**9.9800E−01**	**9.9800E−01**	6.4068E+00	9.9802E−01	**9.9800E−01**	**9.9800E−01**
Std	1.4917E+00	3.3183E−01	3.7478E+00	4.0515E+00	1.7883E−01	**4.4409E−16**	**4.4409E−16**	4.5689E+00	2.5096E−05	**4.4409E−16**	**4.4409E−16**
F15											
Best	3.0823E−04	7.5343E−04	3.0749E−04	3.2394E−04	3.0754E−04	3.0847E−04	3.0782E−04	3.0969E−04	1.2283E−03	3.0749E−04	3.0749E−04
Avg	8.6898E−04	1.0093E−02	2.3498E−03	6.2266E−03	4.2884E−04	5.0490E−04	3.2978E−04	8.9440E−03	1.2910E−03	3.5037E−04	**3.0749E−04**
Std	1.6445E−04	1.4917E−02	6.0067E−03	1.1653E−02	1.9179E−04	2.7825E−04	2.4087E−05	1.0192E−02	5.5654E−05	2.3095E−04	**1.0842E−19**
F16											
Best	− 1.0316E+00	− 1.0316E+00	− 1.0316E+00	− 1.0316E+00	− 1.0316E+00	− 1.0316E+00	− 1.0316E+00	− 1.0316E+00	− 1.0316E+00	− 1.0316E+00	− 1.0316E+00
Avg	− **1.0316E+00**	− 9.4243E−01	− **1.0316E+00**	− **1.0316E+00**	− **1.0316E+00**	− **1.0316E+00**	− 1.0315E+00	**− 1.0316E+00**	**− 1.0316E+00**	**− 1.0316E+00**	**− 1.0316E+00**
Std	**4.4409E−16**	1.1578E−01	**4.4409E−16**	**4.4409E−16**	**4.4409E−16**	**4.4409E−16**	1.3330E−04	**4.4409E−16**	1.2697E−05	**4.4409E−16**	**4.4409E−16**
F17											
Best	3.9789E−01	3.9789E−01	3.9789E−01	3.9807E−01	3.9789E−01	3.9789E−01	3.9791E−01	3.9789E−01	3.9789E−01	3.9789E−01	3.9789E−01
Avg	**3.9789E−01**	4.0004E−01	3.9790E−01	4.1750E−01	**3.9789E−01**	**3.9789E−01**	4.0022E−01	**3.9789E−01**	3.9833E−01	**3.9789E−01**	**3.9789E−01**
Std	**5.5511E−17**	5.1388E−03	5.9832E−05	2.3525E−02	**5.5511E−17**	2.4944E−08	2.6307E−03	5.4840E−06	6.9412E−04	**5.5511E−17**	**5.5511E−17**
F18											
Best	3.0000E+00	3.0000E+00	3.0000E+00	3.0000E+00	3.0000E+00	3.0000E+00	3.0064E+00	3.0000E+00	3.0000E+00	3.0000E+00	3.0000E+00
Avg	**3.0000E+00**	4.8046E+00	**3.0000E+00**	5.7001E+00	3.9000E+00	**3.0000E+00**	3.4644E+00	6.6000E+00	3.0001E+00	**3.0000E+00**	**3.0000E+00**
Std	**0.0000E+00**	6.7466E+00	1.4316E−05	8.1000E+00	4.8466E+00	**0.0000E+00**	5.0397E−01	1.5167E+01	7.4807E−05	**0.0000E+00**	**0.0000E+00**
F19											
Best	− 3.8628E+00	− 3.8494E+00	− 3.8628E+00	− 3.8603E+00	− 3.8628E+00	− 3.8628E+00	− 3.8626E+00	− 3.8628E+00	− 3.8617E+00	− 3.8628E+00	− 3.8628E+00
Avg	**− 3.8628E+00**	− 3.5260E+00	− 3.8614E+00	− 3.8518E+00	**− 3.8628E+00**	**− 3.8628E+00**	− 3.8594E+00	− 3.8404E+00	− 3.8554E+00	**− 3.8628E+00**	**− 3.8628E+00**
Std	**1.7764E−15**	2.4051E−01	2.6807E−03	4.0820E−03	**1.7764E−15**	**1.7764E−15**	2.4693E−03	4.7352E−02	2.2711E−03	**1.7764E−15**	**1.7764E−15**
F20											
Best	− 3.3220E+00	− 2.9413E+00	− 3.3220E+00	− 3.2073E+00	− 3.3220E+00	− 3.3220E+00	− 3.3198E+00	− 3.3078E+00	− 3.2875E+00	− 3.3220E+00	− 3.3220E+00
Avg	− 3.2467E+00	− 1.8449E+00	− 3.2576E+00	− 3.0840E+00	− 3.3180E+00	− 3.2346E+00	− 3.2980E+00	− 3.1241E+00	− 2.6835E+00	− 3.2625E+00	**− 3.3220E+00**
Std	5.7294E−02	4.7140E−01	7.1450E−02	7.9507E−02	2.1342E−02	5.2701E−02	1.5457E−02	1.2271E−01	4.3821E−01	5.9446E−02	**8.8818E−16**
F21											
Best	− 1.0153E+01	− 2.5564E+00	− 1.0153E+01	− 5.0175E+00	− 1.0153E+01	− 1.0153E+01	− 1.0153E+01	− 1.0103E+01	− 5.0273E+00	− 1.0153E+01	− 1.0153E+01
Avg	− 8.3565E+00	− 1.0669E+00	− 8.9699E+00	− 3.4204E+00	− 1.0024E+01	**− 1.0153E+01**	− 1.0147E+01	− 8.0346E+00	− 3.8878E+00	− 9.1336E+00	**− 1.0153E+01**
Std	2.8127E+00	5.8571E−01	2.1422E+00	7.9165E−01	3.3027E−01	1.6260E−04	1.1353E−02	2.4332E+00	1.8133E+00	2.0392E+00	**3.5527E−15**
F22											
Best	− 1.0403E+01	− 2.5751E+00	− 1.0403E+01	− 7.8236E+00	− 1.0403E+01	− 1.0403E+01	− 1.0403E+01	− 1.0360E+01	− 5.0774E+00	− 1.0403E+01	− 1.0403E+01
Avg	− 9.1901E+00	− 1.1637E+00	− 1.0225E+01	− 4.1044E+00	− 1.0322E+01	**− 1.0403E+01**	− 1.0399E+01	− 7.8436E+00	− 3.9189E+00	− 9.2944E+00	**− 1.0403E+01**
Std	2.4766E+00	4.6938E−01	9.5390E−01	1.5359E+00	3.1000E−01	1.1263E−04	4.4455E−03	2.8762E+00	1.8157E+00	2.2286E+00	**0.0000E+00**
F23											
Best	− 1.0536E+01	− 2.4948E+00	− 1.0536E+01	− 6.7476E+00	− 1.0536E+01	− 1.0536E+01	− 1.0536E+01	− 1.0481E+01	− 5.1221E+00	− 1.0536E+01	− 1.0536E+01
Avg	− 9.9070E+00	− 1.4982E+00	− 1.0356E+01	− 3.5807E+00	− 1.0388E+01	**− 1.0536E+01**	− 1.0531E+01	− 7.0774E+00	− 4.9374E+00	− 9.9956E+00	**− 1.0536E+01**
Std	1.9317E+00	4.1636E−01	9.6211E−01	1.4374E+00	3.1099E−01	1.6883E−04	6.2880E−03	3.2220E+00	7.4197E−01	1.6224E+00	**5.3291E−15**
(W|T|L)	(3|20|1)	(0|23|18)	(2|21|0)	(2|19|2)	(3|20|0)	(12|11|0)	(6|17|0)	(4|19|1)	(1|22|1)	(10|13|0)	(20|3|0)
Friedman mean	7.83	10.57	6.57	8.17	5.30	3.61	3.78	7.09	8.30	3.35	1.30
ranking	8	11	6	9	5	3	4	7	10	2	1

The best results and standard deviation values are highlighted in bold.

**Table 2:** Results of IMRFO compared with other enhanced algorithms.

ID	VVSPSO^47^	EWOA^48^	IGWO^49^	dAOA^50^	MCFOA^51^	CSMA^52^	SCSO^53^	DMOA^48^	WLChimp^54^	m- MRFO^55^	IMRFO
F1											
Avg	6.66E−11	2.69E−246	8.46E−04	2.23E−52	2.78E−10	1.2E−280	**0.00E+00**	1.90E−04	4.8514E−310	1.47E−270	**0.00E+00**
Std	1.71E−10	**0.00E+00**	8.18E−05	4.70E−52	4.35E−10	**0.00E+00**	**0.00E+00**	6.24E−05	**0.00E+00**	**0.00E+00**	**0.00E+00**
F2											
Avg	2.73E−01	1.84E−128	8.90E−04	1.12E−39	6.56E−05	3.4E−156	1.80E−166	8.59E−05	6.46E−158	2.93E−135	**1.80E−224**
Std	4.38E−01	3.89E−128	7.21E−05	3.50E−39	6.69E−05	1.7E−155	**0.00E+00**	1.75E−05	8.63E−158	9.31E−135	**0.00E+00**
F3											
Avg	1.74E+01	4.70E−139	8.82E−04	2.33E−08	2.34E−07	**0.00E+00**	1.71E−95	2.23E+04	4.40E−191	1.47E−263	**0.00E+00**
Std	1.20E+01	2.27E−138	1.18E−04	4.96E−08	3.86E−07	**0.00E+00**	9.35E−95	3.00E+03	**0.00E+00**	**0.00E+00**	**0.00E+00**
F4											
Avg	1.39E+00	4.83E−103	9.35E−04	2.93E−13	4.23E−06	5.1E−134	1.21E−48	4.01E+01	7.52E−148	4.51E−135	**2.06E−217**
Std	7.24E−01	1.03E−102	5.31E−05	8.22E−13	7.27E−06	2.8E−133	6.57E−48	3.42E+00	2.98E−147	1.61E−134	**0.00E+00**
F5											
Avg	2.66E+01	2.20E+01	2.19E+01	2.65E+01	**3.55E−04**	5.04E+00	2.78E+01	3.79E+02	2.66E+01	2.40E+01	4.13E−01
Std	1.36E+00	2.48E−01	4.16E−01	4.87E−01	**5.22E−04**	9.28E+00	8.47E−01	1.18E+02	3.94E−01	4.10E−01	7.07E−01
F6											
Avg	4.82E−14	1.74E−12	8.91E−04	1.81E+00	5.50E−05	4.43E−03	2.07E+00	1.84E−04	6.72E−03	3.75E−07	**3.10E−15**
Std	8.19E−14	1.14E−12	9.38E−05	1.65E−01	8.53E−05	3.06E−03	4.41E−01	5.43E−05	1.81E−03	2.53E−07	**5.66E−15**
F7											
Avg	1.36E−02	1.26E−04	8.50E−04	3.75E−01	1.06E−04	3.00E−04	1.19E−04	5.35E−02	9.02E−05	1.19E−04	**2.15E−05**
Std	5.66E−03	9.30E−05	1.48E−04	1.90E−01	9.55E−05	2.10E−04	1.36E−04	1.26E−02	5.97E−05	1.06E−04	**1.59E−05**
F8											
Avg	– 6.07E+03	− 8.59E+03	4.04E+03	− 6.17E+03	**− 1.26E+04**	**− 1.26E+04**	− 7.02E+03	− 5.55E+03	− 5.96E+03	− 7.51E+03	− 1.23E+04
Std	1.26E+02	6.75E+02	6.09E+02	3.40E+02	**4.09E−05**	3.20E−01	8.99E+02	3.47E+02	6.39E+02	6.62E+02	3.58E+02
F9											
Avg	2.59E+00	6.11E+00	8.03E−04	**0.00E+00**	7.39E−08	**0.00E+00**	**0.00E+00**	1.97E+02	**0.00E+00**	**0.00E+00**	**0.00E+00**
Std	3.01E+00	5.75E+00	1.21E−04	**0.00E+00**	1.58E−07	**0.00E+00**	**0.00E+00**	1.22E+01	**0.00E+00**	**0.00E+00**	**0.00E+00**
F10											
Avg	2.41E−06	7.43E−15	9.15E−04	5.15E−15	1.41E−05	**8.88E−16**	**8.88E−16**	5.04E−03	1.72E−15	**8.86E−16**	**8.88E−16**
Std	6.44E−06	1.31E−15	5.68E−05	1.50E−15	1.06E−05	**0.00E+00**	**0.00E+00**	8.75E−04	1.53E−15	**0.00E+00**	1.97E−31
F11											
Avg	2.29E−13	2.95E−03	4.51E−03	6.74E−08	1.20E−11	**0.00E+00**	**0.00E+00**	1.59E−02	**0.00E+00**	**0.00E+00**	**0.00E+00**
Std	2.14E−13	9.98E−03	7.77E−03	1.70E−07	2.30E−11	**0.00E+00**	**0.00E+00**	1.73E−02	**0.00E+00**	**0.00E+00**	**0.00E+00**
F12											
Avg	2.78E−14	3.26E−13	9.21E−04	3.67E−01	2.19E−07	3.94E−03	1.18E−01	8.74E+00	2.64E−04	6.79E−09	**8.57E−17**
Std	7.56E−14	1.69E−13	8.05E−05	3.78E−02	3.47E−07	6.24E−03	5.20E−02	2.11E+00	8.86E−05	4.71E−09	**1.67E−16**
F13											
Avg	6.59E−03	3.83E−03	7.31E−03	2.76E+00	1.92E−06	6.64E−03	2.35E+00	1.64E+01	2.95E+00	2.02E+00	**2.38E−15**
Std	1.39E−02	1.42E−02	2.31E−02	2.56E+00	2.71E−06	9.89E−03	4.11E−01	4.00E+00	8.63E−02	1.37E+00	**5.12E−15**
F14											
Avg	3.66E+00	**9.98E−01**	2.39E−04	1.13E+01	**9.98E−01**	**9.98E−01**	2.51E+00	**9.98E−01**	**9.98E−01**	**9.98E−01**	**9.98E−01**
Std	2.36E+00	1.21E−15	2.75E−04	3.04E+00	4.15E−12	9.26E−13	2.69E+00	2.57E−15	3.98E−16	**1.37E−16**	4.44E−16
F15											
Avg	4.13E−04	2.42E−03	1.44E−03	5.24E−04	4.35E−04	5.50E−04	4.03E−04	4.98E−04	3.12E−04	3.38E−04	**3.07E−04**
Std	3.30E−04	6.02E−03	3.46E−03	4.34E−04	1.96E−04	2.44E−04	2.34E−04	6.99E−05	4.29E−06	1.67E−04	**1.08E−19**
F16											
Avg	**− 1.03E+00**	**− 1.03E+00**	4.57E−04	**− 1.03E+00**	− 6.45E−01	**− 1.03E+00**	**− 1.03E+00**	**− 1.03E+00**	**− 1.03E+00**	**− 1.03E+00**	**− 1.03E+00**
Std	**0.00E+00**	1.36E−15	2.92E−04	1.54E−11	2.84E−01	1.51E−09	5.36E−10	1.56E−15	6.25E−16	6.52E−16	4.44E−16
F17											
Avg	**3.98E−01**	**3.98E−01**	4.80E−04	1.79E+00	4.96E−01	**3.98E−01**	**3.98E−01**	**3.98E−01**	**3.98E−01**	**3.98E−01**	**3.98E−01**
Std	**0.00E+00**	1.06E−15	2.91E−04	2.24E+00	1.42E−01	6.82E−08	2.98E−08	1.06E−15	**0.00E+00**	**0.00E+00**	5.55E−17
F18											
Avg	**3.00E+00**	**3.00E+00**	5.50E−04	4.35E+01	2.76E+01	**3.00E+00**	**3.00E+00**	**3.00E+00**	**3.00E+00**	**3.00E+00**	**3.00E+00**
Std	1.48E−16	8.73E−15	3.16E−04	4.27E+01	7.80E+00	8.43E−12	5.97E−06	1.07E−15	2.03E−15	1.57E−15	**0.00E+00**
F19											
Avg	−3.7855E+00	**− 3.86E+00**	**5.51E−04**	**− 3.86E+00**	− 3.69E+00	**− 3.86E+00**	**− 3.86E+00**	**− 3.86E+00**	**− 3.86E+00**	**− 3.86E+00**	**− 3.86E+00**
Std	2.44E−01	8.73E−15	2.95E−04	9.63E−04	1.33E−01	4.21E−07	4.05E−03	6.25E−15	2.99E−13	2.67E−15	**1.78E−15**
F20											
Avg	−3.2863 E+00	− 3.26E+00	4.43E−02	− 3.27E+00	− 2.24E+00	− 3.26E+00	− 3.21E+00	**− 3.32E+00**	**− 3.32E+00**	− 3.28E+00	**− 3.32E+00**
Std	5.74E−02	6.30E−02	5.71E−02	6.14E−02	4.54E−01	6.07E−02	1.84E−01	2.20E−15	1.80E−11	5.83E−02	**8.88E−16**
F21											
Avg	−8.6238 E+00	− 9.62E+00	6.80E−01	− 7.63E+00	**− 1.02E+01**	**− 1.02E+01**	− 5.04E+00	**− 1.02E+01**	− 9.03E+00	− 9.98E+00	**− 1.02E+01**
Std	2.46E+00	1.63E+00	1.73E+00	2.66E+00	1.23E−06	2.74E−04	1.79E+00	2.05E−14	1.78E+00	9.31E−01	**3.55E−15**
F22											
Avg	−8.536 E+00	− 1.01E+01	1.78E−01	− 9.35E+00	**− 1.04E+01**	**− 1.04E+01**	− 6.91E+00	**− 1.04E+01**	− 9.79E+00	− 1.00E+01	**− 1.04E+01**
Std	3.03E+00	1.28E+00	9.54E−01	2.22E+00	1.91E−06	2.08E−04	2.74E+00	4.54E−09	1.31E+00	1.35E+00	**0.00E+00**
F23											
Avg	−9.866 E+00	− 1.03E+01	6.14E−04	− 9.46E+00	**− 1.05E+01**	**− 1.05E+01**	− 5.45E+00	**− 1.05E+01**	− 1.01E+01	**− 1.05E+01**	**− 1.05E+01**
Std	2.12E+00	1.07E+00	2.60E−04	2.27E+00	4.56E−06	2.99E−04	2.72E+00	1.93E−07	1.04E+00	**1.51E−15**	5.33E−15
(W|T|L)	(3|20|1)	(5|18|1)	(1|22|8)	(3|19|4)	(6|17|0)	(13|10|0)	(8|15|1)	(9|14|7)	(8|15|1)	(9|14|0)	(21|2|0)
Friedman mean	4.83	3.65	6.57	5.43	3.74	2.96	4.39	5.83	3.30	2.74	1.09
ranking	8	5	11	9	6	3	7	10	4	2	1

The best results and standard deviation values are highlighted in bold.

**Table 3:** Experimental results of 11 algorithms on CEC 2017 benchmark suite.

ID	**PSO**	**GA**	**GWO**	**AOA**	**SO**	**SMA**	**BWO**	**SFO**	**Chimp**	**MRFO**	**IMRFO**
F1											
Best	1.2510E+02	1.5113E+05	3.1548E+04	2.4407E+09	1.3452E+02	1.4716E+02	5.0555E+07	1.0576E+09	3.1344E+08	1.0151E+02	1.0598E+02
Avg	1.9442E+03	7.9566E+07	2.9879E+07	6.9146E+09	1.7575E+03	7.2120E+03	1.6069E+08	3.3542E+09	1.7059E+09	1.4992E+03	**6.2460E+02**
Std	2.5508E+03	1.0250E+08	1.1020E+08	2.9439E+09	1.6953E+03	4.2535E+03	6.2297E+07	1.9081E+09	1.5439E+09	1.4927E+03	**8.8606E+02**
F2											
Best	2.0000E+02	4.1320E+03	5.7700E+02	2.5876E+09	2.0000E+02	2.0000E+02	8.4852E+04	8.0997E+06	3.9513E+04	2.0000E+02	2.0000E+02
Avg	**2.0000E+02**	2.3746E+09	7.4599E+06	3.5858E+13	5.4380E+02	**2.0000E+02**	2.6228E+06	3.6277E+10	3.6769E+08	2.0003E+02	**2.0000E+02**
Std	**0.0000E+00**	6.3636E+09	1.4545E+07	1.1056E+14	1.7932E+03	**0.0000E+00**	2.9941E+06	5.8021E+10	7.2173E+08	1.7951E−01	**0.0000E+00**
F3											
Best	3.0000E+02	2.3117E+04	3.0519E+02	6.9183E+03	3.0405E+02	3.0000E+02	1.1133E+03	2.9893E+03	1.3719E+03	3.0000E+02	3.0000E+02
Avg	3.0005E+02	5.3770E+04	2.4739E+03	1.1425E+04	4.9549E+02	3.0001E+02	2.2984E+03	1.0135E+04	3.1257E+03	**3.0000E+02**	**3.0000E+02**
Std	6.1830E−02	2.0999E+04	2.5797E+03	2.0876E+03	2.4043E+02	1.3175E−02	7.9182E+02	3.0850E+03	8.8463E+02	**0.0000E+00**	**0.0000E+00**
F4											
Best	4.0002E+02	4.0962E+02	4.0459E+02	4.6038E+02	4.0015E+02	4.0083E+02	4.0834E+02	4.5945E+02	4.6335E+02	4.0006E+02	4.0006E+02
Avg	4.0384E+02	4.9992E+02	4.1378E+02	8.2313E+02	4.0343E+02	4.2575E+02	4.1378E+02	5.8778E+02	6.2441E+02	4.0112E+02	**4.0083E+02**
Std	1.9610E+00	6.1897E+01	1.3898E+01	3.0730E+02	1.8272E+00	3.1200E+01	3.8551E+00	1.2308E+02	1.5713E+02	5.2815E−01	**5.0111E−01**
F5											
Best	5.0796E+02	5.4668E+02	5.0525E+02	5.1849E+02	5.0398E+02	5.0697E+02	5.2351E+02	5.3750E+02	5.4176E+02	5.0696E+02	5.0796E+02
Avg	5.3426E+02	5.8095E+02	5.1734E+02	5.4621E+02	**5.1211E+02**	5.1991E+02	5.3303E+02	5.7272E+02	5.5752E+02	5.2196E+02	5.1383E+02
Std	1.5904E+01	2.0941E+01	9.9472E+00	1.5187E+01	4.4658E+00	6.9465E+00	4.7195E+00	2.3928E+01	8.6521E+00	9.5844E+00	**3.2194E+00**
F6											
Best	6.0001E+02	6.3026E+02	6.0008E+02	6.2586E+02	6.0000E+02	6.0005E+02	6.0472E+02	6.2518E+02	6.1437E+02	6.0000E+02	6.0000E+02
Avg	6.0926E+02	6.5362E+02	6.0055E+02	6.3743E+02	6.0004E+02	6.0013E+02	6.0774E+02	6.4403E+02	6.2945E+02	6.0076E+02	**6.0001E+02**
Std	8.6921E+00	1.1479E+01	5.1301E−01	8.2343E+00	5.2812E−02	6.9129E−02	1.7114E+00	1.3901E+01	8.9232E+00	2.0431E+00	**2.8742E−02**
F7											
Best	7.1413E+02	7.3721E+02	7.1824E+02	7.7649E+02	7.1374E+02	7.1807E+02	7.3660E+02	7.6964E+02	7.5860E+02	7.2387E+02	7.1209E+02
Avg	7.2566E+02	7.9125E+02	7.3175E+02	8.0050E+02	7.3204E+02	7.2646E+02	7.4739E+02	8.0892E+02	7.9616E+02	7.4281E+02	**7.2329E+02**
Std	7.2075E+00	3.3177E+01	1.1500E+01	1.1657E+01	9.1303E+00	5.2954E+00	6.4256E+00	2.0796E+01	1.7462E+01	1.7206E+01	**3.9884E+00**
F8											
Best	8.0597E+02	8.2524E+02	8.0201E+02	8.1577E+02	8.0497E+02	8.0796E+02	8.1732E+02	8.2476E+02	8.2976E+02	8.0895E+02	8.0597E+02
Avg	8.2007E+02	8.6168E+02	8.1499E+02	8.3228E+02	**8.1317E+02**	8.1768E+02	8.2467E+02	8.5290E+02	8.4486E+02	8.2023E+02	8.1443E+02
Std	8.2540E+00	1.8512E+01	6.9964E+00	8.6186E+00	4.0675E+00	5.9624E+00	4.0030E+00	1.4404E+01	9.2545E+00	7.7850E+00	**2.7399E+00**
F9											
Best	9.0000E+02	9.3272E+02	9.0007E+02	1.0425E+03	9.0000E+02	9.0000E+02	9.0838E+02	9.8941E+02	9.8020E+02	9.0000E+02	9.0000E+02
Avg	9.0546E+02	1.1030E+03	9.0853E+02	1.4054E+03	9.0213E+02	9.0208E+02	9.3778E+02	1.5126E+03	1.3287E+03	9.0148E+02	**9.0012E+02**
Std	2.9376E+01	2.4494E+02	2.1948E+01	1.8870E+02	3.1985E+00	1.0906E+01	1.5058E+01	2.5352E+02	2.0937E+02	3.2640E+00	**3.1510E−01**
F10											
Best	1.2442E+03	1.4284E+03	1.2385E+03	1.5971E+03	1.0037E+03	1.1370E+03	1.5812E+03	2.2269E+03	2.5520E+03	1.1255E+03	1.0035E+03
Avg	1.8865E+03	2.1367E+03	1.7028E+03	2.1773E+03	**1.3513E+03**	1.6397E+03	1.8339E+03	2.7937E+03	2.9056E+03	1.7293E+03	1.5506E+03
Std	1.2442E+03	1.4284E+03	1.2385E+03	1.5971E+03	1.0037E+03	1.1370E+03	1.5812E+03	2.2269E+03	2.5520E+03	1.1255E+03	**1.0035E+03**
F11											
Best	1.1087E+03	1.2539E+03	1.1058E+03	1.2158E+03	1.1021E+03	1.1031E+03	1.1245E+03	1.1775E+03	1.1766E+03	1.1010E+03	1.1030E+03
Avg	1.1353E+03	4.4260E+03	1.1371E+03	2.2508E+03	1.1144E+03	1.1867E+03	1.1708E+03	1.9074E+03	1.3735E+03	**1.1107E+03**	1.1210E+03
Std	1.8987E+01	4.8546E+03	3.8240E+01	1.3818E+03	9.6549E+00	9.4651E+01	3.9058E+01	7.1790E+02	1.0036E+02	**7.4134E+00**	1.4578E+01
F12											
Best	2.3262E+03	8.6317E+04	1.7652E+04	1.1758E+04	2.7228E+03	5.7112E+03	2.7970E+05	3.9816E+05	1.7008E+06	1.7565E+03	1.7597E+03
Avg	1.3378E+04	3.6293E+06	7.9182E+05	5.8381E+07	1.4678E+04	1.1653E+05	1.9905E+06	2.1349E+07	1.1094E+07	1.6488E+04	**9.0641E+03**
Std	1.0374E+04	3.2502E+06	8.2568E+05	1.2910E+08	1.4273E+04	2.1749E+05	1.3355E+06	3.1821E+07	7.2616E+06	1.4657E+04	**4.7257E+03**
F13											
Best	1.5116E+03	2.3296E+03	2.0991E+03	3.5789E+03	1.3494E+03	1.3594E+03	4.2804E+03	2.0442E+03	4.8014E+03	1.3969E+03	1.6044E+03
Avg	1.1006E+04	1.3807E+04	1.1771E+04	1.1387E+04	5.1558E+03	1.3306E+04	3.0584E+04	1.4776E+04	2.8414E+04	2.1885E+03	**2.1551E+03**
Std	6.9913E+03	9.2991E+03	9.0529E+03	9.0225E+03	5.6093E+03	1.3346E+04	2.2059E+04	1.0004E+04	1.3288E+04	4.9171E+02	**3.1665E+02**
F14											
Best	1.4192E+03	1.5466E+03	1.4514E+03	1.5157E+03	1.4445E+03	1.4200E+03	1.4702E+03	1.5133E+03	2.5791E+03	1.4300E+03	1.4275E+03
Avg	1.9365E+03	1.1169E+04	3.5094E+03	6.4908E+03	1.5272E+03	2.7821E+03	2.1676E+03	5.8921E+03	6.0147E+03	1.4613E+03	**1.4547E+03**
Std	7.2219E+02	8.2884E+03	1.9426E+03	6.1070E+03	5.7954E+01	2.6291E+03	6.7310E+02	5.7826E+03	7.7503E+02	1.5529E+01	**1.5332E+01**
F15											
Best	1.5247E+03	1.8785E+03	1.5547E+03	4.0295E+03	1.5863E+03	1.5247E+03	1.6873E+03	1.9335E+03	4.0258E+03	1.5199E+03	1.5223E+03
Avg	3.5379E+03	1.3401E+04	5.3416E+03	1.6058E+04	1.8902E+03	4.3908E+03	3.5391E+03	2.3596E+04	1.5032E+04	1.6674E+03	**1.6073E+03**
Std	2.4194E+03	1.1035E+04	4.9382E+03	5.6787E+03	2.9338E+02	3.6203E+03	1.3269E+03	1.6456E+04	8.8064E+03	1.1025E+02	**5.9864E+01**
F16											
Best	1.6014E+03	1.6149E+03	1.6111E+03	1.7568E+03	1.6013E+03	1.6020E+03	1.6110E+03	1.6736E+03	1.6721E+03	1.6007E+03	1.6006E+03
Avg	1.8300E+03	1.9515E+03	1.7272E+03	2.0477E+03	1.6976E+03	1.6998E+03	**1.6865E+03**	2.0232E+03	1.8991E+03	1.6923E+03	1.8080E+03
Std	1.1800E+02	1.4600E+02	9.9328E+01	1.2040E+02	9.0369E+01	1.0773E+02	**6.6054E+01**	1.6589E+02	1.2919E+02	9.8313E+01	1.0814E+02
F17											
Best	1.7193E+03	1.7474E+03	1.7274E+03	1.7444E+03	1.7077E+03	1.7228E+03	1.7407E+03	1.7729E+03	1.7500E+03	1.7059E+03	1.7025E+03
Avg	1.7662E+03	1.8356E+03	1.7735E+03	1.8839E+03	1.7420E+03	1.7683E+03	1.7517E+03	1.8886E+03	1.7836E+03	1.7422E+03	**1.7310E+03**
Std	4.6296E+01	7.2547E+01	4.1820E+01	1.0312E+02	2.5796E+01	4.5068E+01	**5.7783E+00**	7.9807E+01	2.0876E+01	2.4472E+01	1.3467E+01
F18											
Best	2.1934E+03	2.3327E+03	3.4961E+03	2.2733E+03	2.0887E+03	4.3599E+03	5.8048E+03	2.5950E+03	2.3383E+04	2.0198E+03	1.9292E+03
Avg	1.0973E+04	1.6298E+04	2.7965E+04	1.7658E+04	8.6026E+03	3.0251E+04	4.6922E+04	1.8272E+04	7.9461E+04	5.5418E+03	**3.8134E+03**
Std	5.9228E+03	8.4938E+03	1.4133E+04	1.1956E+04	6.4948E+03	1.2671E+04	4.4331E+04	1.5246E+04	6.3846E+04	4.1581E+03	**1.6953E+03**
F19											
Best	1.9080E+03	2.0931E+03	1.9444E+03	1.9963E+03	1.9468E+03	1.9228E+03	1.9238E+03	2.1698E+03	2.4930E+03	1.9163E+03	1.9273E+03
Avg	5.7643E+03	1.5548E+04	9.1861E+03	5.5341E+04	2.5372E+03	1.2451E+04	5.1410E+03	2.7926E+04	2.1521E+04	2.0093E+03	**1.9990E+03**
Std	3.8204E+03	9.2201E+03	6.4372E+03	4.1133E+04	1.1335E+03	9.5010E+03	4.7732E+03	4.6595E+04	7.7252E+03	8.8793E+01	**5.3467E+01**
F20											
Best	2.0220E+03	2.0354E+03	2.0227E+03	2.0529E+03	2.0010E+03	2.0020E+03	2.0327E+03	2.0851E+03	2.0674E+03	2.0006E+03	2.0003E+03
Avg	2.0802E+03	2.1903E+03	2.0685E+03	2.1340E+03	2.0274E+03	2.0354E+03	2.0553E+03	2.2674E+03	2.2435E+03	2.0162E+03	**2.0119E+03**
Std	4.5052E+01	6.8896E+01	4.3799E+01	5.8659E+01	1.6542E+01	2.0340E+01	1.1521E+01	9.2808E+01	8.9273E+01	1.8980E+01	**1.0214E+01**
F21											
Best	2.2000E+03	2.2665E+03	2.2017E+03	2.2336E+03	2.3079E+03	2.2031E+03	2.2043E+03	2.2228E+03	2.2066E+03	2.2000E+03	2.2000E+03
Avg	2.2831E+03	2.3810E+03	2.3096E+03	2.3233E+03	2.3158E+03	2.3134E+03	2.2171E+03	2.3550E+03	2.3289E+03	2.2161E+03	**2.2007E+03**
Std	6.3080E+01	3.5854E+01	2.9716E+01	3.7478E+01	5.0807E+00	3.7402E+01	3.0657E+01	4.5263E+01	4.8825E+01	3.9270E+01	**1.1634E+00**
F22											
Best	2.2219E+03	2.2753E+03	2.3014E+03	2.4463E+03	2.3006E+03	2.2187E+03	2.2472E+03	2.2749E+03	2.3960E+03	2.2392E+03	2.3004E+03
Avg	2.3017E+03	2.4603E+03	2.3083E+03	2.9094E+03	2.3022E+03	2.3223E+03	2.3181E+03	2.6126E+03	3.5213E+03	**2.2997E+03**	2.3019E+03
Std	1.4989E+01	9.2046E+01	6.5062E+00	2.9541E+02	1.4276E+00	1.2407E+02	1.8077E+01	3.7577E+02	7.1968E+02	1.1265E+01	**1.3058E+00**
F23											
Best	2.6433E+03	2.6669E+03	2.6051E+03	2.6638E+03	2.6074E+03	2.6071E+03	2.6126E+03	2.6364E+03	2.6313E+03	2.6122E+03	2.3000E+03
Avg	2.6861E+03	2.7199E+03	2.6192E+03	2.7216E+03	2.61520E+03	2.6213E+03	2.6320E+03	2.6980E+03	2.6556E+03	2.6261E+03	**2.61517E+03**
Std	2.6513E+01	2.9870E+01	9.7841E+00	3.2126E+01	5.4409E+00	8.6167E+00	**6.5538E+00**	3.4455E+01	8.3167E+00	1.2794E+01	5.9748E+01
F24											
Best	2.5000E+03	2.7238E+03	2.5054E+03	2.6038E+03	2.7334E+03	2.5000E+03	2.5313E+03	2.6069E+03	2.7748E+03	2.5000E+03	2.5000E+03
Avg	2.7968E+03	2.8628E+03	2.7354E+03	2.8255E+03	2.7434E+03	2.7512E+03	2.6796E+03	2.7971E+03	2.7998E+03	2.6421E+03	**2.5385E+03**
Std	6.1586E+01	5.7150E+01	6.0523E+01	8.3357E+01	**5.0548E+00**	4.7867E+01	1.0971E+02	7.0875E+01	1.6037E+01	1.2493E+02	8.9968E+01
F25											
Best	2.8977E+03	2.9579E+03	2.8998E+03	3.0395E+03	2.8978E+03	2.8978E+03	2.9097E+03	2.9714E+03	2.9526E+03	2.8978E+03	2.8979E+03
Avg	2.9317E+03	3.0655E+03	2.9389E+03	3.2781E+03	**2.9299E+03**	2.9350E+03	2.9396E+03	3.0768E+03	3.0003E+03	2.9283E+03	2.9338E+03
Std	1.9847E+01	7.7023E+01	**1.4003E+01**	2.0241E+02	2.1483E+01	3.3596E+01	1.7465E+01	1.1295E+02	3.6107E+01	2.2373E+01	1.9273E+01
F26											
Best	2.6000E+03	3.2358E+03	2.6011E+03	3.3941E+03	2.8000E+03	2.9000E+03	2.7546E+03	3.0281E+03	3.0781E+03	2.9000E+03	2.6000E+03
Avg	3.0118E+03	3.9326E+03	3.0525E+03	3.9079E+03	3.1819E+03	3.0656E+03	2.9917E+03	3.7059E+03	3.7671E+03	2.9401E+03	**2.8449E+03**
Std	3.3370E+02	4.1035E+02	3.3912E+02	3.1386E+02	3.4640E+02	3.3451E+02	**5.4277E+01**	5.1745E+02	4.2714E+02	5.7514E+01	8.1886E+01
F27											
Best	3.0843E+03	3.1526E+03	3.0901E+03	3.1660E+03	3.0941E+03	3.0890E+03	3.0942E+03	3.1082E+03	3.0971E+03	3.0920E+03	3.0946E+03
Avg	3.1459E+03	3.2300E+03	3.0998E+03	3.2399E+03	3.1046E+03	**3.0917E+03**	3.0993E+03	3.1650E+03	3.1035E+03	3.1050E+03	3.1145E+03
Std	4.7183E+01	5.0084E+01	1.2927E+01	5.2085E+01	6.9598E+00	**1.6499E+00**	2.9890E+00	3.7809E+01	1.3176E+01	1.2535E+01	2.3110E+01
F28											
Best	3.1000E+03	3.4119E+03	3.1674E+03	3.4463E+03	3.1000E+03	3.1006E+03	3.1227E+03	3.2487E+03	3.2367E+03	3.1000E+03	2.8000E+03
Avg	3.1929E+03	3.7140E+03	3.3577E+03	3.7561E+03	3.3659E+03	3.3098E+03	3.2731E+03	3.5247E+03	3.2511E+03	3.2265E+03	**3.1642E+03**
Std	**7.4894E+01**	1.3874E+02	9.0618E+01	1.4067E+02	8.3596E+01	1.1694E+02	1.0775E+02	1.7932E+02	3.0475E+01	1.4181E+02	1.5910E+02
F29											
Best	3.1514E+03	3.1770E+03	3.1343E+03	3.2338E+03	3.1374E+03	3.1335E+03	3.1650E+03	3.2042E+03	3.2010E+03	3.1510E+03	3.1426E+03
Avg	3.2132E+03	3.3211E+03	3.1971E+03	3.4068E+03	**3.1713E+03**	3.2154E+03	3.2048E+03	3.4035E+03	3.3502E+03	3.2053E+03	3.1997E+03
Std	4.3124E+01	7.1151E+01	5.4604E+01	1.0413E+02	3.1279E+01	7.7830E+01	**2.1968E+01**	1.3476E+02	6.7979E+01	4.0689E+01	4.0703E+01
(W|T|L)	(1|22|0)	(0|23|9)	(0|23|0)	(0|23|12)	(4|19|0)	(2|22|0)	(1|22|1)	(0|23|4)	(0|23|3)	(4|19|0)	(20|3|0)
Friedman mean	4.69	9.17	5.24	9.76	3.41	5.00	5.59	9.55	8.66	3.00	1.79
ranking	4	9	6	11	3	5	7	10	8	2	1

The best results and standard deviation values are highlighted in bold.

**Table 4:** Experimental results of 11 algorithms on CEC 2022 benchmark suite.

ID	PSO	GA	GWO	AOA	SO	SMA	BWO	SFO	Chimp	MRFO	IMRFO
F1											
Best	3.0000E+02	5.5199E+03	4.0869E+02	5.0989E+03	3.0040E+02	3.0000E+02	2.3068E+03	3.9629E+03	7.5828E+02	3.0000E+02	3.0000E+02
Avg	3.0006E+02	2.8581E+04	2.3732E+03	1.0471E+04	4.3187E+02	3.0001E+02	5.4467E+03	1.1954E+04	3.5244E+03	**3.0000E+02**	**3.0000E+02**
Std	5.4308E-02	1.3418E+04	1.6596E+03	3.1712E+03	1.6535E+02	5.3363E-03	1.9639E+03	3.8841E+03	1.7716E+03	**0.0000E+00**	**0.0000E+00**
F2											
Best	4.0000E+02	4.3213E+02	4.0053E+02	4.9577E+02	4.0000E+02	4.0481E+02	4.0712E+02	4.8223E+02	4.3405E+02	4.0000E+02	4.0000E+02
Avg	4.0895E+02	4.9475E+02	4.2634E+02	1.2427E+03	4.0436E+02	4.1019E+02	4.2073E+02	6.3153E+02	5.7406E+02	4.0564E+02	**4.0074E+02**
Std	2.0904E+01	3.7097E+01	2.3543E+01	5.1176E+02	3.6339E+00	1.2951E+01	7.9694E+00	1.4311E+02	1.2558E+02	1.2762E+01	**1.9384E+00**
F3											
Best	6.0000E+02	6.3686E+02	6.0009E+02	6.2217E+02	6.0000E+02	6.0006E+02	6.0493E+02	6.1744E+02	6.1735E+02	6.0000E+02	6.0000E+02
Avg	6.0694E+02	6.5314E+02	6.0113E+02	6.3694E+02	**6.0007E+02**	6.0017E+02	6.0862E+02	6.4190E+02	6.3249E+02	6.0085E+02	6.0304E+02
Std	5.5306E+00	9.2872E+00	1.2931E+00	7.2870E+00	1.9130E-01	**1.8154E-01**	1.8073E+00	1.2462E+01	9.9407E+00	2.3727E+00	6.5935E+00
F4											
Best	8.0497E+02	8.2846E+02	8.0697E+02	8.0888E+02	8.0199E+02	8.1194E+02	8.1077E+02	8.2465E+02	8.2420E+02	8.0696E+02	8.0796E+02
Avg	8.2060E+02	8.5822E+02	8.1386E+02	8.3102E+02	**8.1035E+02**	8.2482E+02	8.2652E+02	8.5714E+02	8.3967E+02	8.1871E+02	8.2089E+02
Std	9.5576E+00	1.1676E+01	5.9365E+00	9.0989E+00	**3.6205E+00**	6.9370E+00	5.3310E+00	1.3592E+01	5.8425E+00	8.5180E+00	6.0575E+00
F5											
Best	9.0000E+02	9.0914E+02	9.0005E+02	1.0572E+03	9.0000E+02	9.0000E+02	9.0728E+02	9.7243E+02	9.9638E+02	9.0000E+02	9.0000E+02
Avg	9.4565E+02	9.7074E+02	9.2124E+02	1.3632E+03	9.0584E+02	**9.0118E+02**	9.4310E+02	1.3786E+03	1.3144E+03	9.0238E+02	9.2261E+02
Std	1.0661E+02	6.0671E+01	5.3755E+01	1.8022E+02	1.1669E+01	**1.2403E+00**	2.5577E+01	2.5675E+02	2.0888E+02	4.6204E+00	6.8088E+01
F6											
Best	1.8715E+03	1.9022E+03	2.3279E+03	2.0285E+03	1.8754E+03	2.2819E+03	3.0237E+03	2.1518E+03	1.2962E+05	1.8407E+03	1.8329E+03
Avg	3.8585E+03	3.4680E+03	5.8972E+03	5.5320E+04	3.2326E+03	5.3252E+03	1.2478E+05	2.9517E+05	1.5223E+06	3.0012E+03	**2.4028E+03**
Std	2.1798E+03	1.5051E+03	2.1357E+03	2.7771E+05	1.1891E+03	1.8274E+03	1.1064E+05	5.4215E+05	9.8262E+05	1.1024E+03	**6.5065E+02**
F7											
Best	2.0000E+03	2.0457E+03	2.0104E+03	2.0570E+03	2.0017E+03	2.0017E+03	2.0267E+03	2.0511E+03	2.0387E+03	2.0000E+03	2.0000E+03
Avg	2.0287E+03	2.0915E+03	2.0315E+03	2.1026E+03	2.0230E+03	2.0209E+03	2.0334E+03	2.1077E+03	2.0589E+03	2.0155E+03	**2.0129E+03**
Std	1.7155E+01	3.3868E+01	1.2119E+01	2.5574E+01	9.1264E+00	**3.7216E+00**	4.2607E+00	4.1405E+01	8.4714E+00	1.0963E+01	9.5843E+00
F8											
Best	2.2194E+03	2.2266E+03	2.2057E+03	2.2261E+03	2.2066E+03	2.2003E+03	2.2193E+03	2.2270E+03	2.2265E+03	2.2028E+03	2.2003E+03
Avg	2.2214E+03	2.2551E+03	2.2244E+03	2.2702E+03	2.2209E+03	2.2205E+03	2.2254E+03	2.2944E+03	2.3259E+03	2.2199E+03	**2.2195E+03**
Std	**1.4708E+00**	3.5091E+01	5.4605E+00	6.8717E+01	3.0907E+00	3.8547E+00	2.1125E+00	7.2502E+01	5.0103E+01	6.6350E+00	6.5479E+00
F9											
Best	2.4855E+03	2.6285E+03	2.5293E+03	2.6160E+03	2.5293E+03	2.5293E+03	2.5301E+03	2.6031E+03	2.5339E+03	2.5293E+03	2.5293E+03
Avg	**2.4855E+03**	2.7178E+03	2.5482E+03	2.7285E+03	2.5293E+03	2.5293E+03	2.5429E+03	2.7046E+03	2.5815E+03	2.5391E+03	2.5293E+03
Std	7.0632E-04	4.3662E+01	2.3180E+01	3.9434E+01	1.6680E−01	8.7496E-−04	1.0621E+01	5.1458E+01	2.0629E+01	3.6651E+01	**0.0000E+00**
F10											
Best	2.5001E+03	2.5070E+03	2.5003E+03	2.5097E+03	2.4072E+03	2.5003E+03	2.5005E+03	2.5026E+03	2.5009E+03	2.5002E+03	2.5002E+03
Avg	2.5696E+03	2.6070E+03	2.5532E+03	2.6178E+03	2.5024E+03	2.5288E+03	2.5523E+03	2.8071E+03	2.7450E+03	2.5005E+03	**2.5004E+03**
Std	6.0848E+01	1.4241E+02	5.6832E+01	1.0466E+02	4.7871E+01	5.1552E+01	6.2657E+01	4.8372E+02	4.9960E+02	5.7527E−01	**2.2018E-01**
F11											
Best	2.6000E+03	2.7900E+03	2.6014E+03	2.8191E+03	2.6000E+03	2.6000E+03	2.6725E+03	2.7626E+03	2.7883E+03	2.6000E+03	2.6000E+03
Avg	2.7184E+03	3.2257E+03	2.7132E+03	3.3792E+03	2.6600E+03	2.7422E+03	2.7245E+03	3.1620E+03	3.3354E+03	2.6517E+03	**2.6401E+03**
Std	1.6149E+02	3.2804E+02	7.7588E+01	4.2509E+02	7.3497E+01	1.7602E+02	**6.3494E+01**	3.8836E+02	2.5143E+02	1.2145E+02	8.6100E+01
F12											
Best	2.8513E+03	2.9125E+03	2.8623E+03	2.9043E+03	2.8627E+03	2.8586E+03	2.8623E+03	2.8739E+03	2.8646E+03	2.8649E+03	2.8647E+03
Avg	2.8864E+03	3.0064E+03	2.8666E+03	3.0093E+03	2.8692E+03	**2.8622E+03**	2.8653E+03	2.9257E+03	2.8701E+03	2.8704E+03	2.8682E+03
Std	5.9332E+01	4.3984E+01	6.2175E+00	6.0599E+01	4.4655E+00	1.4992E+00	**1.2479E+00**	3.9180E+01	1.1485E+01	7.0005E+00	3.6828E+00
(W|T|L)	(1|11|0)	(0|12|3)	(0|12|0)	(0|12|4)	(2|10|0)	(2|10|0)	(0|12|0)	(0|12|3)	(0|12|2)	(1|11|0)	(7|5|0)
Friedman mean	5.08	8.92	5.17	9.67	3.17	3.67	6.33	9.92	8.83	2.83	2.33
Ranking	5	9	6	10	3	4	7	11	8	2	1

The best results and standard deviation values are highlighted in bold.

**Table 5:** The comparison and results on benchmark functions of IMRFO and modifications.

ID		MRFO	IMRFO1	IMRFO2	IMRFO3	IMRFO			MRFO	IMRFO1	IMRFO2	IMRFO3	IMRFO
F1	Best	0.0000E+00	0.0000E+00	0.0000E+00	0.0000E+00	0.0000E+00	F12	Best	1.0661E−13	6.0886E−14	1.6443E−13	1.6846E−15	2.3056E−18
	Avg	**0.0000E+00**	**0.0000E+00**	**0.0000E+00**	**0.0000E+00**	**0.0000E+00**		Avg	2.4471E−12	4.0039E−12	3.5018E−12	2.5647E−13	**5.5269E−14**
	Std	**0.0000E+00**	**0.0000E+00**	**0.0000E+00**	**0.0000E+00**	**0.0000E+00**		Std	3.0317E−12	5.6537E−12	7.4263E−12	6.1177E−13	**2.0693E−13**
F2	Best	3.2475E−228	2.1400E−226	7.9077E−227	9.9300E−236	2.5294E−235	F13	Best	1.0987E−02	8.7689E−18	1.2611E−13	6.0400E−17	8.7689E−18
	Avg	2.3247E−218	2.4172E−218	2.1523E−216	2.5416E−222	**5.1279E−223**		Avg	2.5053E+00	7.4792E−01	2.1558E+00	1.0524E+00	**6.8072E−01**
	Std	**0.0000E+00**	**0.0000E+00**	**0.0000E+00**	**0.0000E+00**	**0.0000E+00**		Std	1.0319E+00	1.0415E+00	1.2644E+00	1.1222E+00	**9.5397E−01**
F3	Best	0.0000E+00	0.0000E+00	0.0000E+00	0.0000E+00	0.0000E+00	F14	Best	9.9800E−01	9.9800E−01	9.9800E−01	9.9800E−01	9.9800E−01
	Avg	**0.0000E+00**	**0.0000E+00**	**0.0000E+00**	**0.0000E+00**	**0.0000E+00**		Avg	**9.9800E−01**	**9.9800E−01**	**9.9800E−01**	**9.9800E−01**	**9.9800E−01**
	Std	**0.0000E+00**	**0.0000E+00**	**0.0000E+00**	**0.0000E+00**	**0.0000E+00**		Std	**4.4409E−16**	**4.4409E−16**	**4.4409E−16**	**4.4409E−16**	**4.4409E−16**
F4	Best	3.8333E−215	3.1013E−212	5.7332E−211	4.9522E−213	3.4214E−215	F15	Best	3.0749E−04	3.0749E−04	3.0749E−04	3.0749E−04	3.0749E−04
	Avg	5.2344E−199	1.0406E−197	8.9676E−198	2.0709E−202	**6.8027E−203**		Avg	4.4194E−04	3.2306E−04	3.8089E−04	**3.0749E−04**	**3.0749E−04**
	Std	**0.0000E+00**	**0.0000E+00**	**0.0000E+00**	**0.0000E+00**	**0.0000E+00**		Std	3.4777E−04	3.9707E−05	2.7881E−04	**1.0842E−19**	**1.0842E−19**
F5	Best	2.0490E+01	2.0200E+01	2.0808E+01	2.2800E−03	5.3239E−06	F16	Best	− 1.0316E+00	− 1.0316E+00	− 1.0316E+00	− 1.0316E+00	− 1.0316E+00
	Avg	2.1797E+01	2.1756E+01	2.1737E+01	**1.4118E+00**	2.8761E+00		Avg	**− 1.0316E+00**	**− 1.0316E+00**	**− 1.0316E+00**	**− 1.0316E+00**	**− 1.0316E+00**
	Std	7.6652E−01	8.0625E−01	**6.9434E−01**	3.6763E+00	5.5865E+00		Std	**4.4409E−16**	**4.4409E−16**	**4.4409E−16**	**4.4409E−16**	**4.4409E−16**
F6	Best	1.6382E−12	4.9320E−13	3.4210E−12	1.0102E−12	8.0158E−14	F17	Best	3.9789E−01	3.9789E−01	3.9789E−01	3.9789E−01	3.9789E−01
	Avg	1.3001E−10	6.4008E−11	1.2252E−10	1.2089E−10	**1.4301E−11**		Avg	**3.9789E−01**	**3.9789E−01**	**3.9789E−01**	**3.9789E−01**	**3.9789E−01**
	Std	2.0806E−10	1.0668E−10	2.9303E−10	3.5769E−10	**2.2153E−11**		Std	**5.5511E−17**	**5.5511E−17**	**5.5511E−17**	**5.5511E−17**	**5.5511E−17**
F7	Best	6.3815E−06	6.3593E−06	1.3596E−05	6.6100E−06	6.3593E−06	F18	Best	3.0000E+00	3.0000E+00	3.0000E+00	3.0000E+00	3.0000E+00
	Avg	1.3599E−04	9.1745E−05	9.7882E−05	1.1619E−04	**8.5304E−05**		Avg	**3.0000E+00**	**3.0000E+00**	**3.0000E+00**	**3.0000E+00**	**3.0000E+00**
	Std	1.4057E−04	7.0733E−05	6.2142E−05	8.9288E−05	**5.9415E−05**		Std	**0.0000E+00**	**0.0000E+00**	**0.0000E+00**	**0.0000E+00**	**0.0000E+00**
F8	Best	− 9.5492E+03	− 1.2400E+04	− 1.0181E+04	− 1.2600E+04	− 1.2569E+04	F19	Best	− 3.8628E+00	− 3.8628E+00	− 3.8628E+00	− 3.8628E+00	− 3.8628E+00
	Avg	− 8.4193E+03	− 9.0040E+03	− 8.3377E+03	− 9.8723E+03	**− 1.0264E+04**		Avg	**− 3.8628E+00**	**− 3.8628E+00**	**− 3.8628E+00**	**− 3.8601E+00**	**− 3.8628E+00**
	Std	**6.6862E+02**	1.0152E+03	8.1194E+02	2.1929E+03	1.7762E+03		Std	**3.2311E−06**	**3.2311E−06**	**3.2311E−06**	**4.9938E−04**	**3.2311E−06**
F9	Best	0.0000E+00	0.0000E+00	0.0000E+00	0.0000E+00	0.0000E+00	F20	Best	− 3.3220E+00	− 3.3220E+00	− 3.3220E+00	− 3.3220E+00	− 3.3220E+00
	Avg	**0.0000E+00**	**0.0000E+00**	**0.0000E+00**	**0.0000E+00**	**0.0000E+00**		Avg	− 3.2665E+00	− 3.2824E+00	− 3.2903E+00	− 3.2586E+00	**− 3.3141E+00**
	Std	**0.0000E+00**	**0.0000E+00**	**0.0000E+00**	**0.0000E+00**	**0.0000E+00**		Std	5.9314E−02	5.6047E−02	5.2576E−02	5.9314E−02	**2.9657E−02**
F10	Best	8.8818E−16	8.8818E−16	8.8818E−16	8.8818E−16	8.8818E−16	F21	Best	− 1.0153E+01	− 1.0153E+01	− 1.0153E+01	− 1.0153E+01	− 1.0153E+01
	Avg	**8.8818E−16**	**8.8818E−16**	**8.8818E−16**	**8.8818E−16**	**8.8818E−16**		Avg	− 8.6238E+00	− 8.1223E+00	− 8.4539E+00	− 7.9441E+00	**− 8.9637E+00**
	Std	**1.9722E−31**	**1.9722E−31**	**1.9722E−31**	**1.9722E−31**	**1.9722E−31**		Std	2.3362E+00	2.7286E+00	2.4032E+00	2.5262E+00	**2.1562E+00**
F11	Best	0.0000E+00	0.0000E+00	0.0000E+00	0.0000E+00	0.0000E+00	F22	Best	− 1.0403E+01	− 1.0403E+01	− 1.0403E+01	− 1.0403E+01	− 1.0403E+01
	Avg	**0.0000E+00**	**0.0000E+00**	**0.0000E+00**	**0.0000E+00**	**0.0000E+00**		Avg	**− 1.0003E+01**	− 9.8715E+00	− 9.1628E+00	− 8.8071E+00	− 1.0049E+01
	Std	**0.0000E+00**	**0.0000E+00**	**0.0000E+00**	**0.0000E+00**	**0.0000E+00**		Std	1.5062E+00	1.5946E+00	2.2481E+00	2.4338E+00	**1.3259E+00**
							F23	Best	− 1.0536E+01	− 1.0536E+01	− 1.0536E+01	− 1.0500E+01	− 1.0536E+01
								Avg	− 9.6348E+00	**− 1.0356E+01**	− 9.7719E+00	− 9.7840E+00	**− 1.0356E+01**
								Std	2.0153E+00	**9.7068E−01**	1.9588E+00	1.8254E+00	**9.7068E−01**
			MRFO		IMRFO1			IMRFO2		IMRFO3		IMRFO	
(W|T|L)			(11|12|5)		(11|12|2)			(10|13|2)		(12|11|3)		(20|3|1)	
Friedman mean			2.57		2.17			2.52		2.09		1.22	
Ranking			5		3			4		2		1	

The best results and standard deviation values are highlighted in bold.

### Comparisons and results

#### Comparison with Benchmark Functions

Table 1 highlights that the unimodal functions F1–F7 can be used to test the local search ability. The IMRFO algorithm obtained 5 of the best results in F1–F7, and the MRFO algorithm obtained 2, showing obvious advantages over MRFO and the other competitors. The results indicate that the chaotic mapping strategy in the algorithm initialization phase and bidirectional search strategy after the chain foraging phase effectively enhance the global optimization ability of IMRFO.

The F8–F23 functions have many local optimal values, making finding the global optimal more difficult. According to Table 1, IMRFO finds 14 of the best results for the F8–F23 benchmark functions, and MRFO obtains 8, while SMA obtains 7 best results among other competitors and 11 global optimal values. The results indicate a bidirectional search strategy after the chain foraging phase and that the Levy flight in the somersault foraging phase effectively enhances the ability of IMRFO to jump out of a local optimum.

**Figure 9:**
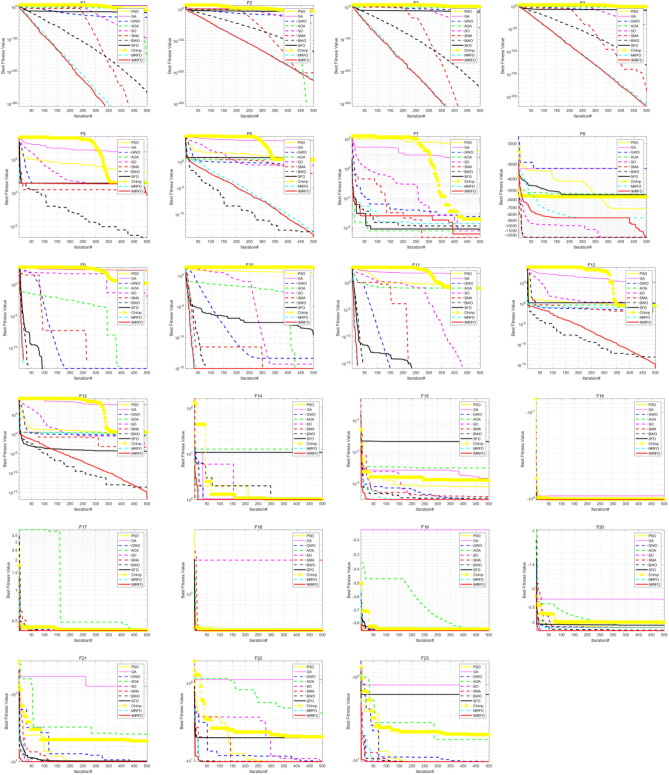
Iterative convergence curves of benchmark functions F1–F23.

The (W|T|L) result of IMRFO is (20|3|0), and IMRFO achieved the best result. The second best is the SO algorithm, and the third best is MRFO. The IMRFO’s Friedman mean value is 1.30, ranking first. The iterative convergence behaviors of all 23 functions are depicted in Fig. 9, which reveals that IMRFO performs well for most functions.

**Figure 10:**
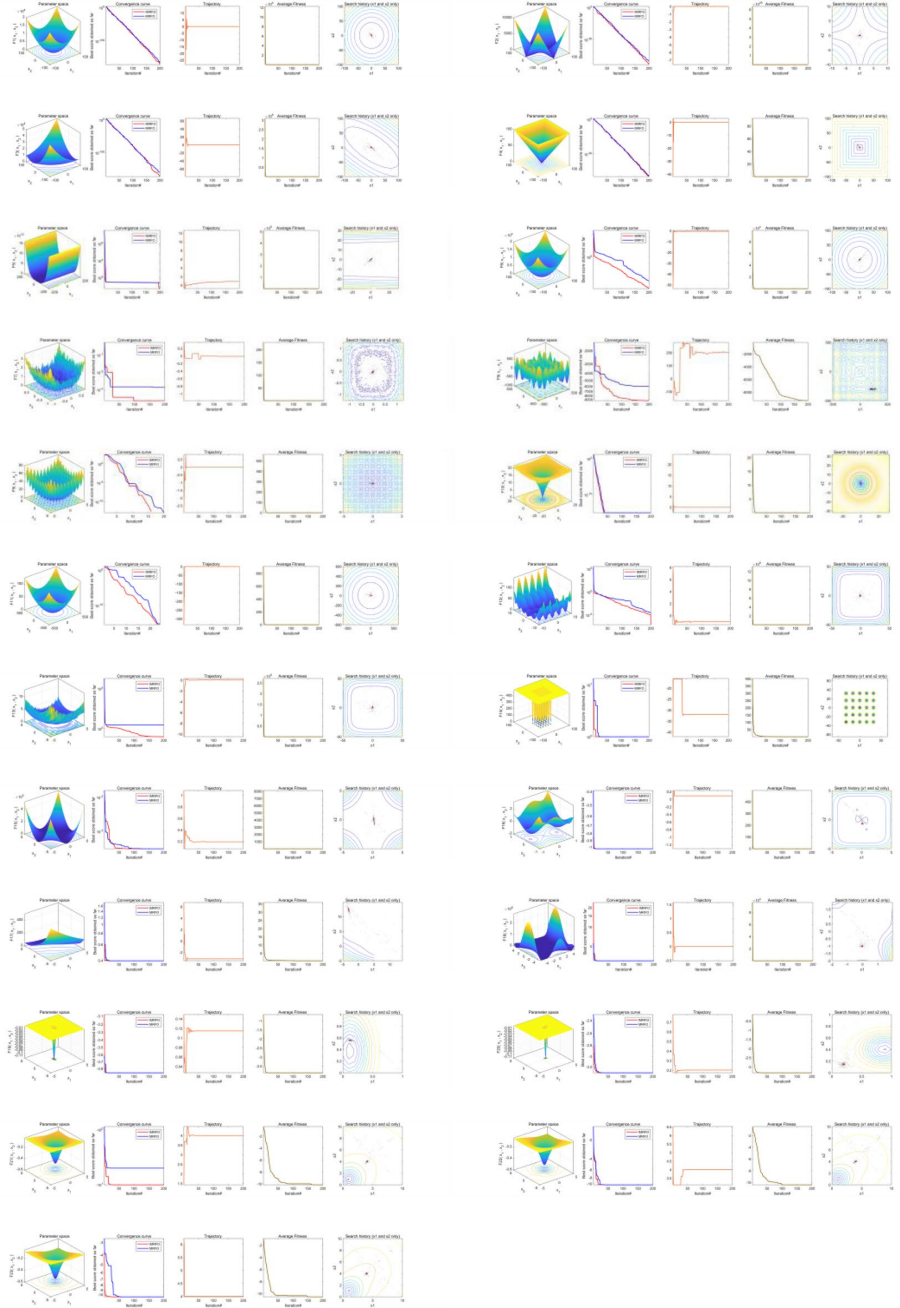
Convergence behaviors of IMRFO on benchmark functions in the search process.

Figure 10 presents the convergence behaviors of 23 benchmark functions to demonstrate the convergence of IMRFO. The first column presents a two-dimensional shape of the benchmark function. The second column presents the convergence curve, which is approximately linear or stepped. The stepped convergence curve proves that it is necessary to jump out of the local optimal to reach the global value in solving some benchmark functions, indicating that the optimal value can be reached without iteration. IMRFO demonstrates convergence. The third column presents the search agent’s trajectory in the first dimension. Specifically, the search agent starts fluctuating in the early iteration and ends once it converges and stabilizes during the iterations. This demonstrates that IMRFO performs well. The fourth column shows the changes in average fitness values throughout the iterations, which are unstable and large. The value becomes small and steady for the iteration $T = 150$, indicating the quick convergence of IMRFO. In the last column, the red point location is the best solution, and the search agents are gradually approaching it. Figure 10 demonstrates IMRFO’s good performance.

**Figure 11:**
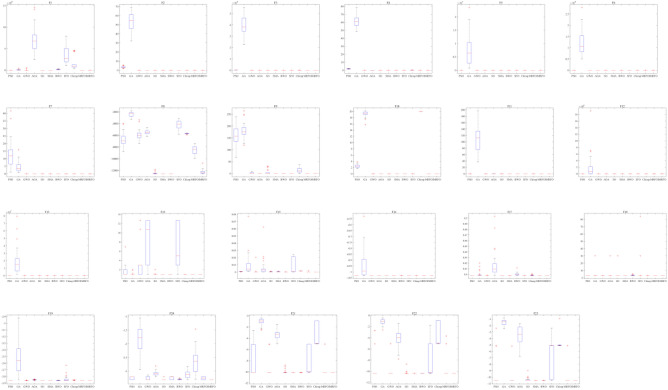
Box plot analysis for benchmark functions F1–F23.

Additionally, a box plot analysis scheme is used to analyze the distribution characteristics of the above functions by all 11 algorithms. Figure 11 reveals that IMRFO performs well in most functions compared to the competitor methods. The median, maximum, and minimum values of the objective functions obtained by IMRFO are almost the same as the optimal solutions, especially for functions F3, F9, F10, F11, F14, F15, F16, F17, F18, F19, F20, F21, F22 and F23.

#### Comparison with enhanced versions of different algorithms

For better credibility of the proposed IMRFO, we compared the proposed method with enhanced versions of different algorithms on classic benchmark functions. Table 2 infers that IMRFO has the best performance.

#### Comparison with CEC 2017 benchmark suite

**Figure 12:**
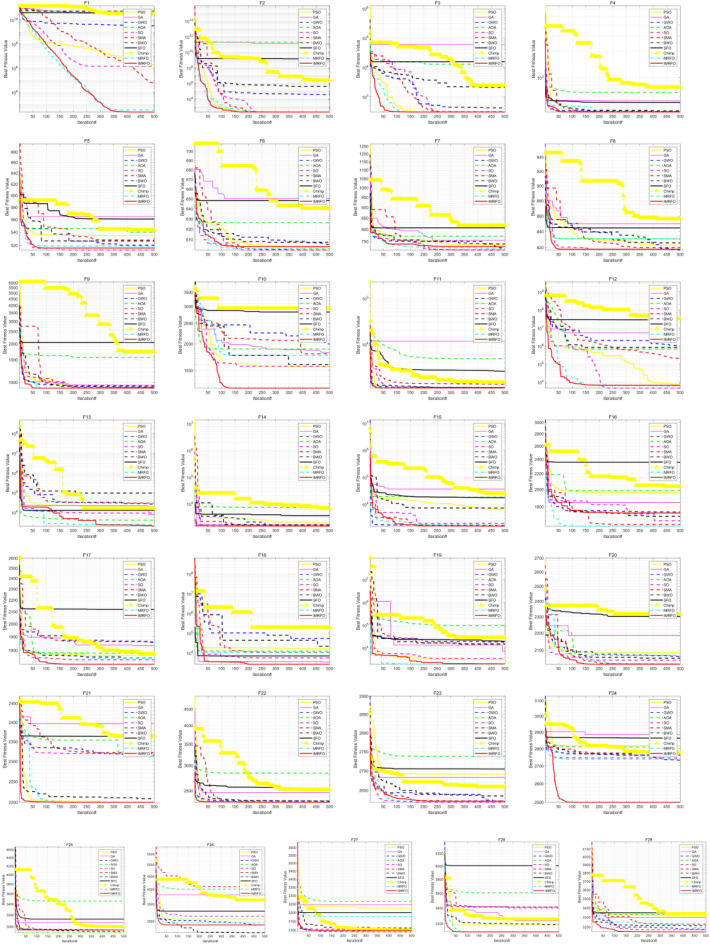
Iterative convergence curves of CEC 2017 benchmark suite of IMRFO and competitors.

Table 3 reports the results of 11 algorithms on the CEC 2017 benchmark suite, evaluating further the performance of IMRFO. Specifically, Table 2 highlights that IMRFO achieves the most wins without any losses, obtaining 15 best results and 20 global optimal values for the F1-F29 CEC 2017 benchmark suite. The Friedman mean ranking value of IMRFO is 1.79, ranking first. Figure 12 presents the iterative convergence curves of the optimization process of all 29 algorithms, revealing that IMRFO performs well for most functions. The effectiveness and superiority of our IMRFO are thus confirmed.

**Figure 13:**
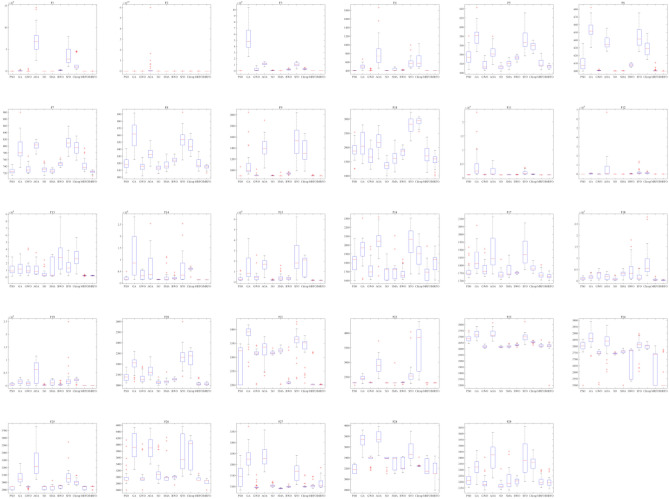
Box plot analysis for CEC 2017 benchmark suite.

Moreover, a box plot analysis is conducted to study and analyze the distribution characteristics of the CEC 2017 benchmark suite solved by IMRFO and other competitors. Figure 13 reveals that the proposed IMRFO performs well in most functions compared to competitors. The valid values of the objective functions obtained by IMRFO are almost identical to the optimal solutions, especially for functions F2, F3, F4, F11, F14 and F19.

#### Comparison with CEC 2022 benchmark suite

**Figure 14:**
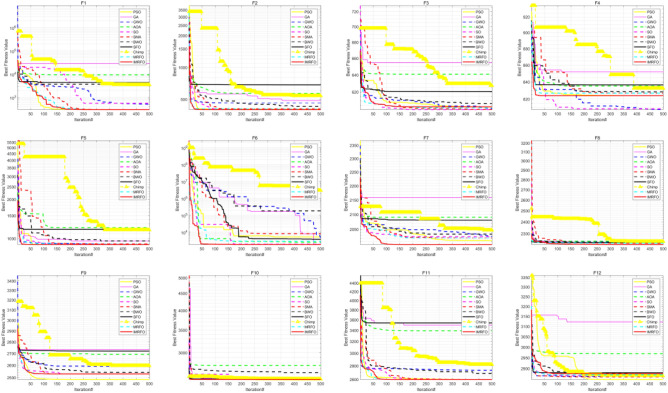
Iterative convergence curve of CEC 2022 benchmark suite of IMRFO and competitors.

The results of 11 algorithms on the CEC 2022 benchmark suite are listed in Table 4, indicating that IMRFO achieves the most wins without any losses, obtaining 7 global optimal values for the CEC 2022 benchmark suite. The Friedman mean ranking value is 2.33, ranking first. The iterative convergence curves of the optimization process of all 12 algorithms are depicted in Fig. 14, which shows that IMRFO performs well for most functions. The effectiveness and superiority of our IMRFO are thus confirmed.

**Figure 15:**
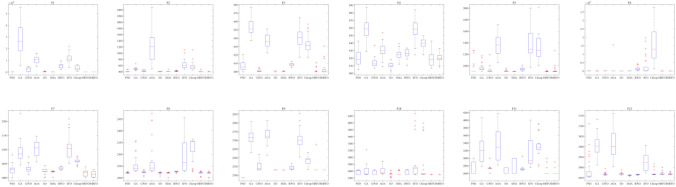
Box plot analysis for CEC 2022 benchmark suite.

Moreover, a box plot analysis is conducted, with the corresponding results illustrated in Fig. 15. The valid values of the objective functions obtained by IMRFO are almost the same as the optimal solutions, especially for functions F1 and F3.

### Impact analysis of the modifications

Next, to verify the effectiveness of the proposed strategy in the IMRFO algorithm, we compared IMRFO with MRFO and three other modifications on 23 benchmark functions. Precisely, IMRFO1 (improved algorithm using reverse search strategy and Tent chaos mapping), IMRFO2 (improved algorithm using bidirectional search strategy), IMRFO3 (improved algorithm using bidirectional search strategy and Levy flight strategy), IMRFO (improved algorithm using Tent Chaos mapping, bidirectional search strategy, and Levy flight improvements simultaneously).

According to Table 5, IMRFO finds 20 best results and 21 global optimal values for F1–F23 benchmark functions, MRFO obtains 10 best results and 11 global optimal values, among other modifications, IMRFO1 obtains 11 best results, IMRFO2 obtains 10 best results, and IMRFO3 finds 11 best results and 12 global optimal values. The results reveal that IMRFO using Tent Chaos mapping, bidirectional search strategy, and Levy flight improvements simultaneously have more advantages over MRFO and other modifications, achieving a good balance between search for global optimal and accelerated convergence.

**Figure 16:**
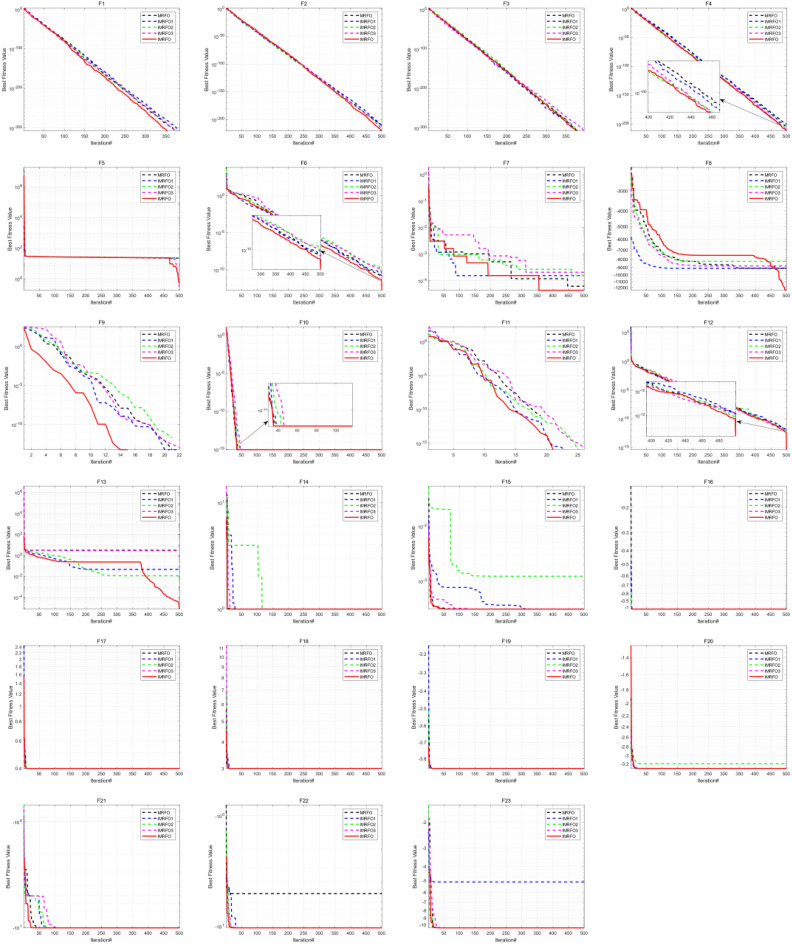
Iterative convergence curve of benchmark functions F1–F23 of IMRFO and modifications.

According to Table 5, the (W|T|L) result of IMRFO is (20|3|1), which is the best result. The Friedman mean ranking value of IMRFO is 1.22, ranking first. The value of IMRFO3 is 2.09, ranking second, the value of IMRFO1 is 2.17, ranking third, and the value of IMRFO2 and MRFO are 2.52 and 2.57, respectively, ranking fourth and last. To further compare the convergence of various algorithms in the optimization process, we draw the iterative convergence curves of the optimization process of all 23 algorithms, as depicted in Fig. 16, which suggests that IMRFO performs well for most functions among the modifications.

### Wilcoxon rank sum test

Wilcoxon’s rank sum test evaluates the difference between IMRFO and competitor methods^56^. The significance level value is set at 0.05, and Table 6 reports the significant differences between our proposed IMRFO and the other algorithms in most functions. The results are 116/14/0, 79/20/1, 280/3/7, and 117/1/2.

**Table 6:** Wilcoxon’s rank sum test statistical results.

IMRFO VS	Benchmark functions		CEC2017	CEC2022
	F1–F13	F14–F23	F1–F29	F1–F12
	Dim = 30	Fixed-dim	Dim = 30	Dim = 30
PSO	13/0/0	6/4/0	28/1/0	11/0/1
GA	13/0/0	10/0/0	29/0/0	12/0/0
GWO	13/0/0	9/1/0	26/0/3	12/0/0
AOA	11/2/0	9/1/0	29/0/0	12/0/0
SO	13/0/0	6/3/1	28/0/1	12/0/0
SMA	10/3/0	6/4/0	25/1/3	12/0/0
BWO	10/3/0	9/1/0	29/0/0	12/0/0
SFO	11/2/0	9/1/0	29/0/0	12/0/0
Chimp	13/0/0	10/0/0	29/0/0	12/0/0
MRFO	9/4/0	5/5/0	28/1/0	10/1/1
Overall	116/14/0	79/20/1	280/3/7	117/1/2

As indicated by all the results, the results of our proposed IMRFO have significant differences compared with the other 10 algorithms. Combined with the above tables and figures, our proposed IMRFO is superior to the competitor algorithms, especially for global optimization problems.

### Detailed analysis of the experimental results

Among these experimental results, the unimodal functions of benchmark functions, CEC2017 and CEC2022, can be used to test the local search ability. The IMRFO algorithm obtains the most optimal results, which indicate a chaotic mapping strategy in the algorithm initialization phase and bidirectional search strategy after the chain foraging phase, effectively enhancing the global optimization ability of IMRFO.

Other benchmark functions, CEC2017 and CEC2022, have several local optimal values, making finding the global optimal more difficult. IMRFO finds the most optimal results, which indicate a bidirectional search strategy after the chain foraging phase. Besides, the Levy flight in the somersault foraging phase effectively enhances the ability of IMRFO to jump out local optimal. Tables 1, 2, 3, 4, 5 and 6 illustrate that these three strategies can effectively improve IMRFO’s searchability.

## IMRFO for engineering problems

**Figure 17:**
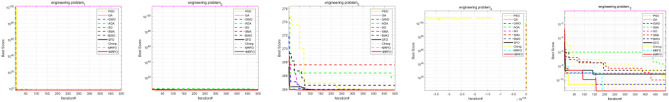
Iterative convergence curve of 5 engineering problems of IMRFO and competitors.

This section employs IMRFO to solve 5 engineering problems with 10 mentioned algorithms and other new algorithms. The 5 engineering problems aim to find the minimum objective value under certain restrictions. Each problem is solved by setting $T = 500$ and the population to $N = 50$. Table 7 presents the experimental results of 11 algorithms on engineering problems. IMRFO finds the optimal solution in all five engineering problems. Compared with other algorithms, IMRFO’s advantage is very obvious. The iterative convergence curve of IMRFO and the competitors is presented in Fig. 17.

**Table 7:** Experimental results of 11 algorithms on engineering problems.

ID	Problem 1	Problem 2	Problem 3	Problem 4	Problem 5
PSO					
Best	− 1.577790193E+02	5.973500000E+03	− 8.661914388E+05	N	**2.700857149E−12**
Avg	− 1.407730822E+01	6.239005850E+03	− 8.573769879E+05	N	2.715171837E−10
Std	3.369173242E+01	1.637231609E+02	5.050316485E+03	N	5.914319801E−10
GA					
Best	9.896153515E+100	1.679600000E+113	0.000000000E+00	2.549322238E+118	7.322578740E−01
Avg	9.945517373E+100	1.679600533E+113	Inf	2.549322238E+118	7.322578740E−01
Std	2.498100954E+98	2.872087897E+107	Inf	1.791795794E+103	3.330669074E−16
GWO					
Best	1.268537669E−02	5.894300000E+03	2.638962056E+02	1.694001433E+00	**2.700857149E−12**
Avg	1.276314281E−02	6.083072896E+03	2.639004812E+02	1.696552783E+00	2.793500390E−10
Std	8.066299708E−05	4.071524479E+02	3.753117599E−03	2.351955933E−03	4.031453341E−10
AOA					
Best	1.300218220E−02	8.262800000E+03	2.640446503E+02	1.749579186E+00	9.745653447E−10
Avg	1.324796982E−02	2.949184403E+04	2.649987263E+02	2.294724637E+00	1.102702522E−08
Std	7.664194132E−05	1.827433203E+04	8.336887591E−01	3.057771014E−01	9.878488766E−09
SO					
Best	1.266962349E−02	5.897400000E+03	2.638958434E+02	1.692775199E+00	**2.700857149E−12**
Avg	1.301239904E−02	6.225445380E+03	2.638963529E+02	1.715474537E+00	1.337003589E−10
Std	4.169371480E−04	2.245562778E+02	5.621351984E−04	9.403403306E−02	2.382489410E−10
SMA					
Best	1.271540950E−02	5.895700000E+03	2.642181311E+02	1.692803409E+00	9.939875595E−11
Avg	1.364419346E−02	6.545436516E+03	2.694822211E+02	1.694818257E+00	8.682324756E−09
Std	1.212194924E−03	5.972191403E+02	2.531450147E+00	3.500276696E−03	9.494697976E−09
BWO					
Best	1.290433339E−02	6.157400000E+03	2.639011387E+02	1.874322832E+00	**2.700857149E−12**
Avg	1.392665013E−02	8.522307913E+03	2.645354120E+02	2.137480875E+00	4.177711240E−09
Std	1.756453464E−03	1.095732384E+03	5.013816018E−01	1.927367119E−01	4.470671609E−09
SFO					
Best	1.267338076E−02	6.460200000E+03	2.638962030E+02	1.711892401E+00	**2.700857149E−12**
Avg	1.357330059E−02	3.658720187E+04	2.639721030E+02	2.353358814E+00	1.420831158E−09
Std	8.470150253E−04	3.937232581E+04	1.849232128E−01	5.402787322E−01	1.111169796E−09
Chimp					
Best	1.276091503E−02	7.145084584E+03	2.639077952E+02	1.737140646E+00	**2.700857149E−12**
Avg	1.314641822E−02	7.700362819E+03	2.640075663E+02	1.797684758E+00	9.431333745E−10
Std	3.753132062E−04	2.552801892E+02	7.723203040E−02	2.948456974E−02	7.959068647E−10
MRFO					
Best	1.266564806E−02	5.894600000E+03	2.638958469E+02	1.692768268E+00	**2.700857149E−12**
Avg	1.272938426E−02	5.935959192E+03	2.638959164E+02	1.692768402E+00	1.623612326E−10
Std	4.288311229E−05	4.813085094E+01	1.184437255E−04	3.202281848E−07	3.575332770E−10
IMRFO					
Best	**1.266548284E−02**	**5.885500000E+03**	**2.638958339E+02**	**1.692768266E+00**	**2.700857149E−12**
Avg	**1.271510560E−02**	**5.911304273E+03**	**2.638959122E+02**	**1.692768336E+00**	**8.134803865E−12**
Std	**3.707430386E−05**	**1.671781498E+01**	**8.609776904E−05**	**1.141814404E−07**	**9.011181194E−12**

Table 7 presents the experimental results of 11 algorithms on 5 engineering problems.

### Engineering problem 1: Tension/compression spring design problem (TCSD)

**Figure 18:**
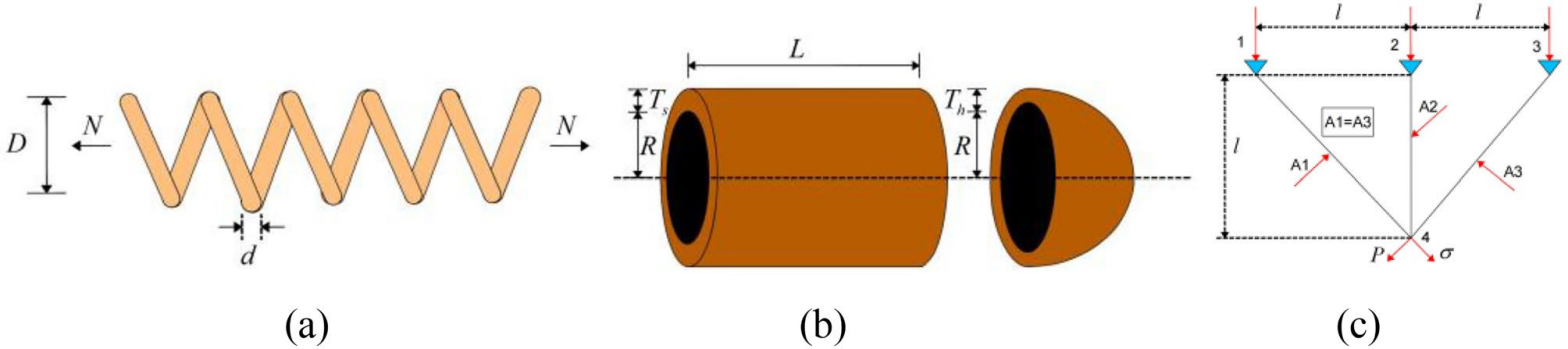
(**a**) Tension/compression spring design problem, (**b**) pressure vessel design problem and (**c**) three-bar truss design problem.

TCSD involves finding the minimum weight of a tension/compression spring, as depicted in Fig. 18a. The mathematical description of TCTD is presented below. Table 8 presents the optimal results on the TCSD problem compared with 10 new optimization algorithms, namely, MVO^57^, EEGWO^58^, CSA^59^, GSA^60^, SO^42^, OBLGOA^61^, SMONM^62^, BGWO^63^, MGPEA^64^, TTAO^65^. The result is $$\overrightarrow {x} = \left[ {x_{1} \, x_{2} \, x_{3} } \right] = \left[ {d \, D \, N} \right]$$$$= \left[ {0.05200,0.36500,10.82120} \right]$$, and the minimum weight is $$f\left( {\vec{\user2{x}}} \right) = 0.012665$$.

**Table 8:** The optimal results on TCSD problem.

Algorithm	$d$	$D$	$N$	$f_{\min }$
MVO	0.05251	0.37602	10.33513	0.012790
EEGWO	0.051673	0.35634	11.3113	**0.012665**
CSA	0.051689	0.356717	11.289012	0.0126652
GSA	0.05000	0.317312	14.22867	0.0128739
SO	0.05330	0.39360	9.57990	0.012670
OBLGOA	0.053018	0.389532	9.600160	0.012701
SMONM	0.051918	0.362248	10.97194	0.012666
BGWO	0.051621	0.355100	11.384638	**0.012665**
MGPEA	0.051688	0.356701	11.289650	**0.012665**
TTAO	0.051674	0.356352	11.31044	**0.012665**
IMRFO	0.05200	0.36500	10.82120	**0.012665**

Table 8 presents the optimal results solved by IMRFO on the TCSD problem compared with 10 new optimization algorithms.

Consider: $$\overrightarrow {x} = \left[ {x_{1} \, x_{2} \, x_{3} } \right] = \left[ {d \, D \, N} \right]$$ Minimize: $$\, f\left( {\overrightarrow {{\varvec{x}}} } \right) = \left( {x_{3} + 2} \right)x_{2} x_{1}^{2}$$.

Subject to: $$g_{1} \left( {\overrightarrow {{\varvec{x}}} } \right) = 1 - \frac{{x_{2}^{3} x_{3} }}{{71785x_{4} }} \le 0$$, $$g_{2} \left( {\overrightarrow {{\varvec{x}}} } \right) = \frac{{4x_{2}^{2} - x_{1} x_{2} }}{{12566\left( {x_{2} x_{1}^{3} - x_{1}^{4} } \right)}} + \frac{1}{{5108x_{1}^{2} }} \le 0$$, $$g_{3} \left( {\overrightarrow {{\varvec{x}}} } \right) = 1 - \frac{{140.45x_{1} }}{{x_{2}^{3} x_{3} }} \le 0$$, $$g_{4} \left( {\overrightarrow {{\varvec{x}}} } \right) = \frac{{x_{1} + x_{2} }}{1.5} - 1 \le 0$$.

Parameters range: $$0 \le x_{1} \le 2$$, $$0.25 \le x_{2} \le 1.3$$, $$2 \le x_{3} \le 15$$.

### Engineering problem 2: Pressure vessel design problem (PVD)

The PVD problem^66^ considers minimizing the manufacturing cost of pressure vessels (Fig. 18b). As is shown in Table 9, the optimal results on the PVD problem compared with 8 new optimization algorithms, namely, GA2^67^, CPSO^68^, MPA^22^, MRFO^27^, AO^46^, SFS^69^, AAA^70^, iDEaSm^71^. The result is $\overrightarrow {{\varvec{x}}} = \left[ {x_{1} \, x_{2} \, x_{3} \, x_{4} } \right] = \left[ {T_{s} \, T_{h} \, R \, L} \right] = \left[ {0.7787 \, 0.3854 \, 40.3410 \, 199.9296} \right]$, and the manufacturing cost is $f\left( {\overrightarrow {{\varvec{x}}} } \right) = 5885.5000$.

**Table 9:** Optimal results on PVD problem.

Algorithm	$T_{s}$	$T_{h}$	$R$	$L$	$f_{\min }$
GA2	0.812500	0.437500	42.097398	176.654047	6059.946341
CPSO	0.8125	0.4375	42.098445596	176.636595842	6059.714335048
MPA	0.8125	0.4375	42.098445	176.636607	6059.7144
MRFO	0.7786	0.3870	40.3313	199.8672	5894.6000
AO	1.0540	0.182806	59.6219	38.8050	5949.2258
SFS	0.8125	0.4375	42.0984	176.6366	6059.7143
AAA	1.1391	0.6250	58.2902	43.6927	7197.72893
iDEaSm	0.7782	0.3847	40.3211	199.9802	5988.0275
IMRFO	0.7787	0.3854	40.3410	199.9296	**5885.5000**

Table 9 presents the optimal results solved by IMRFO on the PVD problem compared with 8 new optimization algorithms and MRFO.

Consider: $$\overrightarrow {{\varvec{x}}} = \left[ {x_{1} \, x_{2} \, x_{3} \, x_{4} } \right] = \left[ {T_{s} \, T_{h} \, R \, L} \right]$$.

Minimize: $$f\left( {\overrightarrow {{\varvec{x}}} } \right) = 0.6224x_{1} x_{3} x_{4} + 1.7781x_{2} x_{3}^{2} + 3.1661x_{1}^{2} x_{4} + 19.84x_{1}^{2} x_{3}$$.

Subject to: $$g_{1} \left( {\overrightarrow {{\varvec{x}}} } \right) = - x_{1} + 0.0193x_{3} \le 0$$, $$g_{2} \left( {\overrightarrow {{\varvec{x}}} } \right) = - x_{3} + 0.00954x_{3} \le 0$$, $$g_{3} \left( {\overrightarrow {{\varvec{x}}} } \right) = - \pi x_{3}^{2} x_{4} - \frac{4}{3}\pi x_{3}^{3} + 1296000 \le 0$$, $$g_{4} \left( {\overrightarrow {{\varvec{x}}} } \right) = x_{4} - 240 \le 0$$.

Parameters range: $$0 \le x_{1} ,x_{2} \le 99$$, $$0 \le x_{3} ,x_{4} \le 200$$.

### Engineering problem 3: Three-bar truss design problem (TBTD)

The three-bar truss design problem (TBTD) is to minimize the total weight of the structure (Fig. 18c). Table 10 shows the optimal results of the TBTD problem compared with 8 new optimization algorithms, namely, DSS-MDE^72^, HEA-ACT^73^, DELC^74^, MDPEA^64^, WSA^75^, SSA^76^, GOA^24^, TTAO^27,65^, MRFO^27^. The result is $\overrightarrow {{\varvec{x}}} = \left[ {x_{1} \, x_{2} \, x_{3} } \right] = \left[ {l \, P{ = }\sigma } \right] = \left[ {0.788613317782741 \, 0.408423616744838} \right]$, and the minimum of total weight of the structure is $f\left( {\overrightarrow {{\varvec{x}}} } \right) = 263.895833882434$.

**Table 10:** Optimal results on TBTD Problem.

Algorithm	$l$	$P = \sigma$	$f_{\min }$
DSS-MDE	0.788675	0.408248	263.895843
HEA-ACT	0.788680	0.408234	263.895843
DELC	0.788675	0.408248	263.895843
MDPEA	0.788675	0.408248	263.895843
WSA	0.788683	0.408227	263.89584340
SSA	0.78866541	0.408275784	263.89584
GOA	0.78889755557	0.40761957011	263.89588149
TTAO	0.788688	0.408213	263.8958431
MRFO	0.788895187725278	0.407626505601074	263.895846948519
IMRFO	0.788613317782741	0.408423616744838	**263.895833882434**

Table 10 presents the optimal results solved by IMRFO on the TBTD problem compared with 8 new optimization algorithms and MRFO.

Consider: $$\overrightarrow {{\varvec{x}}} = \left[ {x_{1} \, x_{2} \, x_{3} } \right] = \left[ {l \, P{ = }\sigma } \right]$$. Minimize:$$\, f\left( {\overrightarrow {{\varvec{x}}} } \right) = \left( {2\sqrt 2 x_{1} + x_{2} } \right) \cdot l$$.

Subject to: $g_{1} \left( {\overrightarrow {{\varvec{x}}} } \right) = \frac{{\sqrt 2 x_{1} + x_{2} }}{{\left( {\sqrt 2 x_{1}^{2} + 2x_{1} x_{2} } \right)}}P - \sigma \le 0$, $g_{2} \left( {\overrightarrow {{\varvec{x}}} } \right) = \frac{{x_{2} }}{{\left( {\sqrt 2 x_{1}^{2} + 2x_{1} x_{2} } \right)}}P - \sigma \le 0$, $g_{3} \left( {\overrightarrow {{\varvec{x}}} } \right) = \frac{1}{{\left( {\sqrt 2 x_{2} + x_{1} } \right)}}P - \sigma \le 0$. Parameters range: $0 \le x_{1} ,x_{2} ,x_{3} \le 1$.

Parameters $$l = 100\;{\text{cm}},P = \sigma = 2\;{\text{kN}}/\left( {{\text{cm}}^{2} } \right)$$.

### Engineering problem 4: welded beam design problem (WBD)

**Figure 19:**
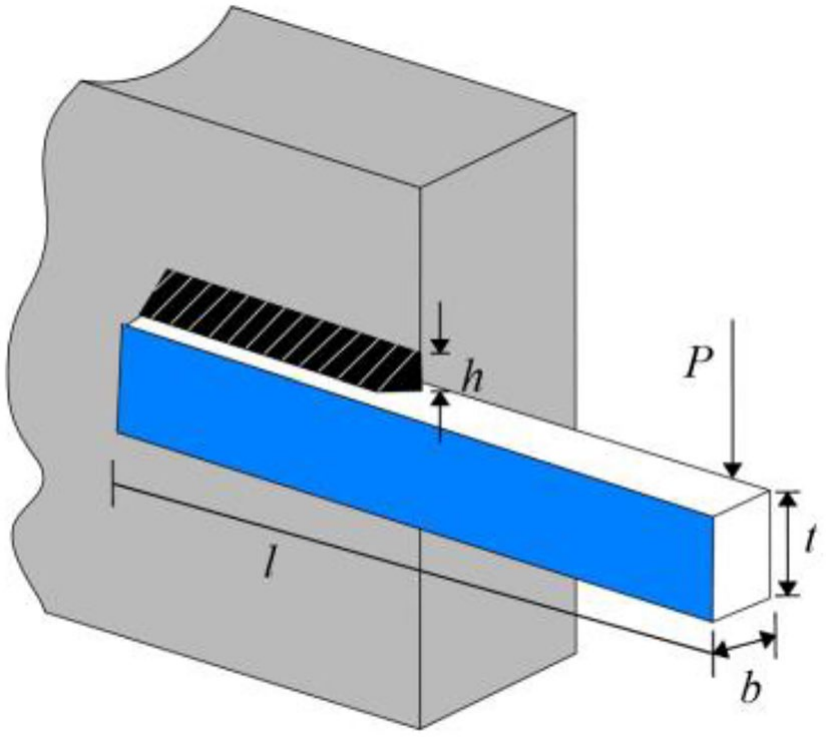
Welded beam design problem.

The welded beam design problem (WBD) is to minimize the fabrication cost of a welded beam (Fig. 19). Table 11 reports the optimal results of the WBD problem compared with 11 new optimization algorithms, namely, EO^77^, LFD^78^, SHO^79^, HGSO^80^, AOS^81^, CDE^82^, OMGSCA^83^, RO^84^, PFA^85^, MRFO^27^, SO^42^. The result is $\overrightarrow {{\varvec{x}}} = \left[ {x_{1} \, x_{2} \, x_{3} {\text{ x}}_{{4}} } \right] = \left[ {h \, l \, t \, b} \right] = \left[ {0.20572964 \, 3.23491935 \, 9.03662391 \, 0.20572964} \right]$, and the minimum of the fabrication cost is $f\left( {\overrightarrow {{\varvec{x}}} } \right) = 1.6927682655$.

**Table 11:** Optimal results on WBD problem.

Algorithm	$h$	$l$	$t$	$b$	$f_{\min }$
EO	0.2057	3.4705	9.0366	0.2057	1.7449
LFD	0.1857	3.9070	9.1552	0.2051	1.77E+00
SHO	0.205563	3.474846	9.035799	0.205811	1.72566
HGSO	0.2054	3.4476	9.0269	0.2060	1.7260
AOS	0.012665233	0.051689535	0.356729145	11.288297130	1.724852309
CDE	0.205730	3.470489	9.036624	0.205730	1.724852
OMGSCA	0.1997	3.3585	9.0576	0.2057	1.7039
RO	0.2037	3.5285	9.0042	0.2072	1.7353
PFA	0.2057	3.4705	9.0366	0.2057	1.7249
MRFO	0.20572964	3.23491937	9.03662391	0.20572964	1.6927682680
SO	0.20572596	3.23499900	9.03661552	0.20573002	1.6927751986
IMRFO	0.20572964	3.23491935	9.03662391	0.20572964	**1.6927682655**

Table 11 presents the optimal results solved by IMRFO on the WBD problem compared with 11 new optimization algorithms.

Consider: $$\overrightarrow {{\varvec{x}}} = \left[ {x_{1} \, x_{2} \, x_{3} {\text{ x}}_{{4}} } \right] = \left[ {h \, l \, t \, b} \right]$$. Minimize: $$\, f\left( {\overrightarrow {{\varvec{x}}} } \right) = 1.10471x_{1}^{2} x_{2} + 0.04811x_{3} x_{4} \left( {14 + x_{2} } \right)$$.

Subject to: $g_{1} \left( {\overrightarrow {{\varvec{x}}} } \right) = \tau \left( {\overrightarrow {{\varvec{x}}} } \right) - \tau_{\max } \le 0$, $g_{2} \left( {\overrightarrow {{\varvec{x}}} } \right) = \sigma \left( {\overrightarrow {{\varvec{x}}} } \right) - \sigma_{\max } \le 0$, $g_{3} \left( {\overrightarrow {{\varvec{x}}} } \right) = \delta \left( {\overrightarrow {{\varvec{x}}} } \right) - \delta_{\max } \le 0$,$g_{4} \left( {\overrightarrow {{\varvec{x}}} } \right) = x_{1} - x_{4} \le 0$, $g_{5} \left( {\overrightarrow {{\varvec{x}}} } \right) = P - P_{c} \left( {\overrightarrow {{\varvec{x}}} } \right) \le 0$, $g_{6} \left( {\overrightarrow {{\varvec{x}}} } \right) = 0.125 - x_{1} \le 0$, $g_{7} \left( {\overrightarrow {{\varvec{x}}} } \right) = 1.10471x_{1}^{2} + 0.04811x_{3} x_{4} \left( {14 + x_{2} } \right) - 5 \le 0$. Parameters range: $0.1 \le x_{1} ,x_{4} \le 2,0.1 \le x_{2} ,x_{3} \le 10$.

Parameters: $$\tau \left( {\overrightarrow {{\varvec{x}}} } \right) = \sqrt {\left( {\tau{\prime} } \right)^{2} + \tau{\prime} \tau^{^{\prime\prime}} \frac{{x_{2} }}{R} + \left( {\tau^{^{\prime\prime}} } \right)^{2} }$$, $$\tau{\prime} = \frac{P}{{\sqrt {2x_{1} x_{2} } }}$$, $$\tau^{^{\prime\prime}} = \frac{MR}{J}$$, $$M = P\left( {L + \frac{{x_{2} }}{2}} \right)$$, $$R = \sqrt {\frac{{x_{2}^{2} + \left( {x_{1} + x_{3} } \right)^{2} }}{4}}$$, $$J = \sqrt 2 x_{1} x_{2} \left( {\frac{{x_{2}^{2} }}{6} + \frac{{\left( {x_{1} + x_{3} } \right)^{2} }}{2}} \right)$$, $$\sigma \left( {\overrightarrow {{\varvec{x}}} } \right) = \frac{6PL}{{x_{4} x_{3}^{2} }}$$, $$\delta \left( {\overrightarrow {{\varvec{x}}} } \right) = \frac{{4PL^{3} }}{{Ex_{4} x_{3}^{3} }}$$, $$P = 60\;{\text{lb}}$$, $$P_{c} \left( {\overrightarrow {{\varvec{x}}} } \right) = \frac{{4.013E\sqrt {\frac{{x_{3}^{2} x_{4}^{6} }}{36}} }}{{L^{2} }}\left( {1 - \frac{{x_{3} }}{2L}\sqrt{\frac{E}{4G}} } \right)$$, $$L = 14\;{\text{in}}$$,$$\delta_{\max } = 0.25\;{\text{in}}$$, $$E = 3 \times 10^{7} \;{\text{psi}}$$, $$G = 12 \times 10^{6} \;{\text{psi}}$$, $$\tau_{\max } = 13600\;{\text{psi}}$$, $$\sigma_{\max } = 30000\;{\text{psi}}$$.

### Engineering problem 5: Gear train design problem (GTD)

**Figure 20:**
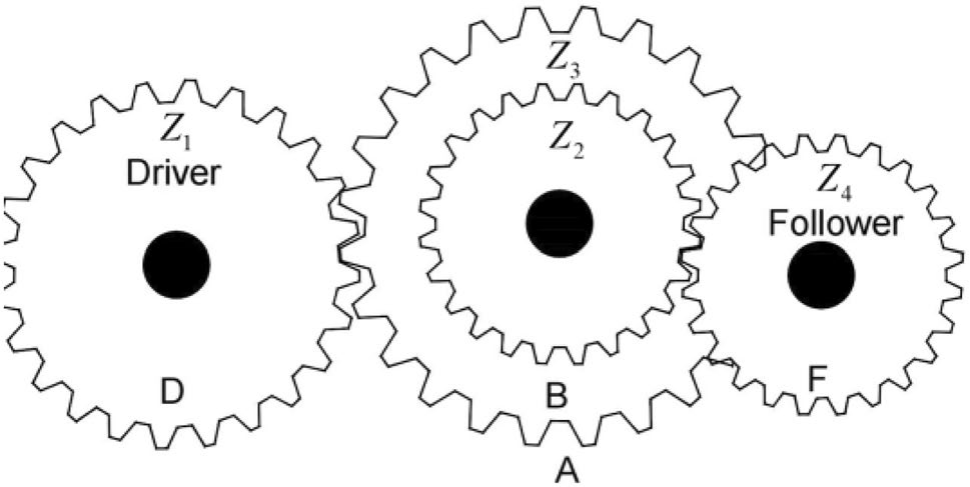
Gear train design problem.

The gear train design problem (GTD) is to minimize the specific transmission costs of the gear train (Fig. 20). Table 12 shows the optimal results of the GTD problem compared with 9 new optimization algorithms, namely, MFO^86^, ABC^87^, MBA^87^, CBO^88^, NNA^87^, SCA^89^, SO^42^, AOA^90^, MRFO^27^. The result is $\overrightarrow {{\varvec{x}}} = \left[ {x_{1} \, x_{2} \, x_{3} {\text{ x}}_{{4}} } \right] = \left[ {N_{a} \, N_{b} \, N_{c} \, N_{d} } \right] = \left[ {42.91783 \, 18.58480 \, 16.30035 \, 49.23052} \right]$, and the minimum of the specific transmission costs of the gear train is $f\left( {\overrightarrow {{\varvec{x}}} } \right) = 2.7009 \, E - 12$.

**Table 12:** Optimal results on GTD Problem.

Algorithm	$N_{a}$	$N_{b}$	$N_{d}$	$N_{f}$	$f_{\min }$
MFO	51	30	13	53	2.3078E−11
ABC	49	16	19	43	**2.7009e**−**12**
MBA	43	16	19	49	**2.7009e**−**12**
CBO	53	13	20	34	2.3078E−11
NNA	49	16	19	43	**2.7009E**−**12**
SCA	43	16	19	49	**2.7009E**−**12**
SO	42.56196	16.35570	19.36730	49.25434	**2.7009 E**−**12**
AOA	46.99578	16.09135	25.09845	58.94793	9.7457E−10
MRFO	43.48570	19.26329	16.28897	48.99045	**2.7009 E**−**12**
IMRFO	42.91783	18.58480	16.30035	49.23052	**2.7009 E**−**12**

Table 12 presents the optimal results solved by IMRFO on the GTD problem compared with 8 new optimization algorithms and MRFO.

Consider: $\overrightarrow {{\varvec{x}}} = \left[ {x_{1} \, x_{2} \, x_{3} {\text{ x}}_{{4}} } \right] = \left[ {N_{a} \, N_{b} \, N_{d} \, N_{f} } \right]$. Minimize: $\, f\left( {\overrightarrow {{\varvec{x}}} } \right) = \left( {\frac{1}{6.391} - \frac{{x_{2} x_{3} }}{{x_{1} x_{4} }}} \right)^{2}$. Parameters range: $12 \le x_{i} \le 60,i = 1,2,3,4$.

The results in Tables 8, 9, 10, 11 and 12 highlight that IMRFO is generally better than MRFO and the other optimizers. IMRFO can improve the global search ability, improve solution accuracy, and solve engineering design problems effectively.

## Conclusion

This paper overcomes the defects of MRFO, such as low solving precision and easily trapped into local optimal, by proposing the IMRFO algorithm, which extends MRFO by incorporating Tent Chaos mapping, bidirectional search, and the Levy flight strategy. The Tent chaos mapping strategy in the algorithm’s initialization phase helps the manta ray to distribute more uniformly and improve the quality of the initial solution. After the cyclone foraging phase, the bidirectional search strategy into the algorithm helps IMRFO for a bidirectional search, expanding the search area and preventing the algorithm from being trapped in a local optimum. During the somersault foraging stage, the Levy flight strategy uses a random step size, strengthening the algorithm’s ability to escape from a local optimum.

To verify IMRFO’s performance, it is evaluated on 23 benchmark functions and the CEC2017 and CEC2022 benchmark suites. The corresponding results highlight that IMRFO has high solving precision and a strong ability to avoid the local optimal compared with the competitors. Secondly, we compared IMRFO with MRFO and three IMRFO modifications on 23 benchmark functions to test the effectiveness of the proposed strategies. The results indicate that the three strategies introduced into MFRO simultaneously can improve the algorithm’s capability compared to one or two strategies. Thirdly, we use statistical analysis such as Friedman mean ranking and the Wilcoxon rank sum test to increase the credibility of the results. The results further confirm the proposed IMRFO's superior performance. Moreover, IMRFO and other algorithms are implemented for 5 engineering design problems. The results demonstrate the competitiveness and applicability of IMRFO compared to other advanced algorithms (Supplemenatry material).

Although IMRFO has been proven competitive, it is still underperforming in some areas, specifically in some hybrid function composition functions. Thus, future research will consider adding other strategies to improve the algorithm. Moreover, IMRFO will be used to solve several real-world problems, such as logistics distribution route planning, laser cutting path planning, and 3D printing layout problems.

### Supplementary Information

Supplementary Information.

## Data Availability

All data generated or analyzed during this study are included in this published article [and its supplementary information files].
