# Supplementary figures and images for: An improved manta ray foraging optimization algorithm (part 2 of 2)
Source: Sci Rep. 2024 May 5;14:10301. doi: 10.1038/s41598-024-59960-1 (PMC11070432; doi:10.1038/s41598-024-59960-1)

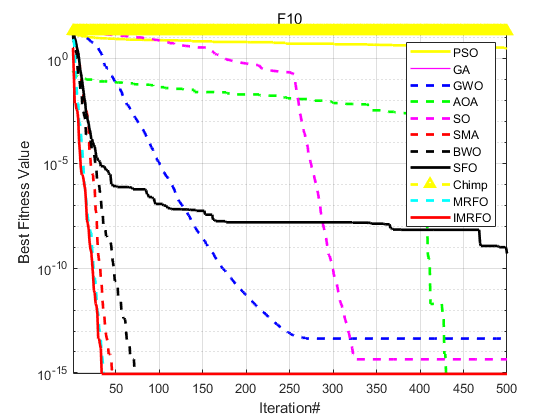

Supplement: Supplementary file 1 — Supplementary Information. [file 41598_2024_59960_MOESM1_ESM.zip › All research figures/All research figures/1 Figures of benchmark functions/Figures of all benchmark functions/10/10-4.tif]

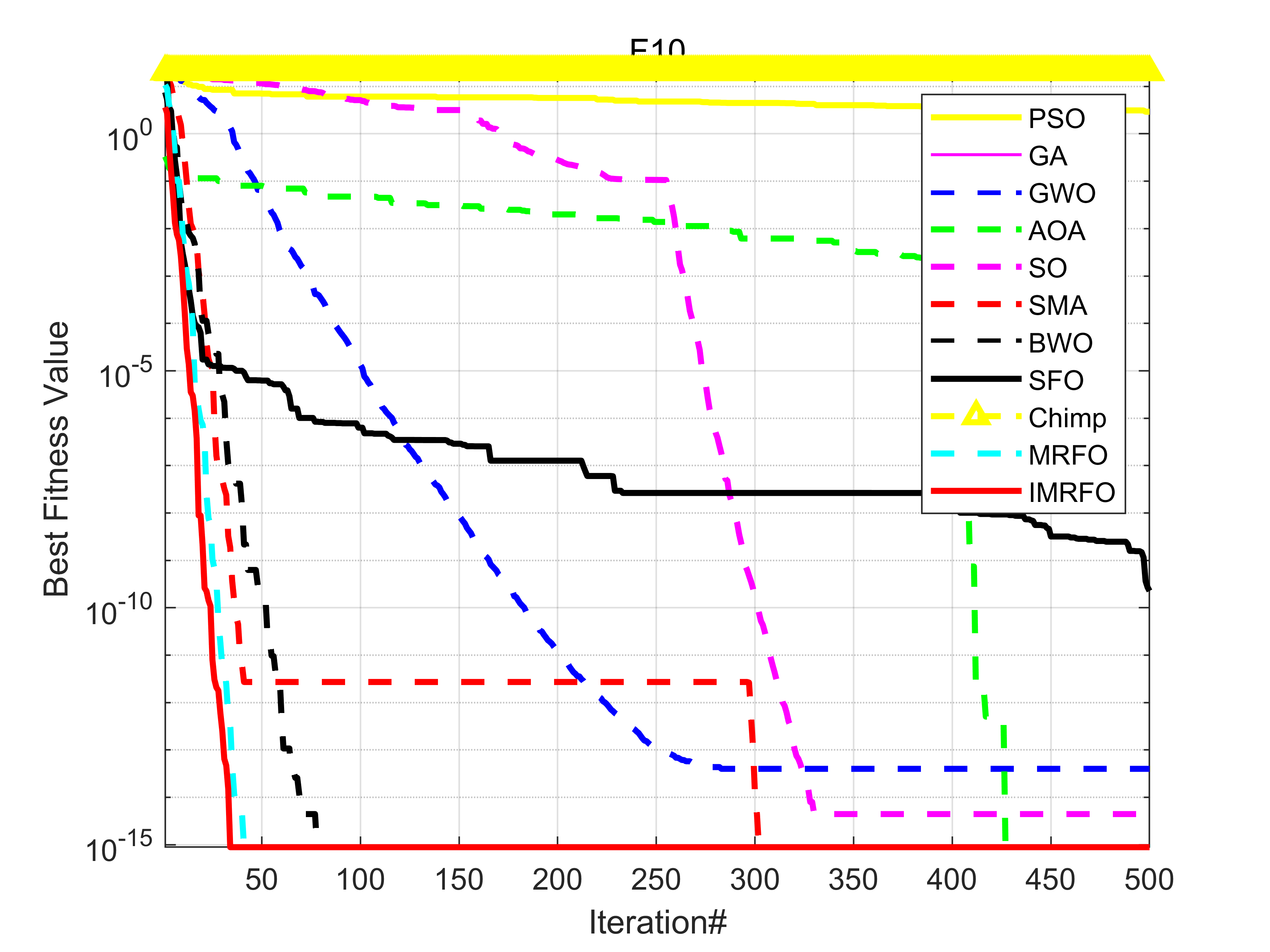

Supplement: Supplementary file 1 — Supplementary Information. [file 41598_2024_59960_MOESM1_ESM.zip › All research figures/All research figures/1 Figures of benchmark functions/Figures of all benchmark functions/10/10-5.tif]

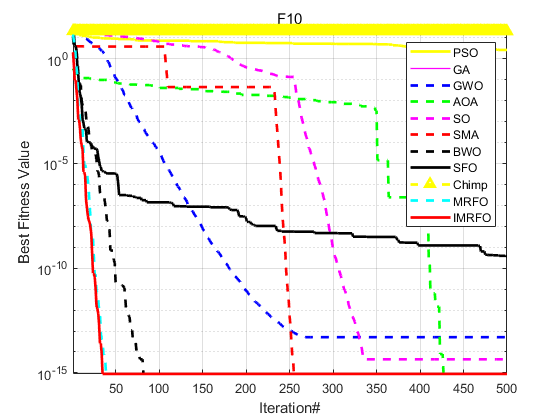

Supplement: Supplementary file 1 — Supplementary Information. [file 41598_2024_59960_MOESM1_ESM.zip › All research figures/All research figures/1 Figures of benchmark functions/Figures of all benchmark functions/10/10-6.tif]

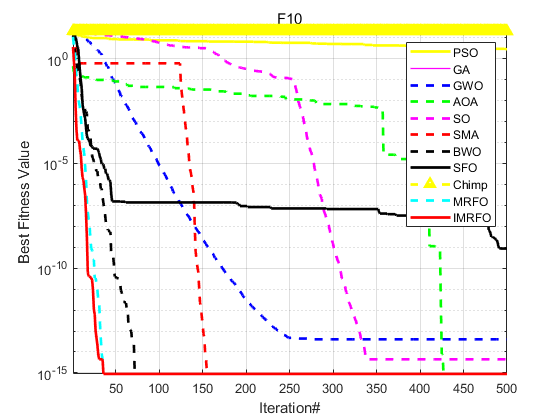

Supplement: Supplementary file 1 — Supplementary Information. [file 41598_2024_59960_MOESM1_ESM.zip › All research figures/All research figures/1 Figures of benchmark functions/Figures of all benchmark functions/10/10-7.tif]

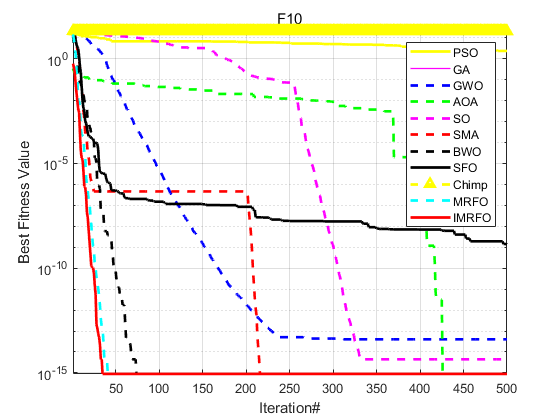

Supplement: Supplementary file 1 — Supplementary Information. [file 41598_2024_59960_MOESM1_ESM.zip › All research figures/All research figures/1 Figures of benchmark functions/Figures of all benchmark functions/10/10-8.tif]

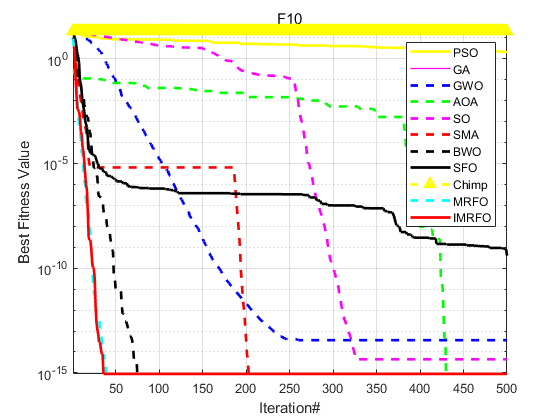

Supplement: Supplementary file 1 — Supplementary Information. [file 41598_2024_59960_MOESM1_ESM.zip › All research figures/All research figures/1 Figures of benchmark functions/Figures of all benchmark functions/10/10-9.tif]

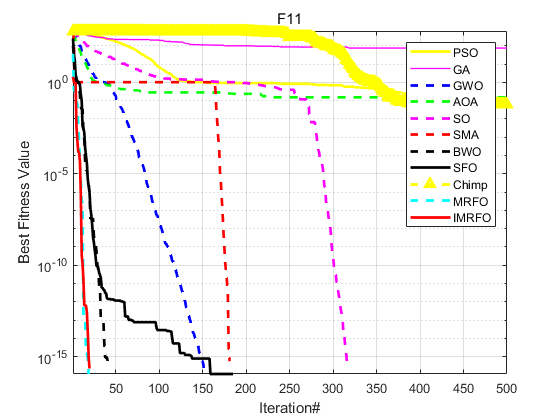

Supplement: Supplementary file 1 — Supplementary Information. [file 41598_2024_59960_MOESM1_ESM.zip › All research figures/All research figures/1 Figures of benchmark functions/Figures of all benchmark functions/11/11-1.tif]

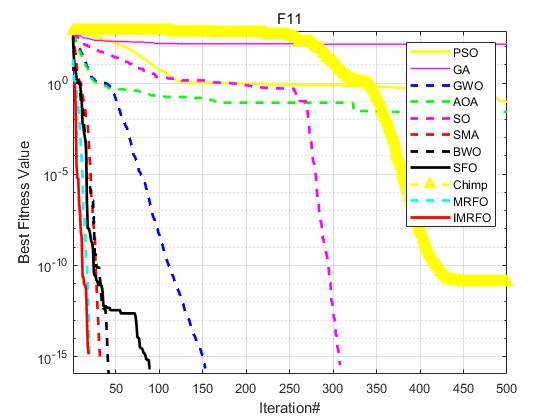

Supplement: Supplementary file 1 — Supplementary Information. [file 41598_2024_59960_MOESM1_ESM.zip › All research figures/All research figures/1 Figures of benchmark functions/Figures of all benchmark functions/11/11-10.tif]

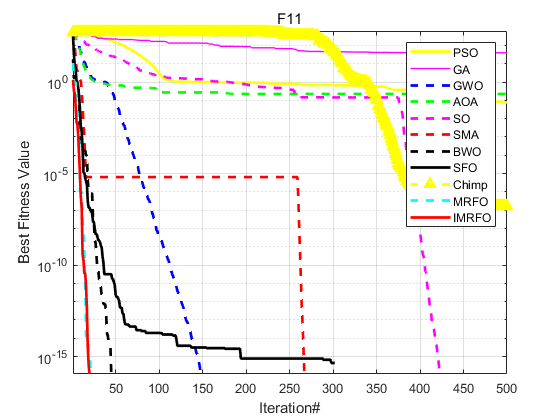

Supplement: Supplementary file 1 — Supplementary Information. [file 41598_2024_59960_MOESM1_ESM.zip › All research figures/All research figures/1 Figures of benchmark functions/Figures of all benchmark functions/11/11-11.tif]

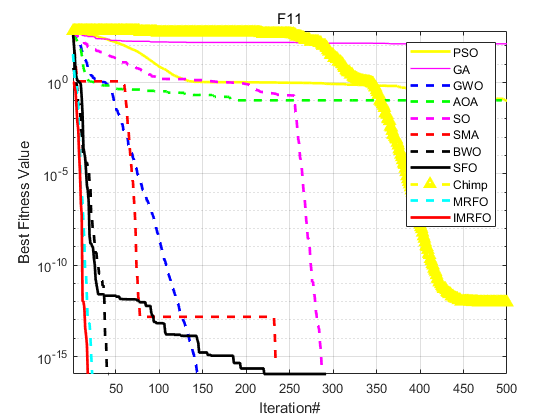

Supplement: Supplementary file 1 — Supplementary Information. [file 41598_2024_59960_MOESM1_ESM.zip › All research figures/All research figures/1 Figures of benchmark functions/Figures of all benchmark functions/11/11-12.tif]

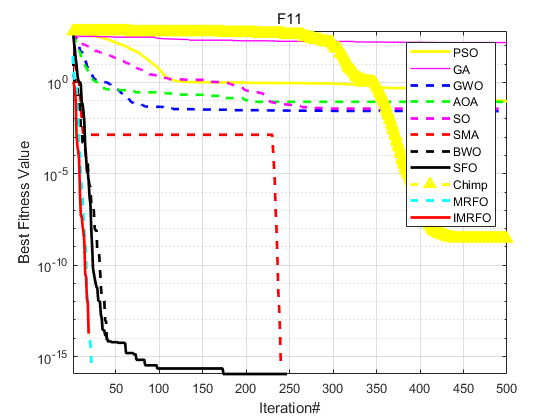

Supplement: Supplementary file 1 — Supplementary Information. [file 41598_2024_59960_MOESM1_ESM.zip › All research figures/All research figures/1 Figures of benchmark functions/Figures of all benchmark functions/11/11-13.tif]

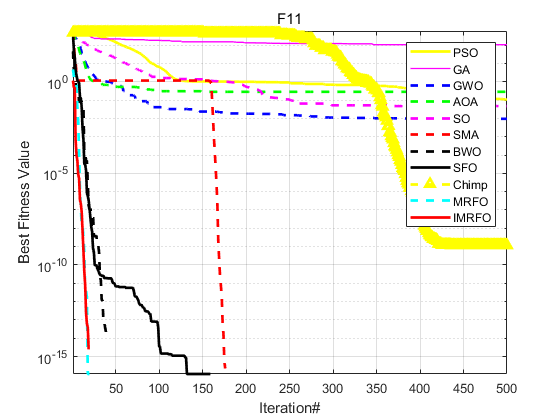

Supplement: Supplementary file 1 — Supplementary Information. [file 41598_2024_59960_MOESM1_ESM.zip › All research figures/All research figures/1 Figures of benchmark functions/Figures of all benchmark functions/11/11-14.tif]

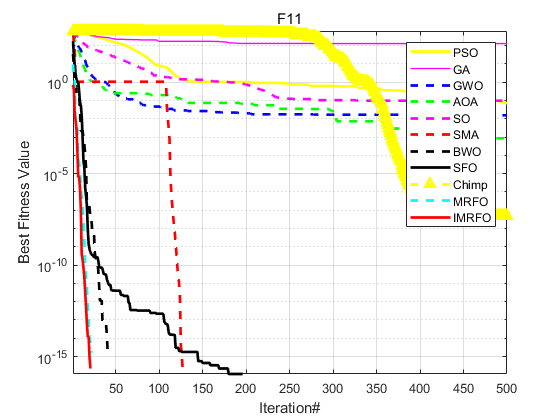

Supplement: Supplementary file 1 — Supplementary Information. [file 41598_2024_59960_MOESM1_ESM.zip › All research figures/All research figures/1 Figures of benchmark functions/Figures of all benchmark functions/11/11-15.tif]

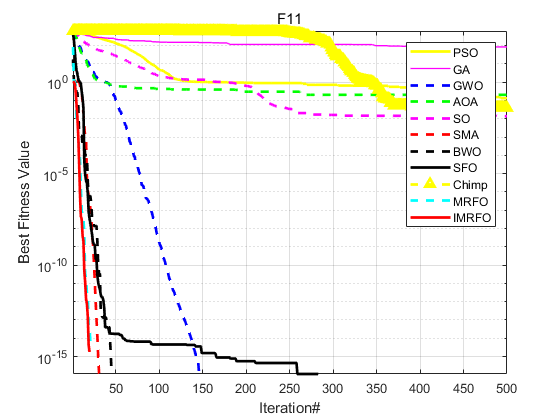

Supplement: Supplementary file 1 — Supplementary Information. [file 41598_2024_59960_MOESM1_ESM.zip › All research figures/All research figures/1 Figures of benchmark functions/Figures of all benchmark functions/11/11-16.tif]

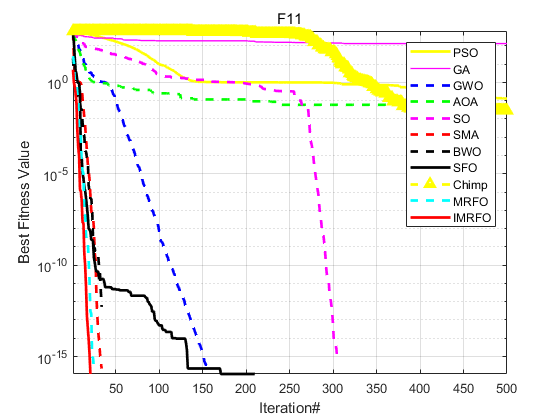

Supplement: Supplementary file 1 — Supplementary Information. [file 41598_2024_59960_MOESM1_ESM.zip › All research figures/All research figures/1 Figures of benchmark functions/Figures of all benchmark functions/11/11-17.tif]

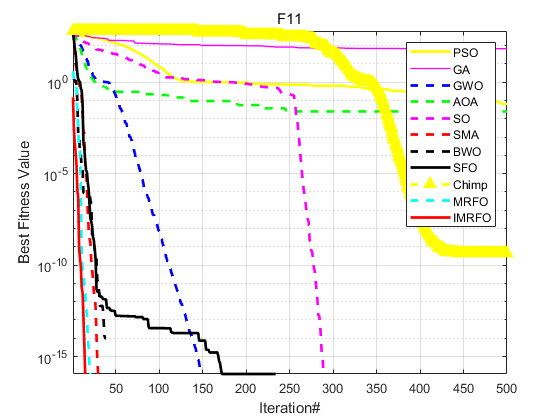

Supplement: Supplementary file 1 — Supplementary Information. [file 41598_2024_59960_MOESM1_ESM.zip › All research figures/All research figures/1 Figures of benchmark functions/Figures of all benchmark functions/11/11-18.tif]

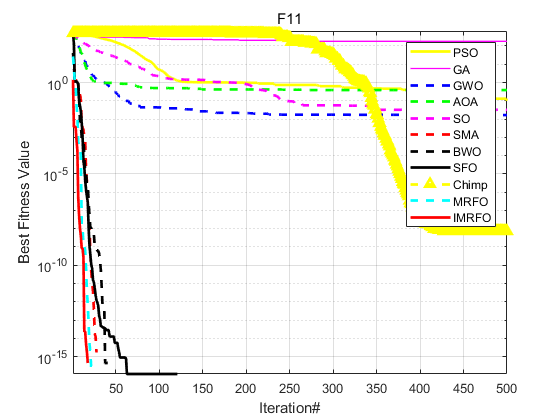

Supplement: Supplementary file 1 — Supplementary Information. [file 41598_2024_59960_MOESM1_ESM.zip › All research figures/All research figures/1 Figures of benchmark functions/Figures of all benchmark functions/11/11-19.tif]

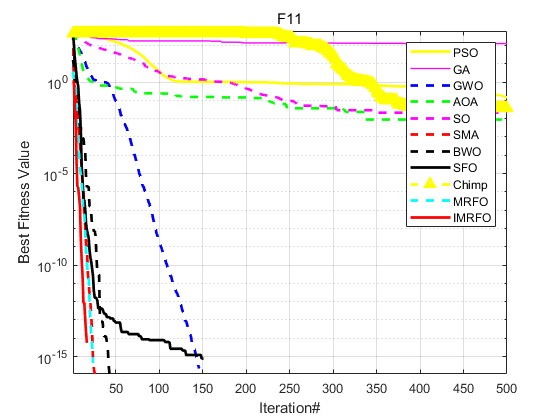

Supplement: Supplementary file 1 — Supplementary Information. [file 41598_2024_59960_MOESM1_ESM.zip › All research figures/All research figures/1 Figures of benchmark functions/Figures of all benchmark functions/11/11-2.tif]

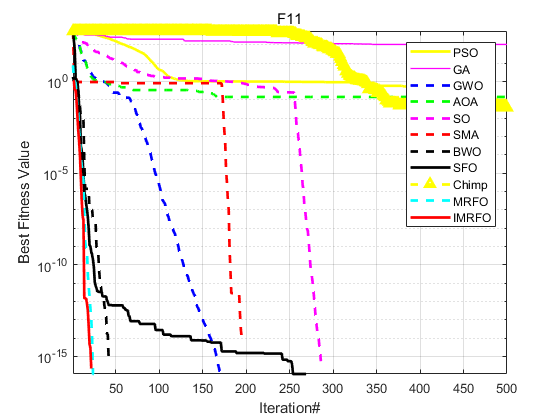

Supplement: Supplementary file 1 — Supplementary Information. [file 41598_2024_59960_MOESM1_ESM.zip › All research figures/All research figures/1 Figures of benchmark functions/Figures of all benchmark functions/11/11-20.tif]

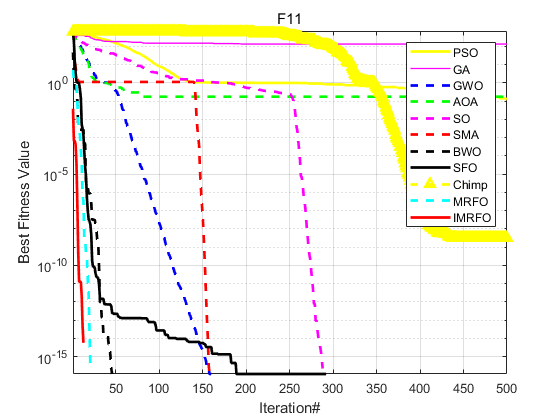

Supplement: Supplementary file 1 — Supplementary Information. [file 41598_2024_59960_MOESM1_ESM.zip › All research figures/All research figures/1 Figures of benchmark functions/Figures of all benchmark functions/11/11-21.tif]

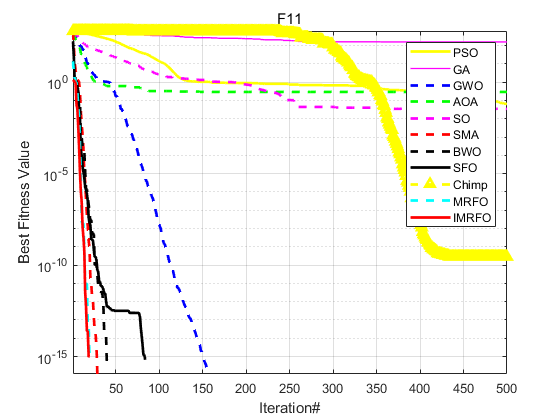

Supplement: Supplementary file 1 — Supplementary Information. [file 41598_2024_59960_MOESM1_ESM.zip › All research figures/All research figures/1 Figures of benchmark functions/Figures of all benchmark functions/11/11-22.tif]

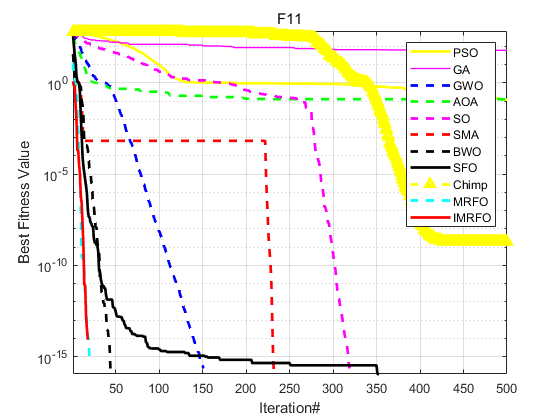

Supplement: Supplementary file 1 — Supplementary Information. [file 41598_2024_59960_MOESM1_ESM.zip › All research figures/All research figures/1 Figures of benchmark functions/Figures of all benchmark functions/11/11-23.tif]

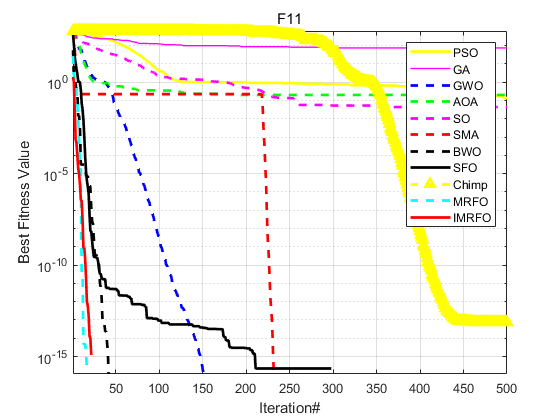

Supplement: Supplementary file 1 — Supplementary Information. [file 41598_2024_59960_MOESM1_ESM.zip › All research figures/All research figures/1 Figures of benchmark functions/Figures of all benchmark functions/11/11-24.tif]

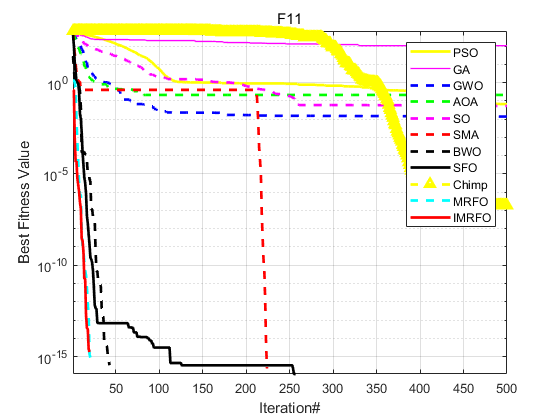

Supplement: Supplementary file 1 — Supplementary Information. [file 41598_2024_59960_MOESM1_ESM.zip › All research figures/All research figures/1 Figures of benchmark functions/Figures of all benchmark functions/11/11-25.tif]

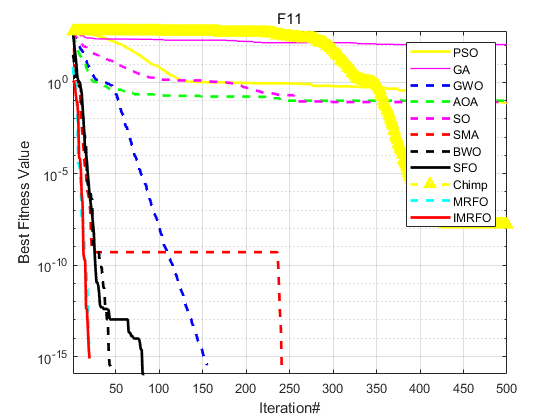

Supplement: Supplementary file 1 — Supplementary Information. [file 41598_2024_59960_MOESM1_ESM.zip › All research figures/All research figures/1 Figures of benchmark functions/Figures of all benchmark functions/11/11-26.tif]

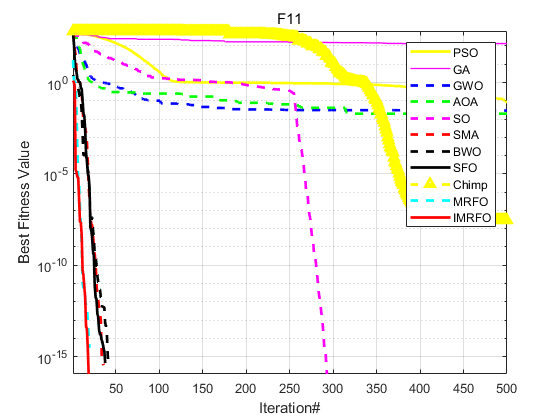

Supplement: Supplementary file 1 — Supplementary Information. [file 41598_2024_59960_MOESM1_ESM.zip › All research figures/All research figures/1 Figures of benchmark functions/Figures of all benchmark functions/11/11-27.tif]

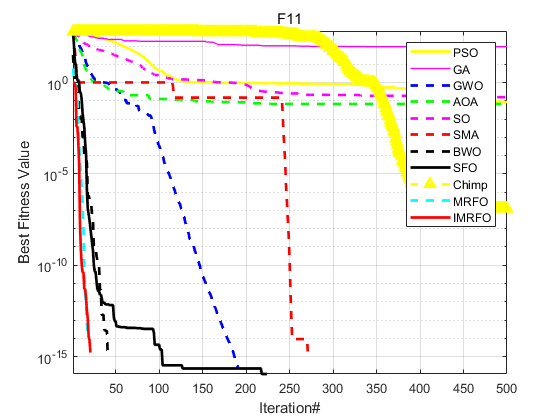

Supplement: Supplementary file 1 — Supplementary Information. [file 41598_2024_59960_MOESM1_ESM.zip › All research figures/All research figures/1 Figures of benchmark functions/Figures of all benchmark functions/11/11-28.tif]

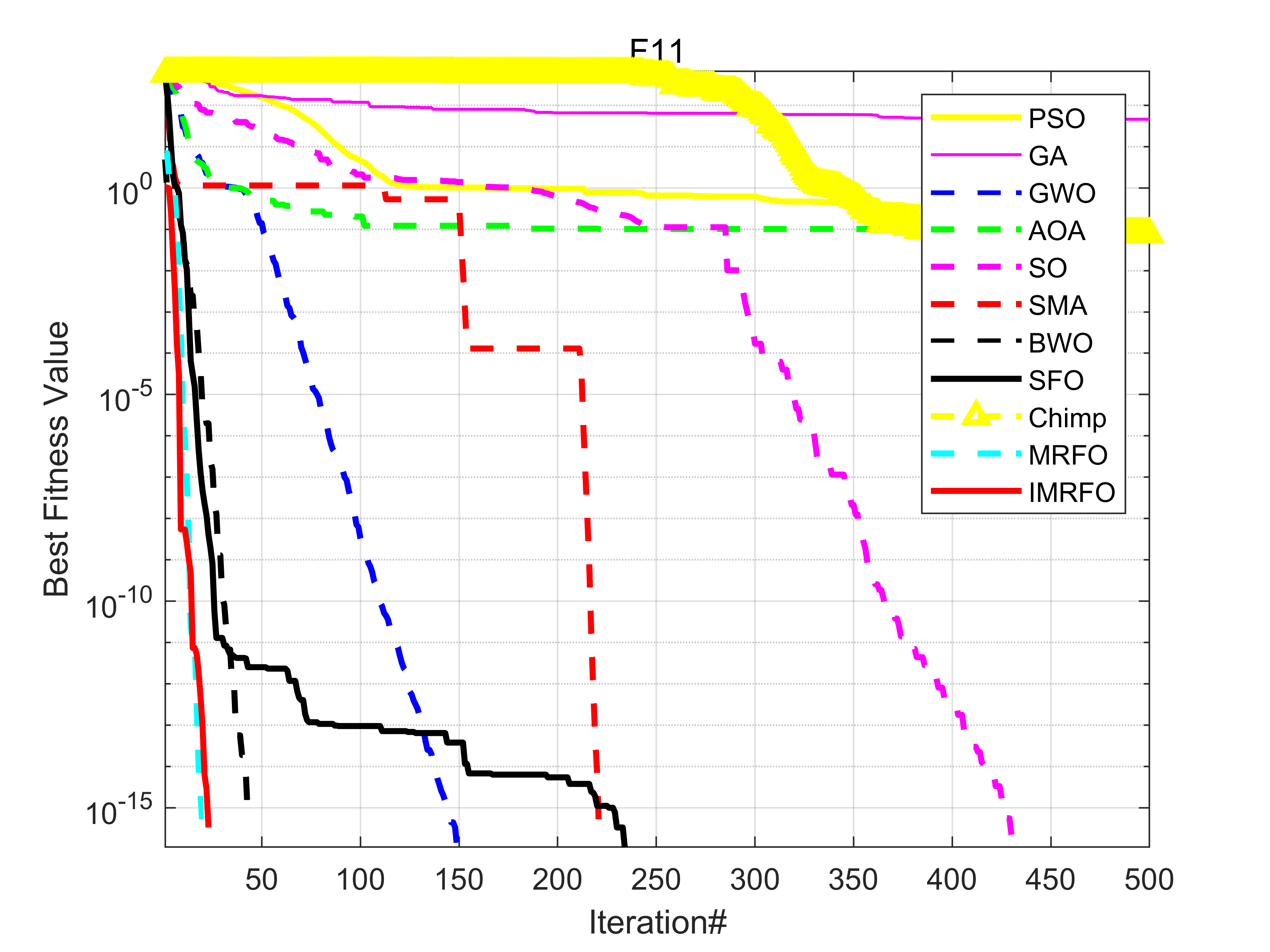

Supplement: Supplementary file 1 — Supplementary Information. [file 41598_2024_59960_MOESM1_ESM.zip › All research figures/All research figures/1 Figures of benchmark functions/Figures of all benchmark functions/11/11-29.tif]

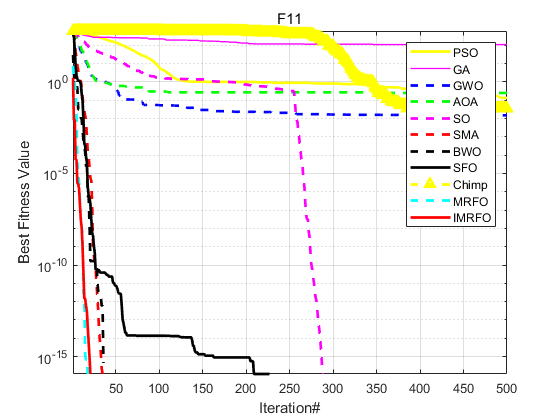

Supplement: Supplementary file 1 — Supplementary Information. [file 41598_2024_59960_MOESM1_ESM.zip › All research figures/All research figures/1 Figures of benchmark functions/Figures of all benchmark functions/11/11-3.tif]

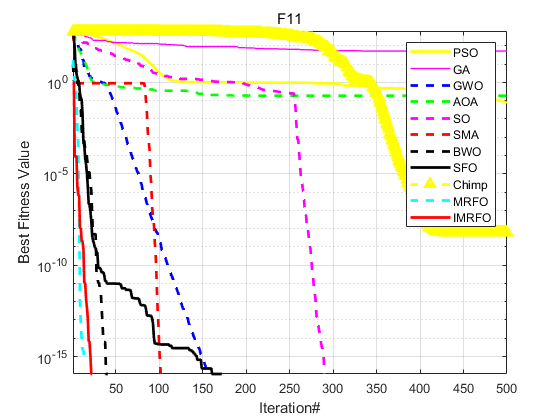

Supplement: Supplementary file 1 — Supplementary Information. [file 41598_2024_59960_MOESM1_ESM.zip › All research figures/All research figures/1 Figures of benchmark functions/Figures of all benchmark functions/11/11-30.tif]

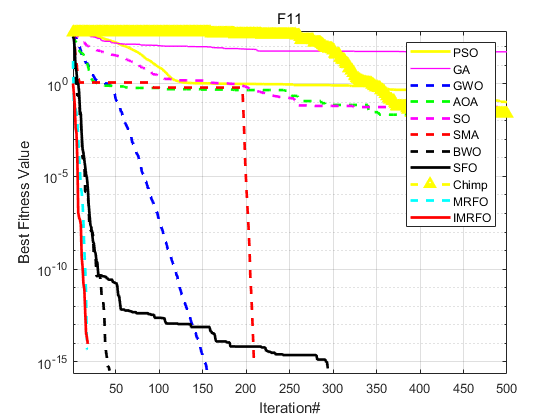

Supplement: Supplementary file 1 — Supplementary Information. [file 41598_2024_59960_MOESM1_ESM.zip › All research figures/All research figures/1 Figures of benchmark functions/Figures of all benchmark functions/11/11-4.tif]

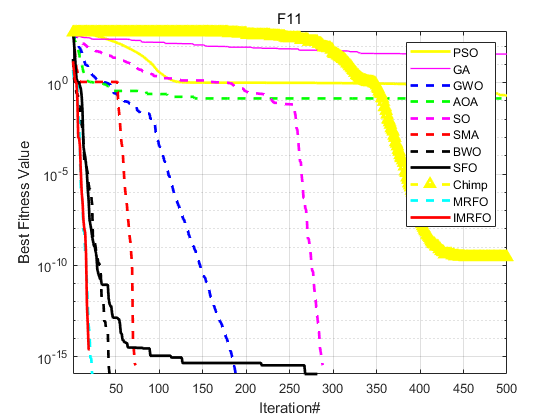

Supplement: Supplementary file 1 — Supplementary Information. [file 41598_2024_59960_MOESM1_ESM.zip › All research figures/All research figures/1 Figures of benchmark functions/Figures of all benchmark functions/11/11-5.tif]

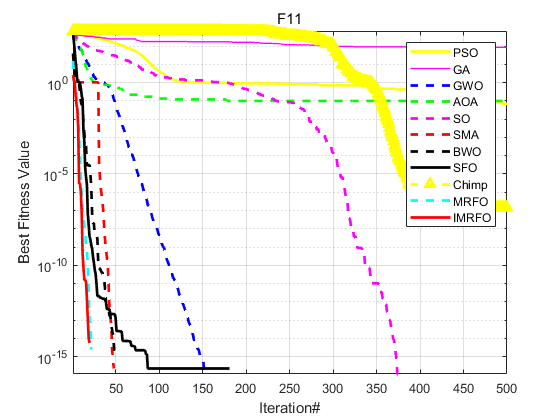

Supplement: Supplementary file 1 — Supplementary Information. [file 41598_2024_59960_MOESM1_ESM.zip › All research figures/All research figures/1 Figures of benchmark functions/Figures of all benchmark functions/11/11-6.tif]

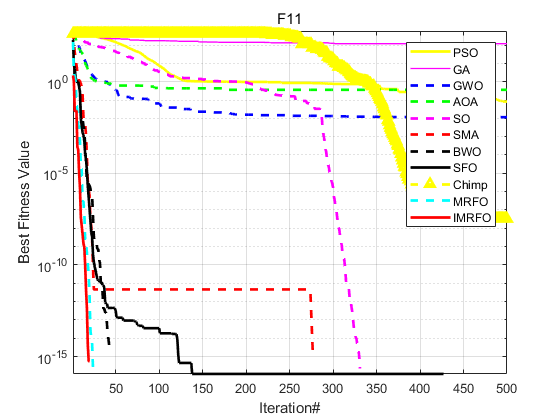

Supplement: Supplementary file 1 — Supplementary Information. [file 41598_2024_59960_MOESM1_ESM.zip › All research figures/All research figures/1 Figures of benchmark functions/Figures of all benchmark functions/11/11-7.tif]

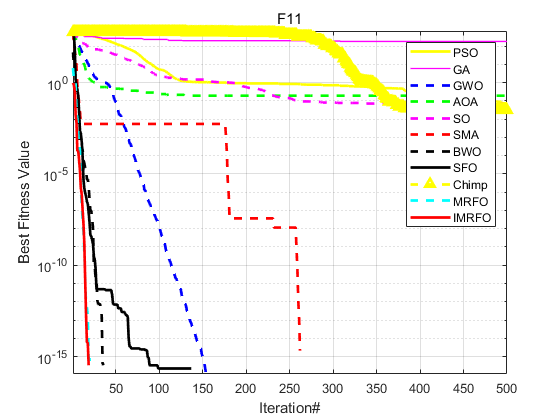

Supplement: Supplementary file 1 — Supplementary Information. [file 41598_2024_59960_MOESM1_ESM.zip › All research figures/All research figures/1 Figures of benchmark functions/Figures of all benchmark functions/11/11-8.tif]

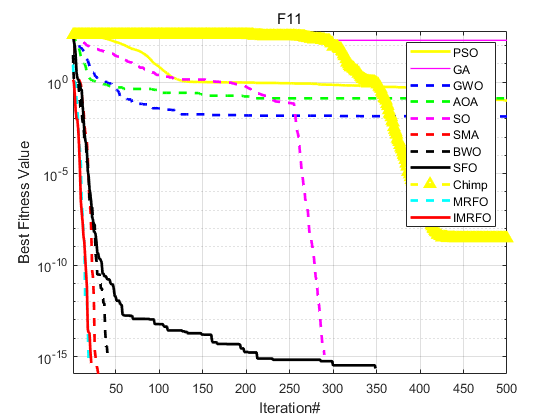

Supplement: Supplementary file 1 — Supplementary Information. [file 41598_2024_59960_MOESM1_ESM.zip › All research figures/All research figures/1 Figures of benchmark functions/Figures of all benchmark functions/11/11-9.tif]

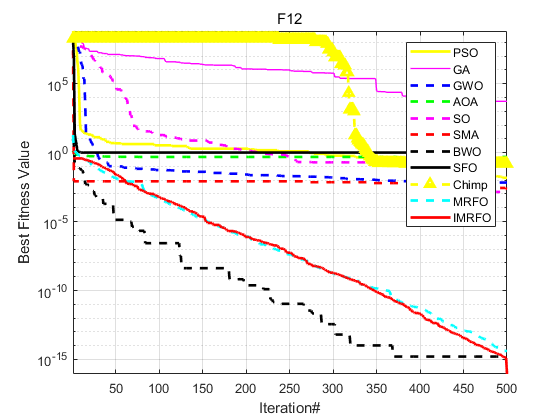

Supplement: Supplementary file 1 — Supplementary Information. [file 41598_2024_59960_MOESM1_ESM.zip › All research figures/All research figures/1 Figures of benchmark functions/Figures of all benchmark functions/12/12-1.tif]

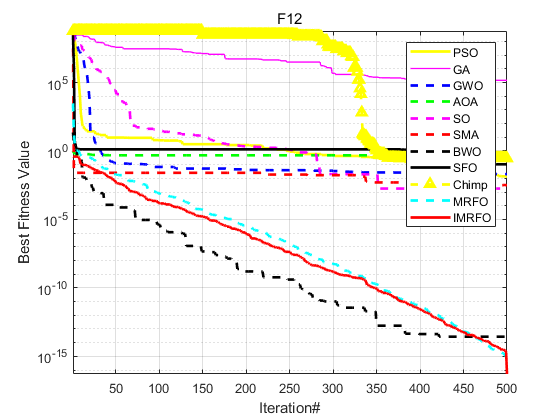

Supplement: Supplementary file 1 — Supplementary Information. [file 41598_2024_59960_MOESM1_ESM.zip › All research figures/All research figures/1 Figures of benchmark functions/Figures of all benchmark functions/12/12-10.tif]

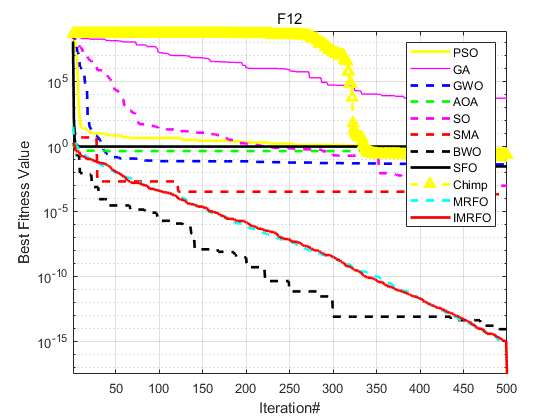

Supplement: Supplementary file 1 — Supplementary Information. [file 41598_2024_59960_MOESM1_ESM.zip › All research figures/All research figures/1 Figures of benchmark functions/Figures of all benchmark functions/12/12-11.tif]

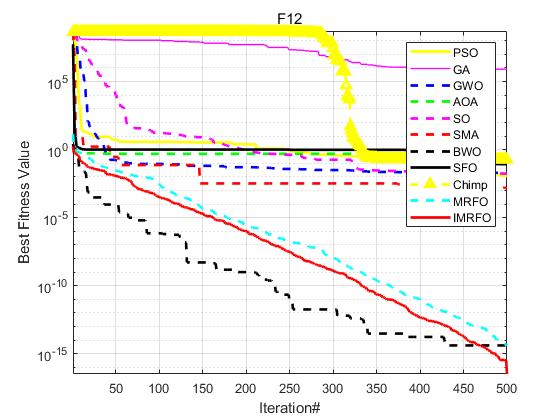

Supplement: Supplementary file 1 — Supplementary Information. [file 41598_2024_59960_MOESM1_ESM.zip › All research figures/All research figures/1 Figures of benchmark functions/Figures of all benchmark functions/12/12-12.tif]

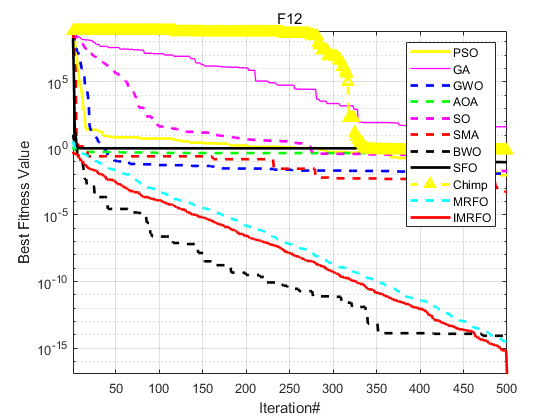

Supplement: Supplementary file 1 — Supplementary Information. [file 41598_2024_59960_MOESM1_ESM.zip › All research figures/All research figures/1 Figures of benchmark functions/Figures of all benchmark functions/12/12-13.tif]

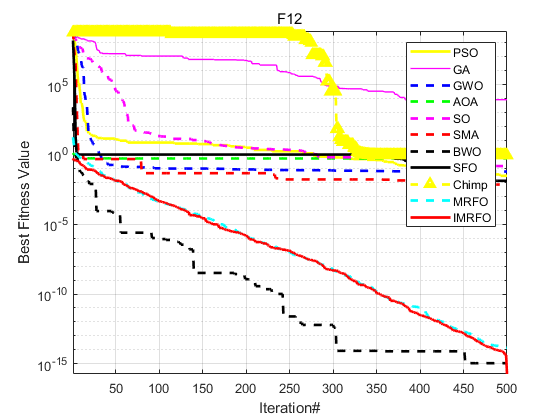

Supplement: Supplementary file 1 — Supplementary Information. [file 41598_2024_59960_MOESM1_ESM.zip › All research figures/All research figures/1 Figures of benchmark functions/Figures of all benchmark functions/12/12-14.tif]

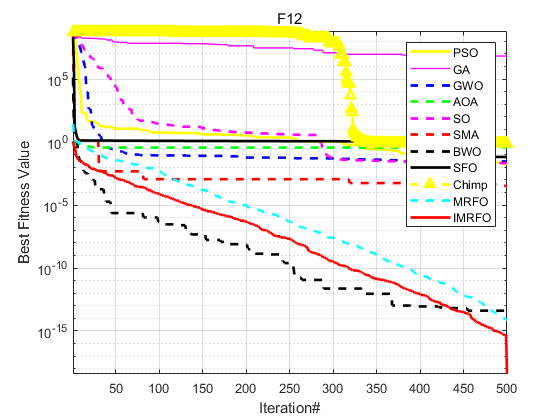

Supplement: Supplementary file 1 — Supplementary Information. [file 41598_2024_59960_MOESM1_ESM.zip › All research figures/All research figures/1 Figures of benchmark functions/Figures of all benchmark functions/12/12-15.tif]

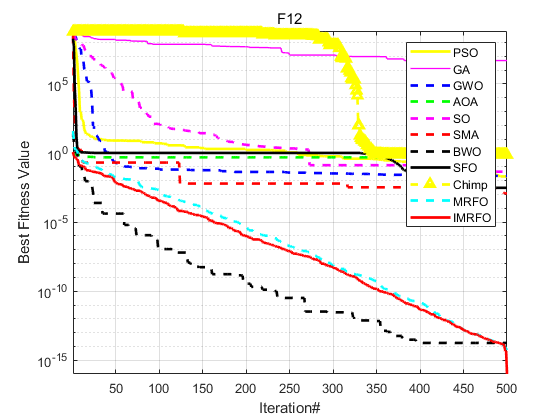

Supplement: Supplementary file 1 — Supplementary Information. [file 41598_2024_59960_MOESM1_ESM.zip › All research figures/All research figures/1 Figures of benchmark functions/Figures of all benchmark functions/12/12-16.tif]

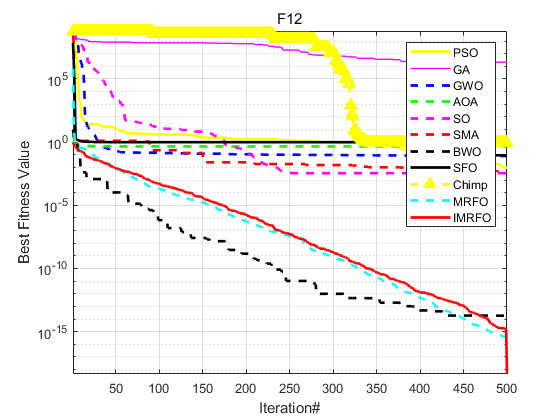

Supplement: Supplementary file 1 — Supplementary Information. [file 41598_2024_59960_MOESM1_ESM.zip › All research figures/All research figures/1 Figures of benchmark functions/Figures of all benchmark functions/12/12-17.tif]

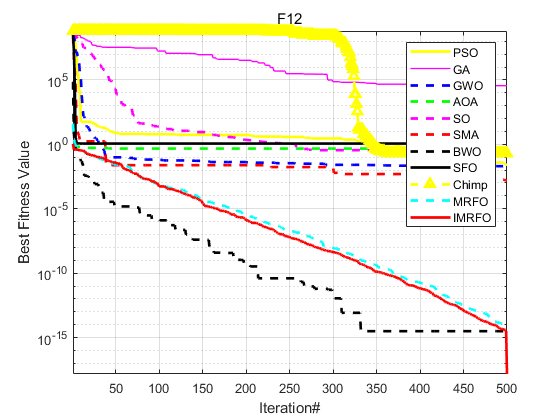

Supplement: Supplementary file 1 — Supplementary Information. [file 41598_2024_59960_MOESM1_ESM.zip › All research figures/All research figures/1 Figures of benchmark functions/Figures of all benchmark functions/12/12-18.tif]

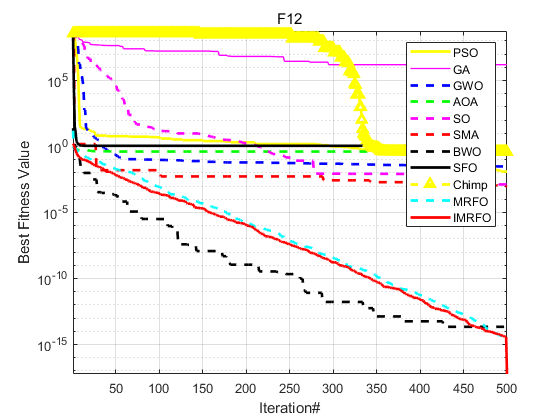

Supplement: Supplementary file 1 — Supplementary Information. [file 41598_2024_59960_MOESM1_ESM.zip › All research figures/All research figures/1 Figures of benchmark functions/Figures of all benchmark functions/12/12-19.tif]

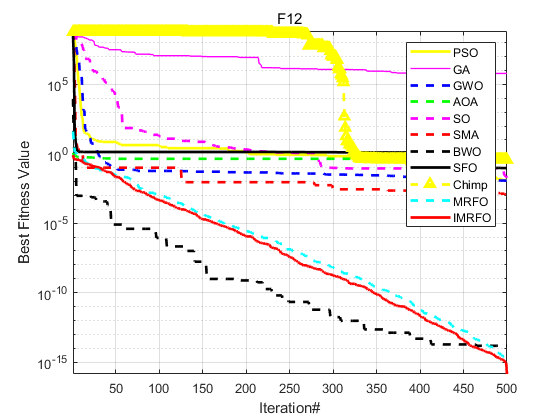

Supplement: Supplementary file 1 — Supplementary Information. [file 41598_2024_59960_MOESM1_ESM.zip › All research figures/All research figures/1 Figures of benchmark functions/Figures of all benchmark functions/12/12-2.tif]

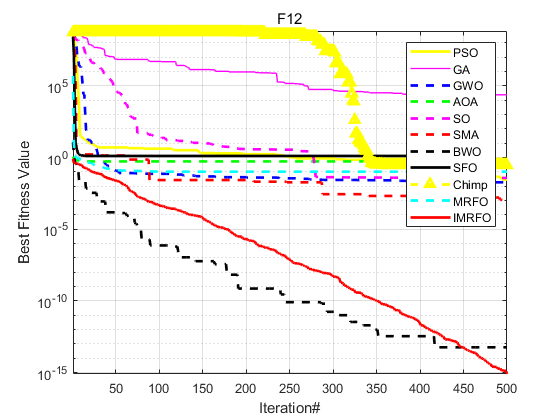

Supplement: Supplementary file 1 — Supplementary Information. [file 41598_2024_59960_MOESM1_ESM.zip › All research figures/All research figures/1 Figures of benchmark functions/Figures of all benchmark functions/12/12-20.tif]

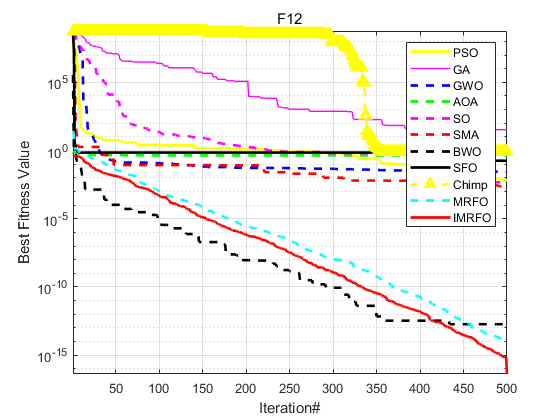

Supplement: Supplementary file 1 — Supplementary Information. [file 41598_2024_59960_MOESM1_ESM.zip › All research figures/All research figures/1 Figures of benchmark functions/Figures of all benchmark functions/12/12-21.tif]

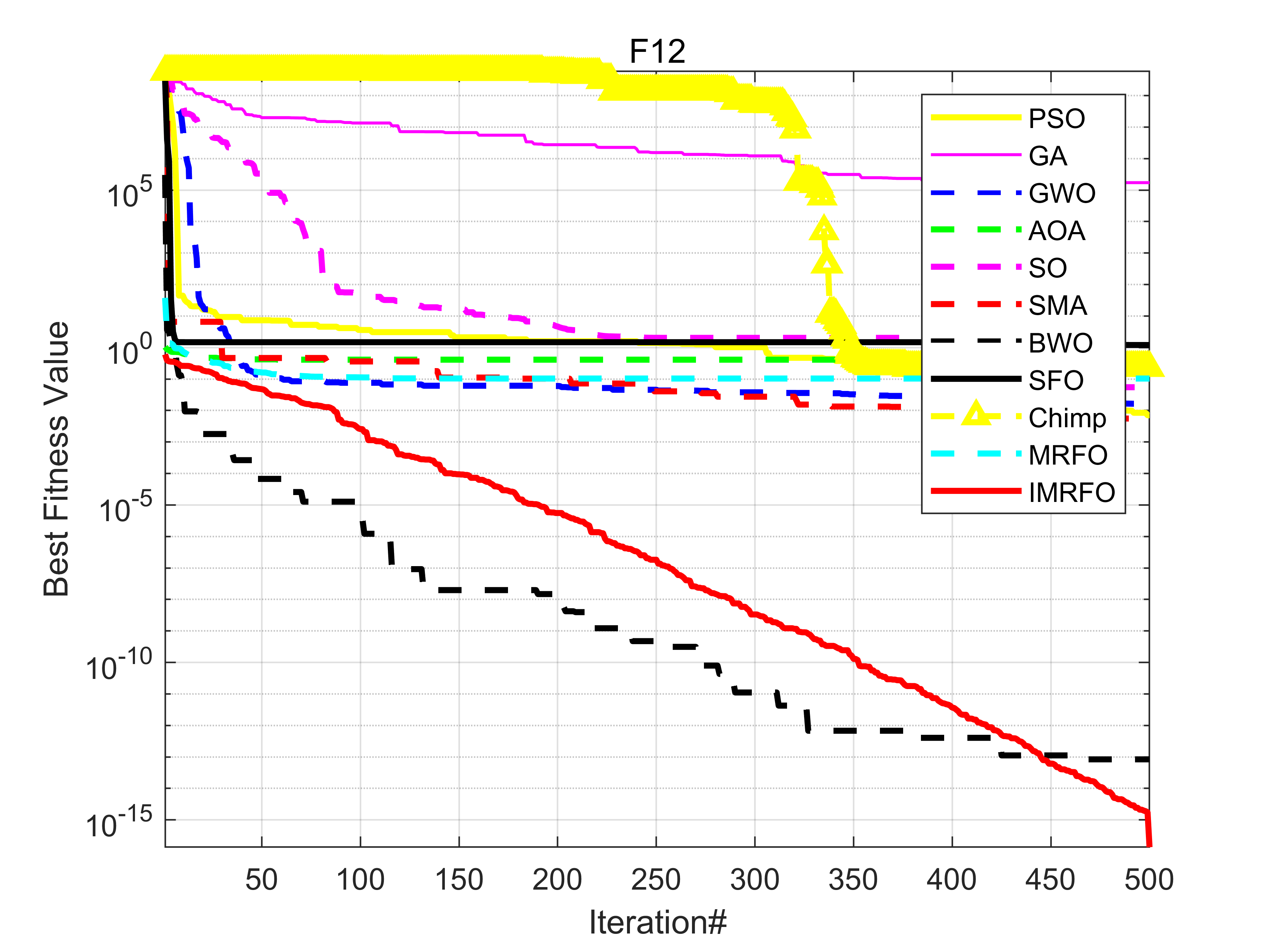

Supplement: Supplementary file 1 — Supplementary Information. [file 41598_2024_59960_MOESM1_ESM.zip › All research figures/All research figures/1 Figures of benchmark functions/Figures of all benchmark functions/12/12-22.tif]

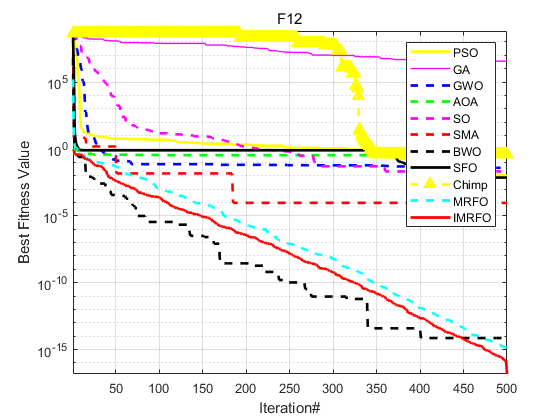

Supplement: Supplementary file 1 — Supplementary Information. [file 41598_2024_59960_MOESM1_ESM.zip › All research figures/All research figures/1 Figures of benchmark functions/Figures of all benchmark functions/12/12-23.tif]

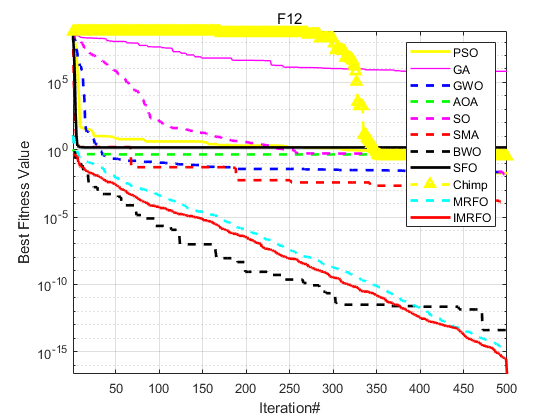

Supplement: Supplementary file 1 — Supplementary Information. [file 41598_2024_59960_MOESM1_ESM.zip › All research figures/All research figures/1 Figures of benchmark functions/Figures of all benchmark functions/12/12-24.tif]

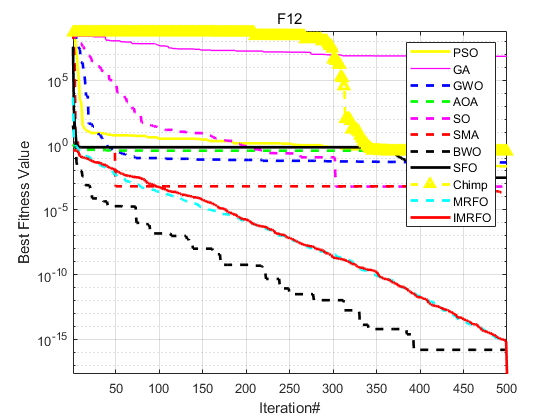

Supplement: Supplementary file 1 — Supplementary Information. [file 41598_2024_59960_MOESM1_ESM.zip › All research figures/All research figures/1 Figures of benchmark functions/Figures of all benchmark functions/12/12-25.tif]

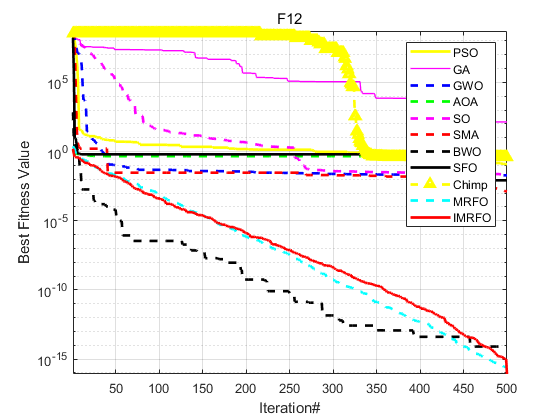

Supplement: Supplementary file 1 — Supplementary Information. [file 41598_2024_59960_MOESM1_ESM.zip › All research figures/All research figures/1 Figures of benchmark functions/Figures of all benchmark functions/12/12-26.tif]

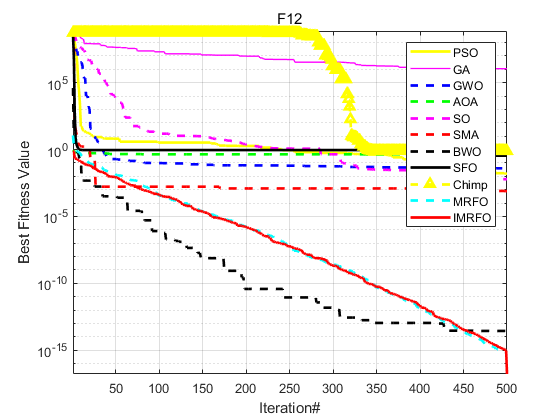

Supplement: Supplementary file 1 — Supplementary Information. [file 41598_2024_59960_MOESM1_ESM.zip › All research figures/All research figures/1 Figures of benchmark functions/Figures of all benchmark functions/12/12-27.tif]

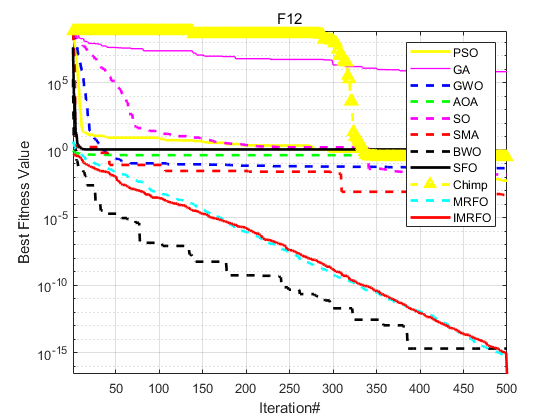

Supplement: Supplementary file 1 — Supplementary Information. [file 41598_2024_59960_MOESM1_ESM.zip › All research figures/All research figures/1 Figures of benchmark functions/Figures of all benchmark functions/12/12-28.tif]

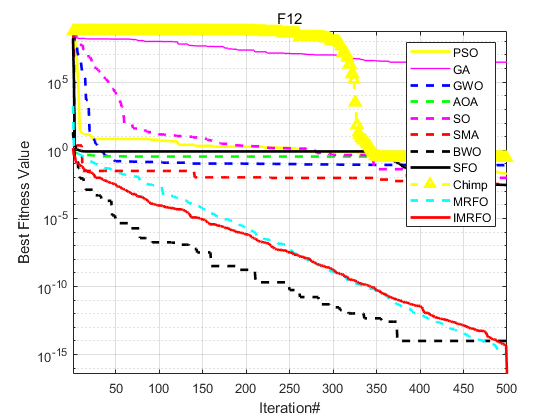

Supplement: Supplementary file 1 — Supplementary Information. [file 41598_2024_59960_MOESM1_ESM.zip › All research figures/All research figures/1 Figures of benchmark functions/Figures of all benchmark functions/12/12-29.tif]

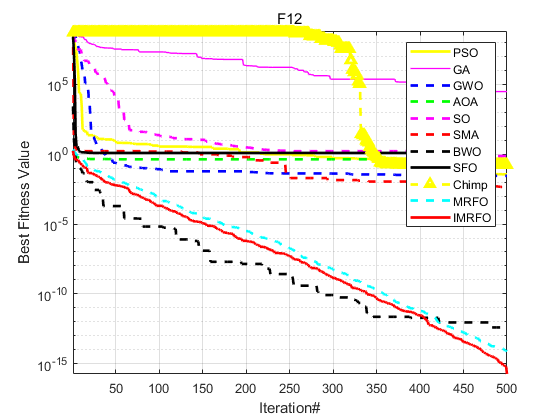

Supplement: Supplementary file 1 — Supplementary Information. [file 41598_2024_59960_MOESM1_ESM.zip › All research figures/All research figures/1 Figures of benchmark functions/Figures of all benchmark functions/12/12-3.tif]

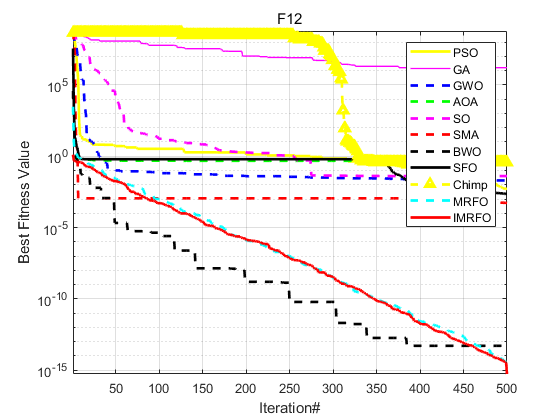

Supplement: Supplementary file 1 — Supplementary Information. [file 41598_2024_59960_MOESM1_ESM.zip › All research figures/All research figures/1 Figures of benchmark functions/Figures of all benchmark functions/12/12-30.tif]

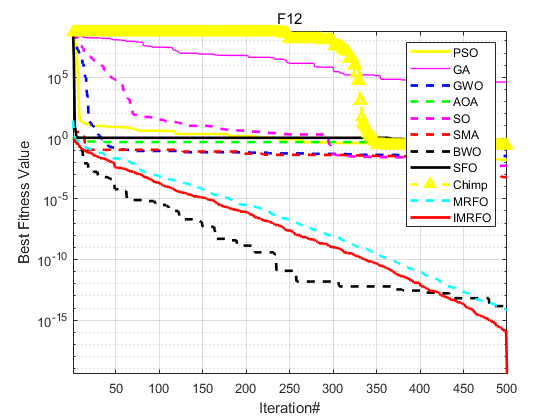

Supplement: Supplementary file 1 — Supplementary Information. [file 41598_2024_59960_MOESM1_ESM.zip › All research figures/All research figures/1 Figures of benchmark functions/Figures of all benchmark functions/12/12-4.tif]

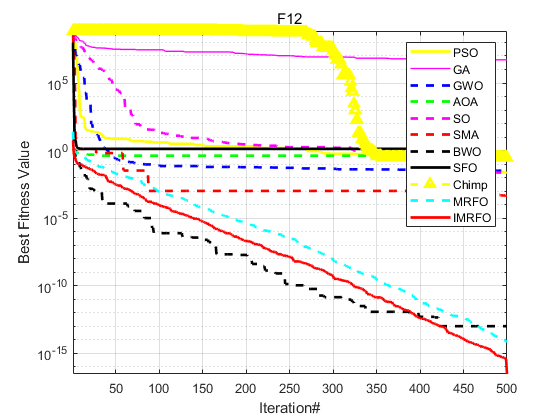

Supplement: Supplementary file 1 — Supplementary Information. [file 41598_2024_59960_MOESM1_ESM.zip › All research figures/All research figures/1 Figures of benchmark functions/Figures of all benchmark functions/12/12-5.tif]

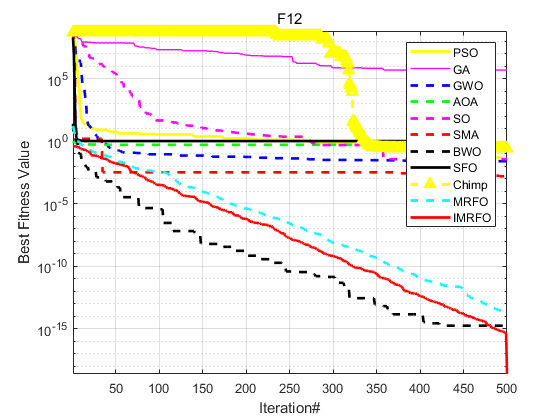

Supplement: Supplementary file 1 — Supplementary Information. [file 41598_2024_59960_MOESM1_ESM.zip › All research figures/All research figures/1 Figures of benchmark functions/Figures of all benchmark functions/12/12-6.tif]

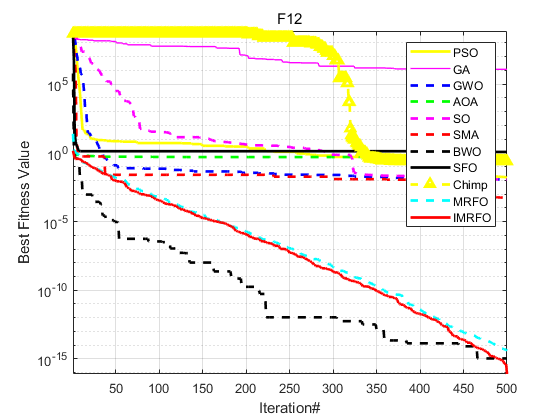

Supplement: Supplementary file 1 — Supplementary Information. [file 41598_2024_59960_MOESM1_ESM.zip › All research figures/All research figures/1 Figures of benchmark functions/Figures of all benchmark functions/12/12-7.tif]

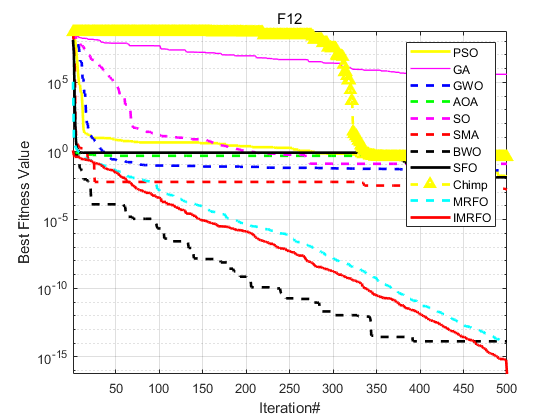

Supplement: Supplementary file 1 — Supplementary Information. [file 41598_2024_59960_MOESM1_ESM.zip › All research figures/All research figures/1 Figures of benchmark functions/Figures of all benchmark functions/12/12-8.tif]

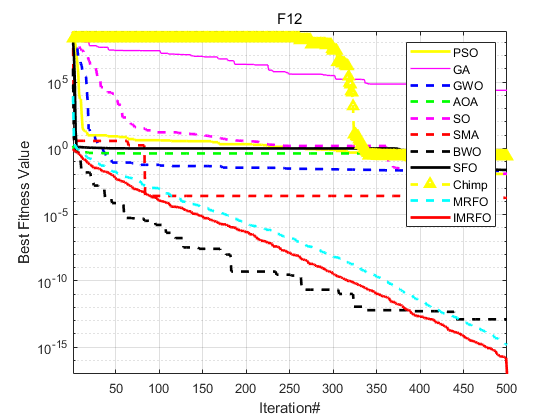

Supplement: Supplementary file 1 — Supplementary Information. [file 41598_2024_59960_MOESM1_ESM.zip › All research figures/All research figures/1 Figures of benchmark functions/Figures of all benchmark functions/12/12-9.tif]

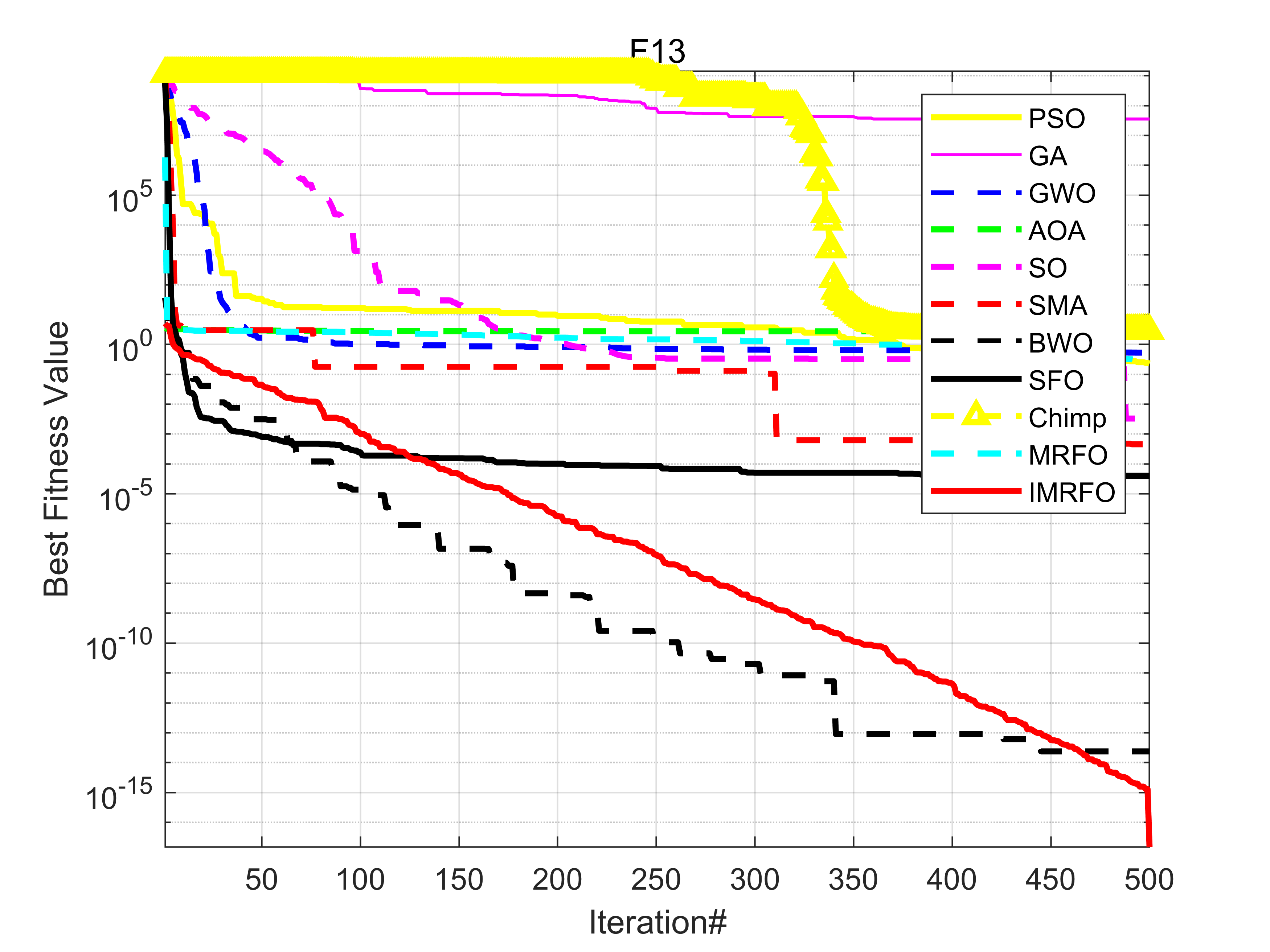

Supplement: Supplementary file 1 — Supplementary Information. [file 41598_2024_59960_MOESM1_ESM.zip › All research figures/All research figures/1 Figures of benchmark functions/Figures of all benchmark functions/12/figure/13-7.tif]

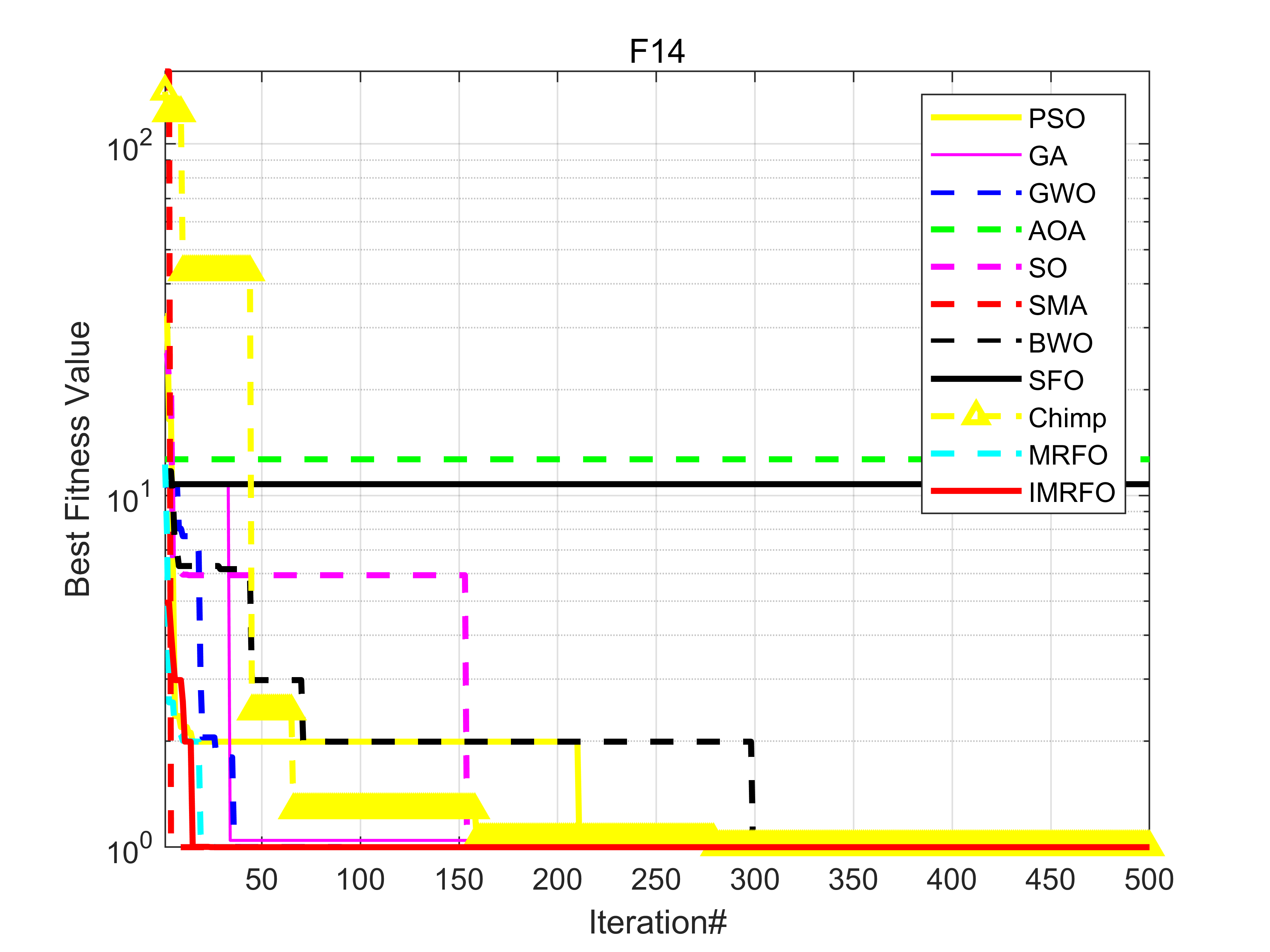

Supplement: Supplementary file 1 — Supplementary Information. [file 41598_2024_59960_MOESM1_ESM.zip › All research figures/All research figures/1 Figures of benchmark functions/Figures of all benchmark functions/12/figure/14-23.tif]

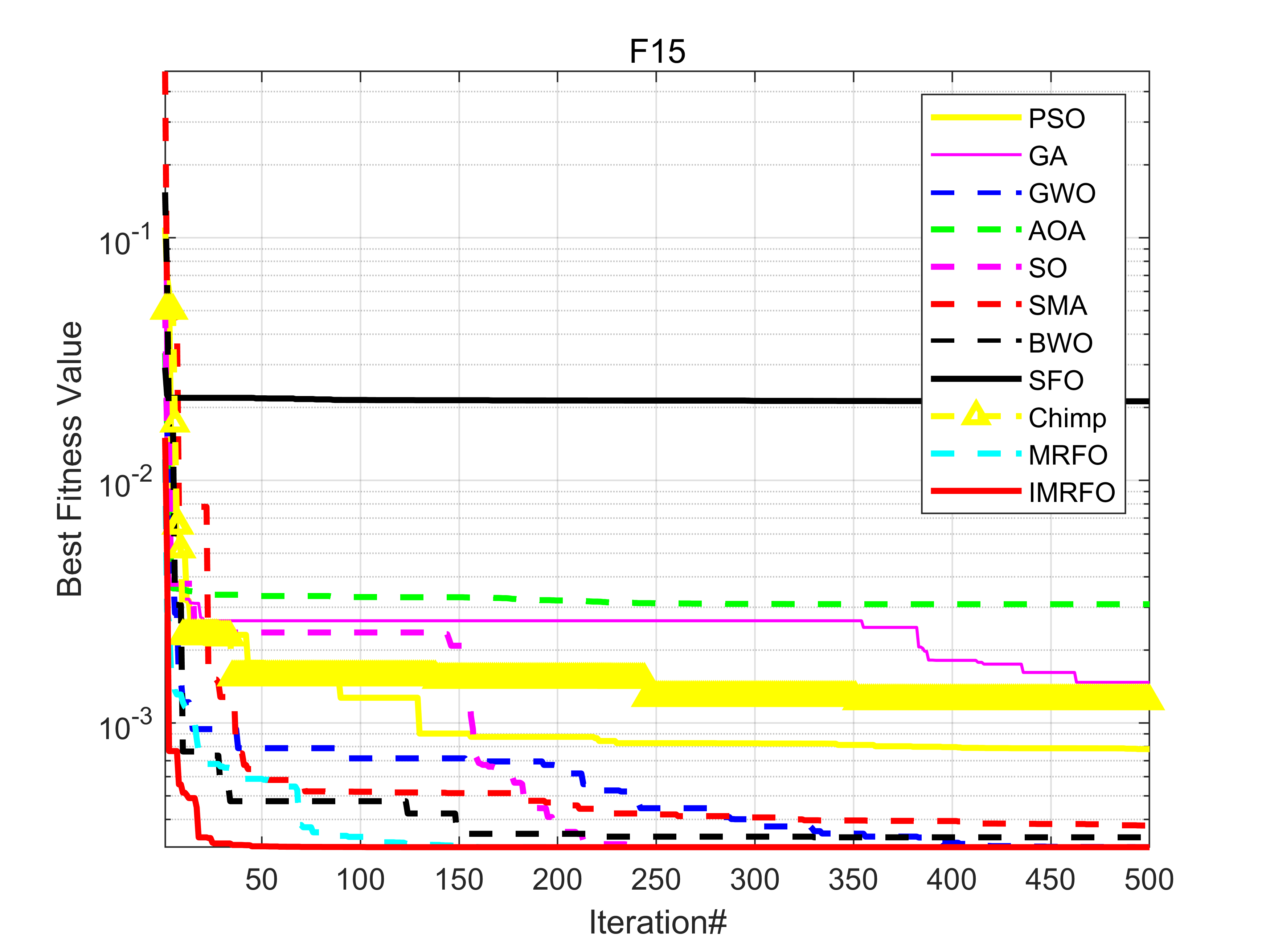

Supplement: Supplementary file 1 — Supplementary Information. [file 41598_2024_59960_MOESM1_ESM.zip › All research figures/All research figures/1 Figures of benchmark functions/Figures of all benchmark functions/12/figure/15-30.tif]

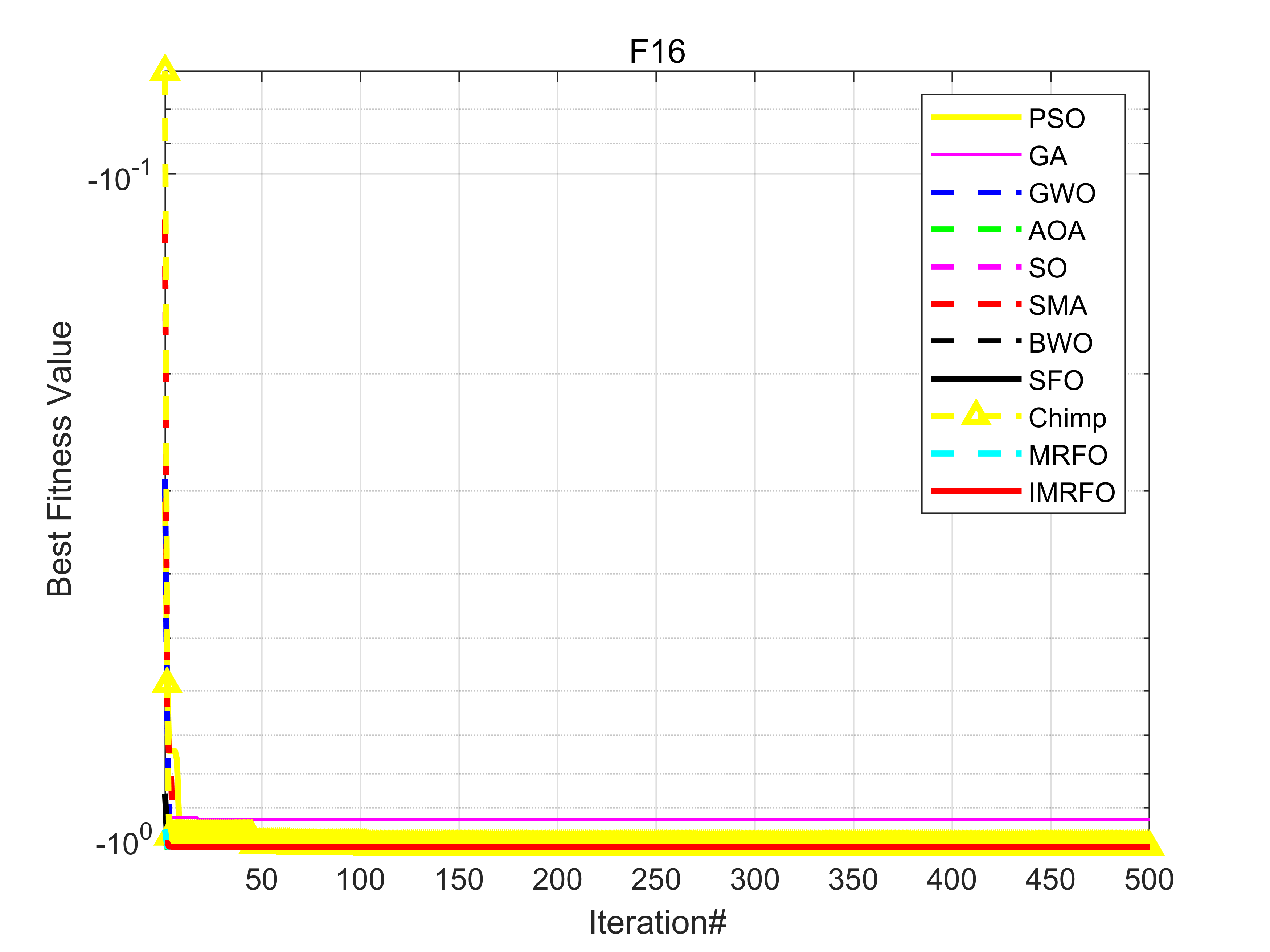

Supplement: Supplementary file 1 — Supplementary Information. [file 41598_2024_59960_MOESM1_ESM.zip › All research figures/All research figures/1 Figures of benchmark functions/Figures of all benchmark functions/12/figure/16-19.tif]

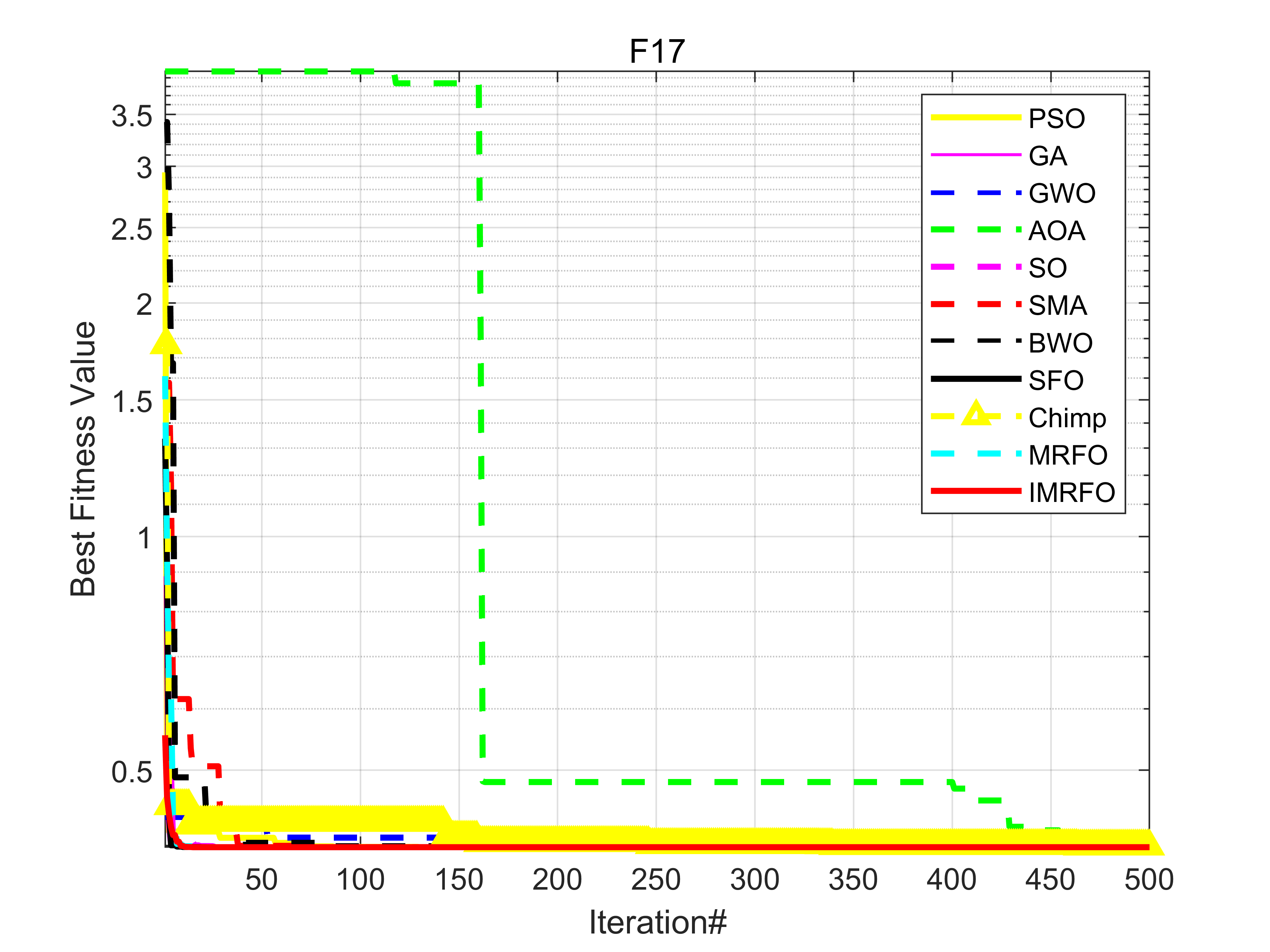

Supplement: Supplementary file 1 — Supplementary Information. [file 41598_2024_59960_MOESM1_ESM.zip › All research figures/All research figures/1 Figures of benchmark functions/Figures of all benchmark functions/12/figure/17-18.tif]

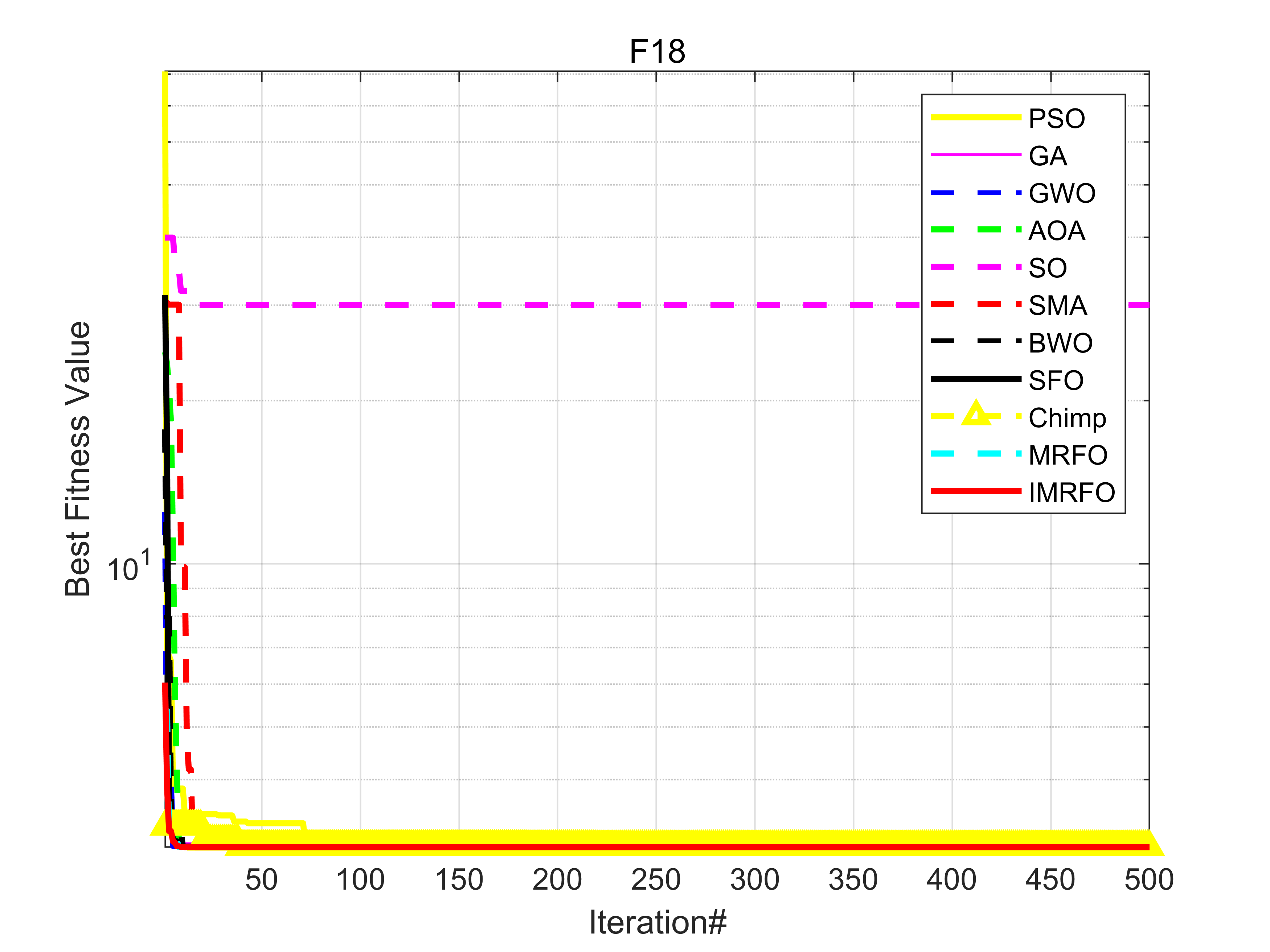

Supplement: Supplementary file 1 — Supplementary Information. [file 41598_2024_59960_MOESM1_ESM.zip › All research figures/All research figures/1 Figures of benchmark functions/Figures of all benchmark functions/12/figure/18-12.tif]

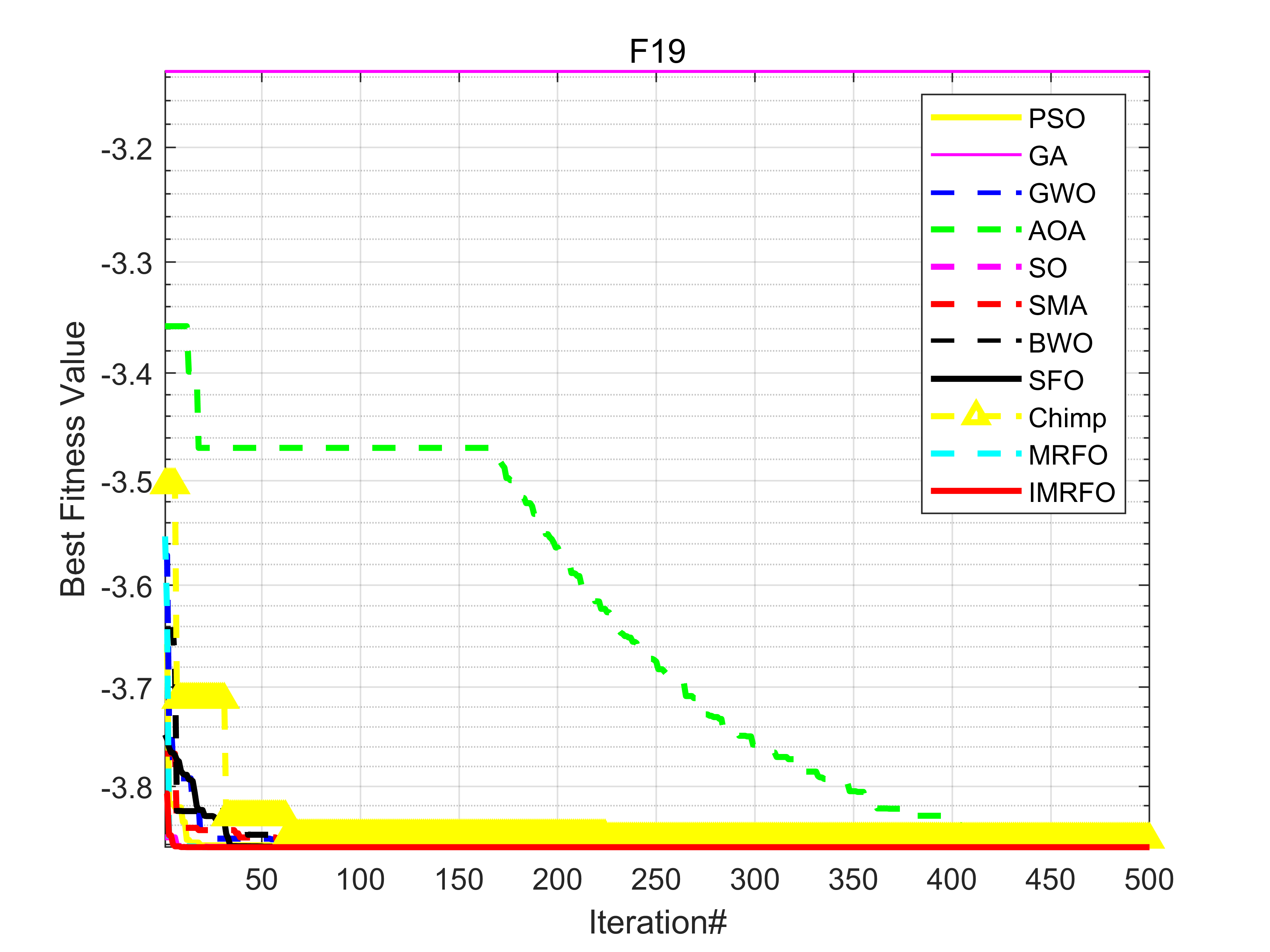

Supplement: Supplementary file 1 — Supplementary Information. [file 41598_2024_59960_MOESM1_ESM.zip › All research figures/All research figures/1 Figures of benchmark functions/Figures of all benchmark functions/12/figure/19-25.tif]

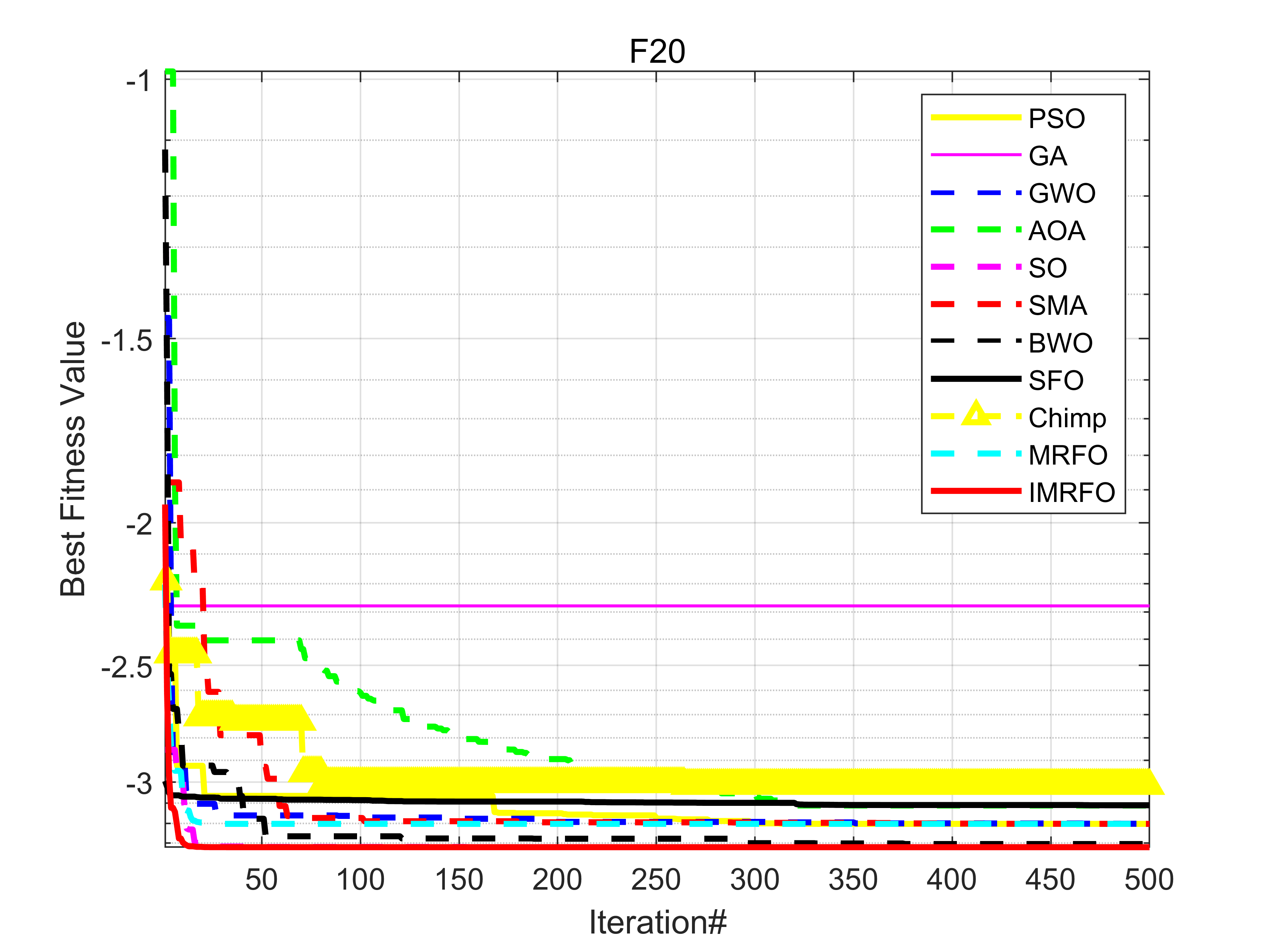

Supplement: Supplementary file 1 — Supplementary Information. [file 41598_2024_59960_MOESM1_ESM.zip › All research figures/All research figures/1 Figures of benchmark functions/Figures of all benchmark functions/12/figure/20-28.tif]

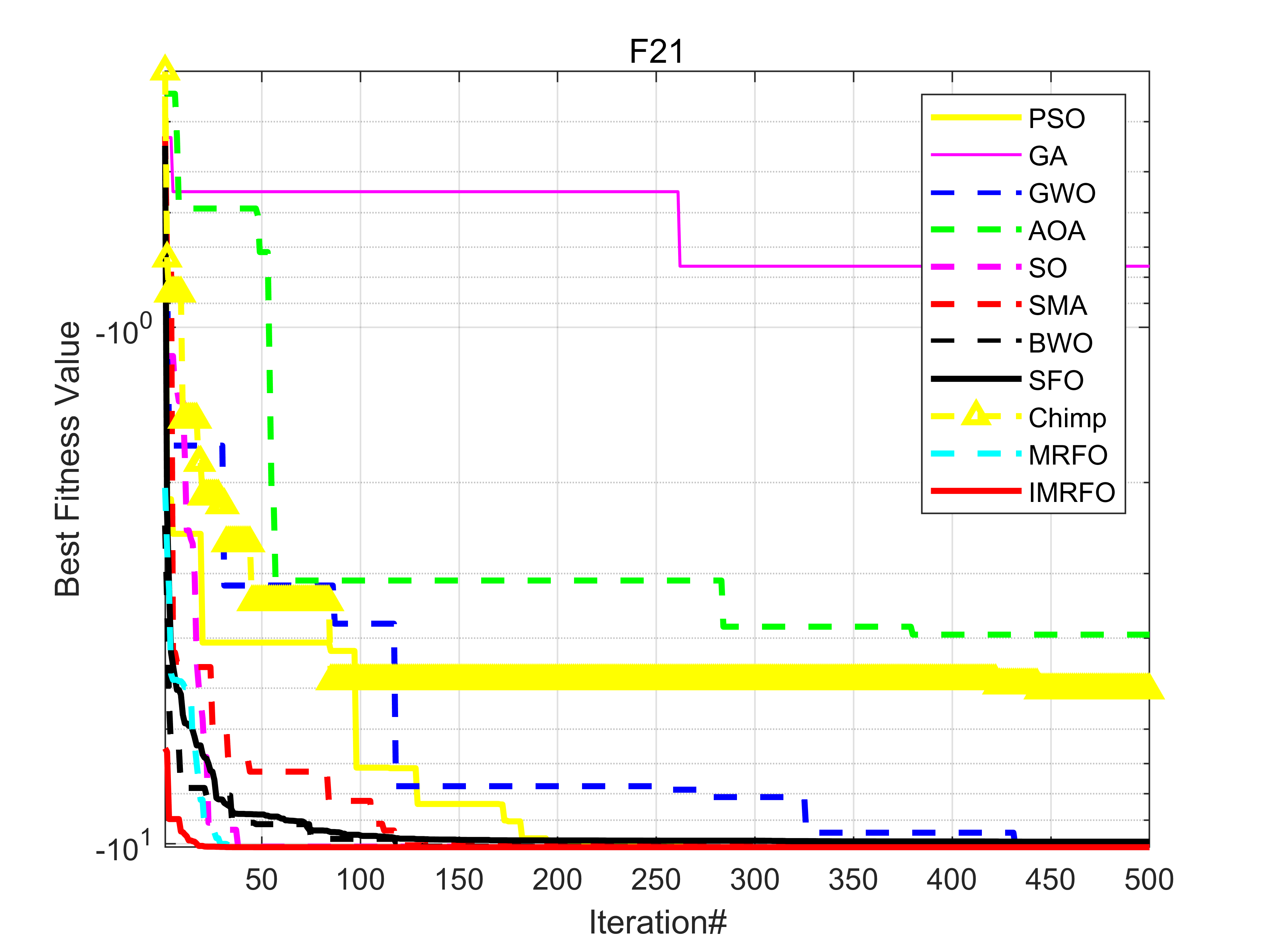

Supplement: Supplementary file 1 — Supplementary Information. [file 41598_2024_59960_MOESM1_ESM.zip › All research figures/All research figures/1 Figures of benchmark functions/Figures of all benchmark functions/12/figure/21-21.tif]

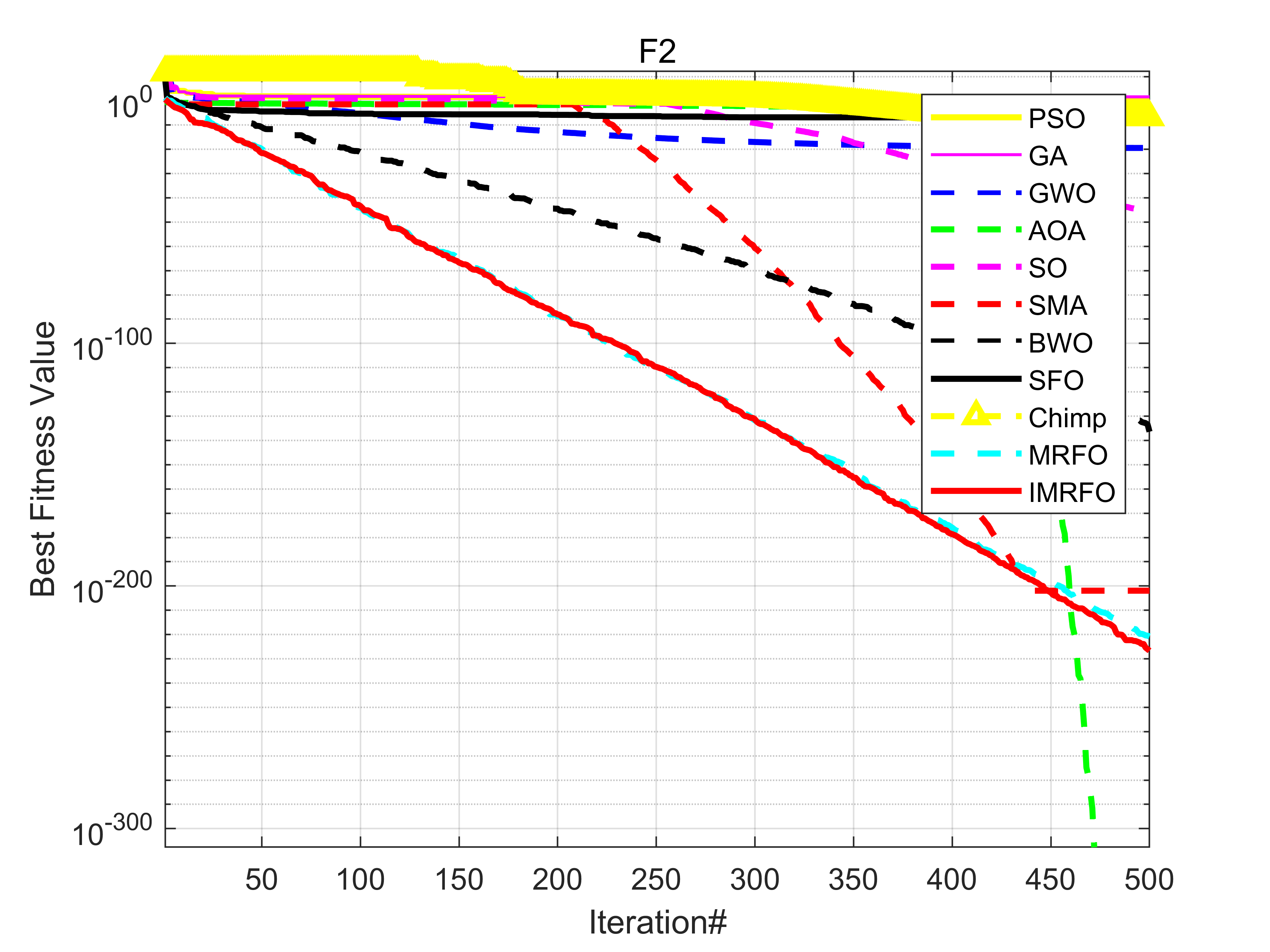

Supplement: Supplementary file 1 — Supplementary Information. [file 41598_2024_59960_MOESM1_ESM.zip › All research figures/All research figures/1 Figures of benchmark functions/Figures of all benchmark functions/12/figure/2-2.tif]

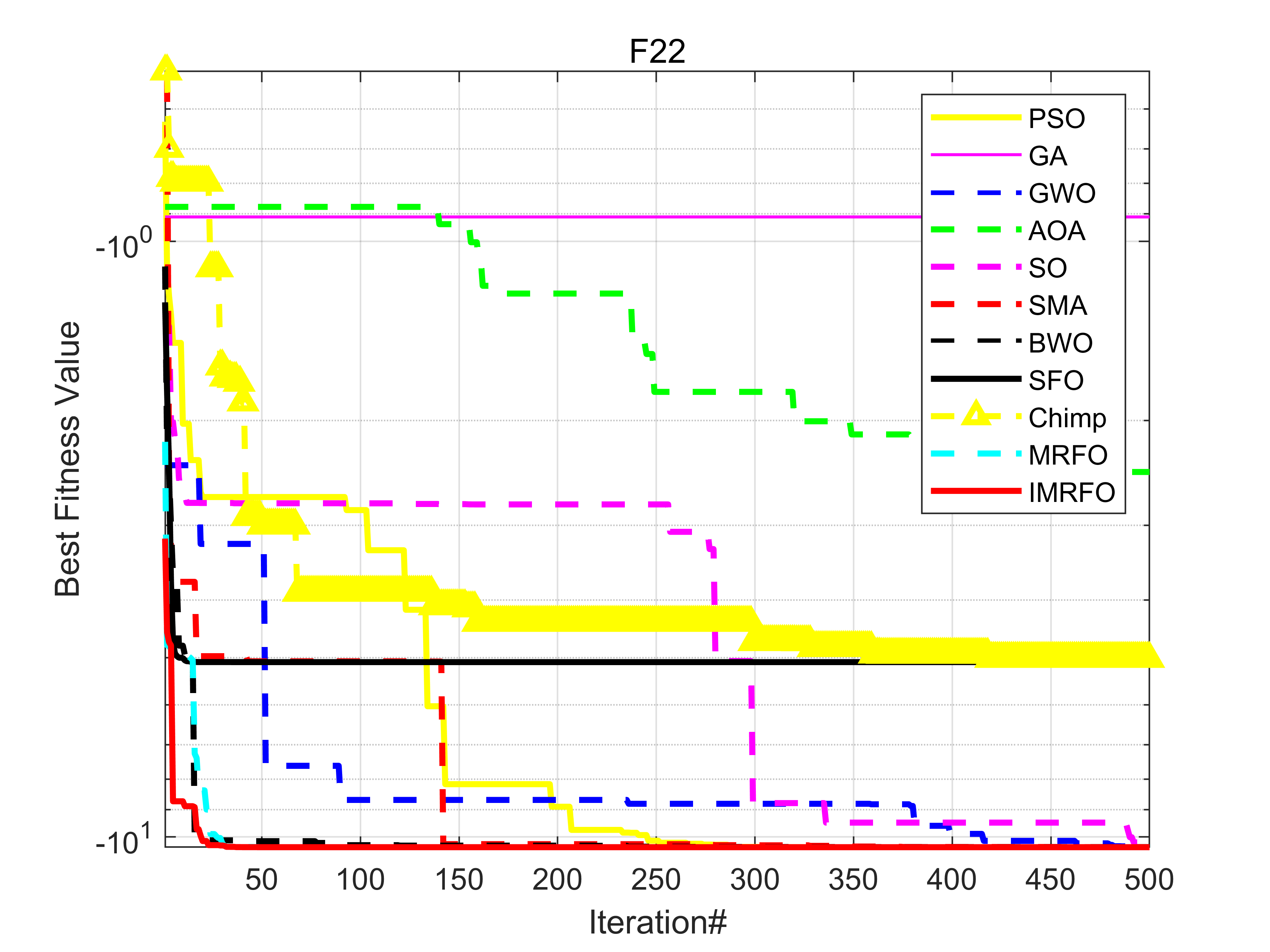

Supplement: Supplementary file 1 — Supplementary Information. [file 41598_2024_59960_MOESM1_ESM.zip › All research figures/All research figures/1 Figures of benchmark functions/Figures of all benchmark functions/12/figure/22-9.tif]

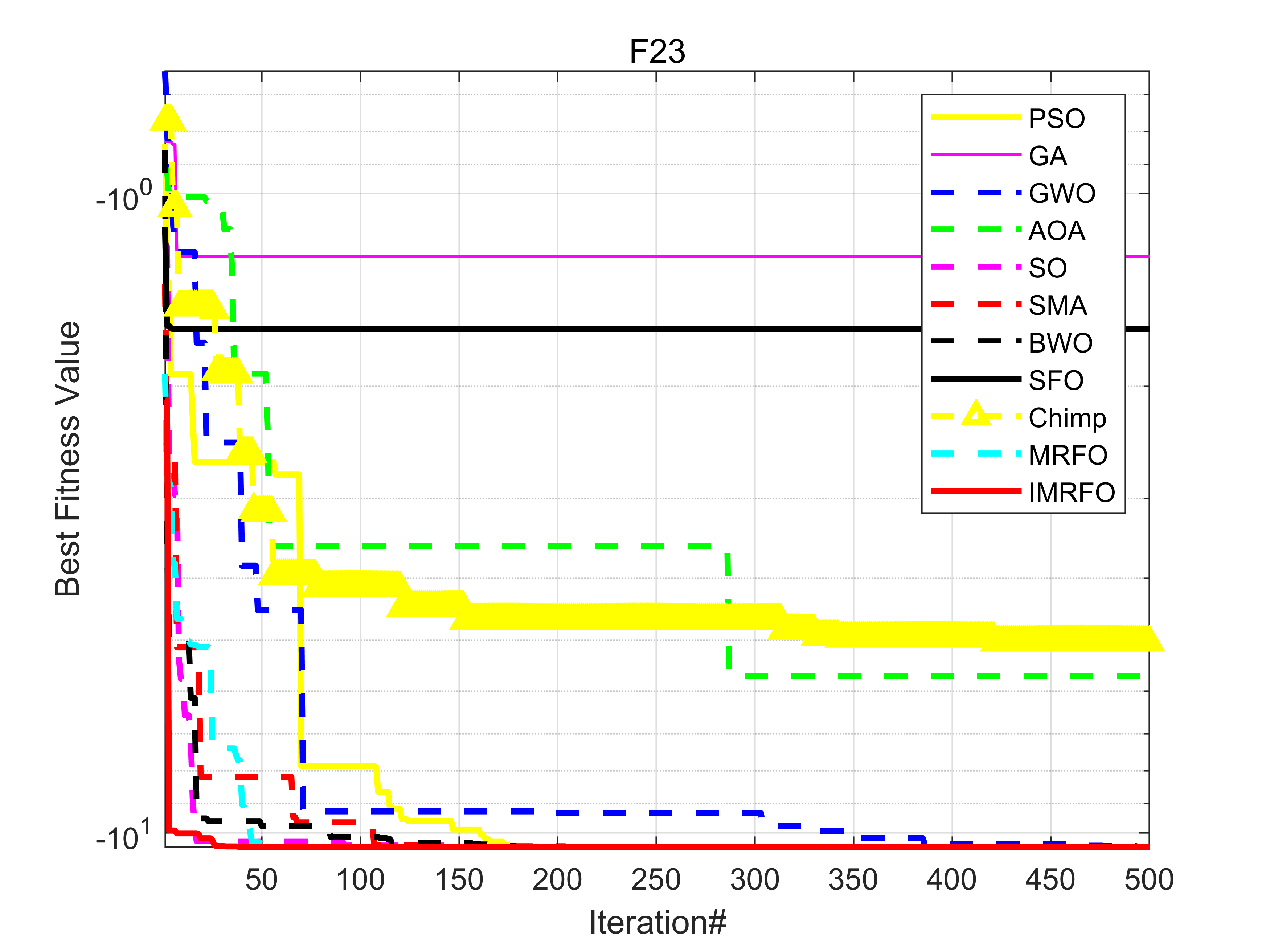

Supplement: Supplementary file 1 — Supplementary Information. [file 41598_2024_59960_MOESM1_ESM.zip › All research figures/All research figures/1 Figures of benchmark functions/Figures of all benchmark functions/12/figure/23-27.tif]

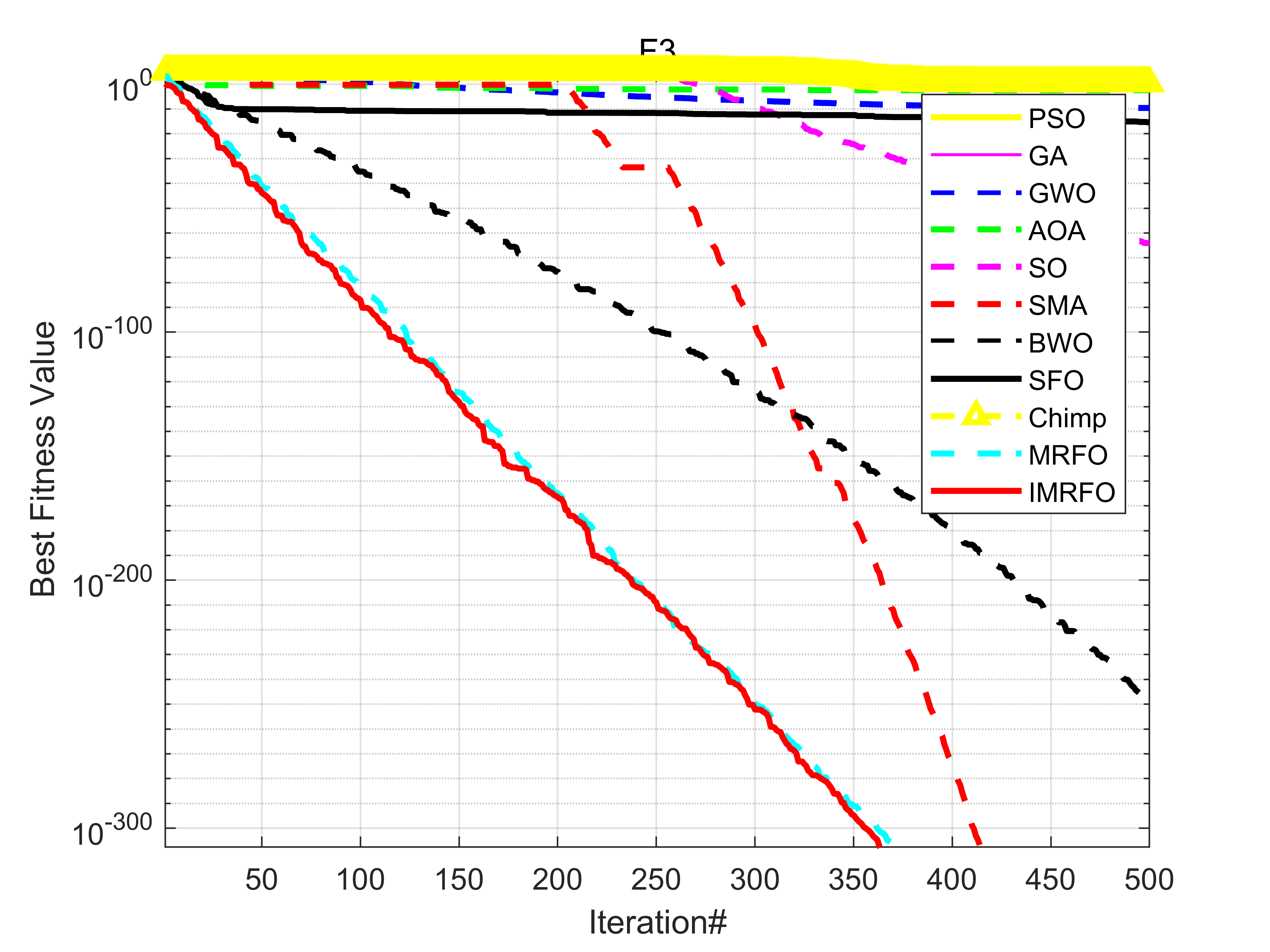

Supplement: Supplementary file 1 — Supplementary Information. [file 41598_2024_59960_MOESM1_ESM.zip › All research figures/All research figures/1 Figures of benchmark functions/Figures of all benchmark functions/12/figure/3-2.tif]

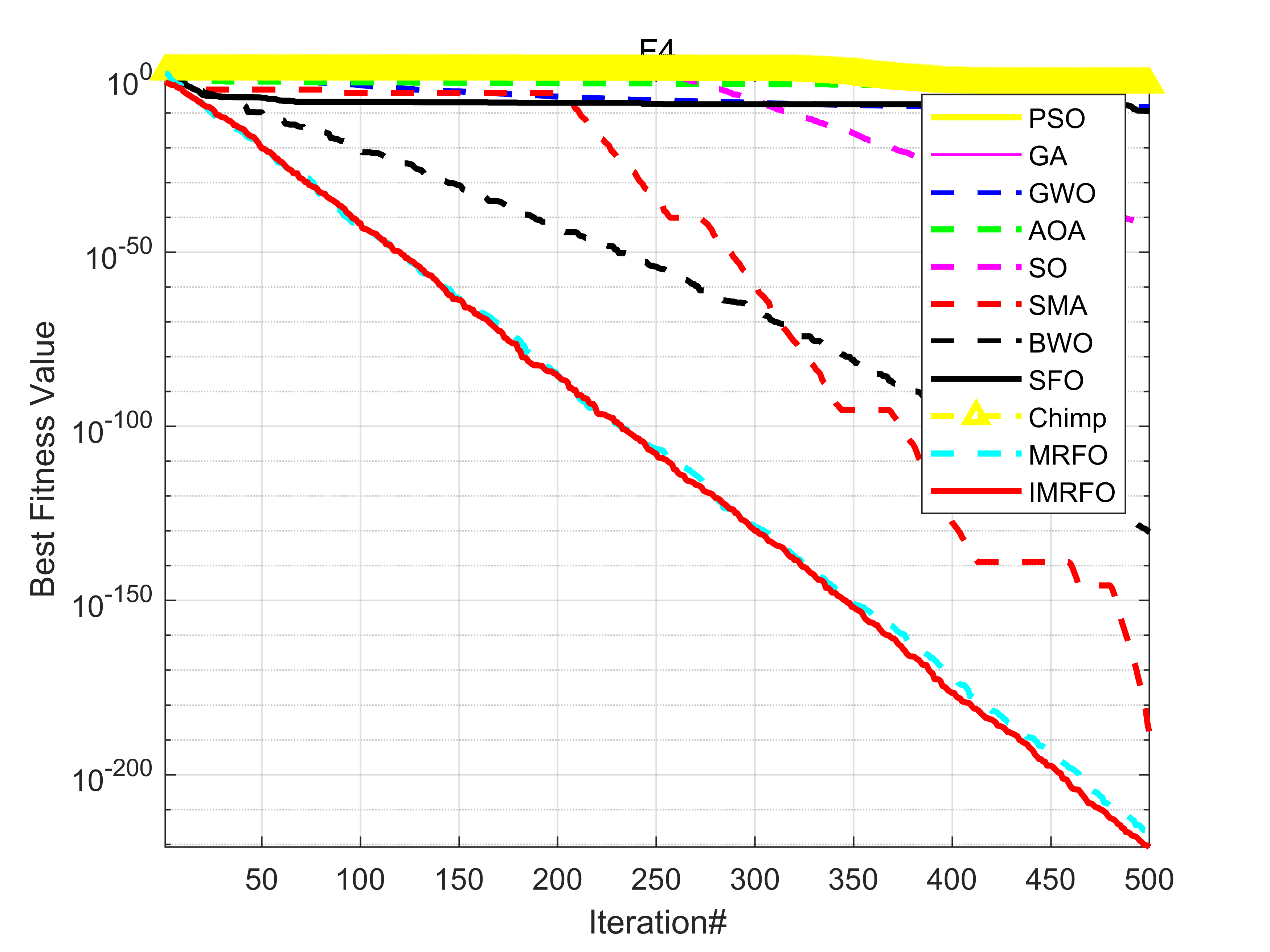

Supplement: Supplementary file 1 — Supplementary Information. [file 41598_2024_59960_MOESM1_ESM.zip › All research figures/All research figures/1 Figures of benchmark functions/Figures of all benchmark functions/12/figure/4-2.tif]

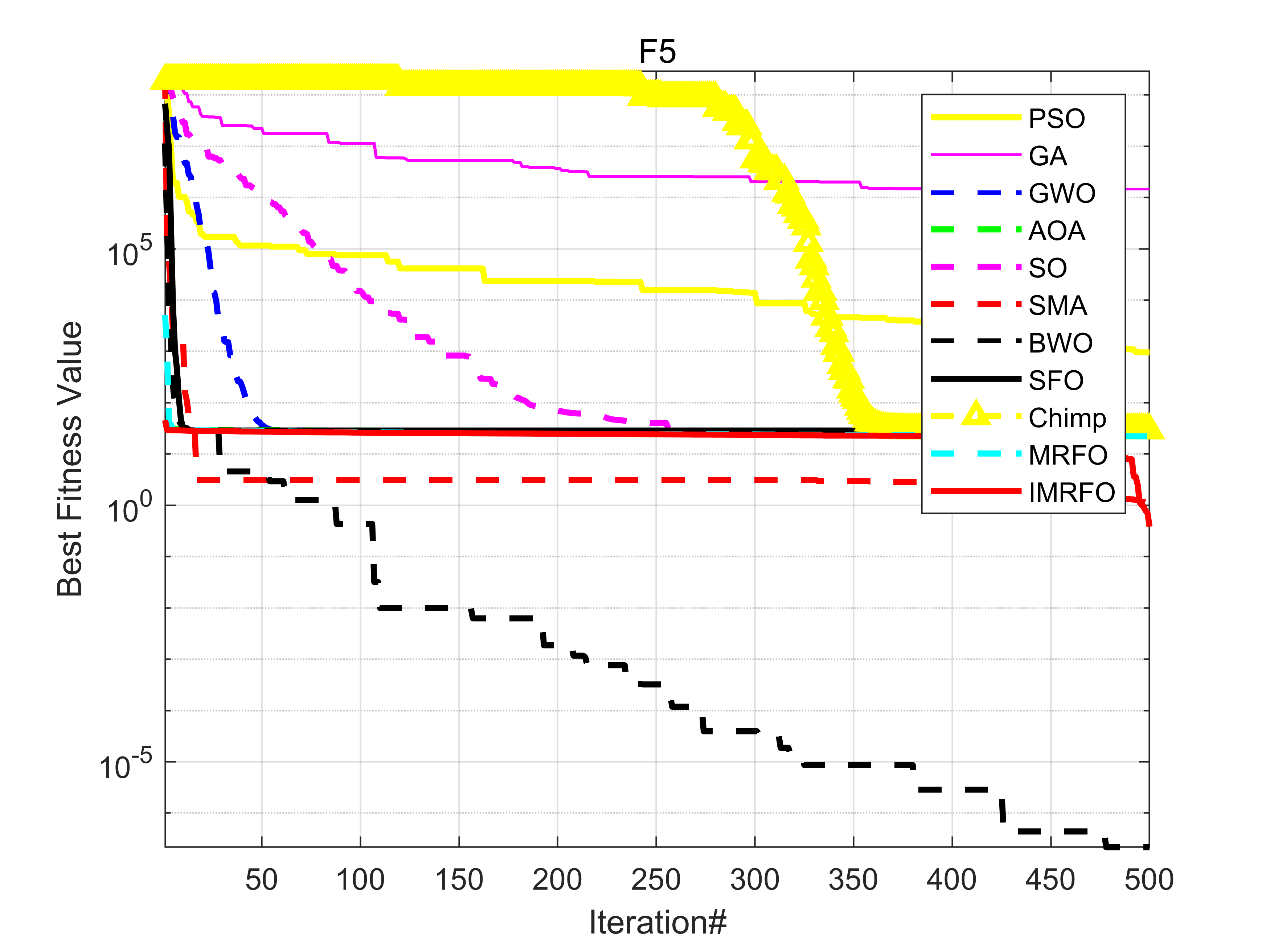

Supplement: Supplementary file 1 — Supplementary Information. [file 41598_2024_59960_MOESM1_ESM.zip › All research figures/All research figures/1 Figures of benchmark functions/Figures of all benchmark functions/12/figure/5-2.tif]

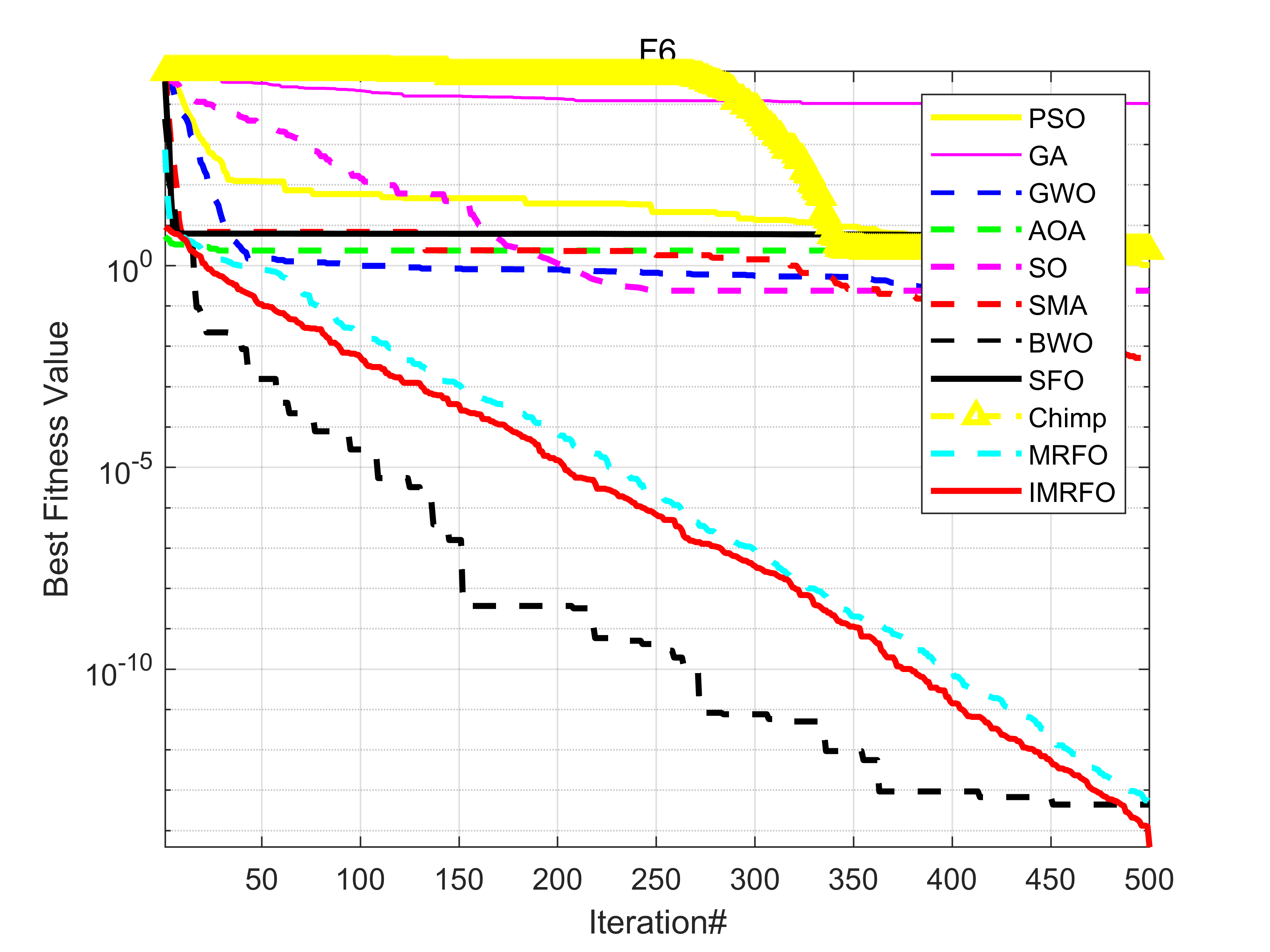

Supplement: Supplementary file 1 — Supplementary Information. [file 41598_2024_59960_MOESM1_ESM.zip › All research figures/All research figures/1 Figures of benchmark functions/Figures of all benchmark functions/12/figure/6-7.tif]

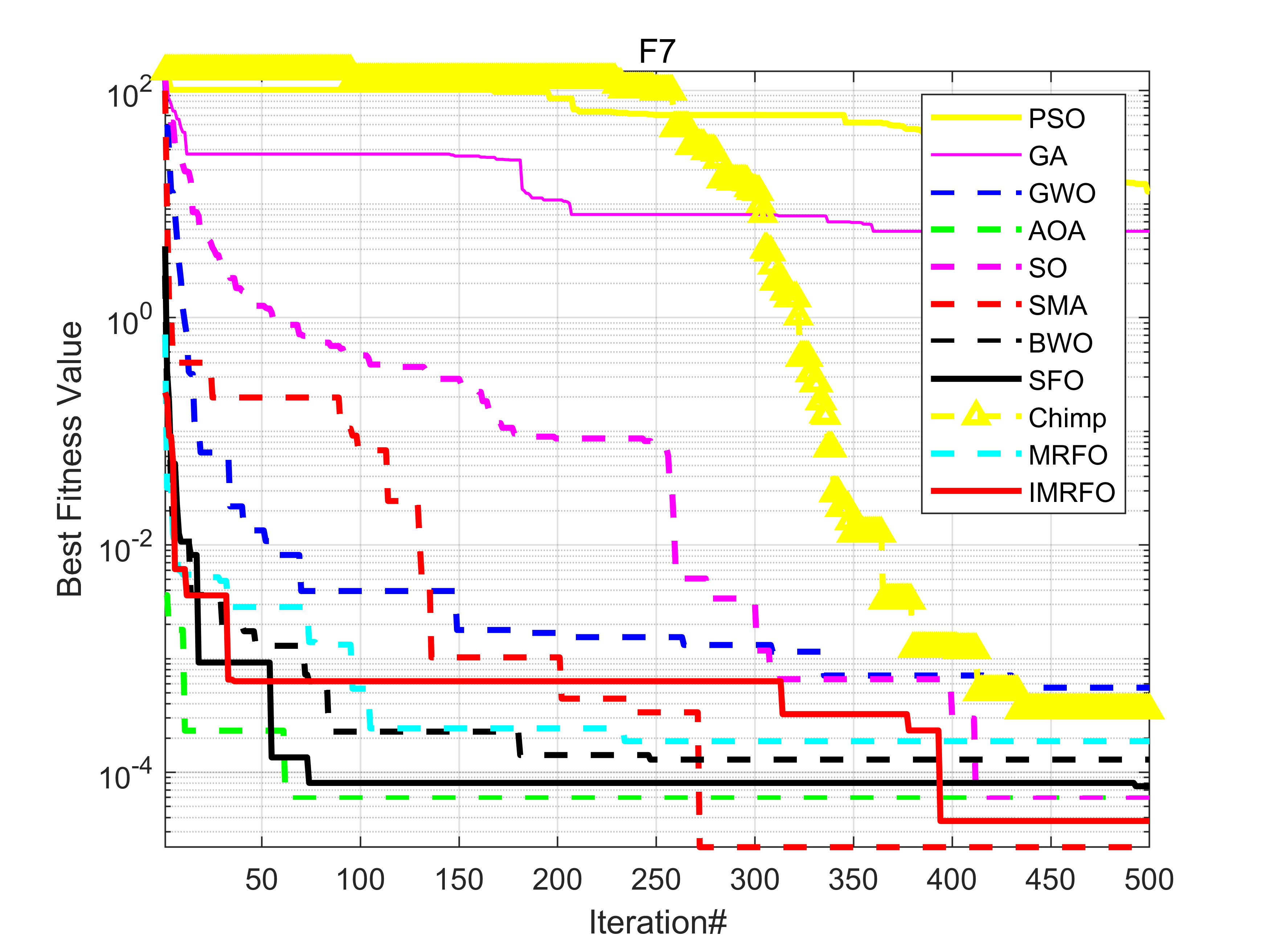

Supplement: Supplementary file 1 — Supplementary Information. [file 41598_2024_59960_MOESM1_ESM.zip › All research figures/All research figures/1 Figures of benchmark functions/Figures of all benchmark functions/12/figure/7-9.tif]

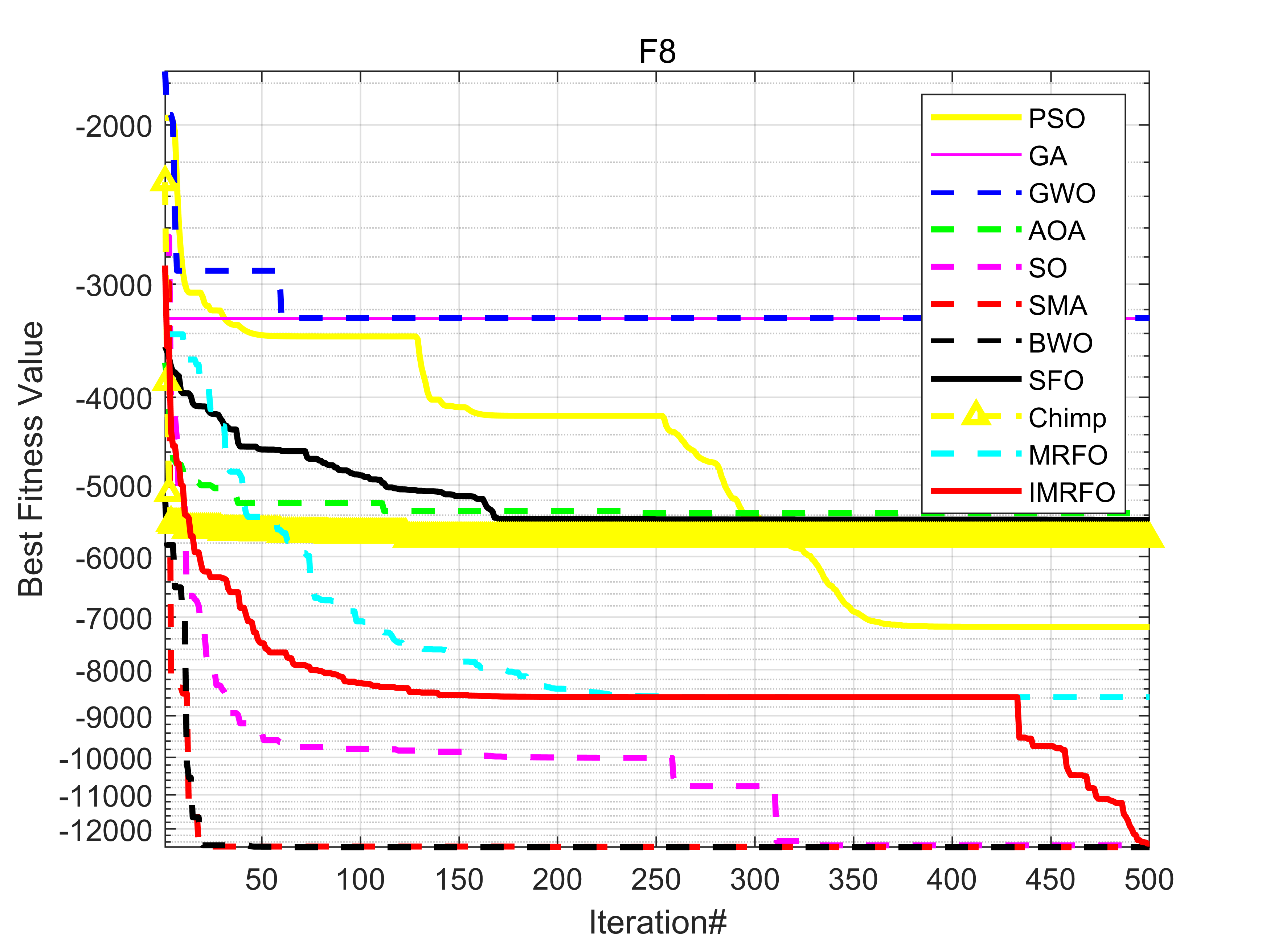

Supplement: Supplementary file 1 — Supplementary Information. [file 41598_2024_59960_MOESM1_ESM.zip › All research figures/All research figures/1 Figures of benchmark functions/Figures of all benchmark functions/12/figure/8-22.tif]

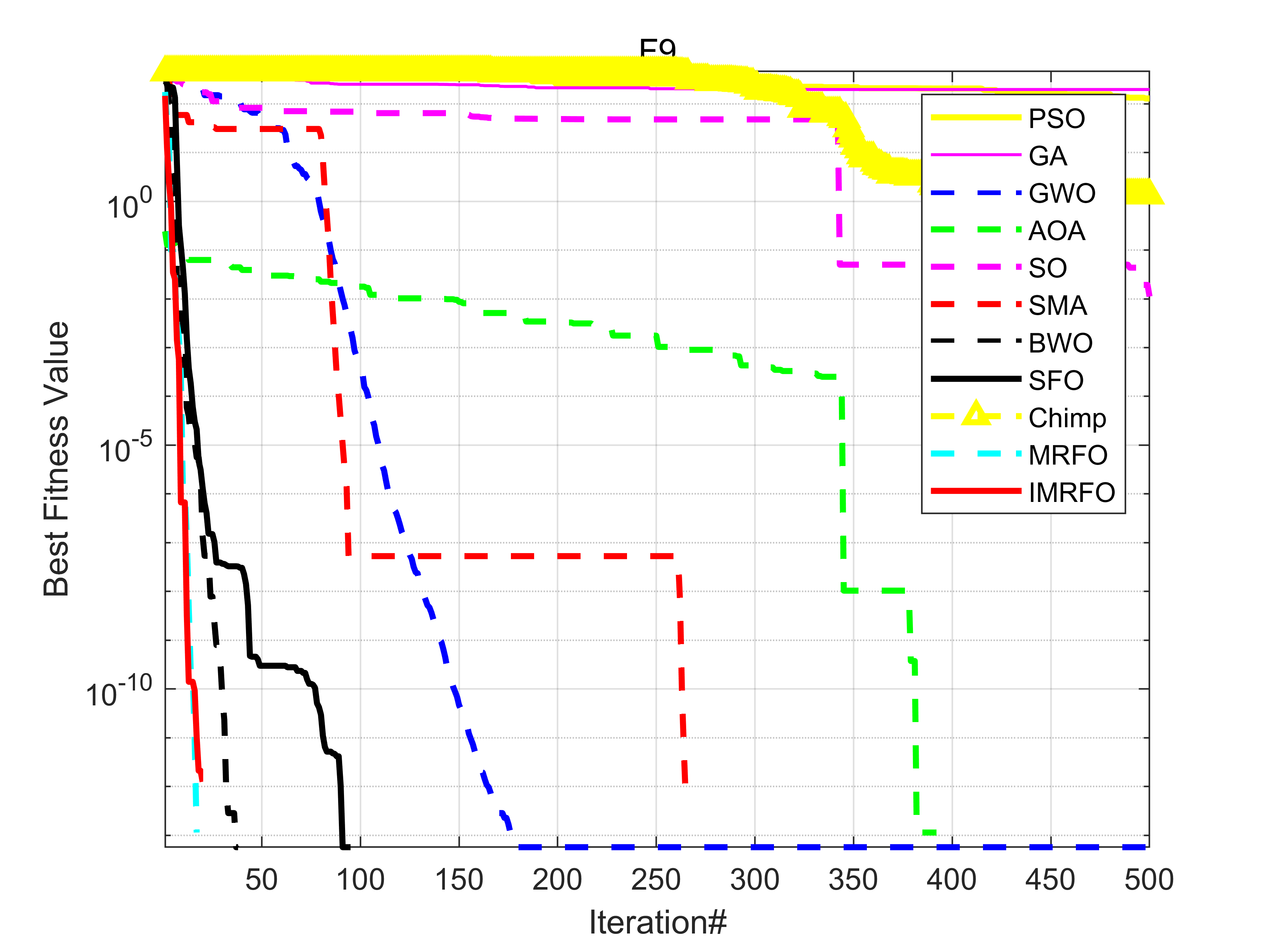

Supplement: Supplementary file 1 — Supplementary Information. [file 41598_2024_59960_MOESM1_ESM.zip › All research figures/All research figures/1 Figures of benchmark functions/Figures of all benchmark functions/12/figure/9-25.tif]

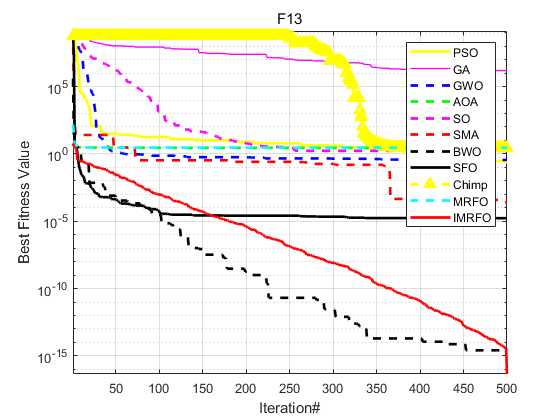

Supplement: Supplementary file 1 — Supplementary Information. [file 41598_2024_59960_MOESM1_ESM.zip › All research figures/All research figures/1 Figures of benchmark functions/Figures of all benchmark functions/13/13-1.tif]

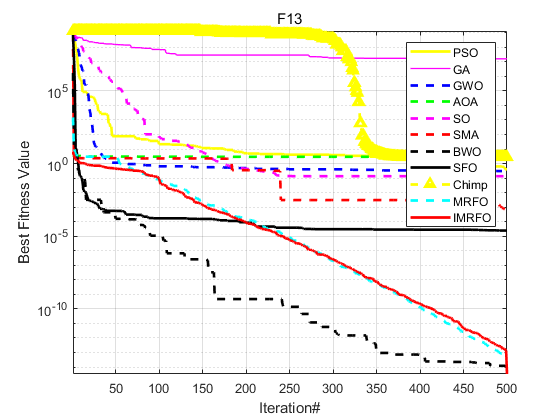

Supplement: Supplementary file 1 — Supplementary Information. [file 41598_2024_59960_MOESM1_ESM.zip › All research figures/All research figures/1 Figures of benchmark functions/Figures of all benchmark functions/13/13-10.tif]

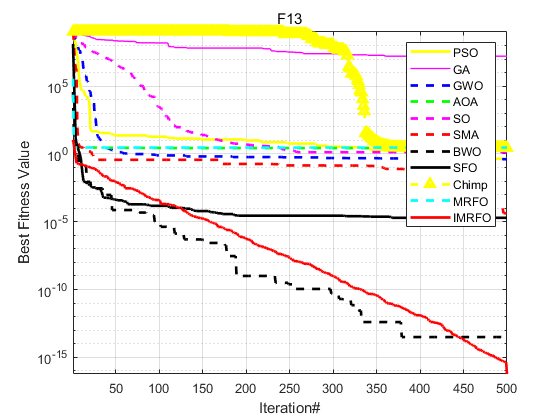

Supplement: Supplementary file 1 — Supplementary Information. [file 41598_2024_59960_MOESM1_ESM.zip › All research figures/All research figures/1 Figures of benchmark functions/Figures of all benchmark functions/13/13-11.tif]

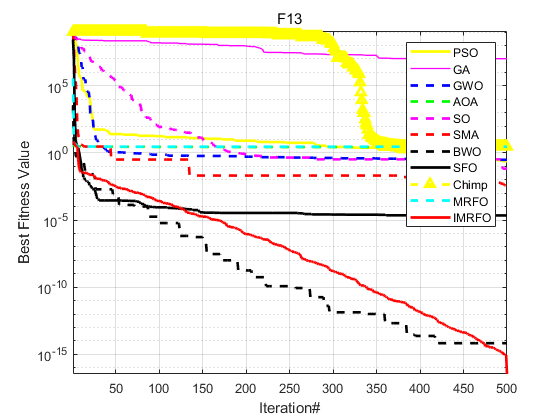

Supplement: Supplementary file 1 — Supplementary Information. [file 41598_2024_59960_MOESM1_ESM.zip › All research figures/All research figures/1 Figures of benchmark functions/Figures of all benchmark functions/13/13-12.tif]

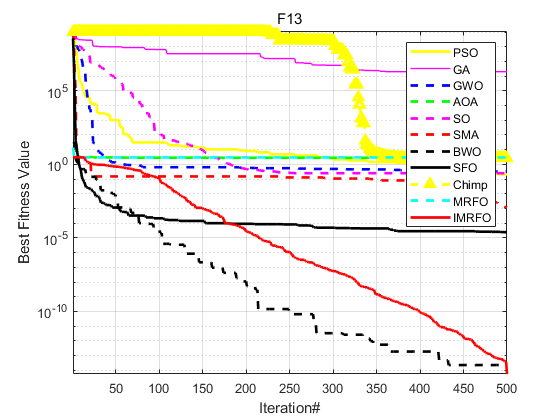

Supplement: Supplementary file 1 — Supplementary Information. [file 41598_2024_59960_MOESM1_ESM.zip › All research figures/All research figures/1 Figures of benchmark functions/Figures of all benchmark functions/13/13-13.tif]

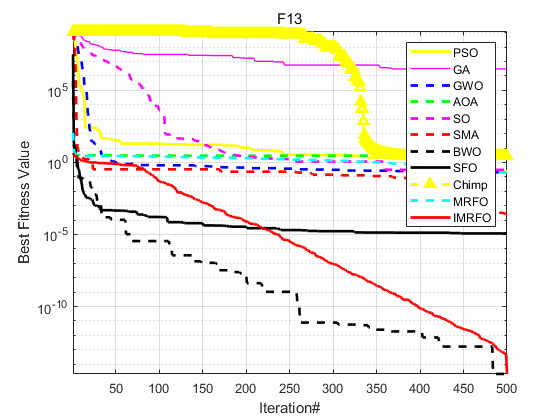

Supplement: Supplementary file 1 — Supplementary Information. [file 41598_2024_59960_MOESM1_ESM.zip › All research figures/All research figures/1 Figures of benchmark functions/Figures of all benchmark functions/13/13-14.tif]

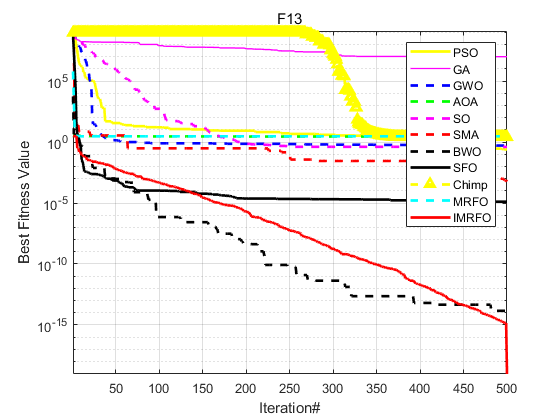

Supplement: Supplementary file 1 — Supplementary Information. [file 41598_2024_59960_MOESM1_ESM.zip › All research figures/All research figures/1 Figures of benchmark functions/Figures of all benchmark functions/13/13-15.tif]

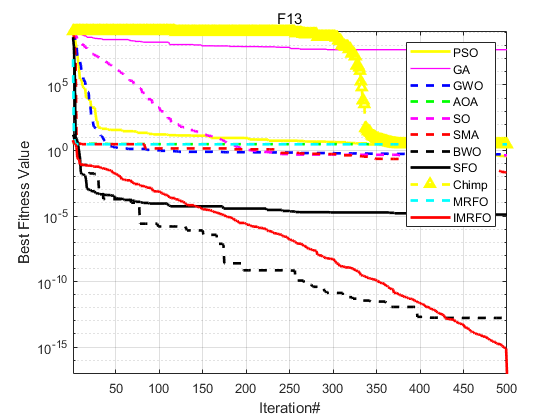

Supplement: Supplementary file 1 — Supplementary Information. [file 41598_2024_59960_MOESM1_ESM.zip › All research figures/All research figures/1 Figures of benchmark functions/Figures of all benchmark functions/13/13-16.tif]

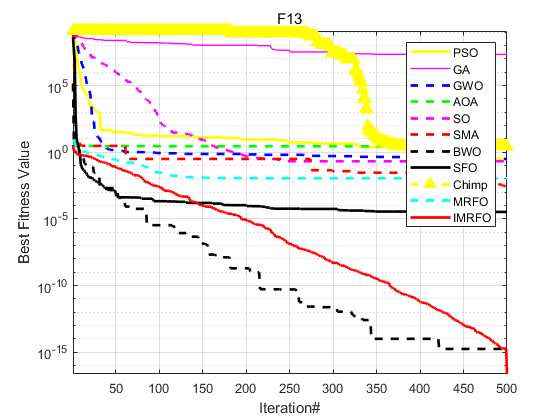

Supplement: Supplementary file 1 — Supplementary Information. [file 41598_2024_59960_MOESM1_ESM.zip › All research figures/All research figures/1 Figures of benchmark functions/Figures of all benchmark functions/13/13-17.tif]

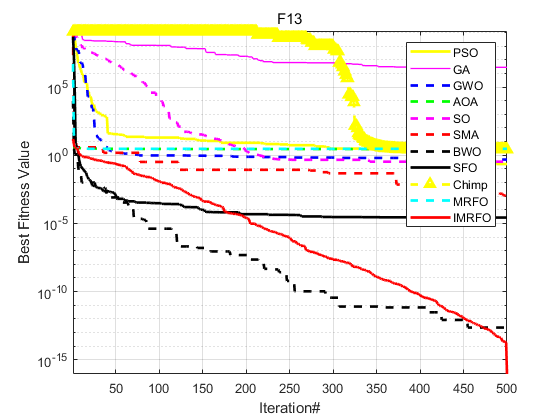

Supplement: Supplementary file 1 — Supplementary Information. [file 41598_2024_59960_MOESM1_ESM.zip › All research figures/All research figures/1 Figures of benchmark functions/Figures of all benchmark functions/13/13-18.tif]

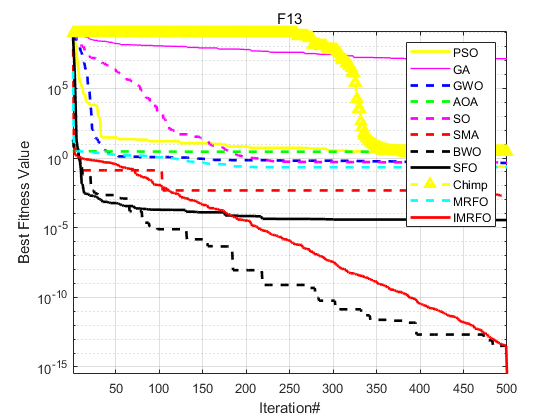

Supplement: Supplementary file 1 — Supplementary Information. [file 41598_2024_59960_MOESM1_ESM.zip › All research figures/All research figures/1 Figures of benchmark functions/Figures of all benchmark functions/13/13-19.tif]
