# Supplementary figures and images for: An improved manta ray foraging optimization algorithm (part 1 of 2)
Source: Sci Rep. 2024 May 5;14:10301. doi: 10.1038/s41598-024-59960-1 (PMC11070432; doi:10.1038/s41598-024-59960-1)

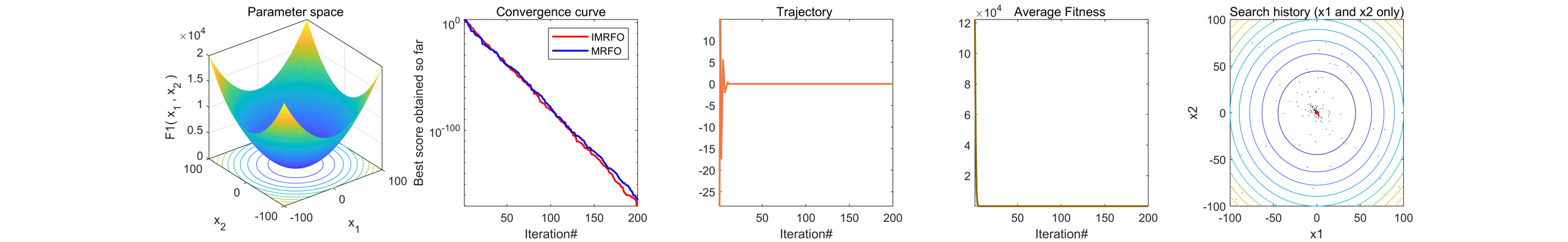

Supplement: Supplementary file 1 — Supplementary Information. [file 41598_2024_59960_MOESM1_ESM.zip › All research figures/All research figures/1 Figures of benchmark functions/Benchmak functions-vision/1.tif]

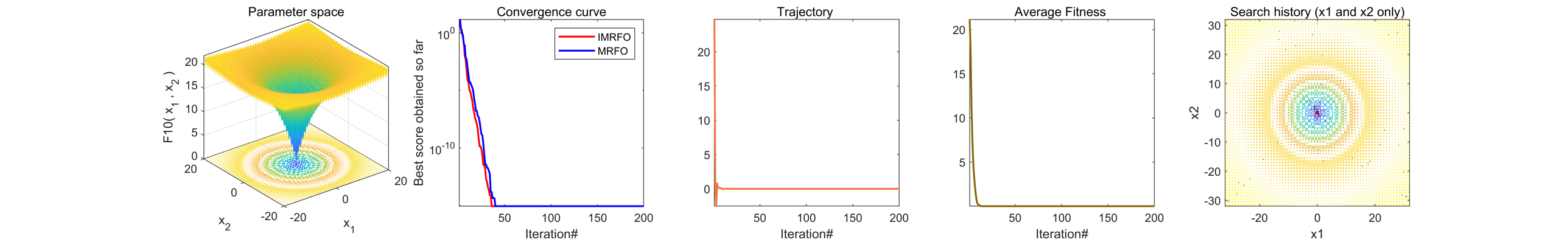

Supplement: Supplementary file 1 — Supplementary Information. [file 41598_2024_59960_MOESM1_ESM.zip › All research figures/All research figures/1 Figures of benchmark functions/Benchmak functions-vision/10.tif]

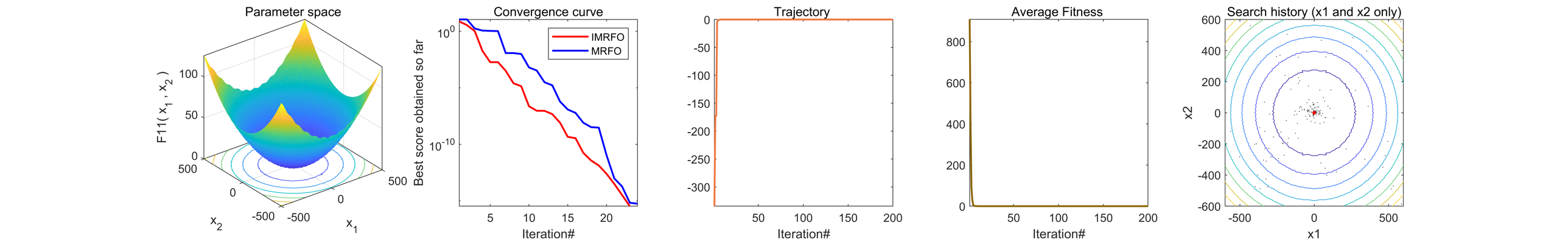

Supplement: Supplementary file 1 — Supplementary Information. [file 41598_2024_59960_MOESM1_ESM.zip › All research figures/All research figures/1 Figures of benchmark functions/Benchmak functions-vision/11.tif]

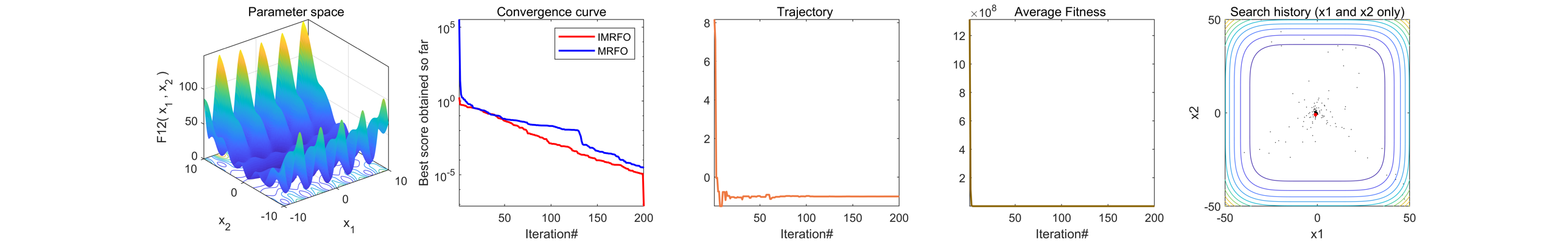

Supplement: Supplementary file 1 — Supplementary Information. [file 41598_2024_59960_MOESM1_ESM.zip › All research figures/All research figures/1 Figures of benchmark functions/Benchmak functions-vision/12.tif]

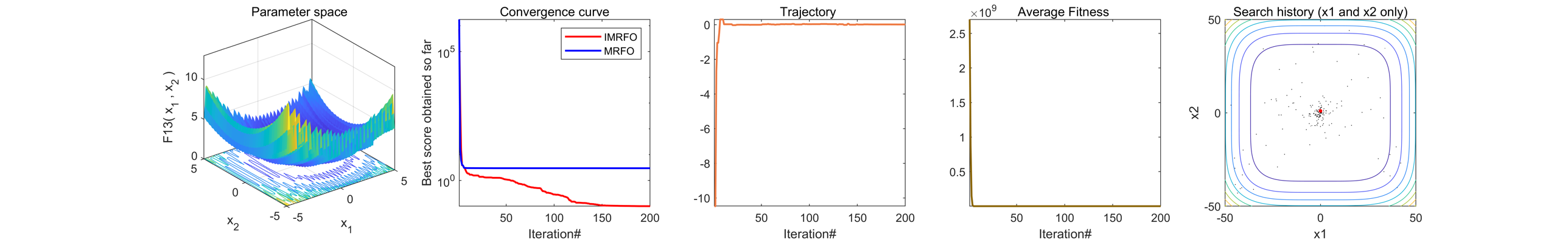

Supplement: Supplementary file 1 — Supplementary Information. [file 41598_2024_59960_MOESM1_ESM.zip › All research figures/All research figures/1 Figures of benchmark functions/Benchmak functions-vision/13.tif]

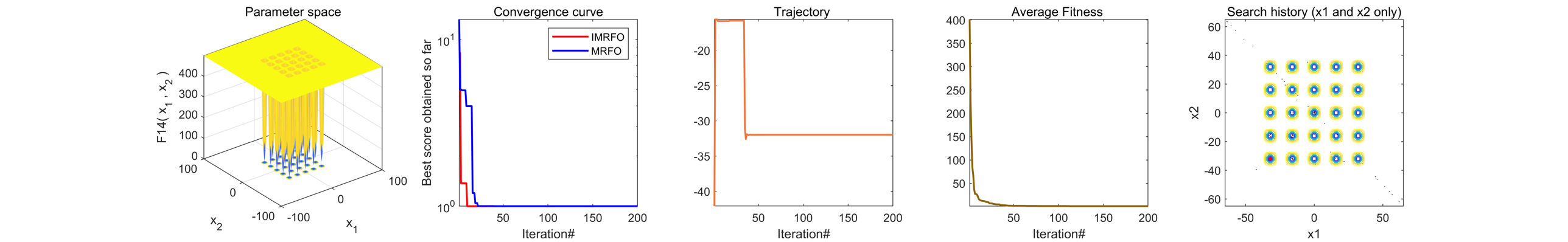

Supplement: Supplementary file 1 — Supplementary Information. [file 41598_2024_59960_MOESM1_ESM.zip › All research figures/All research figures/1 Figures of benchmark functions/Benchmak functions-vision/14.tif]

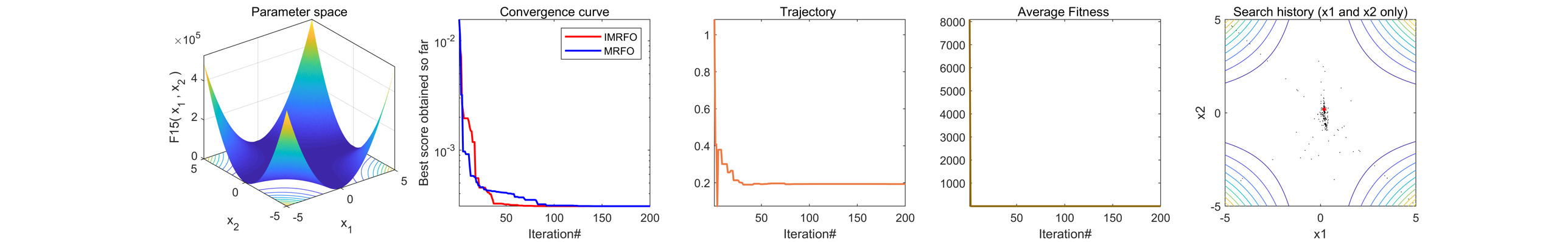

Supplement: Supplementary file 1 — Supplementary Information. [file 41598_2024_59960_MOESM1_ESM.zip › All research figures/All research figures/1 Figures of benchmark functions/Benchmak functions-vision/15.tif]

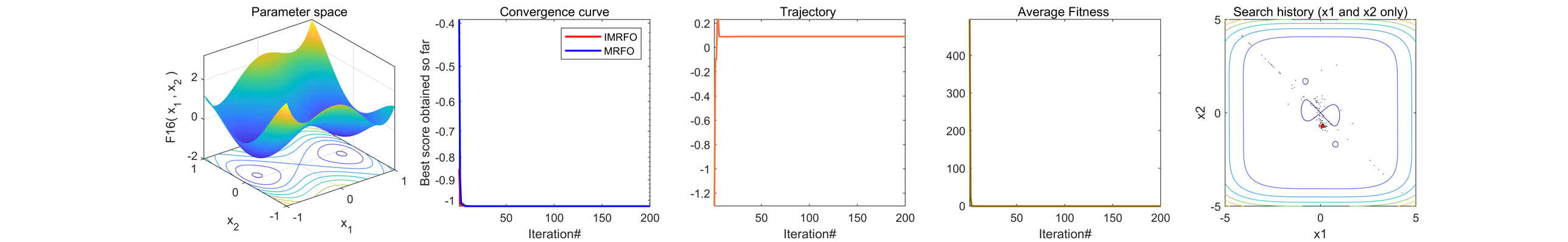

Supplement: Supplementary file 1 — Supplementary Information. [file 41598_2024_59960_MOESM1_ESM.zip › All research figures/All research figures/1 Figures of benchmark functions/Benchmak functions-vision/16.tif]

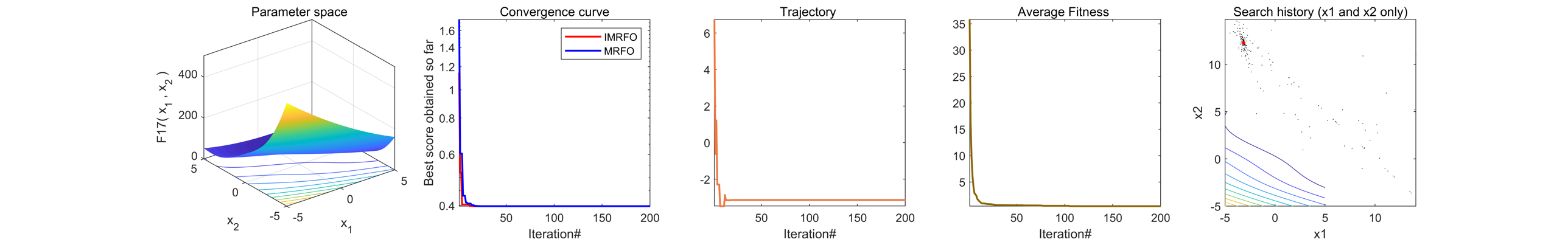

Supplement: Supplementary file 1 — Supplementary Information. [file 41598_2024_59960_MOESM1_ESM.zip › All research figures/All research figures/1 Figures of benchmark functions/Benchmak functions-vision/17.tif]

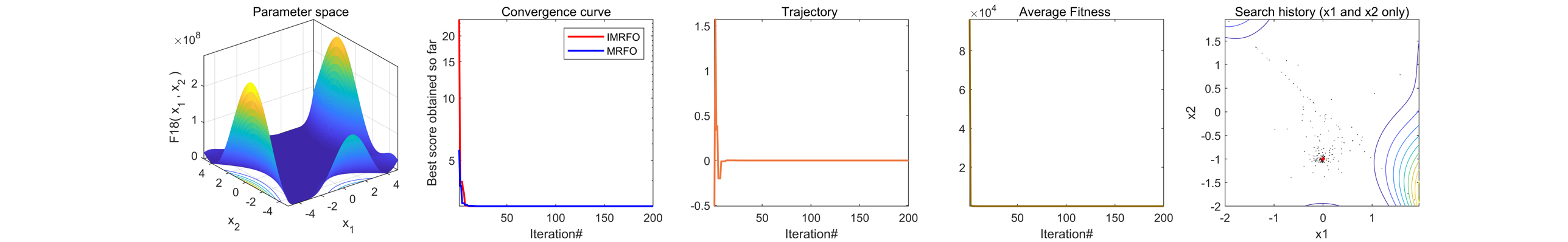

Supplement: Supplementary file 1 — Supplementary Information. [file 41598_2024_59960_MOESM1_ESM.zip › All research figures/All research figures/1 Figures of benchmark functions/Benchmak functions-vision/18.tif]

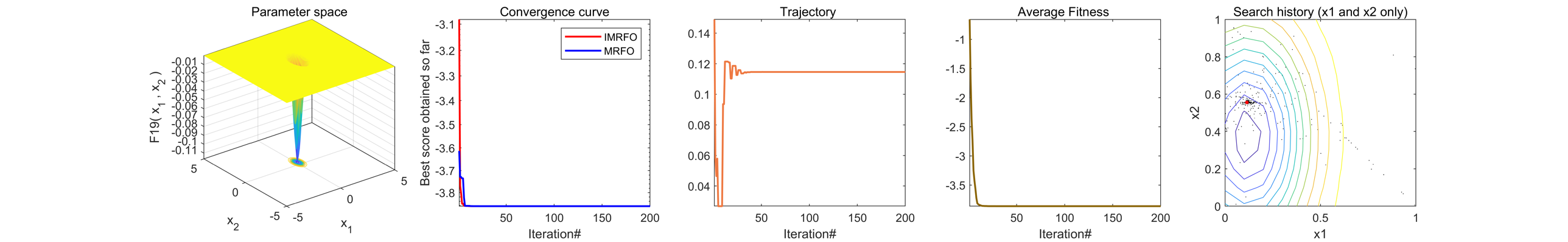

Supplement: Supplementary file 1 — Supplementary Information. [file 41598_2024_59960_MOESM1_ESM.zip › All research figures/All research figures/1 Figures of benchmark functions/Benchmak functions-vision/19.tif]

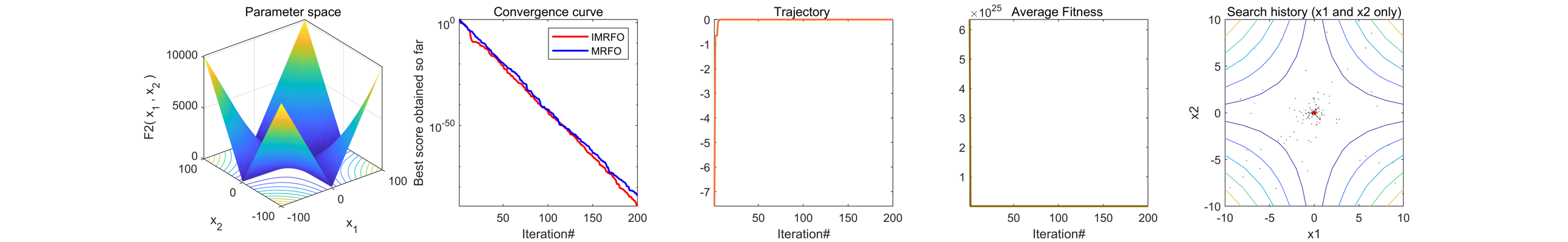

Supplement: Supplementary file 1 — Supplementary Information. [file 41598_2024_59960_MOESM1_ESM.zip › All research figures/All research figures/1 Figures of benchmark functions/Benchmak functions-vision/2.tif]

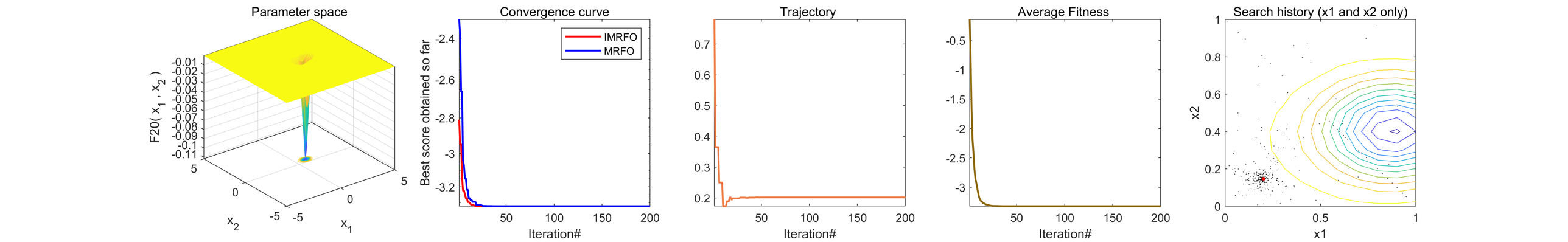

Supplement: Supplementary file 1 — Supplementary Information. [file 41598_2024_59960_MOESM1_ESM.zip › All research figures/All research figures/1 Figures of benchmark functions/Benchmak functions-vision/20.tif]

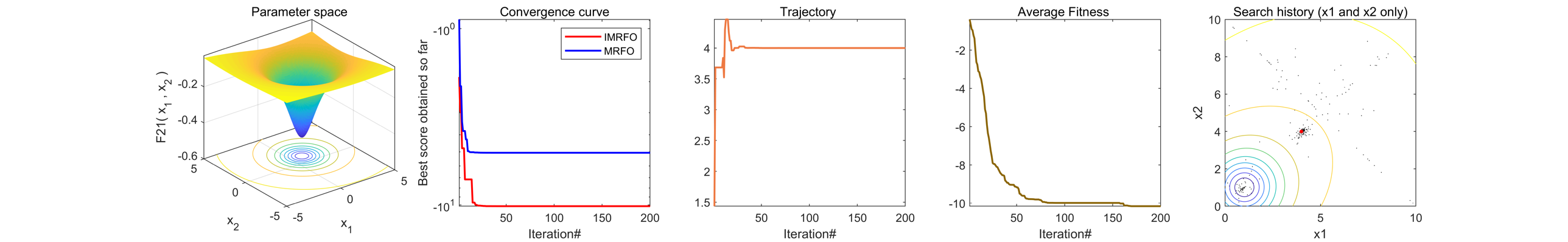

Supplement: Supplementary file 1 — Supplementary Information. [file 41598_2024_59960_MOESM1_ESM.zip › All research figures/All research figures/1 Figures of benchmark functions/Benchmak functions-vision/21.tif]

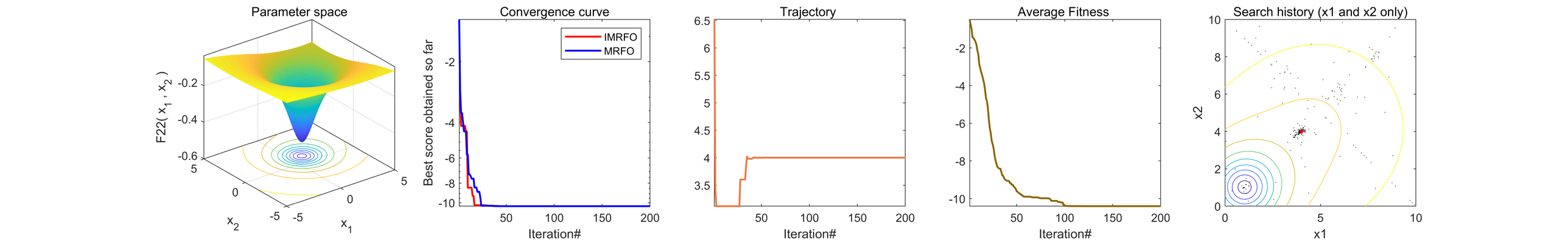

Supplement: Supplementary file 1 — Supplementary Information. [file 41598_2024_59960_MOESM1_ESM.zip › All research figures/All research figures/1 Figures of benchmark functions/Benchmak functions-vision/22.tif]

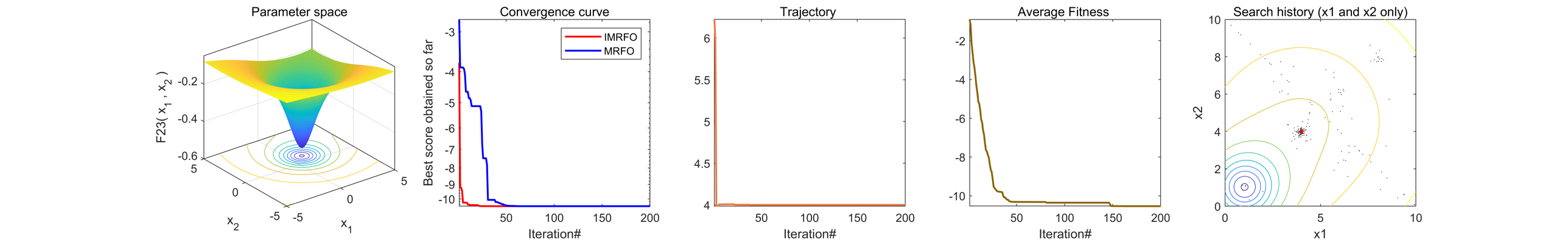

Supplement: Supplementary file 1 — Supplementary Information. [file 41598_2024_59960_MOESM1_ESM.zip › All research figures/All research figures/1 Figures of benchmark functions/Benchmak functions-vision/23.tif]

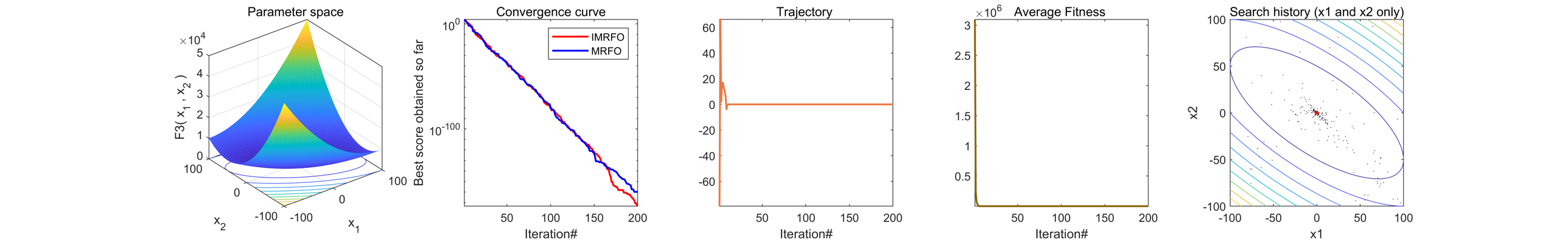

Supplement: Supplementary file 1 — Supplementary Information. [file 41598_2024_59960_MOESM1_ESM.zip › All research figures/All research figures/1 Figures of benchmark functions/Benchmak functions-vision/3.tif]

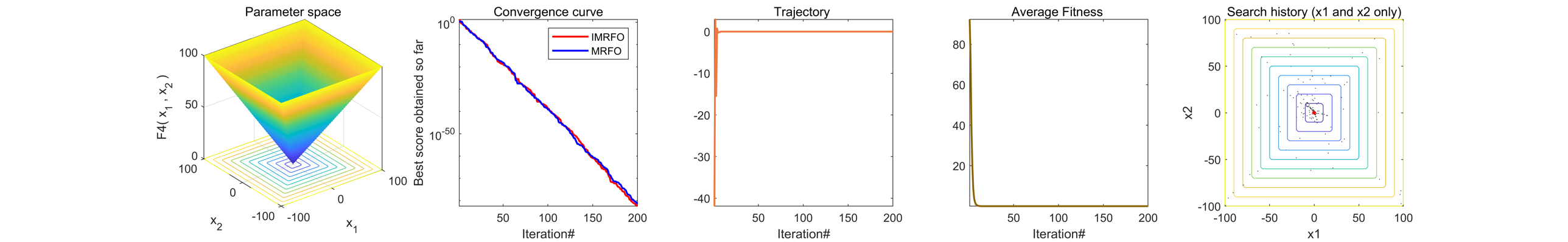

Supplement: Supplementary file 1 — Supplementary Information. [file 41598_2024_59960_MOESM1_ESM.zip › All research figures/All research figures/1 Figures of benchmark functions/Benchmak functions-vision/4.tif]

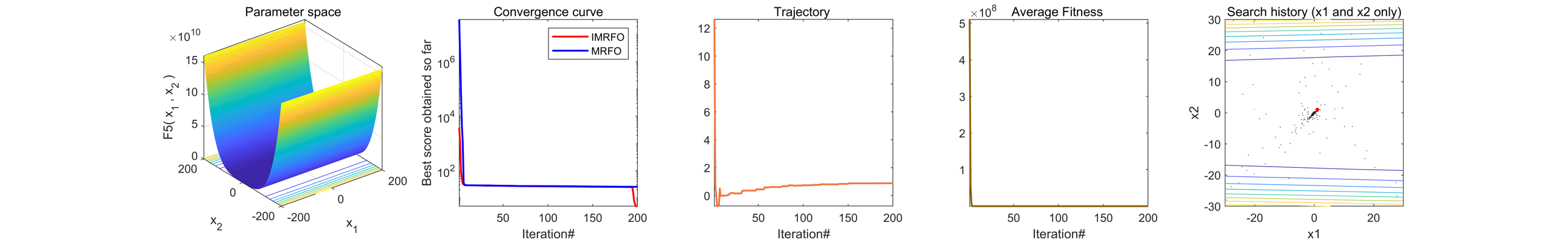

Supplement: Supplementary file 1 — Supplementary Information. [file 41598_2024_59960_MOESM1_ESM.zip › All research figures/All research figures/1 Figures of benchmark functions/Benchmak functions-vision/5.tif]

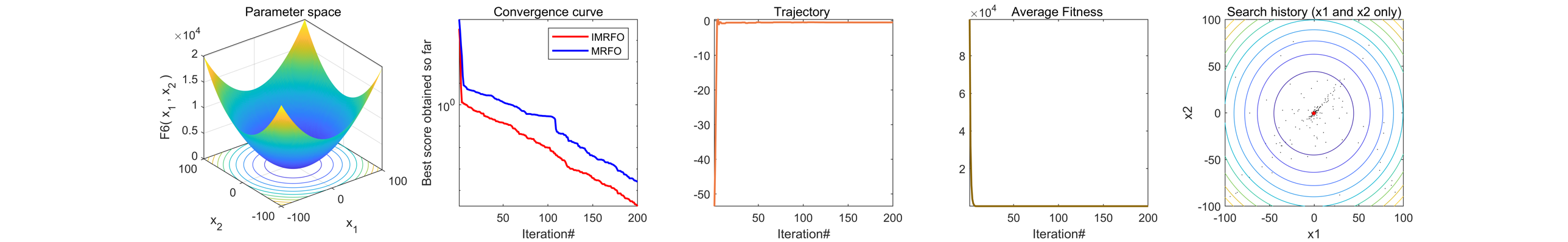

Supplement: Supplementary file 1 — Supplementary Information. [file 41598_2024_59960_MOESM1_ESM.zip › All research figures/All research figures/1 Figures of benchmark functions/Benchmak functions-vision/6.tif]

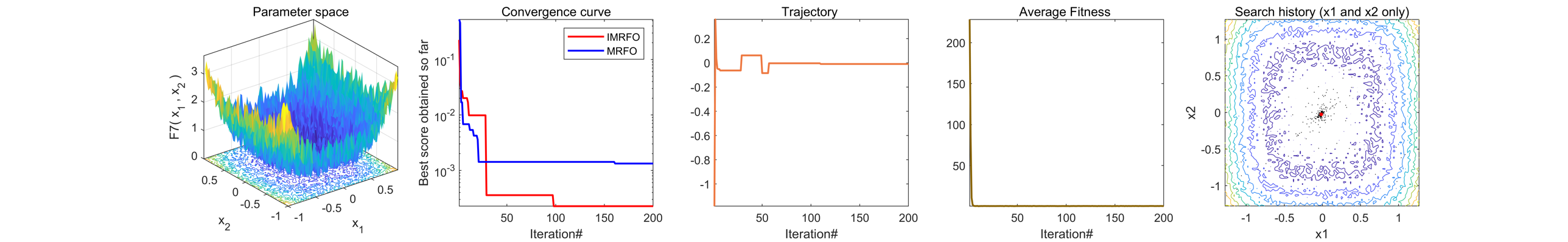

Supplement: Supplementary file 1 — Supplementary Information. [file 41598_2024_59960_MOESM1_ESM.zip › All research figures/All research figures/1 Figures of benchmark functions/Benchmak functions-vision/7.tif]

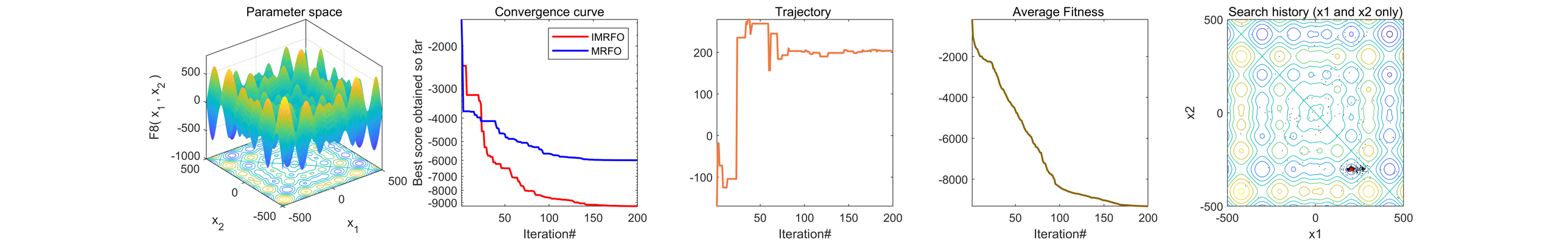

Supplement: Supplementary file 1 — Supplementary Information. [file 41598_2024_59960_MOESM1_ESM.zip › All research figures/All research figures/1 Figures of benchmark functions/Benchmak functions-vision/8.tif]

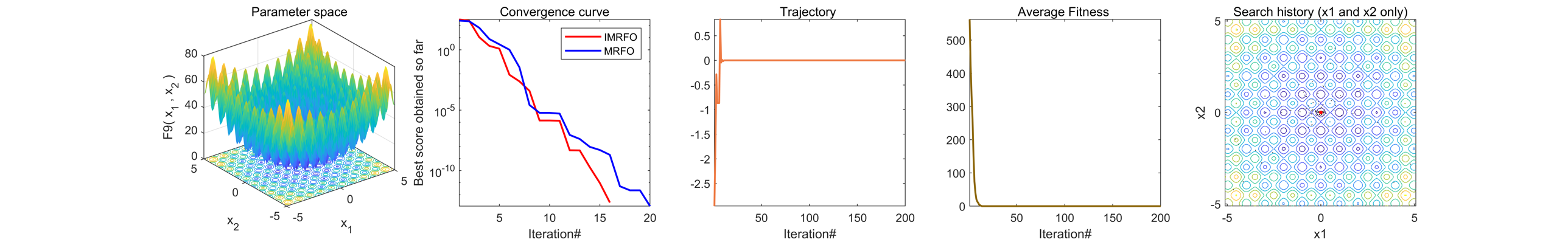

Supplement: Supplementary file 1 — Supplementary Information. [file 41598_2024_59960_MOESM1_ESM.zip › All research figures/All research figures/1 Figures of benchmark functions/Benchmak functions-vision/9.tif]

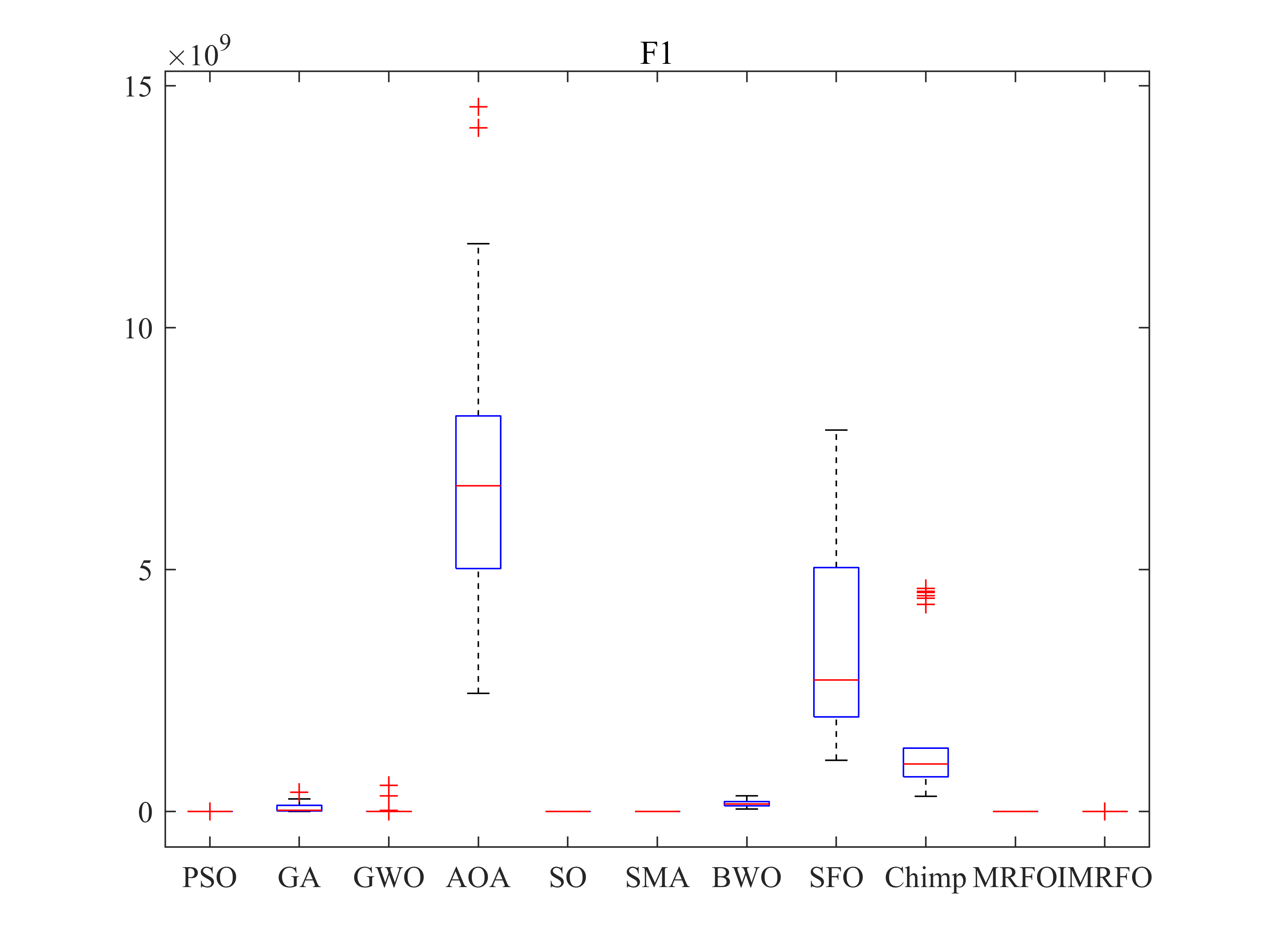

Supplement: Supplementary file 1 — Supplementary Information. [file 41598_2024_59960_MOESM1_ESM.zip › All research figures/All research figures/1 Figures of benchmark functions/Benchmark-functions-box-figure/1.tif]

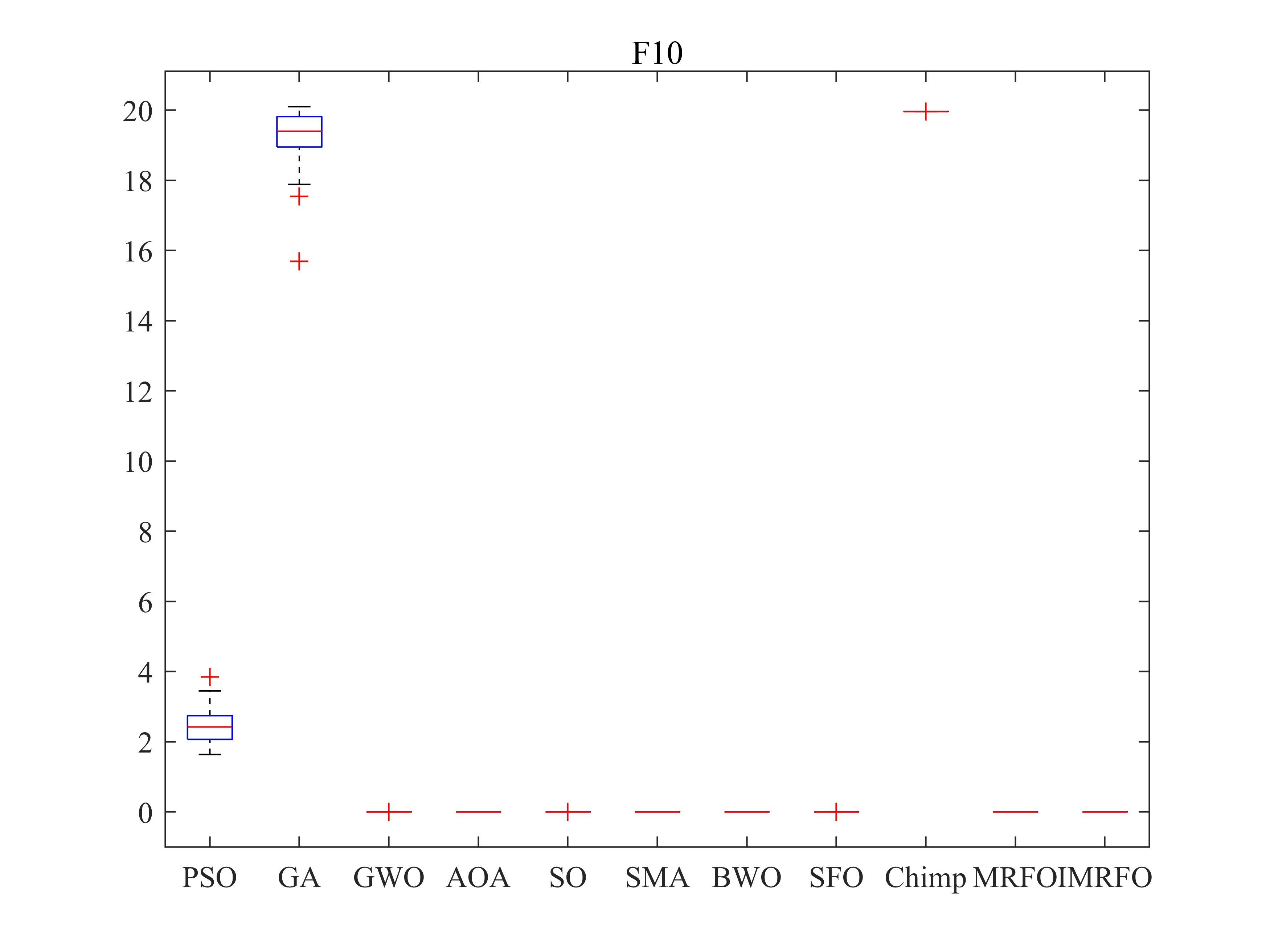

Supplement: Supplementary file 1 — Supplementary Information. [file 41598_2024_59960_MOESM1_ESM.zip › All research figures/All research figures/1 Figures of benchmark functions/Benchmark-functions-box-figure/10.tif]

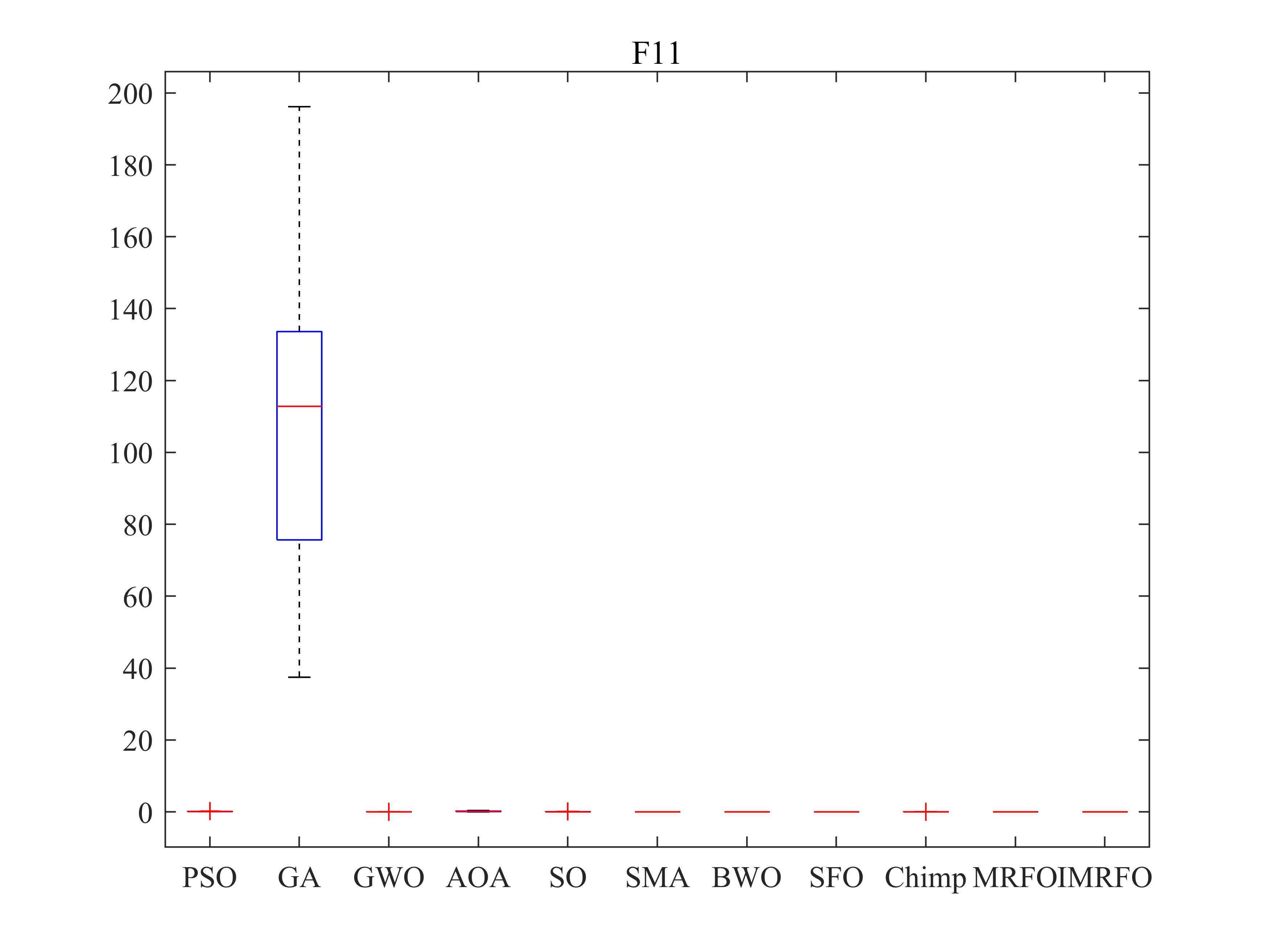

Supplement: Supplementary file 1 — Supplementary Information. [file 41598_2024_59960_MOESM1_ESM.zip › All research figures/All research figures/1 Figures of benchmark functions/Benchmark-functions-box-figure/11.tif]

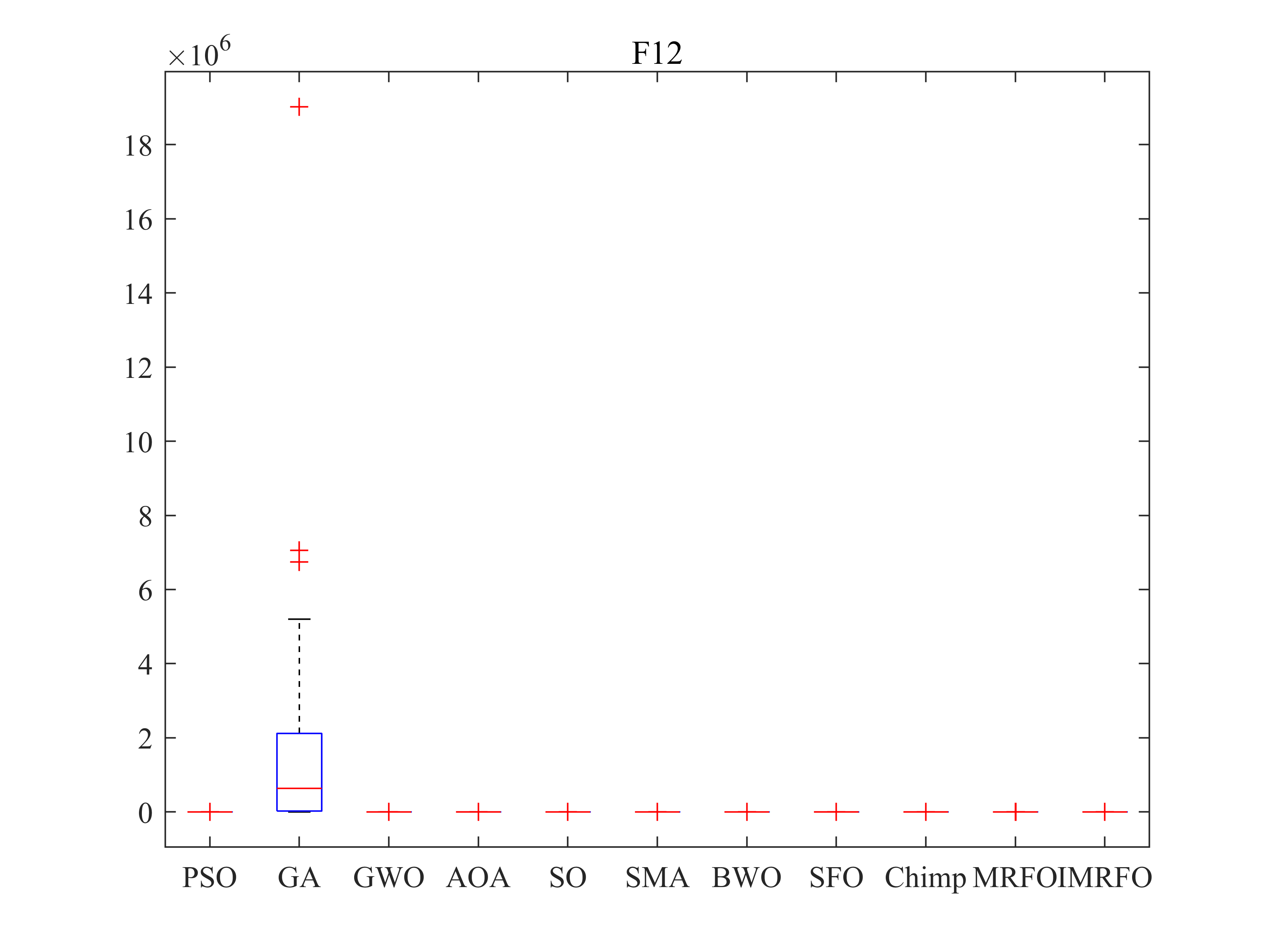

Supplement: Supplementary file 1 — Supplementary Information. [file 41598_2024_59960_MOESM1_ESM.zip › All research figures/All research figures/1 Figures of benchmark functions/Benchmark-functions-box-figure/12.tif]

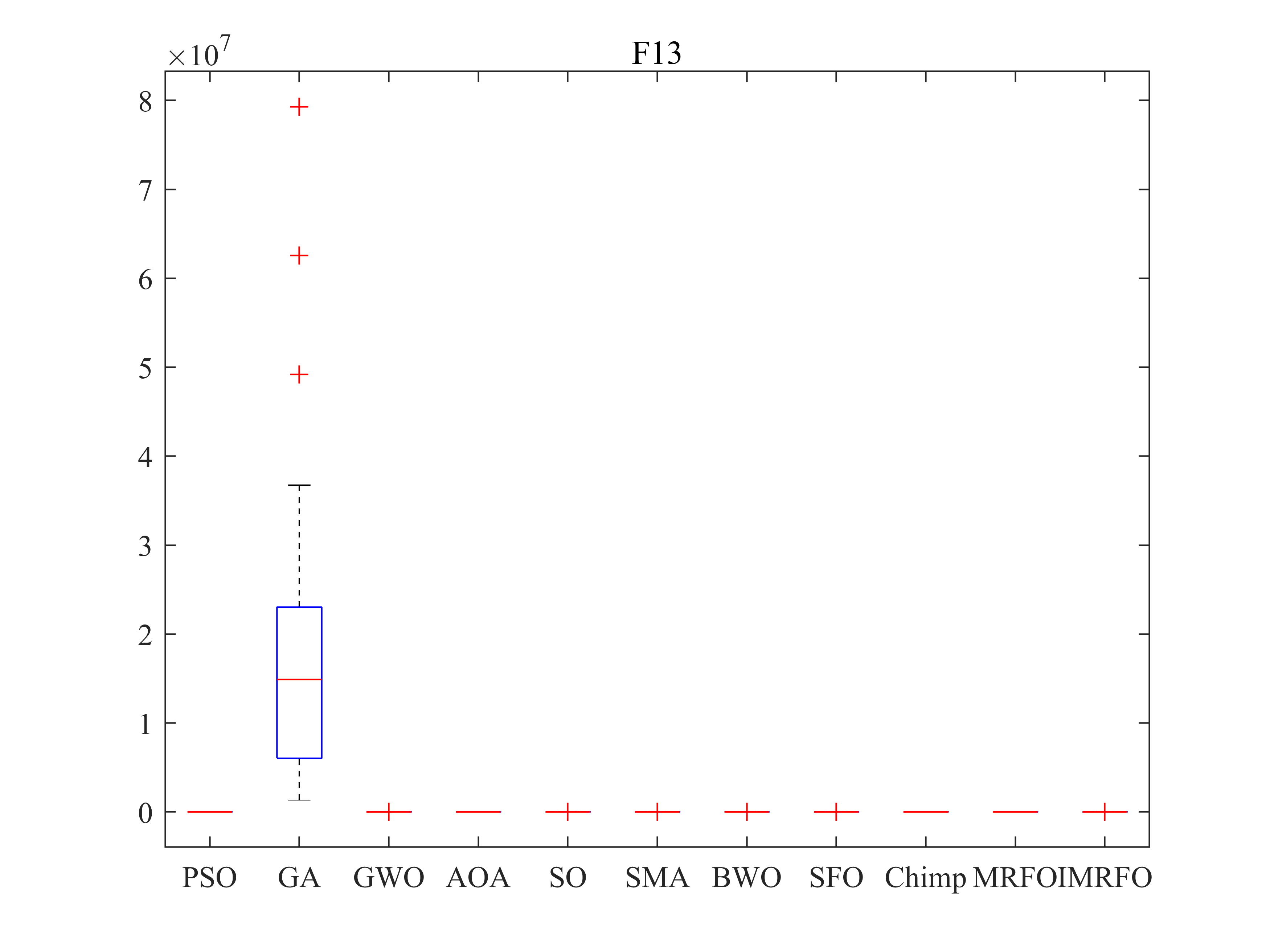

Supplement: Supplementary file 1 — Supplementary Information. [file 41598_2024_59960_MOESM1_ESM.zip › All research figures/All research figures/1 Figures of benchmark functions/Benchmark-functions-box-figure/13.tif]

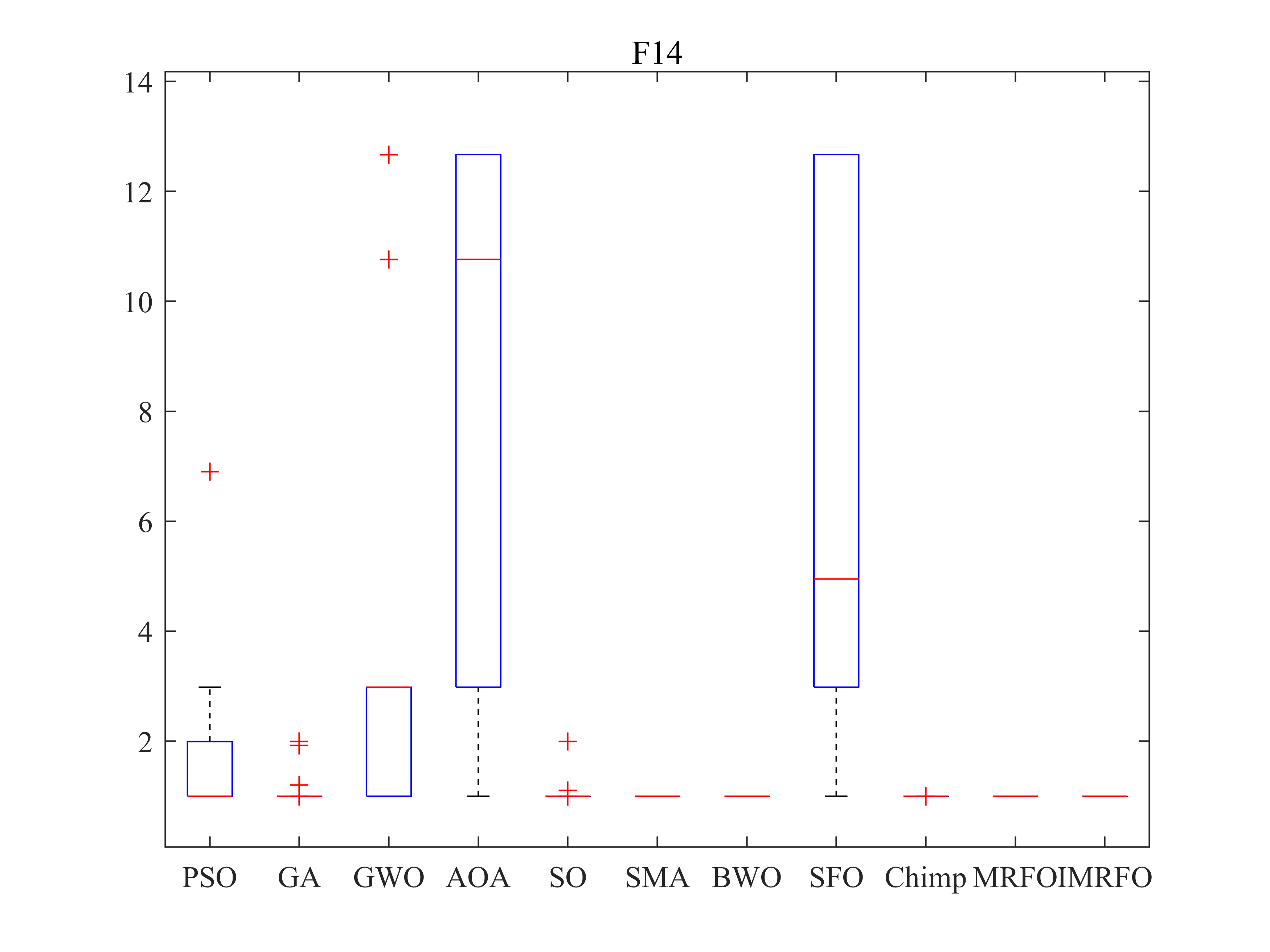

Supplement: Supplementary file 1 — Supplementary Information. [file 41598_2024_59960_MOESM1_ESM.zip › All research figures/All research figures/1 Figures of benchmark functions/Benchmark-functions-box-figure/14.tif]

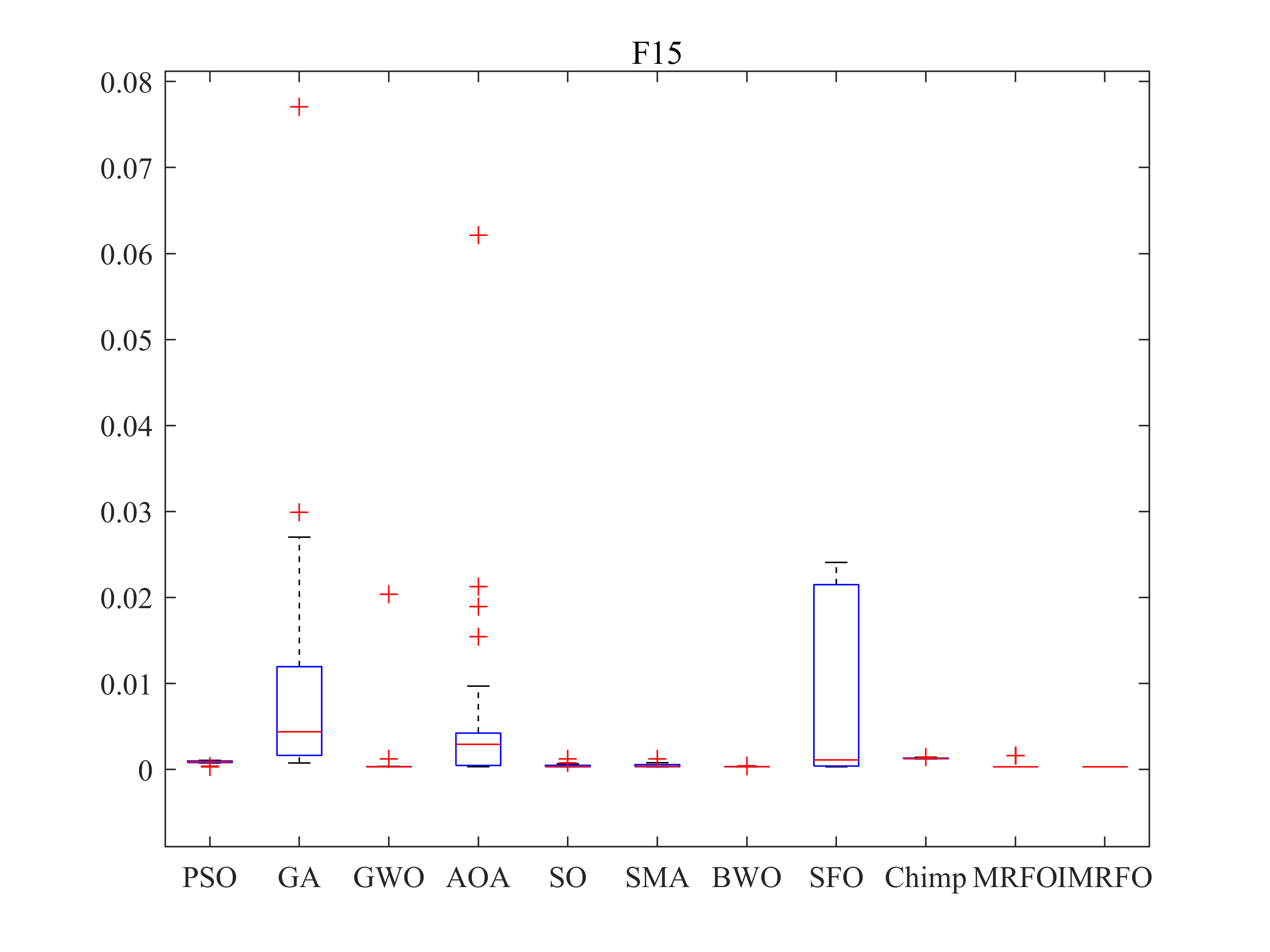

Supplement: Supplementary file 1 — Supplementary Information. [file 41598_2024_59960_MOESM1_ESM.zip › All research figures/All research figures/1 Figures of benchmark functions/Benchmark-functions-box-figure/15.tif]

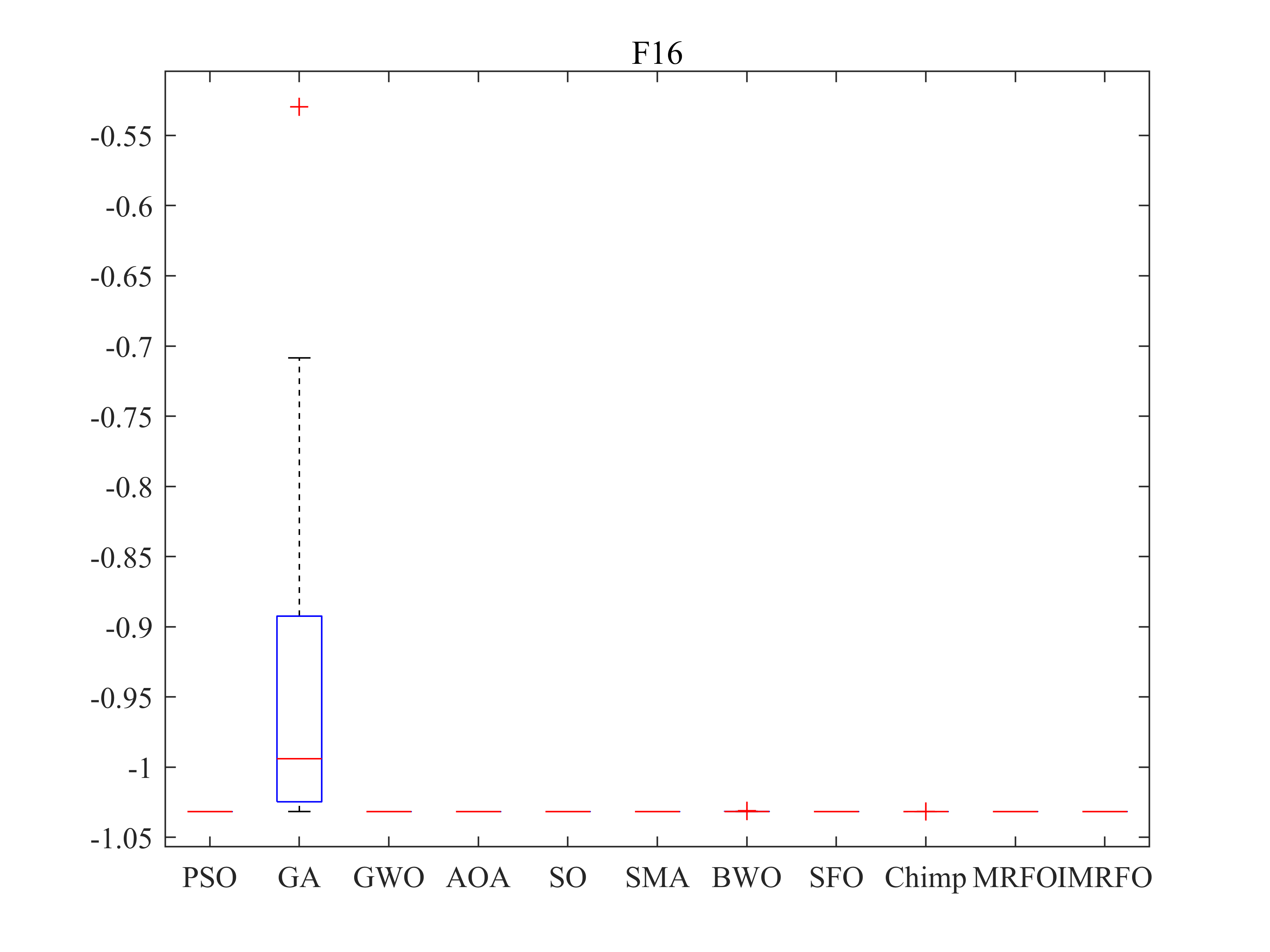

Supplement: Supplementary file 1 — Supplementary Information. [file 41598_2024_59960_MOESM1_ESM.zip › All research figures/All research figures/1 Figures of benchmark functions/Benchmark-functions-box-figure/16.tif]

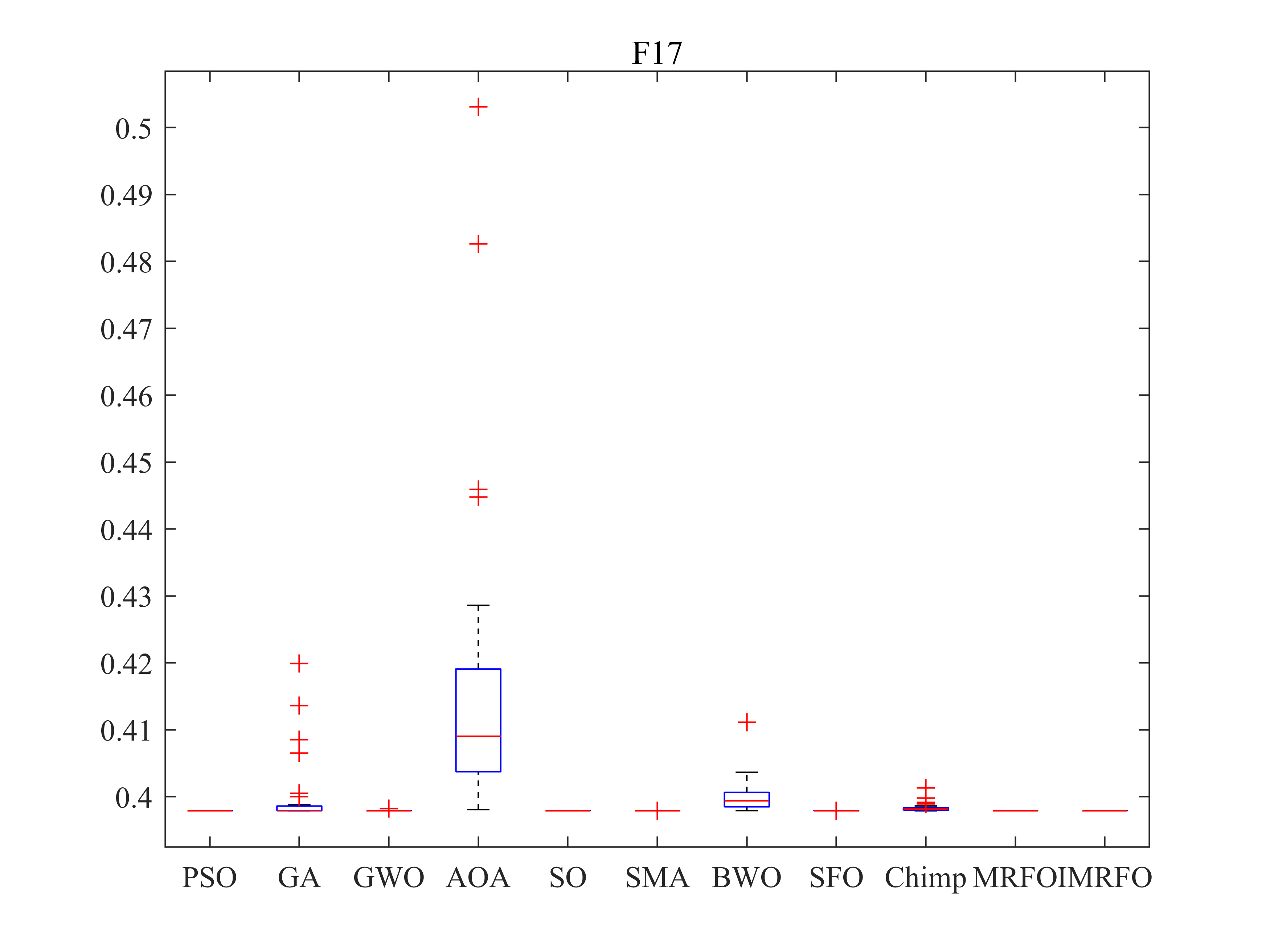

Supplement: Supplementary file 1 — Supplementary Information. [file 41598_2024_59960_MOESM1_ESM.zip › All research figures/All research figures/1 Figures of benchmark functions/Benchmark-functions-box-figure/17.tif]

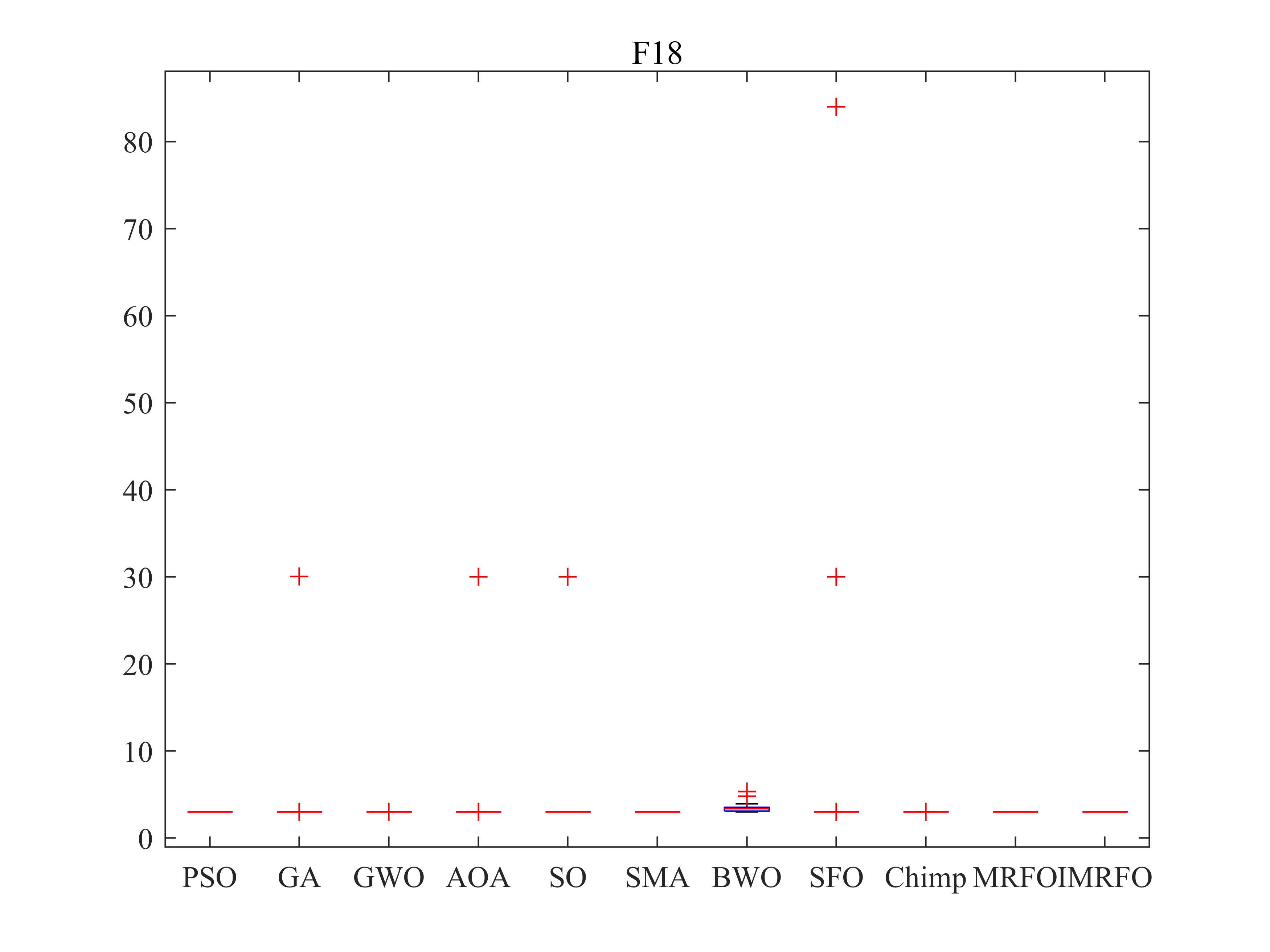

Supplement: Supplementary file 1 — Supplementary Information. [file 41598_2024_59960_MOESM1_ESM.zip › All research figures/All research figures/1 Figures of benchmark functions/Benchmark-functions-box-figure/18.tif]

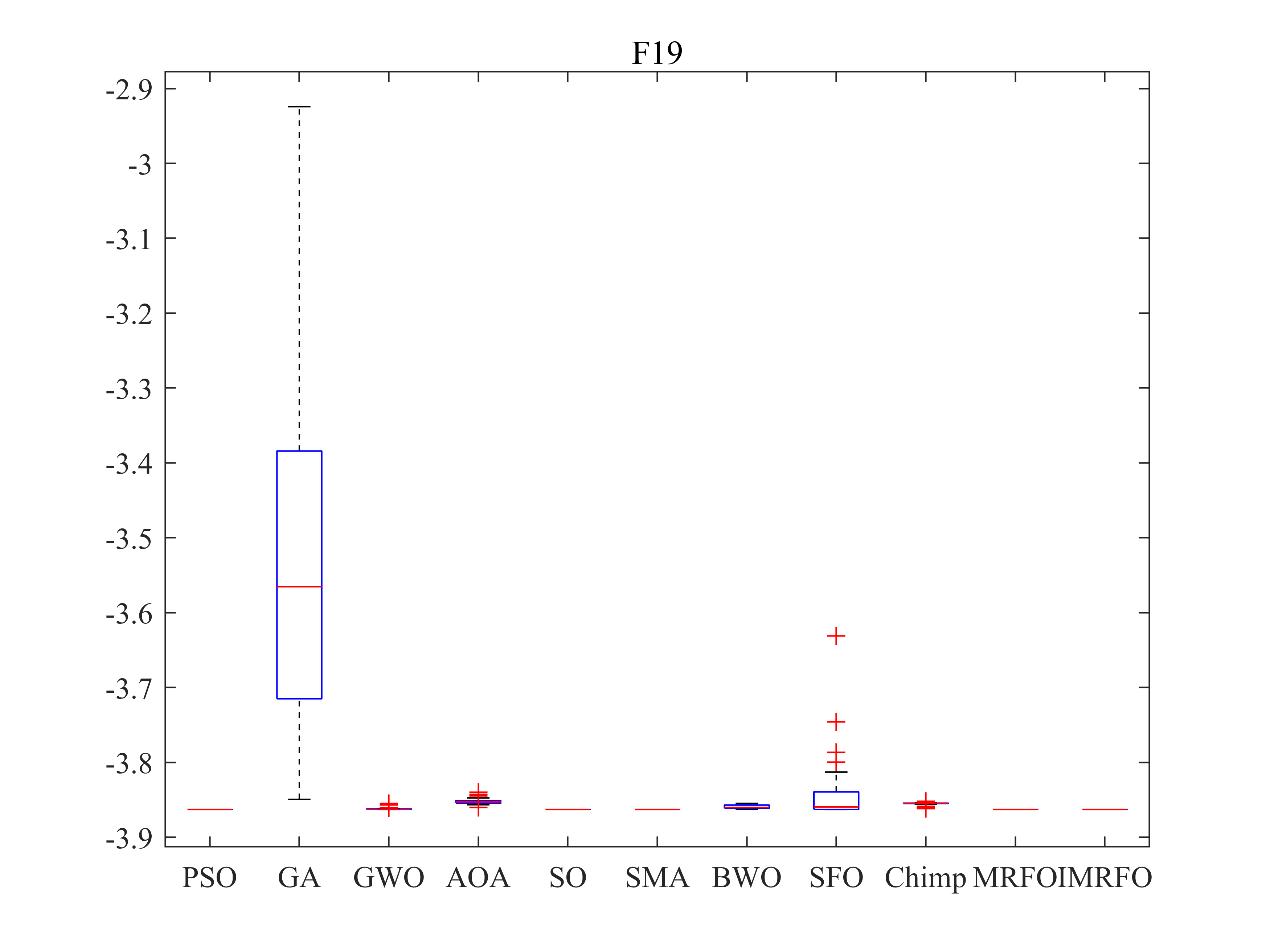

Supplement: Supplementary file 1 — Supplementary Information. [file 41598_2024_59960_MOESM1_ESM.zip › All research figures/All research figures/1 Figures of benchmark functions/Benchmark-functions-box-figure/19.tif]

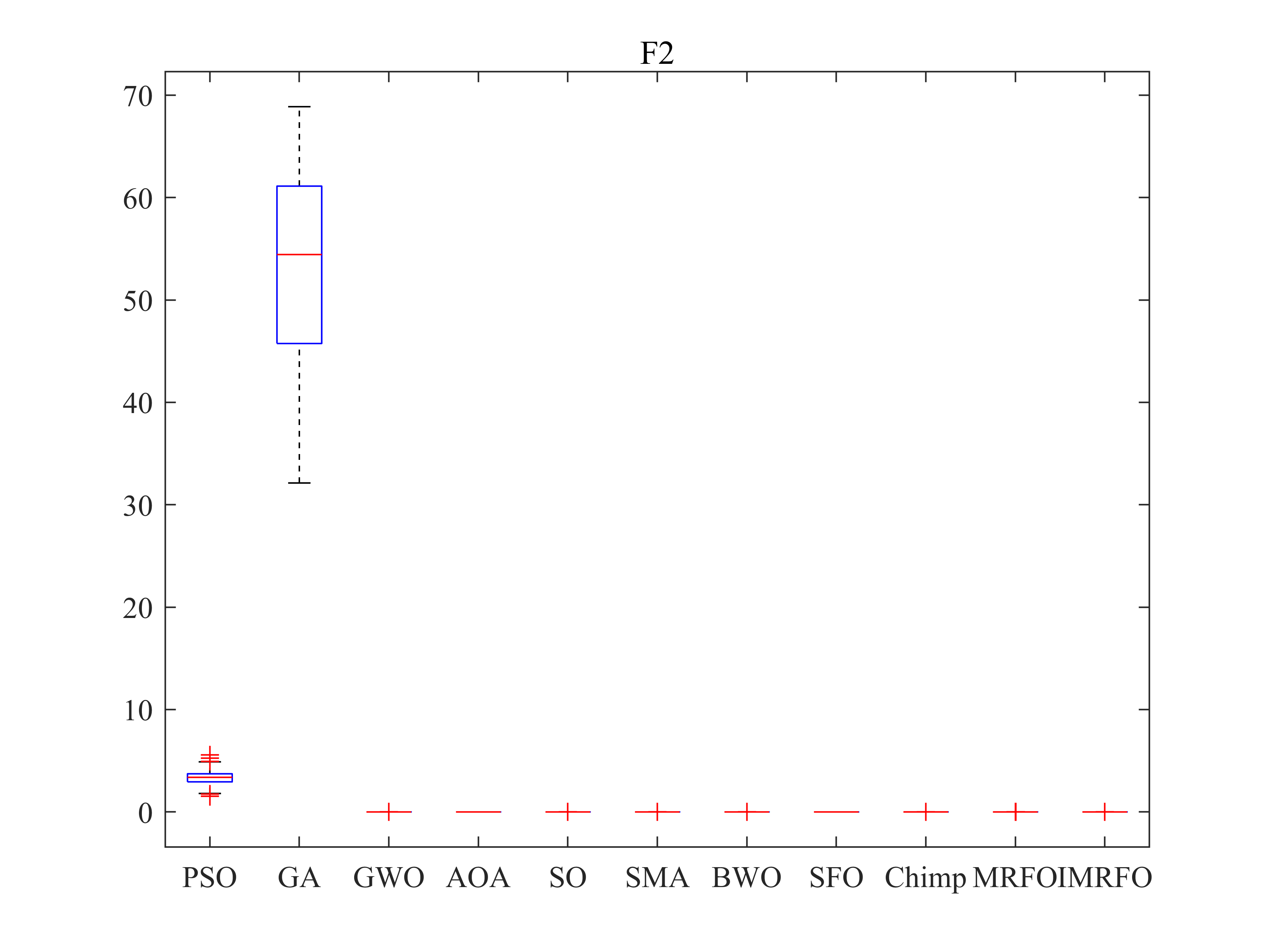

Supplement: Supplementary file 1 — Supplementary Information. [file 41598_2024_59960_MOESM1_ESM.zip › All research figures/All research figures/1 Figures of benchmark functions/Benchmark-functions-box-figure/2.tif]

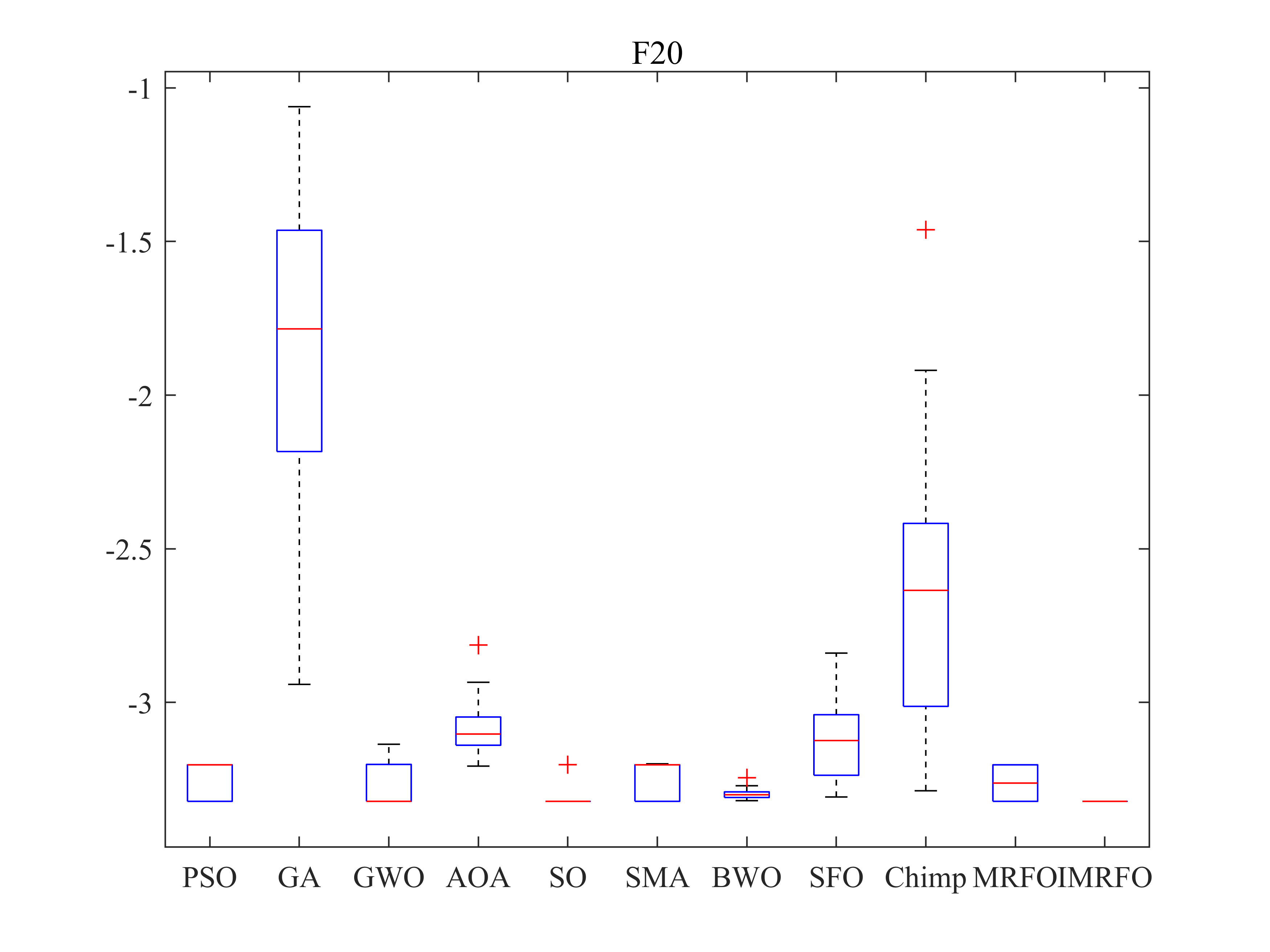

Supplement: Supplementary file 1 — Supplementary Information. [file 41598_2024_59960_MOESM1_ESM.zip › All research figures/All research figures/1 Figures of benchmark functions/Benchmark-functions-box-figure/20.tif]

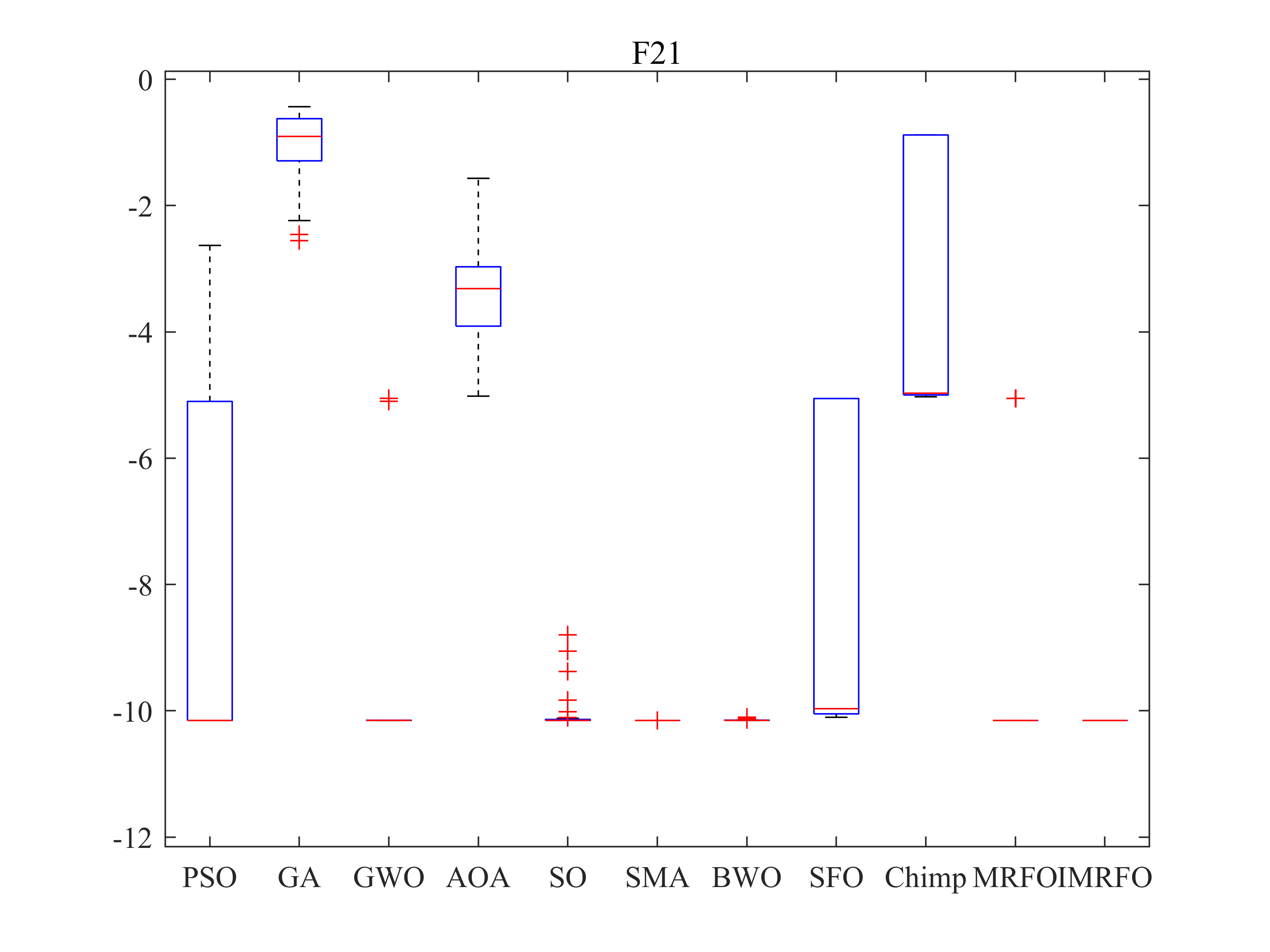

Supplement: Supplementary file 1 — Supplementary Information. [file 41598_2024_59960_MOESM1_ESM.zip › All research figures/All research figures/1 Figures of benchmark functions/Benchmark-functions-box-figure/21.tif]

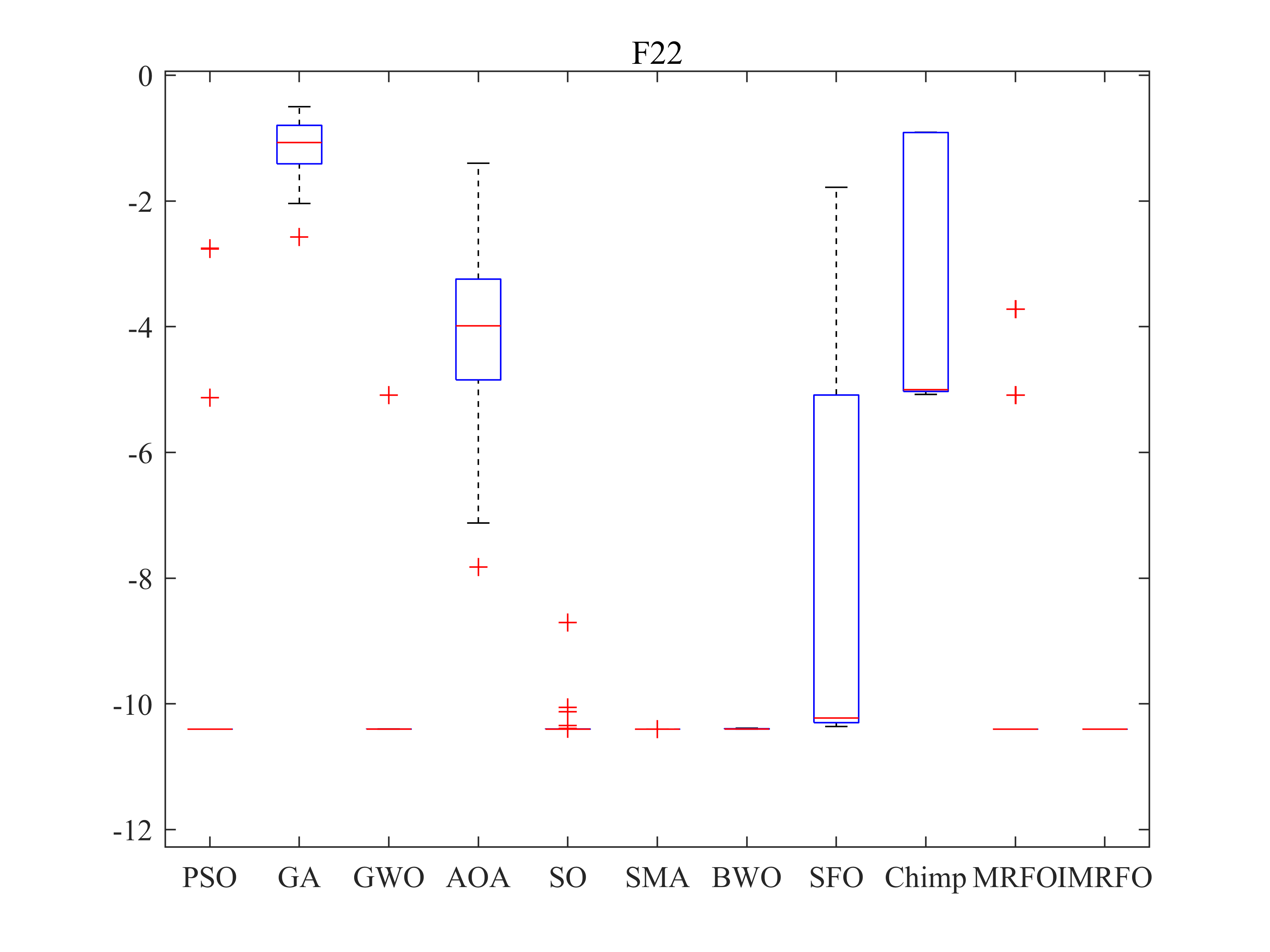

Supplement: Supplementary file 1 — Supplementary Information. [file 41598_2024_59960_MOESM1_ESM.zip › All research figures/All research figures/1 Figures of benchmark functions/Benchmark-functions-box-figure/22.tif]

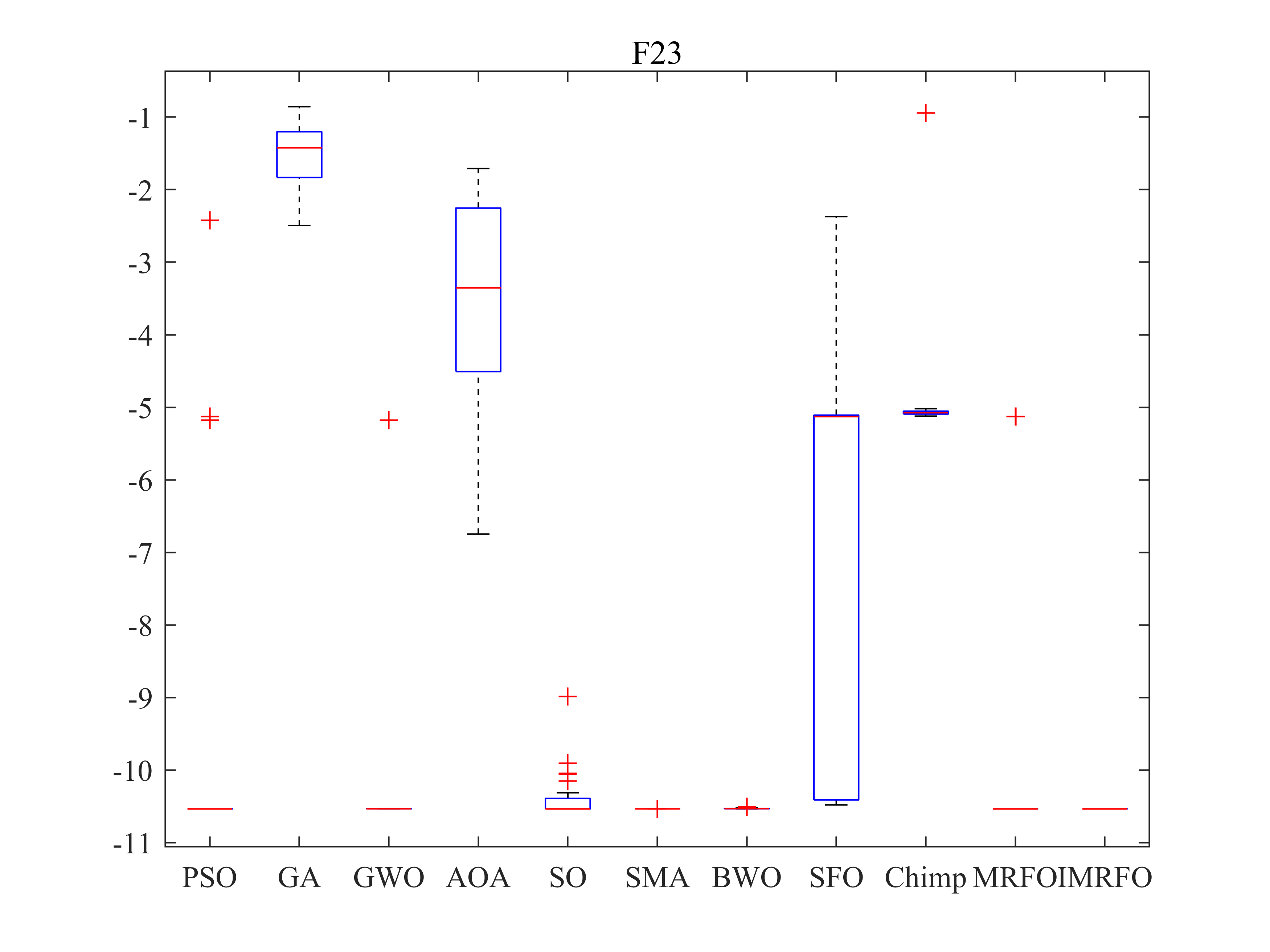

Supplement: Supplementary file 1 — Supplementary Information. [file 41598_2024_59960_MOESM1_ESM.zip › All research figures/All research figures/1 Figures of benchmark functions/Benchmark-functions-box-figure/23.tif]

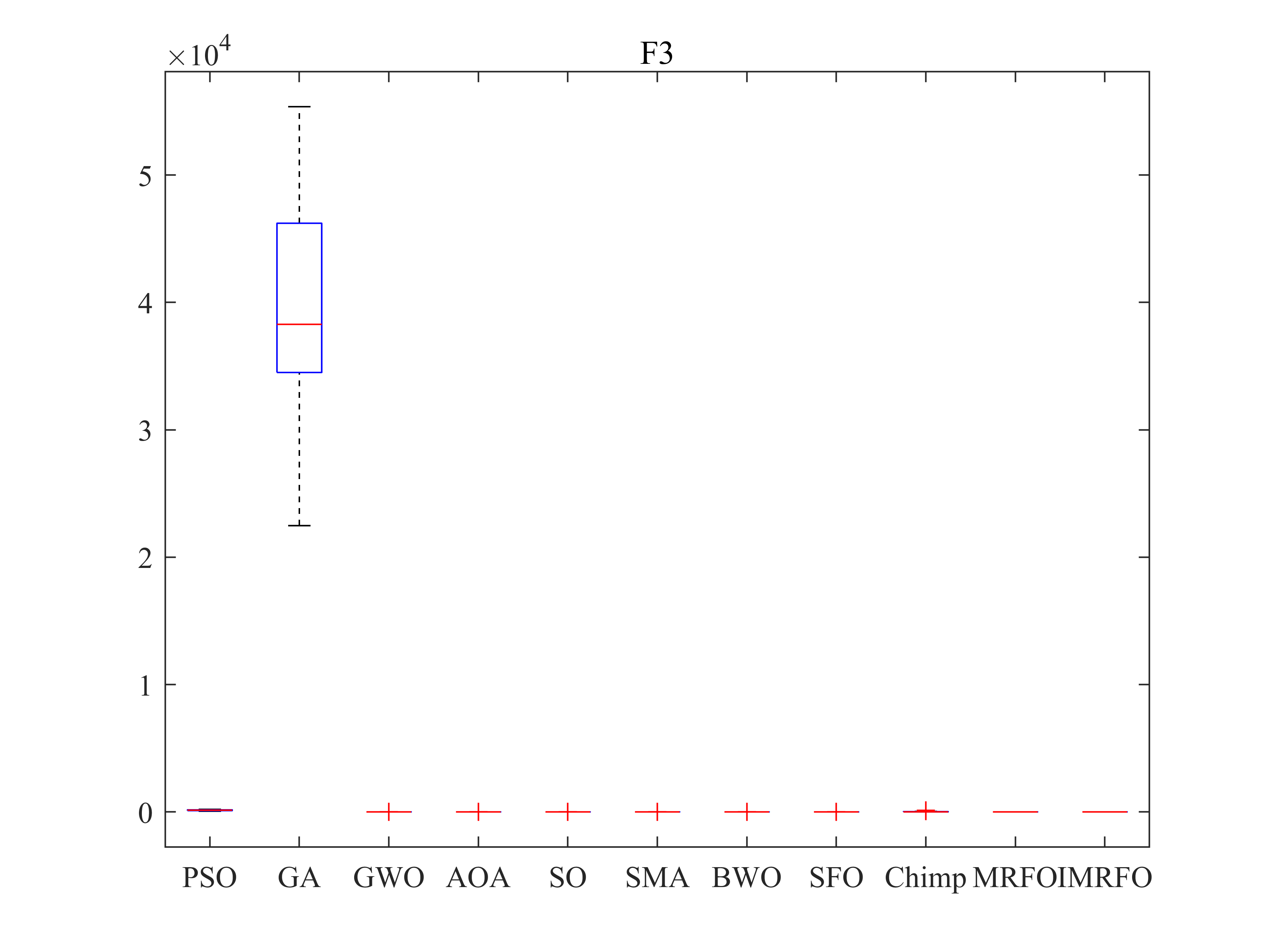

Supplement: Supplementary file 1 — Supplementary Information. [file 41598_2024_59960_MOESM1_ESM.zip › All research figures/All research figures/1 Figures of benchmark functions/Benchmark-functions-box-figure/3.tif]

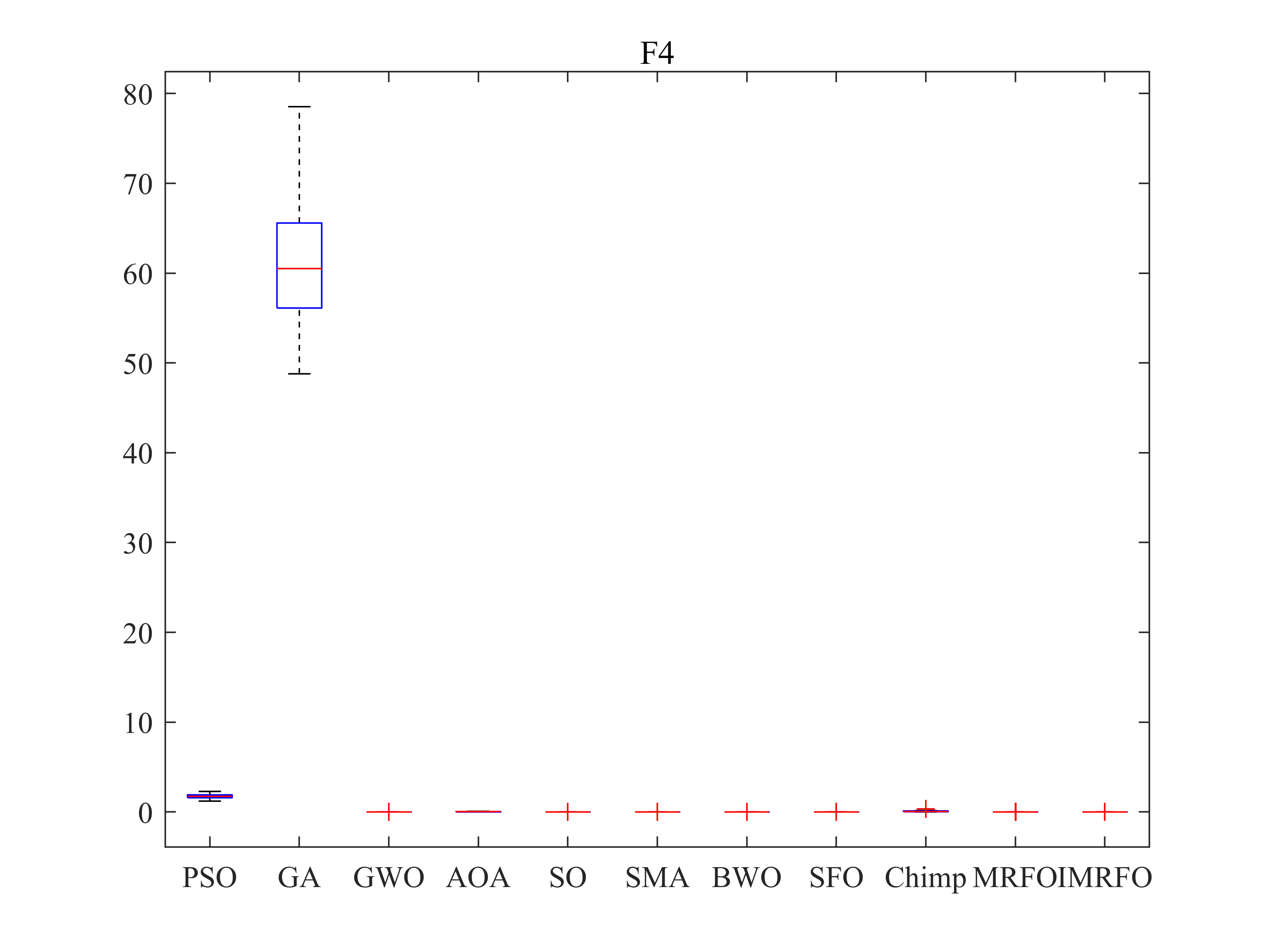

Supplement: Supplementary file 1 — Supplementary Information. [file 41598_2024_59960_MOESM1_ESM.zip › All research figures/All research figures/1 Figures of benchmark functions/Benchmark-functions-box-figure/4.tif]

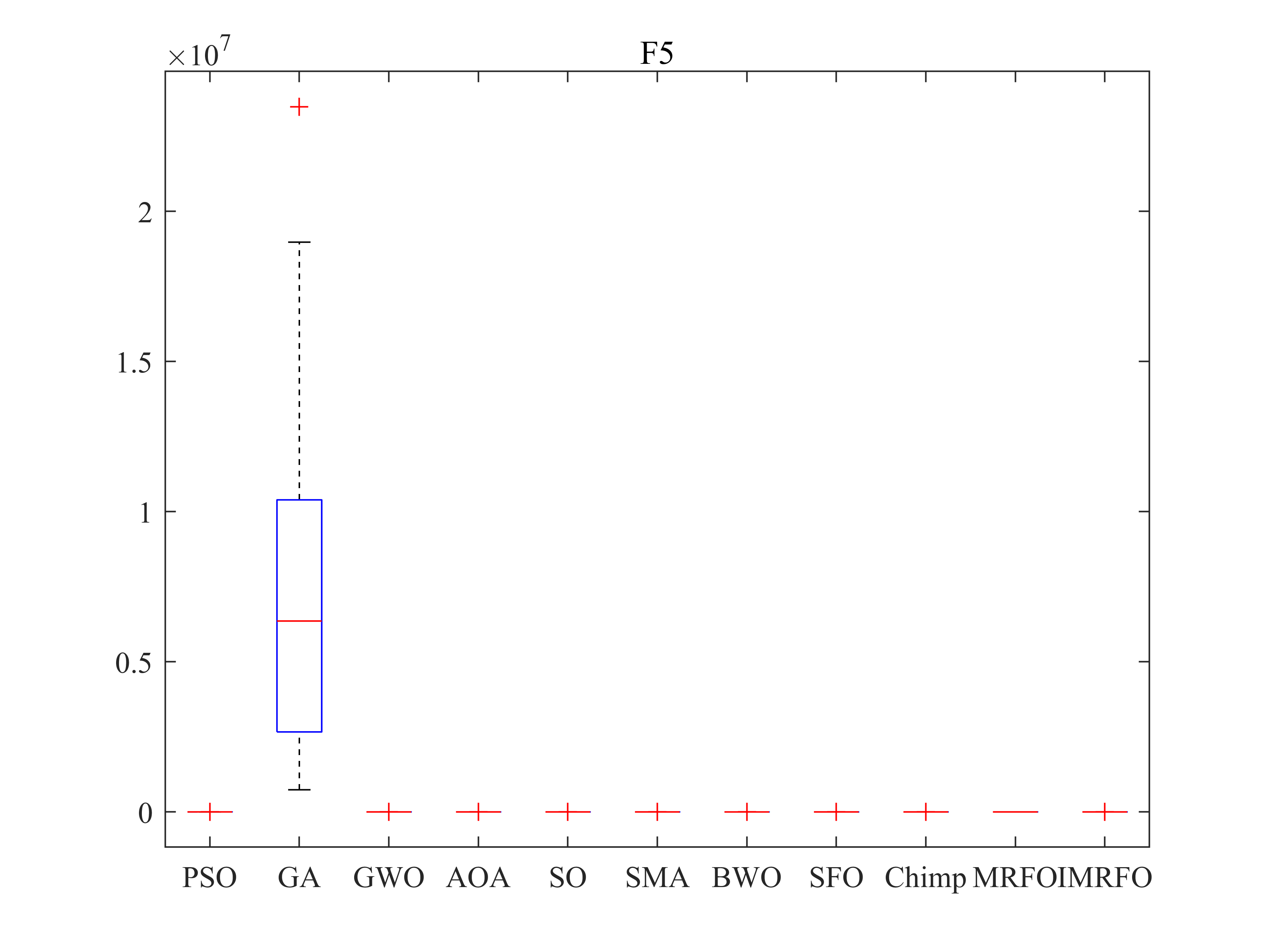

Supplement: Supplementary file 1 — Supplementary Information. [file 41598_2024_59960_MOESM1_ESM.zip › All research figures/All research figures/1 Figures of benchmark functions/Benchmark-functions-box-figure/5.tif]

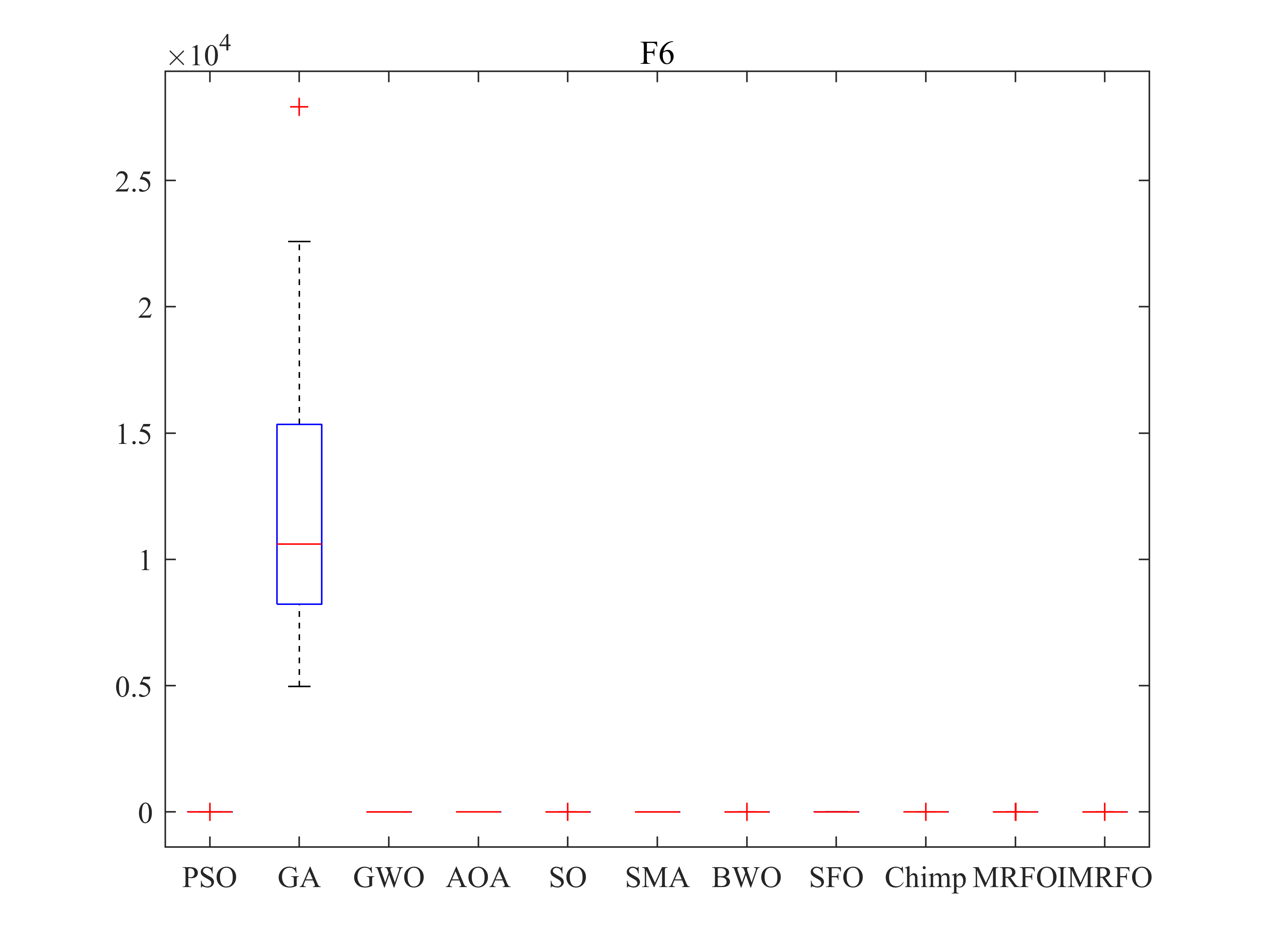

Supplement: Supplementary file 1 — Supplementary Information. [file 41598_2024_59960_MOESM1_ESM.zip › All research figures/All research figures/1 Figures of benchmark functions/Benchmark-functions-box-figure/6.tif]

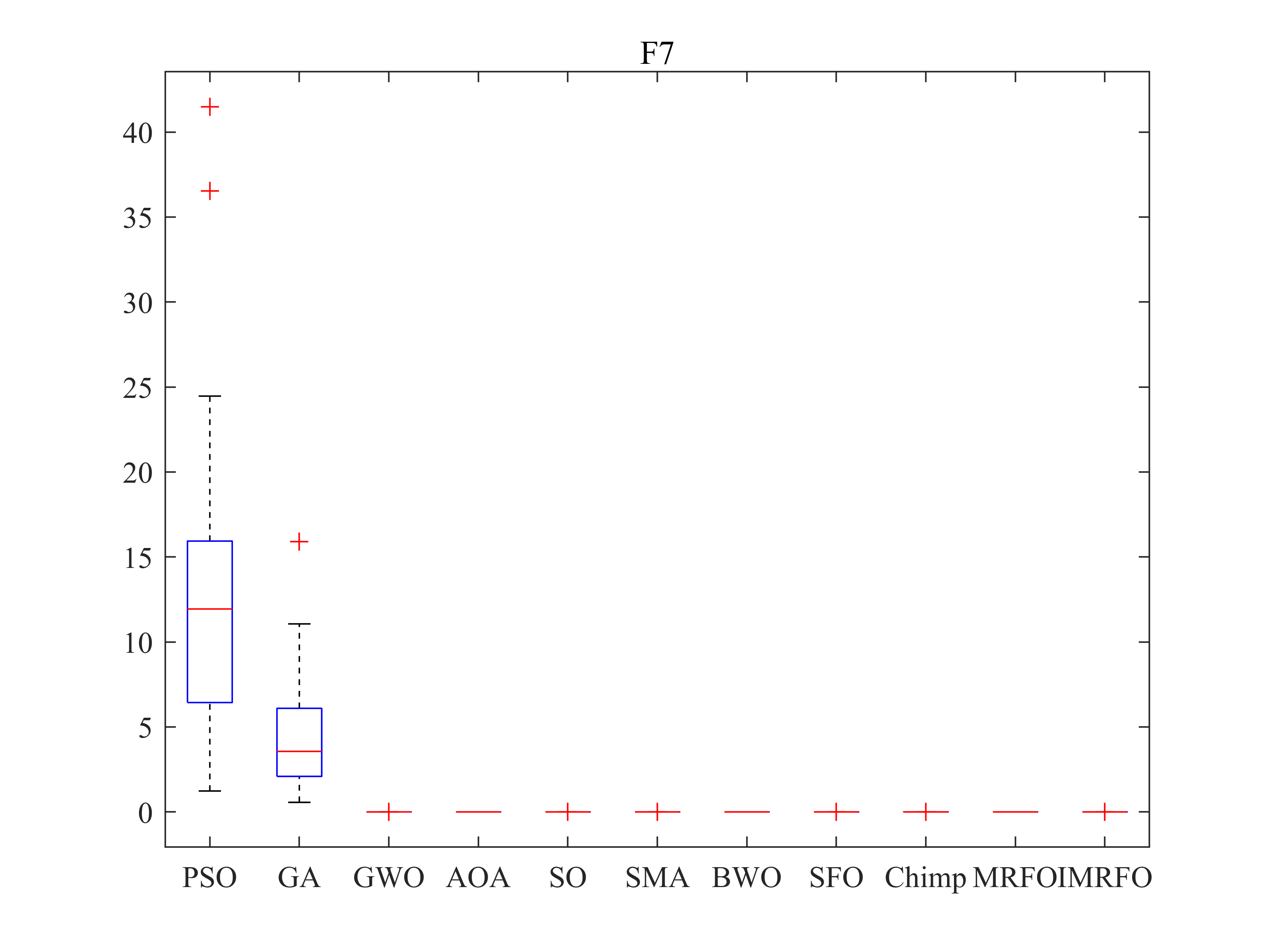

Supplement: Supplementary file 1 — Supplementary Information. [file 41598_2024_59960_MOESM1_ESM.zip › All research figures/All research figures/1 Figures of benchmark functions/Benchmark-functions-box-figure/7.tif]

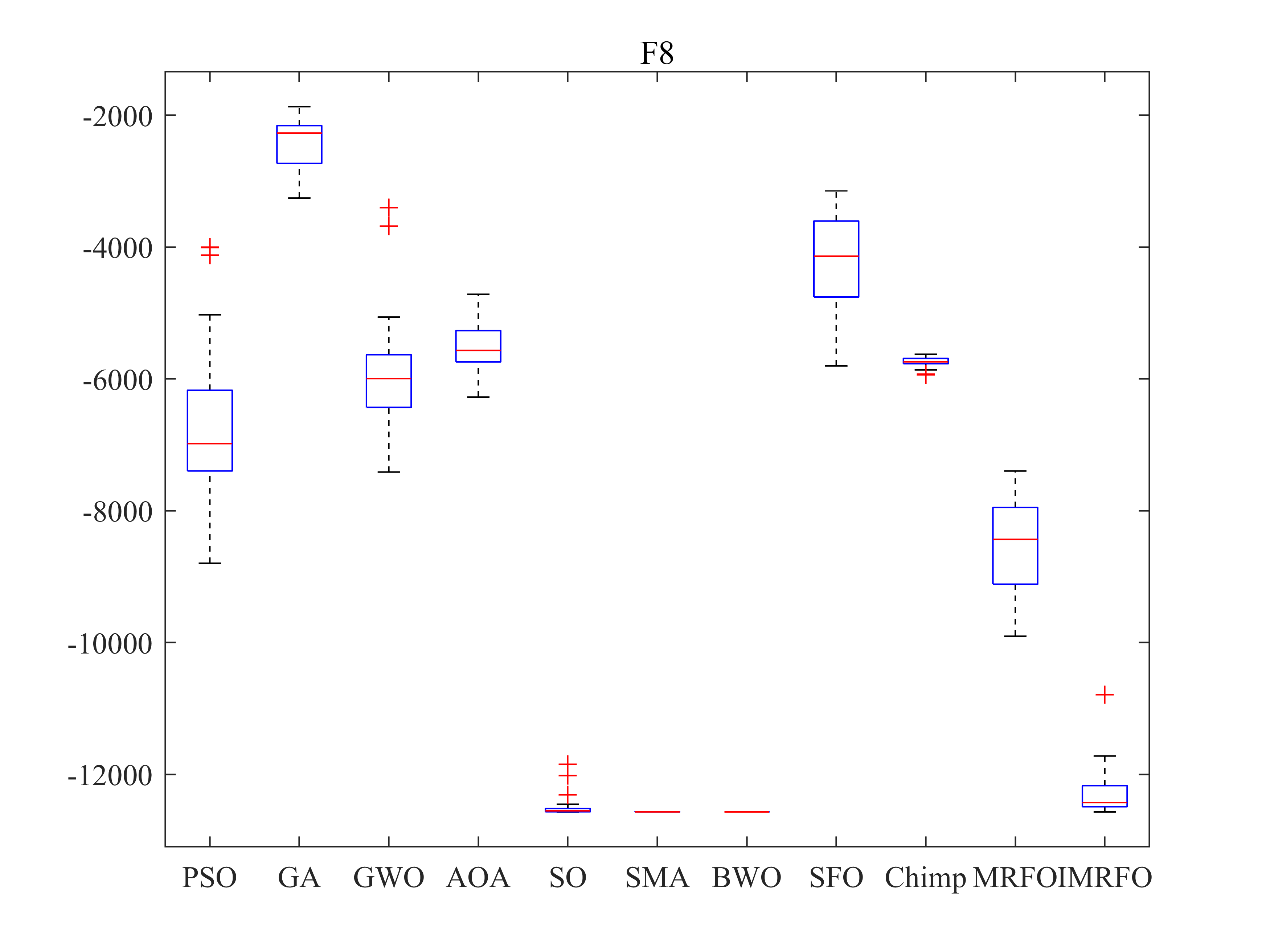

Supplement: Supplementary file 1 — Supplementary Information. [file 41598_2024_59960_MOESM1_ESM.zip › All research figures/All research figures/1 Figures of benchmark functions/Benchmark-functions-box-figure/8.tif]

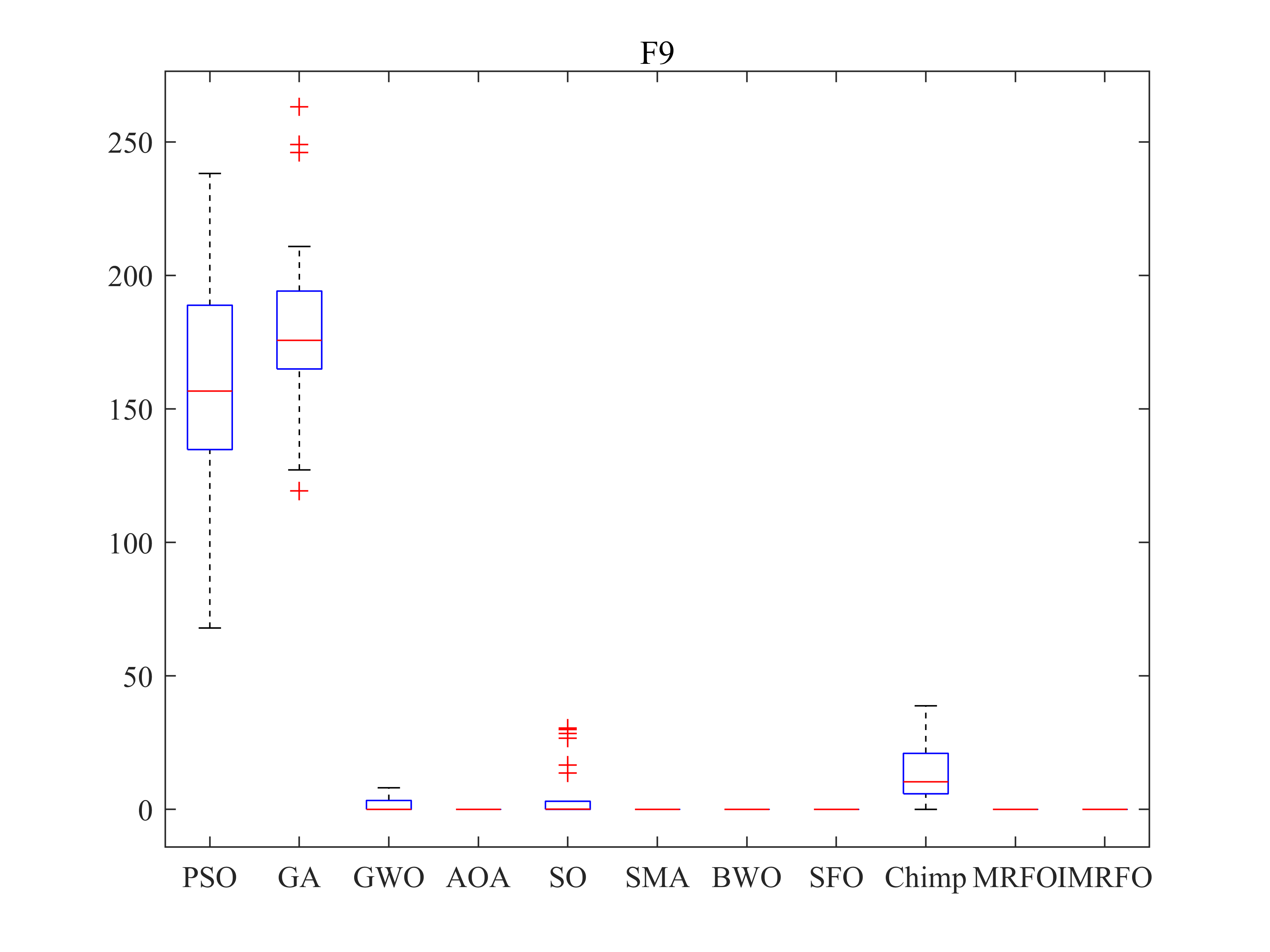

Supplement: Supplementary file 1 — Supplementary Information. [file 41598_2024_59960_MOESM1_ESM.zip › All research figures/All research figures/1 Figures of benchmark functions/Benchmark-functions-box-figure/9.tif]

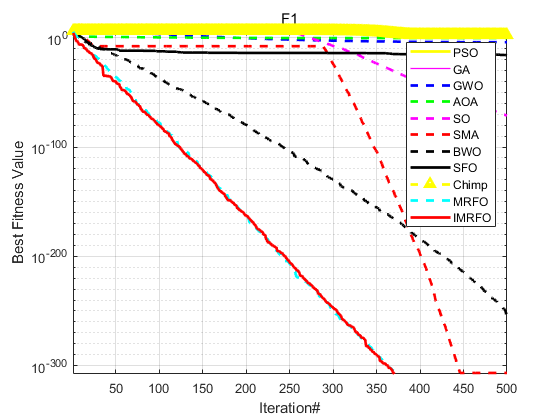

Supplement: Supplementary file 1 — Supplementary Information. [file 41598_2024_59960_MOESM1_ESM.zip › All research figures/All research figures/1 Figures of benchmark functions/Figures of all benchmark functions/1/1-1.tif]

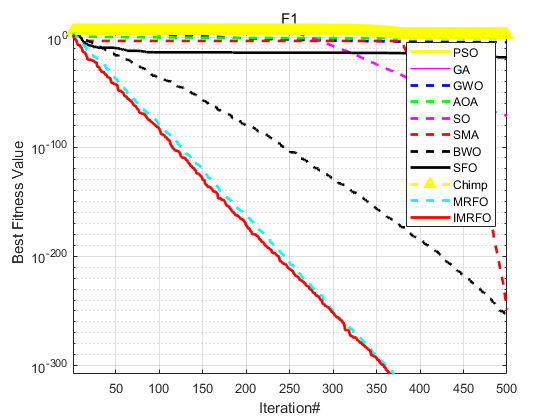

Supplement: Supplementary file 1 — Supplementary Information. [file 41598_2024_59960_MOESM1_ESM.zip › All research figures/All research figures/1 Figures of benchmark functions/Figures of all benchmark functions/1/1-10.tif]

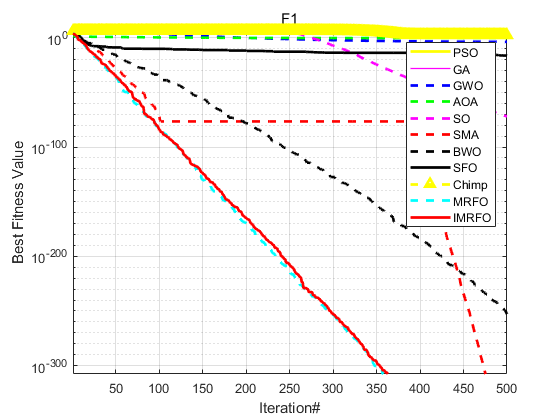

Supplement: Supplementary file 1 — Supplementary Information. [file 41598_2024_59960_MOESM1_ESM.zip › All research figures/All research figures/1 Figures of benchmark functions/Figures of all benchmark functions/1/1-11.tif]

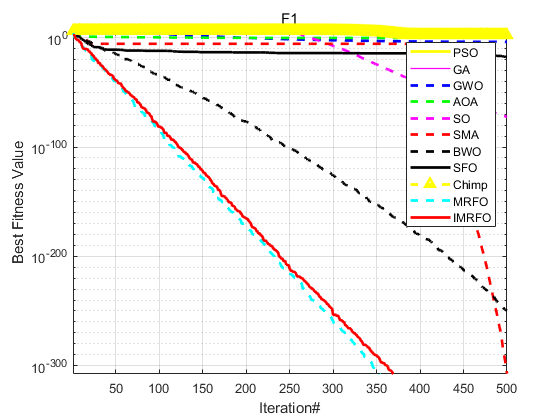

Supplement: Supplementary file 1 — Supplementary Information. [file 41598_2024_59960_MOESM1_ESM.zip › All research figures/All research figures/1 Figures of benchmark functions/Figures of all benchmark functions/1/1-12.tif]

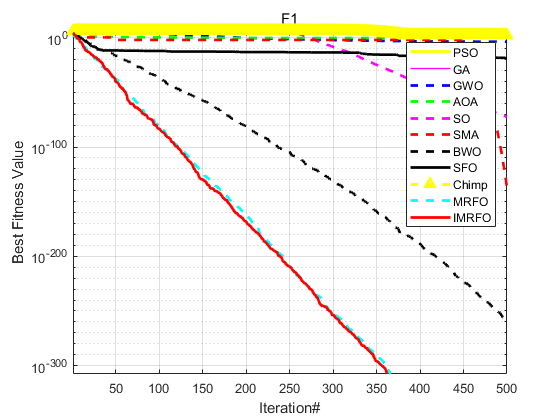

Supplement: Supplementary file 1 — Supplementary Information. [file 41598_2024_59960_MOESM1_ESM.zip › All research figures/All research figures/1 Figures of benchmark functions/Figures of all benchmark functions/1/1-13.tif]

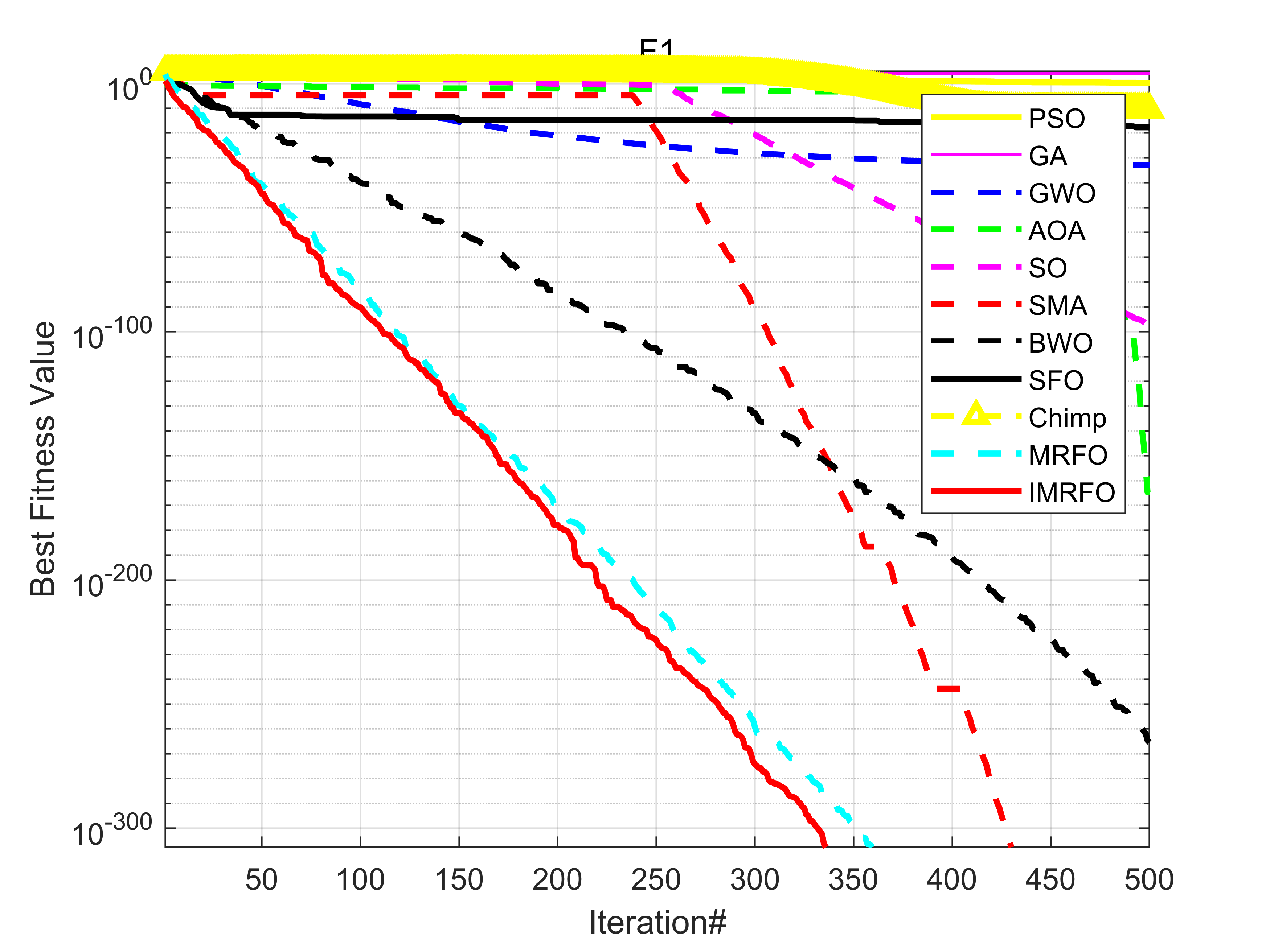

Supplement: Supplementary file 1 — Supplementary Information. [file 41598_2024_59960_MOESM1_ESM.zip › All research figures/All research figures/1 Figures of benchmark functions/Figures of all benchmark functions/1/1-14.tif]

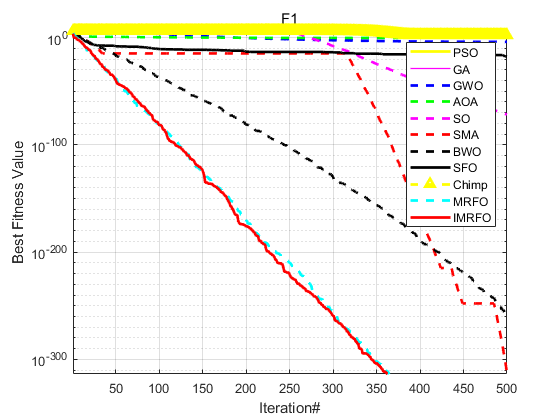

Supplement: Supplementary file 1 — Supplementary Information. [file 41598_2024_59960_MOESM1_ESM.zip › All research figures/All research figures/1 Figures of benchmark functions/Figures of all benchmark functions/1/1-15.tif]

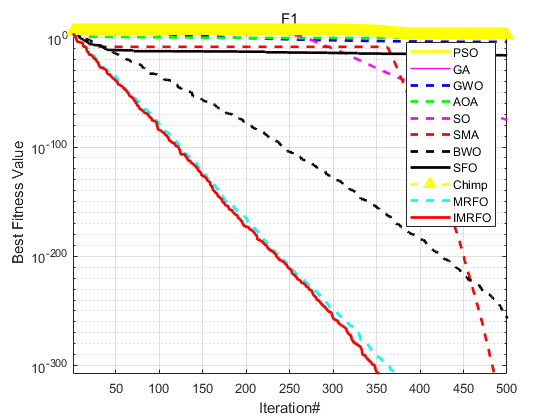

Supplement: Supplementary file 1 — Supplementary Information. [file 41598_2024_59960_MOESM1_ESM.zip › All research figures/All research figures/1 Figures of benchmark functions/Figures of all benchmark functions/1/1-16.tif]

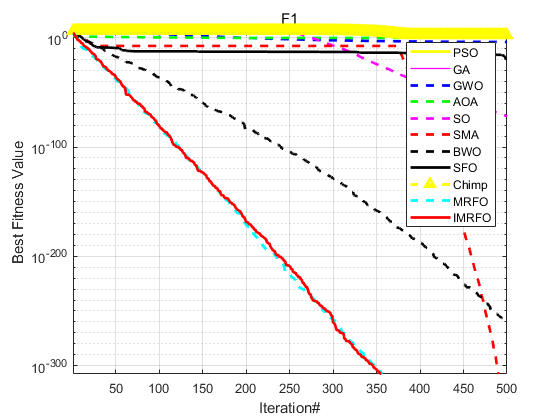

Supplement: Supplementary file 1 — Supplementary Information. [file 41598_2024_59960_MOESM1_ESM.zip › All research figures/All research figures/1 Figures of benchmark functions/Figures of all benchmark functions/1/1-17.tif]

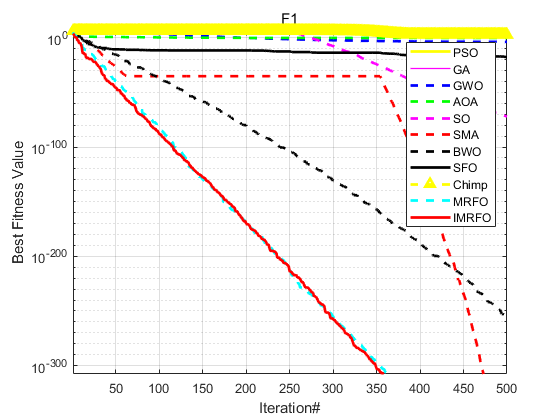

Supplement: Supplementary file 1 — Supplementary Information. [file 41598_2024_59960_MOESM1_ESM.zip › All research figures/All research figures/1 Figures of benchmark functions/Figures of all benchmark functions/1/1-18.tif]

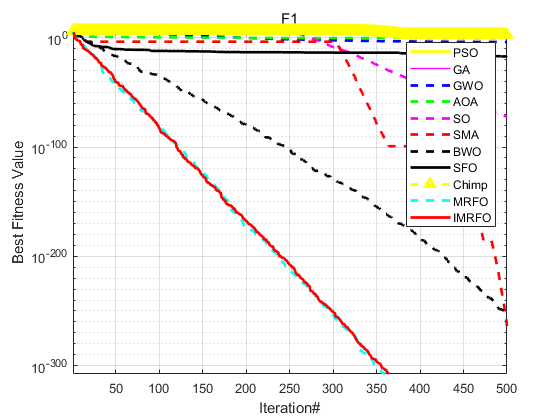

Supplement: Supplementary file 1 — Supplementary Information. [file 41598_2024_59960_MOESM1_ESM.zip › All research figures/All research figures/1 Figures of benchmark functions/Figures of all benchmark functions/1/1-19.tif]

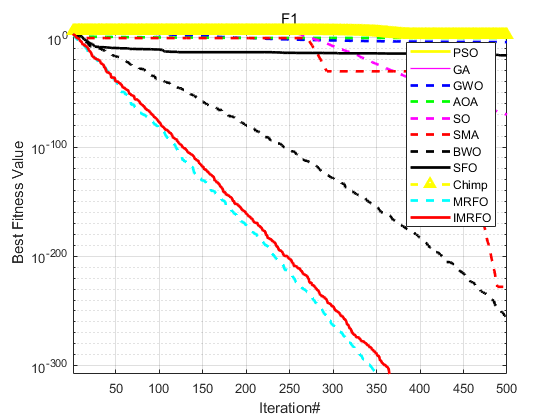

Supplement: Supplementary file 1 — Supplementary Information. [file 41598_2024_59960_MOESM1_ESM.zip › All research figures/All research figures/1 Figures of benchmark functions/Figures of all benchmark functions/1/1-2.tif]

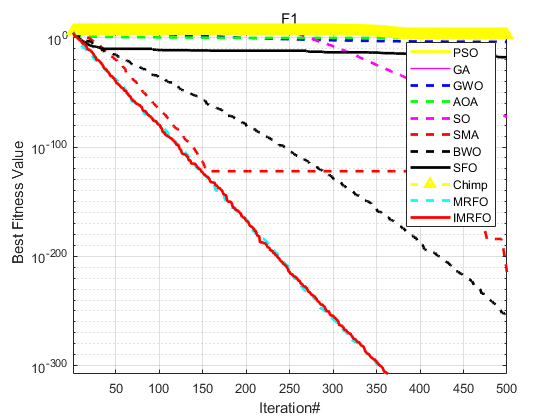

Supplement: Supplementary file 1 — Supplementary Information. [file 41598_2024_59960_MOESM1_ESM.zip › All research figures/All research figures/1 Figures of benchmark functions/Figures of all benchmark functions/1/1-20.tif]

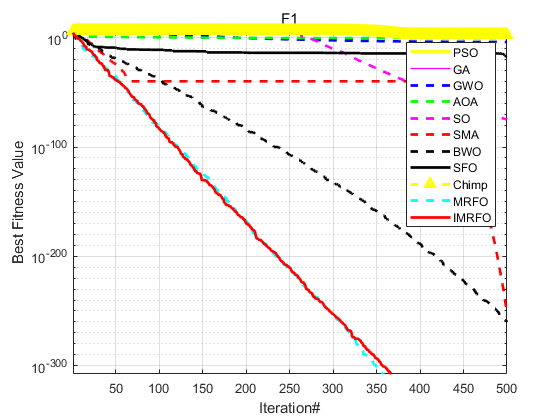

Supplement: Supplementary file 1 — Supplementary Information. [file 41598_2024_59960_MOESM1_ESM.zip › All research figures/All research figures/1 Figures of benchmark functions/Figures of all benchmark functions/1/1-21.tif]

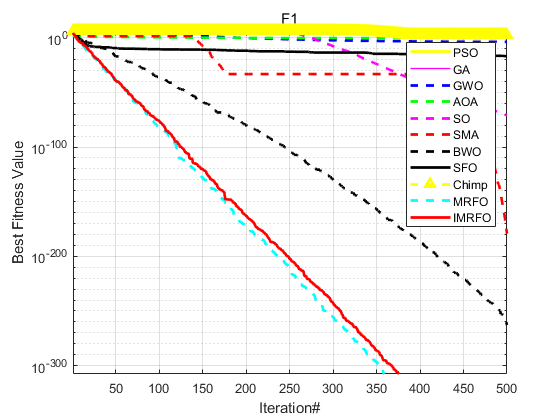

Supplement: Supplementary file 1 — Supplementary Information. [file 41598_2024_59960_MOESM1_ESM.zip › All research figures/All research figures/1 Figures of benchmark functions/Figures of all benchmark functions/1/1-22.tif]

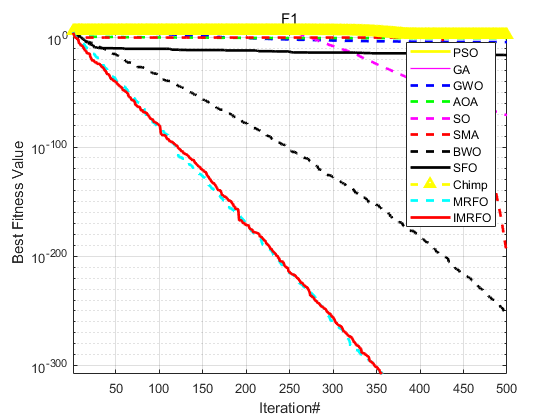

Supplement: Supplementary file 1 — Supplementary Information. [file 41598_2024_59960_MOESM1_ESM.zip › All research figures/All research figures/1 Figures of benchmark functions/Figures of all benchmark functions/1/1-23.tif]

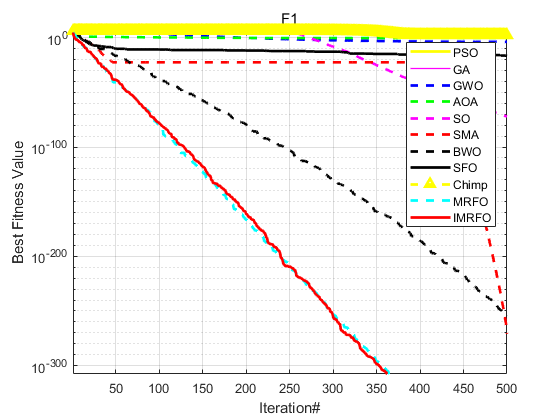

Supplement: Supplementary file 1 — Supplementary Information. [file 41598_2024_59960_MOESM1_ESM.zip › All research figures/All research figures/1 Figures of benchmark functions/Figures of all benchmark functions/1/1-24.tif]

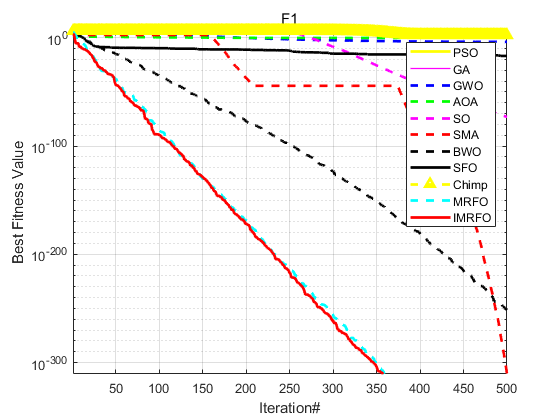

Supplement: Supplementary file 1 — Supplementary Information. [file 41598_2024_59960_MOESM1_ESM.zip › All research figures/All research figures/1 Figures of benchmark functions/Figures of all benchmark functions/1/1-25.tif]

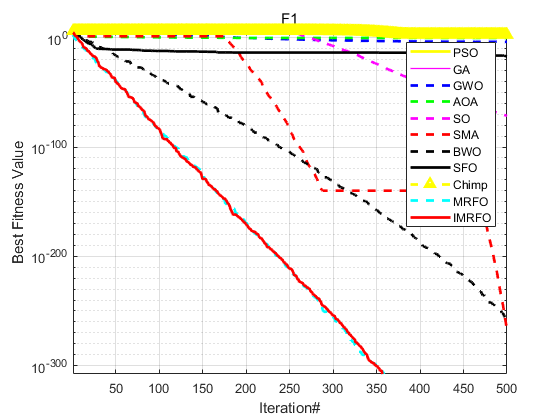

Supplement: Supplementary file 1 — Supplementary Information. [file 41598_2024_59960_MOESM1_ESM.zip › All research figures/All research figures/1 Figures of benchmark functions/Figures of all benchmark functions/1/1-26.tif]

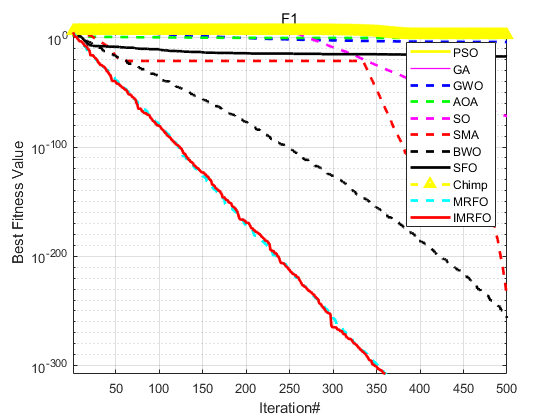

Supplement: Supplementary file 1 — Supplementary Information. [file 41598_2024_59960_MOESM1_ESM.zip › All research figures/All research figures/1 Figures of benchmark functions/Figures of all benchmark functions/1/1-27.tif]

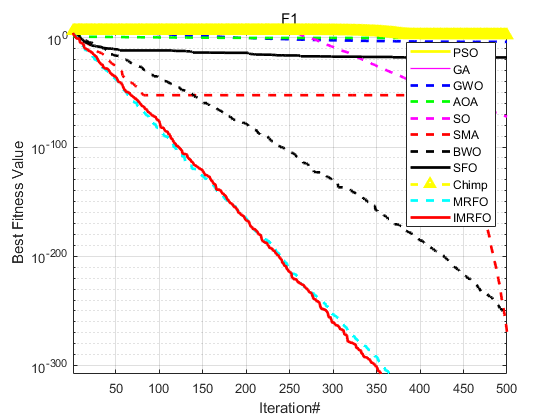

Supplement: Supplementary file 1 — Supplementary Information. [file 41598_2024_59960_MOESM1_ESM.zip › All research figures/All research figures/1 Figures of benchmark functions/Figures of all benchmark functions/1/1-28.tif]

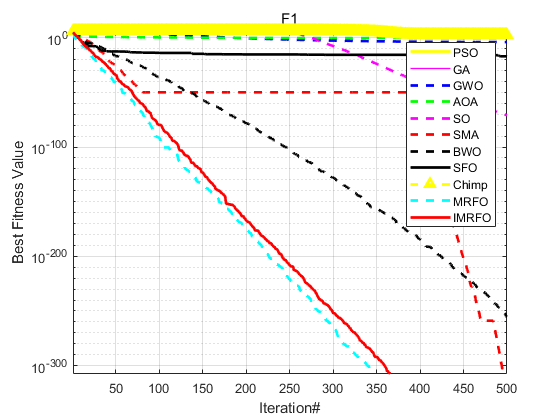

Supplement: Supplementary file 1 — Supplementary Information. [file 41598_2024_59960_MOESM1_ESM.zip › All research figures/All research figures/1 Figures of benchmark functions/Figures of all benchmark functions/1/1-29.tif]

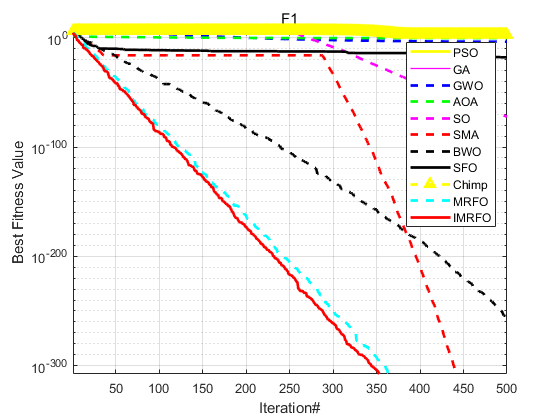

Supplement: Supplementary file 1 — Supplementary Information. [file 41598_2024_59960_MOESM1_ESM.zip › All research figures/All research figures/1 Figures of benchmark functions/Figures of all benchmark functions/1/1-3.tif]

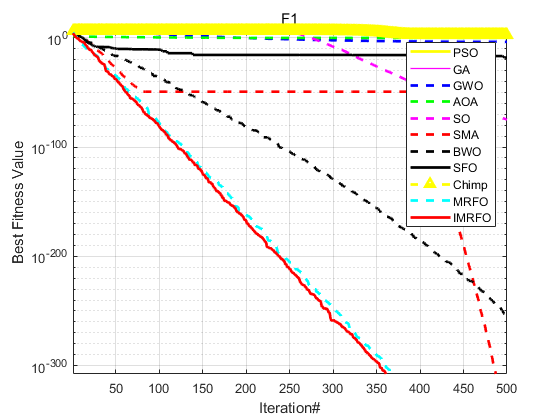

Supplement: Supplementary file 1 — Supplementary Information. [file 41598_2024_59960_MOESM1_ESM.zip › All research figures/All research figures/1 Figures of benchmark functions/Figures of all benchmark functions/1/1-30.tif]

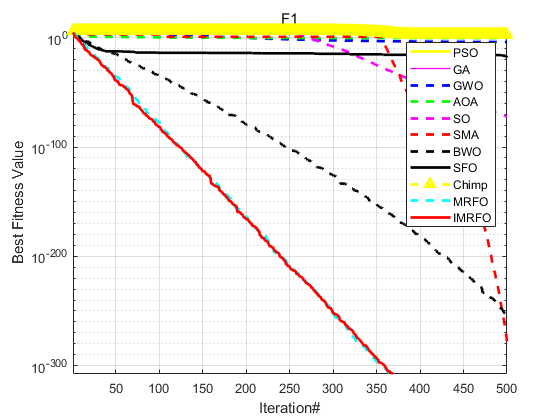

Supplement: Supplementary file 1 — Supplementary Information. [file 41598_2024_59960_MOESM1_ESM.zip › All research figures/All research figures/1 Figures of benchmark functions/Figures of all benchmark functions/1/1-4.tif]

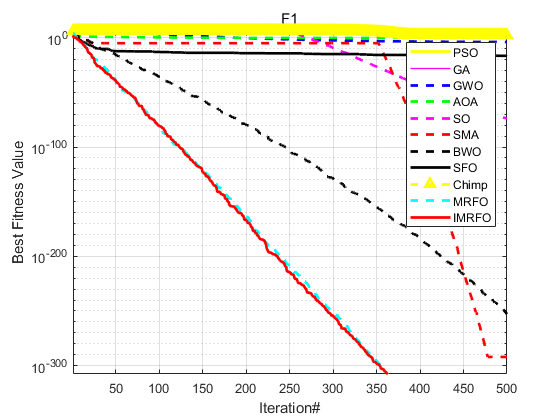

Supplement: Supplementary file 1 — Supplementary Information. [file 41598_2024_59960_MOESM1_ESM.zip › All research figures/All research figures/1 Figures of benchmark functions/Figures of all benchmark functions/1/1-5.tif]

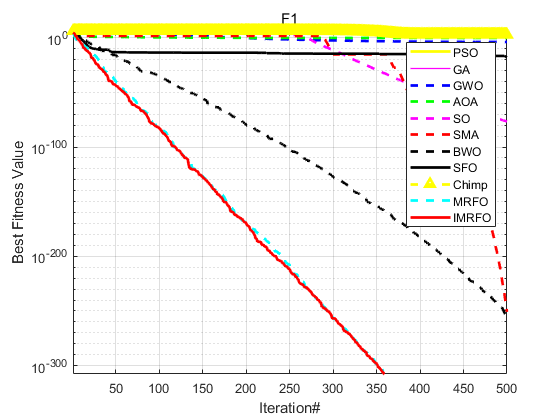

Supplement: Supplementary file 1 — Supplementary Information. [file 41598_2024_59960_MOESM1_ESM.zip › All research figures/All research figures/1 Figures of benchmark functions/Figures of all benchmark functions/1/1-6.tif]

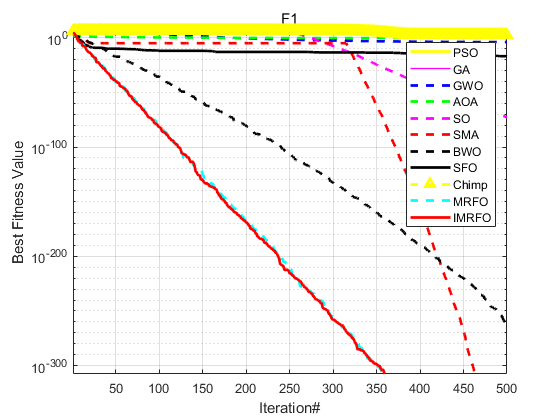

Supplement: Supplementary file 1 — Supplementary Information. [file 41598_2024_59960_MOESM1_ESM.zip › All research figures/All research figures/1 Figures of benchmark functions/Figures of all benchmark functions/1/1-7.tif]

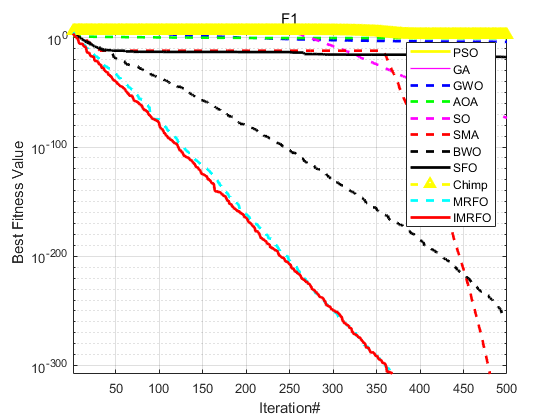

Supplement: Supplementary file 1 — Supplementary Information. [file 41598_2024_59960_MOESM1_ESM.zip › All research figures/All research figures/1 Figures of benchmark functions/Figures of all benchmark functions/1/1-8.tif]

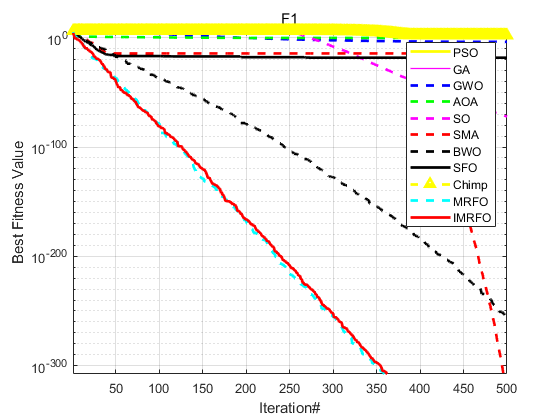

Supplement: Supplementary file 1 — Supplementary Information. [file 41598_2024_59960_MOESM1_ESM.zip › All research figures/All research figures/1 Figures of benchmark functions/Figures of all benchmark functions/1/1-9.tif]

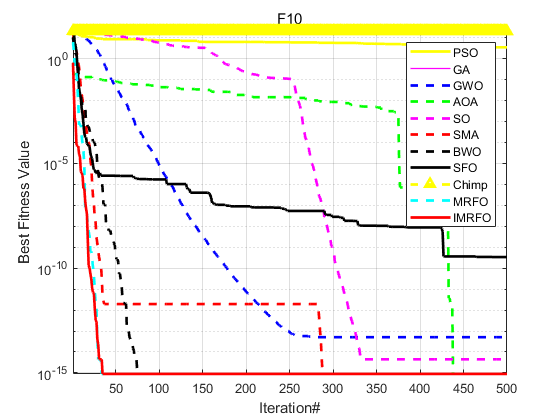

Supplement: Supplementary file 1 — Supplementary Information. [file 41598_2024_59960_MOESM1_ESM.zip › All research figures/All research figures/1 Figures of benchmark functions/Figures of all benchmark functions/10/10-1.tif]

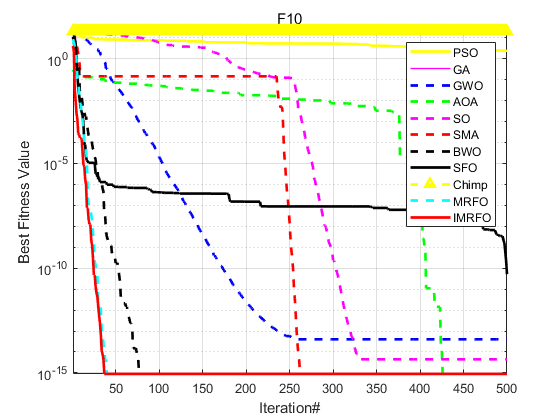

Supplement: Supplementary file 1 — Supplementary Information. [file 41598_2024_59960_MOESM1_ESM.zip › All research figures/All research figures/1 Figures of benchmark functions/Figures of all benchmark functions/10/10-10.tif]

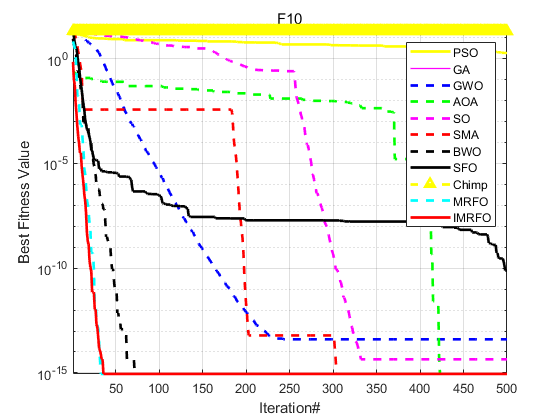

Supplement: Supplementary file 1 — Supplementary Information. [file 41598_2024_59960_MOESM1_ESM.zip › All research figures/All research figures/1 Figures of benchmark functions/Figures of all benchmark functions/10/10-11.tif]

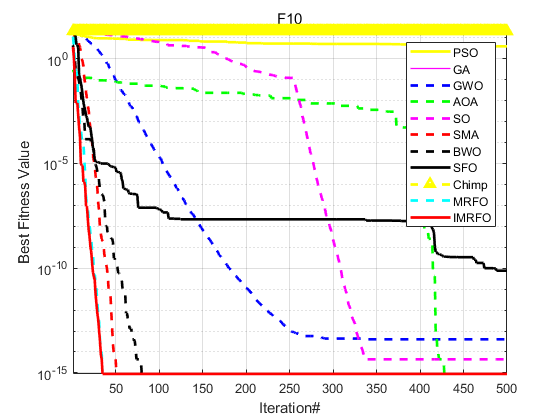

Supplement: Supplementary file 1 — Supplementary Information. [file 41598_2024_59960_MOESM1_ESM.zip › All research figures/All research figures/1 Figures of benchmark functions/Figures of all benchmark functions/10/10-12.tif]

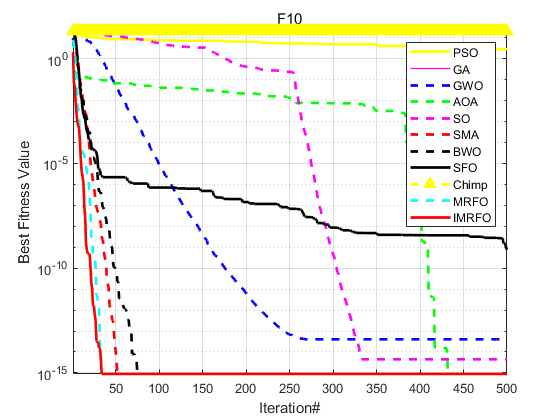

Supplement: Supplementary file 1 — Supplementary Information. [file 41598_2024_59960_MOESM1_ESM.zip › All research figures/All research figures/1 Figures of benchmark functions/Figures of all benchmark functions/10/10-13.tif]

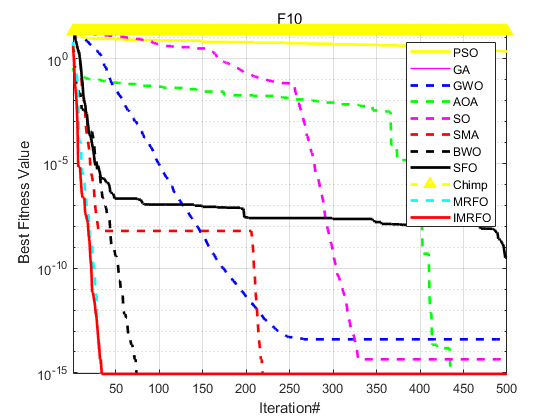

Supplement: Supplementary file 1 — Supplementary Information. [file 41598_2024_59960_MOESM1_ESM.zip › All research figures/All research figures/1 Figures of benchmark functions/Figures of all benchmark functions/10/10-14.tif]

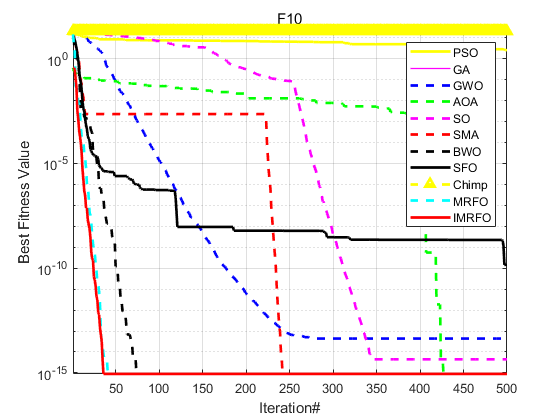

Supplement: Supplementary file 1 — Supplementary Information. [file 41598_2024_59960_MOESM1_ESM.zip › All research figures/All research figures/1 Figures of benchmark functions/Figures of all benchmark functions/10/10-15.tif]

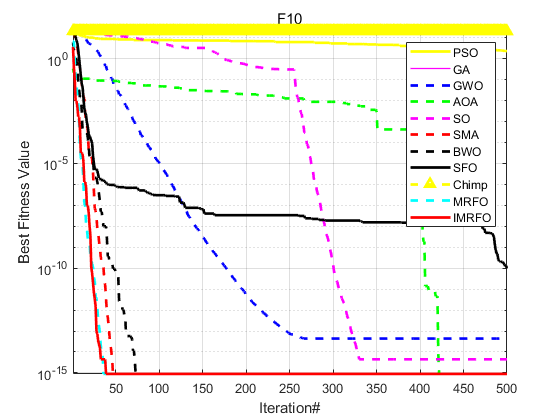

Supplement: Supplementary file 1 — Supplementary Information. [file 41598_2024_59960_MOESM1_ESM.zip › All research figures/All research figures/1 Figures of benchmark functions/Figures of all benchmark functions/10/10-16.tif]

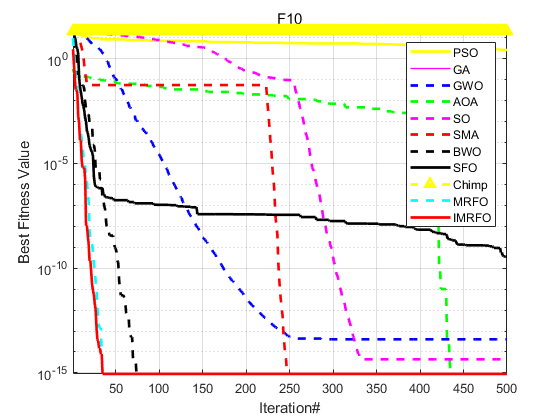

Supplement: Supplementary file 1 — Supplementary Information. [file 41598_2024_59960_MOESM1_ESM.zip › All research figures/All research figures/1 Figures of benchmark functions/Figures of all benchmark functions/10/10-17.tif]

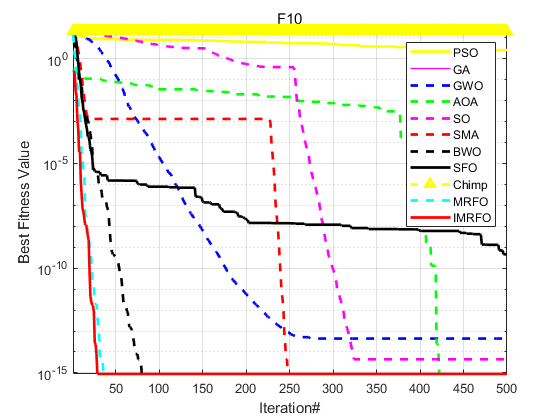

Supplement: Supplementary file 1 — Supplementary Information. [file 41598_2024_59960_MOESM1_ESM.zip › All research figures/All research figures/1 Figures of benchmark functions/Figures of all benchmark functions/10/10-18.tif]

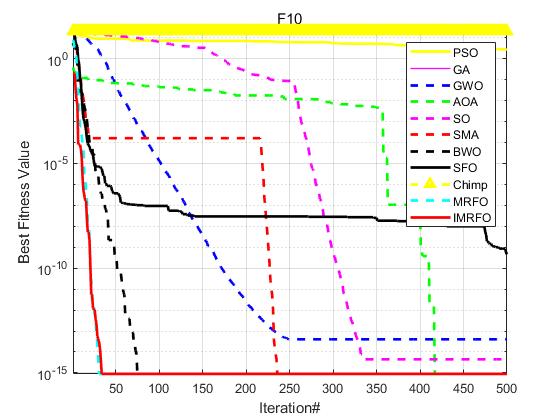

Supplement: Supplementary file 1 — Supplementary Information. [file 41598_2024_59960_MOESM1_ESM.zip › All research figures/All research figures/1 Figures of benchmark functions/Figures of all benchmark functions/10/10-19.tif]

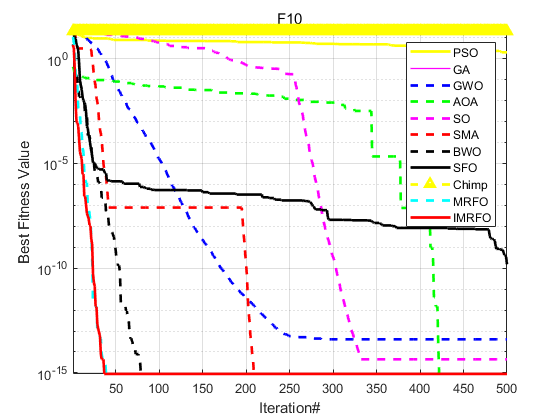

Supplement: Supplementary file 1 — Supplementary Information. [file 41598_2024_59960_MOESM1_ESM.zip › All research figures/All research figures/1 Figures of benchmark functions/Figures of all benchmark functions/10/10-2.tif]

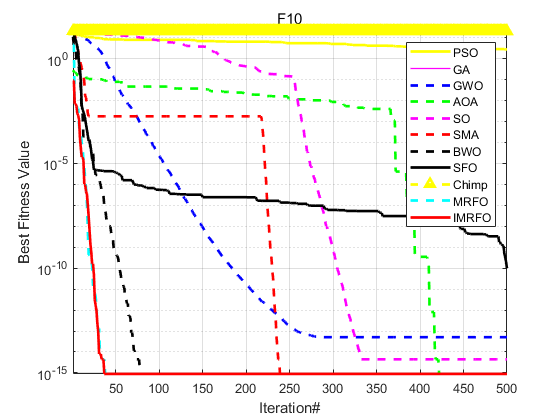

Supplement: Supplementary file 1 — Supplementary Information. [file 41598_2024_59960_MOESM1_ESM.zip › All research figures/All research figures/1 Figures of benchmark functions/Figures of all benchmark functions/10/10-20.tif]

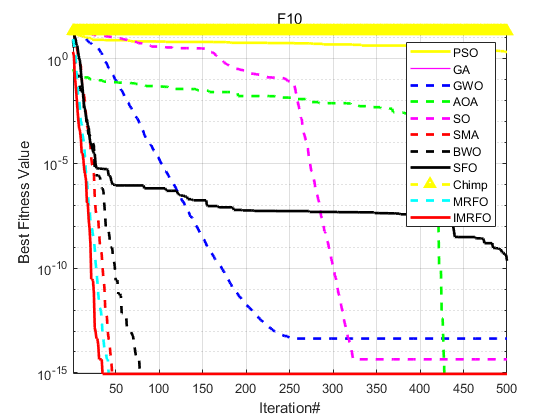

Supplement: Supplementary file 1 — Supplementary Information. [file 41598_2024_59960_MOESM1_ESM.zip › All research figures/All research figures/1 Figures of benchmark functions/Figures of all benchmark functions/10/10-21.tif]

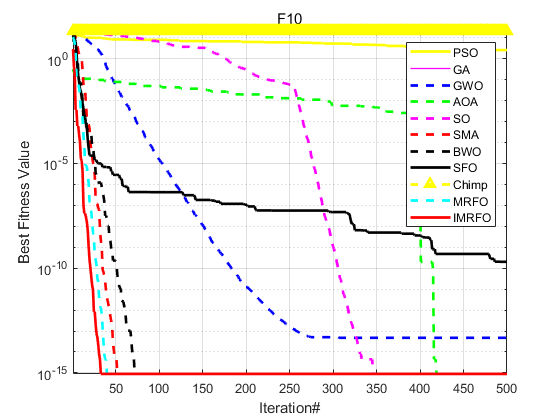

Supplement: Supplementary file 1 — Supplementary Information. [file 41598_2024_59960_MOESM1_ESM.zip › All research figures/All research figures/1 Figures of benchmark functions/Figures of all benchmark functions/10/10-22.tif]

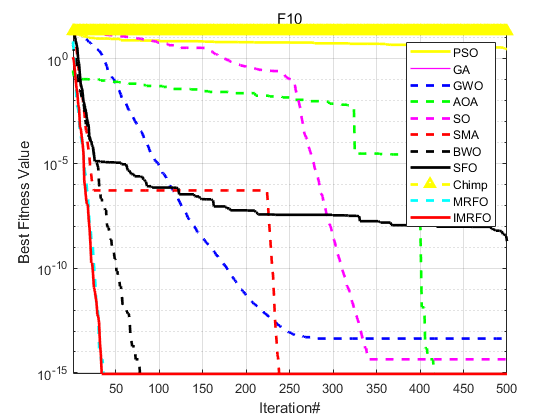

Supplement: Supplementary file 1 — Supplementary Information. [file 41598_2024_59960_MOESM1_ESM.zip › All research figures/All research figures/1 Figures of benchmark functions/Figures of all benchmark functions/10/10-23.tif]

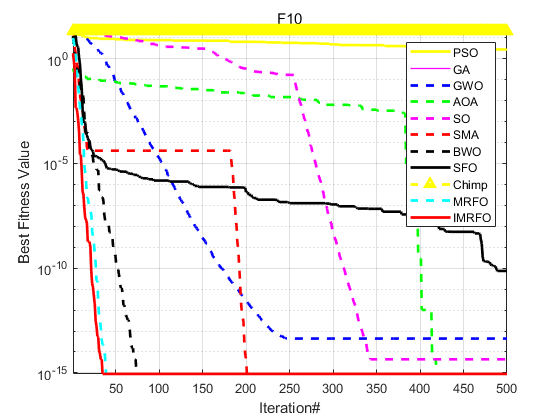

Supplement: Supplementary file 1 — Supplementary Information. [file 41598_2024_59960_MOESM1_ESM.zip › All research figures/All research figures/1 Figures of benchmark functions/Figures of all benchmark functions/10/10-24.tif]

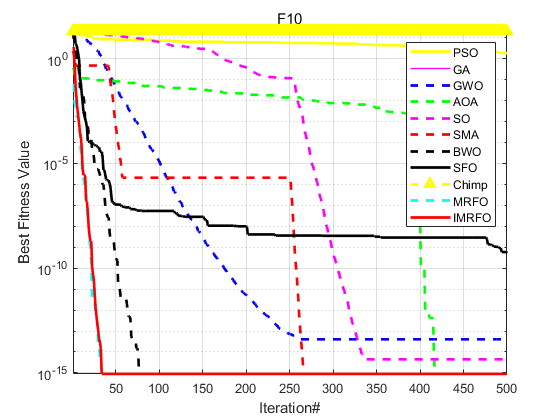

Supplement: Supplementary file 1 — Supplementary Information. [file 41598_2024_59960_MOESM1_ESM.zip › All research figures/All research figures/1 Figures of benchmark functions/Figures of all benchmark functions/10/10-25.tif]

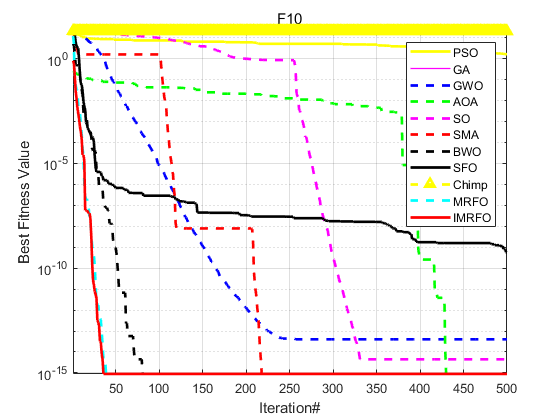

Supplement: Supplementary file 1 — Supplementary Information. [file 41598_2024_59960_MOESM1_ESM.zip › All research figures/All research figures/1 Figures of benchmark functions/Figures of all benchmark functions/10/10-26.tif]

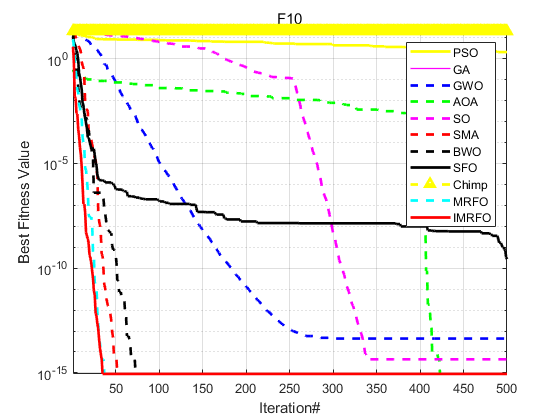

Supplement: Supplementary file 1 — Supplementary Information. [file 41598_2024_59960_MOESM1_ESM.zip › All research figures/All research figures/1 Figures of benchmark functions/Figures of all benchmark functions/10/10-27.tif]

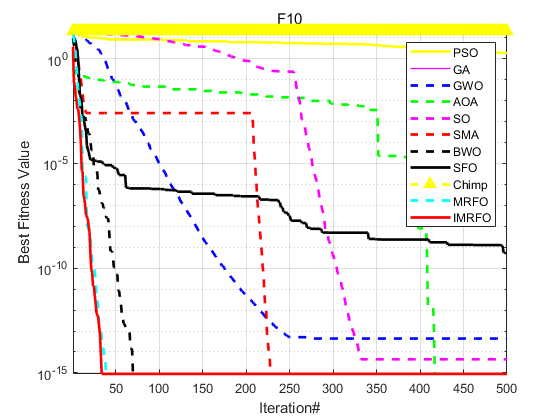

Supplement: Supplementary file 1 — Supplementary Information. [file 41598_2024_59960_MOESM1_ESM.zip › All research figures/All research figures/1 Figures of benchmark functions/Figures of all benchmark functions/10/10-28.tif]

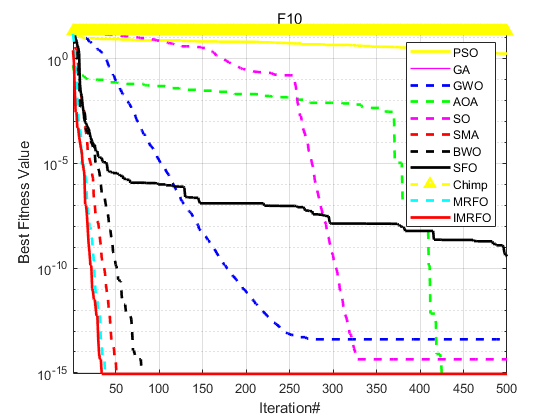

Supplement: Supplementary file 1 — Supplementary Information. [file 41598_2024_59960_MOESM1_ESM.zip › All research figures/All research figures/1 Figures of benchmark functions/Figures of all benchmark functions/10/10-29.tif]

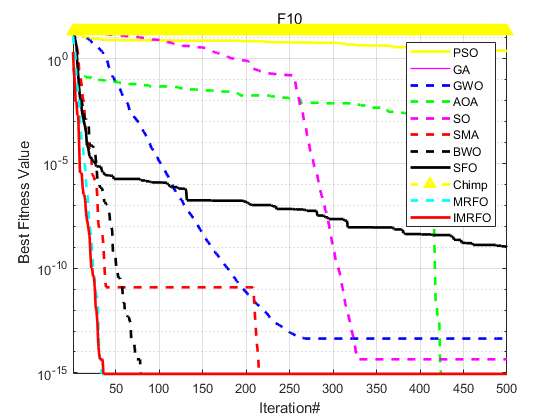

Supplement: Supplementary file 1 — Supplementary Information. [file 41598_2024_59960_MOESM1_ESM.zip › All research figures/All research figures/1 Figures of benchmark functions/Figures of all benchmark functions/10/10-3.tif]

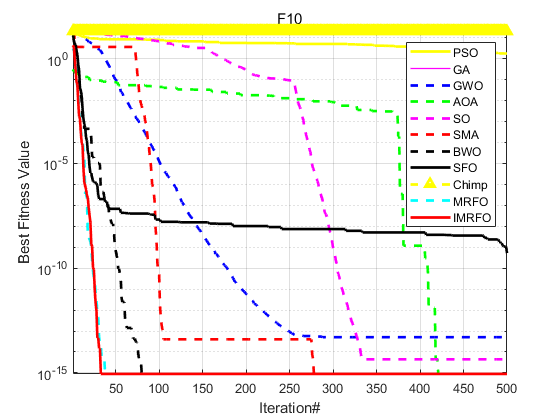

Supplement: Supplementary file 1 — Supplementary Information. [file 41598_2024_59960_MOESM1_ESM.zip › All research figures/All research figures/1 Figures of benchmark functions/Figures of all benchmark functions/10/10-30.tif]
