# Supplementary material for: An improved manta ray foraging optimization algorithm
Source: Sci Rep. 2024 May 5;14:10301. doi: 10.1038/s41598-024-59960-1 (PMC11070432; doi:10.1038/s41598-024-59960-1)
Supplement: Supplementary file 1 — Supplementary Information. [file 41598_2024_59960_MOESM1_ESM.zip › All research figures/All research figures/Instructions for all figures in the research.docx]

Instructions for all figures in the research

To increase the experiment's credibility in our research, we tested IMRFO and 10 other state-of-the-art algorithms under the same test environment on 23 benchmark functions, the CEC 2017 and CEC 2022 benchmark suites. Additionally, we tested three variants of IMRFO and MRFO on 23 benchmark functions simultaneously to analyze the performance of the IMRFO modifications. During the test process on the 23 benchmark functions, F1-F13 refer to Dim=30, and F14-F23 are fixed dimensions. For the CEC 2017 and CEC2022 benchmark suites, all functions are Dim=30, all algorithms run 30 times. During the test process on the 5 engineering problems, we employed IMRFO to solve these problems with 10 mentioned algorithms and other new algorithms, and all algorithms run 30 times as well, and each experimental figure are recoded. There are 5 documents of figures in our research.

1 Figures of benchmark functions

2 Figures of CEC2017 functions

3 Figures of CEC2022 functions

4 Figures of modifications

5 Figures of engineering problems
