# Supplementary material for: An improved manta ray foraging optimization algorithm
Source: Sci Rep. 2024 May 5;14:10301. doi: 10.1038/s41598-024-59960-1 (PMC11070432; doi:10.1038/s41598-024-59960-1)
Supplement: Supplementary file 1 — Supplementary Information. [file 41598_2024_59960_MOESM1_ESM.zip › Instructions for all data and figures in the research.docx]

This file rar package contains all the figures and data in IMRFO research. There are two instructions in each data and figures documents,respectively.
