# Supplementary material for: An improved manta ray foraging optimization algorithm
Source: Sci Rep. 2024 May 5;14:10301. doi: 10.1038/s41598-024-59960-1 (PMC11070432; doi:10.1038/s41598-024-59960-1)
Supplement: Supplementary file 1 — Supplementary Information. [file 41598_2024_59960_MOESM1_ESM.zip › Research Data/Research Data/Instructions for all data in the research.docx]

Instructions for all data in the research

To increase the experiment's credibility in our research, we tested IMRFO and 10 other state-of-the-art algorithms under the same test environment on 23 benchmark functions, the CEC 2017 and CEC 2022 benchmark suites. Additionally, we tested IMRFO with 10 latest enhanced algorithms on 23 benchmark functions to analyze the performance of the IMRFO compared with other enhanced algorithms, and then tested three variants of IMRFO and MRFO on 23 benchmark functions simultaneously to analyze the performance of the IMRFO modifications. During the test process on the 23 benchmark functions, F1-F13 refer to Dim=30, and F14-F23 are fixed dimensions. For the CEC 2017 and CEC2022 benchmark suites, all functions are Dim=30, all algorithms run 30 times. During the test process on the 5 engineering problems, we employed IMRFO to solve these problems with 10 mentioned algorithms and other new algorithms, and all algorithms run 30 times as well, and each experimental data are recoded.

The best (Best), average (Mean), and standard deviation (Std) were recorded in the tables, respectively.

There are 10 documents and files of data in our research.

We tested IMRFO and 10 other state-of-the-art algorithms on benchmark functions and recoded in file ‘1 Benchmark functions data’ and document ‘1.1 Benchmark-functions-box-data’.

We tested IMRFO and 10 other 10 latest enhanced algorithms on benchmark functions and recoded in file ‘2 Results of IMRFO compared with other enhanced algorithms’.

We tested IMRFO and 10 other state-of-the-art algorithms on CEC2017 function suite and recoded in file‘3 CEC2017 data’ and document ‘3.1 CEC2017-box-data’.

We tested IMRFO and 10 other state-of-the-art algorithms on CEC2022 function suite and recoded in file ‘4 CEC2022 data’ and document ‘4.1 CEC2022-box-data’.

We tested three variants of IMRFO and MRFO on 23 benchmark functions simultaneously to analyze the performance of the IMRFO modifications and recoded in file ‘5 The comparison and results on benchmark functions of IMRFO and modifications’.

We report the significant differences between our proposed IMRFO and the other algorithms in most functions and recoded when significance level value is set at 0.05 in file ‘6 Wilcoxon’s rank sum test’.

We employed IMRFO to solve these problems with 10 mentioned algorithms and other new algorithms and recoded in file ‘7 Engineering problems-data’.
